# Multicatalytic Access to Renewable Poly(Silyl Ether)s with Tunable Properties

**DOI:** 10.1002/anie.202521137

**Published:** 2026-01-14

**Authors:** Fan Yang, Fan Sun, Christophe M. Thomas

**Affiliations:** ^1^ Institut de Recherche de Chimie Paris, CNRS, Chimie ParisTech PSL University Paris 75005 France

**Keywords:** Bio‐based polymers, Degradable, Multicatalytic system, One‐pot polymerization, Poly(silyl ether)s

## Abstract

The global reliance on petroleum‐based polymers presents urgent sustainability challenges. Addressing this issue, we present a novel one‐pot multicatalytic strategy for synthesizing partially bio‐based poly(silyl ether)s with tunable properties. This method combines magnesium‐catalyzed esterification of bio‐based diacids and alcohols with borane‐catalyzed hydrosilylation under mild conditions. This approach enables direct incorporation of ester functionalities into the polymer backbone, affording high‐molecular‐weight poly(silyl ether)s with tunable architectures and thermal profiles. The method demonstrates excellent catalyst compatibility and scalability, significantly reducing purification steps while broadening monomer scope. Beyond thermal robustness, the resulting materials exhibit remarkable mechanical performance. Preliminary tensile tests revealed distinctive deformation behavior, with certain polymers achieving extraordinary extensibility (elongation at break > 3800%) and high energy absorption, attributed to the synergistic interplay between flexible siloxane segments and dynamic silyl ether linkages. Degradation studies confirm efficient chemical recycling, underscoring the potential of this one‐pot process to deliver sustainable, high‐performance polymers with customizable properties for advanced applications.

## Graphical abstract

This work presents a one‐pot, multicatalytic approach that combines magnesium‐catalyzed esterification and borane‐mediated hydrosilylation for the synthesis of partially bio‐based poly(silyl ethers). This method allows for the tuning of thermal and mechanical properties, including exceptional extensibility (>3800%), and enables efficient chemical recycling, thus advancing sustainable polymer design.

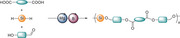

## Introduction

Polymers have transformed modern society, enabling innovations across construction, packaging, aerospace, electronics, and countless other sectors.^[^ ^1^, ^2^ ^]^ However, overwhelming reliance on petroleum‐based monomers, non‐renewable resources, raises serious environmental concerns.^[^ ^3^ ^]^ Alarmingly, nearly one‐third of all plastic waste ends up in marine or terrestrial ecosystems, threatening biodiversity and human health.^[^ ^4^ ^]^ In response to these challenges, the development of sustainable polymers derived from renewable feedstocks has emerged as a critical research frontier.^[^ ^5^, ^6^ ^]^ Despite increasing interest, bio‐based polymers currently account for only about 5% of the global market, hindered by high production costs and limited scalability. To accelerate their industrial adoption, it is imperative to establish cost‐effective and efficient synthetic strategies that align with principles of green chemistry and the circular economy.^[^ ^3^, ^7^ ^]^

One‐pot multicatalytic synthesis represents a promising strategy inspired by the efficiency and complexity of enzymatic cascades in nature.^[^ ^8^, ^9^ ^]^ This approach enables multiple catalytic transformations to proceed either sequentially or concurrently within a single reaction vessel, significantly reducing purification steps, minimizing waste, and enhancing overall process efficiency.^[^ ^10^, ^11^ ^]^ By judiciously selecting and combining compatible catalysts, these systems can be tailored to yield polymers with desirable attributes such as improved thermal stability, mechanical strength, flexibility, and (bio)degradability. However, the design of effective one‐pot multicatalytic systems remains inherently challenging.^[^ ^12^ ^]^ Each catalytic transformation may require distinct reaction conditions,^[^ ^13^ ^]^ and incompatibilities among catalysts, solvents, substrates, or by‐products can compromise activity or lead to undesired side reactions.^[^ ^6^ ^]^ Achieving high selectivity and efficiency thus demands meticulous planning of catalyst compatibility and reaction sequence from the outset.^[^ ^14^ ^]^

**Scheme 1:**
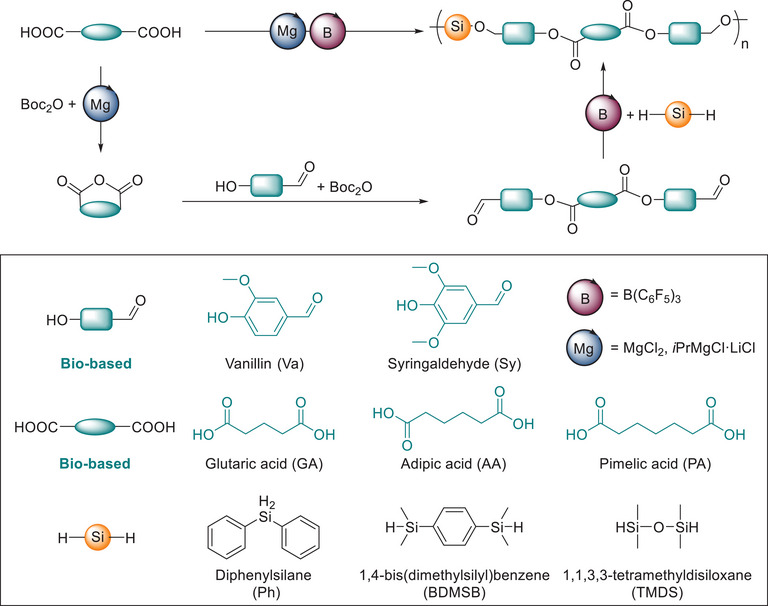
One‐pot multicatalytic synthesis of poly(silyl ether)s from renewable feedstocks.

Poly(silyl ether)s (PSEs) and poly(siloxanes) are recognized as high‐performance polymers, valued for their exceptional flexibility, thermal stability, and chemical resistance.^[^ ^15^ ^]^ While both classes share advantageous properties, PSEs are distinguished by their unique Si─O─C linkages, which impart sensitivity to acidic and basic hydrolysis, as well as alcoholysis.^[^ ^16^, ^17^ ^]^ This structural feature enables enhanced degradability and recyclability,^[^ ^18^ ^]^ making PSEs particularly attractive for sustainable material design. The performance of PSEs can be further tuned by introducing sterically bulky substituents around the silyl ether bond, which improves mechanical strength and thermal robustness.^[^ ^19^ ^]^ Given the relevance of this polymer architecture, various synthetic strategies have been developed to access PSEs.^[^ ^20^, ^21^, ^22^ ^]^ Among these, the hydrosilylation of dialdehydes with dihydrosilanes has emerged as a widely adopted and efficient method (Scheme 1).

Transition metal complexes, such as those based on ruthenium,^[^ ^23^ ^]^ rhodium,^[^ ^24^ ^]^ palladium,^[^ ^18^ ^]^ zinc,^[^ ^25^ ^]^ copper,^[^ ^26^ ^]^ and manganese,^[^ ^27^ ^]^ have been extensively employed for poly(silyl ether) synthesis. Despite their effectiveness, these systems often require harsh reaction conditions and are prone to side reactions. Moreover, their reliance on expensive and/or scarce metals poses significant limitations in terms of scalability and sustainability, particularly within the framework of green chemistry. In contrast, tris(pentafluorophenyl)borane [B(C_6_F_5_)_3_] offers a metal‐free alternative for the synthesis of functional polymers. This Lewis acid catalyst is not only a water‐tolerant catalyst but also highly efficient in promoting hydrosilylation reactions under mild conditions, making it an attractive candidate for environmentally friendly polymer synthesis.^[^ ^28^ ^]^ Additionally, our group has demonstrated that magnesium‐based catalysts, in combination with di‐*tert*‐butyl dicarbonate (Boc_2_O), enable efficient ester formation under mild conditions, expanding the monomer scope for sustainable polymer synthesis.^[^ ^6^, ^29^ ^]^

Building on this foundation, we report a one‐pot multicatalytic strategy for synthesizing PSEs from partially bio‐based monomers (Scheme 1). This dual‐catalyst approach, combining magnesium‐catalyzed esterification of renewable diacids and alcohols with borane‐catalyzed hydrosilylation, enables the direct incorporation of degradable ester functionalities into the polymer backbone.^[^ ^30^ ^]^ The method significantly expands the monomer scope and delivers high‐performance PSEs with tailored properties.

## Results and Discussion

### Rationalization of Reaction Conditions

To evaluate the catalytic efficiency of the magnesium/Boc_2_O system, vanillin and syringaldehyde, which are derived from lignin depolymerization, were selected as bio‐based substrates.^[^ ^31^ ^]^ Using vanillin and adipic acid as a model system, esterification proceeded to full conversion under mild conditions (Table 1), confirming the system's effectiveness and compatibility with biomass‐derived compounds.

**Table 1:** Ester formation from vanillin and adipic acid.^a)^

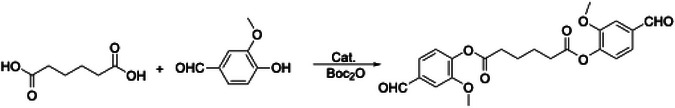				
Entry	Solvent	Catalyst	Temp. (°C)	Time (h)
1	CH_3_CN	MgCl_2_	50	20
2	Toluene	MgCl_2_	50	22.5
3	THF	MgCl_2_	50	47.5
4	THF	*i*PrMgCl·LiCl	50	5
5	THF	*i*PrMgCl·LiCl	40	46
6^b)^	THF	*i*PrMgCl·LiCl	60	5

^a)^ All reactions were performed under argon, with [AA] = 1 eq, [Va] = 2.0 eq, [Boc_2_O] = 4.4 eq, [Cat] = 4 mol% until completion as determined by the integration of the ^1^H NMR aldehyde resonances of vanillin and AA‐Va.

^b)^ Conversion = 68%.

To optimize reaction conditions, the effect of the solvent on the model reaction was investigated. Using MgCl_2_ as a catalyst (4 mol%) under equimolar conditions in acetonitrile, complete conversion was achieved within 20 h at 50 °C (Table 1, Entry 1). In comparison, the same reaction required 22.5 h in toluene (Table 1, Entry 2) and 47.5 h in THF (Table 1, Entry 3), highlighting the significant impact of solvent polarity and coordination ability on reaction kinetics. To further enhance the reaction efficiency, the well‐defined complex (CH_3_)_2_CHMgCl·LiCl, commonly referred to as the turbo Grignard reagent, was employed, achieving full conversion in just 5 h in THF (Table 1, Entry 4). This improvement is attributed to its superior solubility and reactivity in organic solvents. The influence of temperature on the reaction was also investigated. Lowering the temperature to 40 °C markedly prolonged the reaction time to 46 h (Table 1, Entry 5). Conversely, increasing the temperature to 60 °C resulted in a reduced conversion of 68% (Table 1, Entry 6), which can likely be attributed to the thermal decomposition of Boc_2_O.^[^ ^32^ ^]^ Overall, the use of *i*PrMgCl·LiCl in THF demonstrates both practical and economic advantages, offering valuable insights into solvent and reagent selection for optimizing ester formation.

Building on the optimized reaction conditions, we investigated the synthesis of dialdehydes using bio‐based phenols (i.e., vanillin (Va) and syringaldehyde (Sy)) as key building blocks. These were reacted with renewable dicarboxylic acids^[^ ^33^, ^34^, ^35^ ^]^ of varying carbon chain lengths: glutaric acid (GA), adipic acid (AA), and pimelic acid (PA) (Table 2). Reaction times varied significantly depending on the acid‐alcohol combination. For example, Va reacted efficiently with GA, reaching full conversion in 20 h (Table 2, Entry 1), whereas the reaction of Sy with GA required 70 h (Table 2, Entry 2), likely due to increased steric hindrance and reduced solubility associated with the additional methoxy group in syringaldehyde. A similar trend was observed with AA and PA. Vanillin reacted rapidly with adipic acid (5 h), while the reaction of Sy with AA extended to 16 h (Table 2, Entries 3, 4). Pimelic acid exhibited intermediate reactivity with Va, reaching full conversion in 15 h, but its reaction with syringaldehyde was markedly slower, requiring 139 h (Table 2, Entries 5, 6). These results further underscore the impact of steric hindrance and solubility on reaction kinetics. The structures of the synthesized dialdehydes were confirmed by ^1^H NMR and ^13^C NMR spectroscopy, with detailed spectra provided in Figures S1–S12 for GA‐Va, GA‐Sy, AA‐Va, AA‐Sy, PA‐Va, and PA‐Sy.

**Table 2:** Ester formation from various phenols and dicarboxylic acids.^a)^

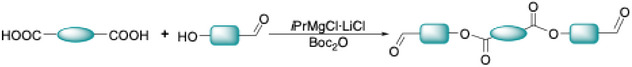			
Entry	Acid	Alcohol	Time(h)
1	GA	Va	20
2	GA	Sy	70
3	AA	Va	5
4	AA	Sy	16
5	PA	Va	15
6	PA	Sy	139

^a)^ All reactions were performed under argon, with [Acid] = 1 eq, [Va] = 2.0 eq, [Boc_2_O] = 4.4 eq, [Cat] = 4 mol% in THF at 50 °C until completion as determined by the integration of the ^1^H NMR aldehyde resonances of phenol and corresponding dialdehyde.

### One‐Pot Synthesis of Poly(silyl ether)s

With an efficient monomer synthesis established, a one‐pot step‐growth polymerization strategy was employed to synthesize poly(silyl ether)s. The process began with the formation of an ester, for which adipic acid (0.5 mmol), vanillin (1.0 mmol), Boc_2_O (1.2 mmol), and *i*PrMgCl·LiCl (20 µmol) were dissolved in anhydrous THF (1.0 mL). The mixture was stirred at 50 °C for 5 h until complete. After removing the solvent from the ester formation step, 0.5 mL of dichloromethane (DCM) and 0.5 mmol of diphenylsilane (Ph_2_SiH_2_) were added. Hydrosilylation was then catalyzed by 2 mol% of B(C_6_F_5_)_3_ at room temperature for 24 h. The resulting polymer exhibited a weight‐average molecular weight (*M*_w_) of 7000 g mol**^−1^** and a dispersity (*Đ*) of 1.52 (Table 3, Entry 1).

**Table 3:** Optimization of the B(C_6_F_5_)_3_‐catalyzed dehydrocoupling polymerization of bis(4‐formyl‐2‐methoxyphenyl) adipate with diphenylsilane.^a)^

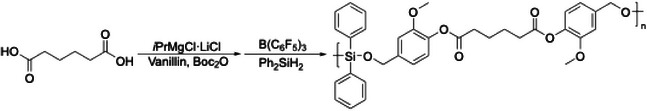					
Entry	Solvent	B(C_6_F_5_)_3_(mol%)	*M*_n_(g mol^−1^)^b)^	*M*_w_(g mol^−1^)^b)^	*Đ* ^b)^
1	DCM	2	4600	7000	1.52
2	THF	2	–	–	–
3	Anisole	2	–	–	–
4	Toluene	2	2400	4200	1.75
5	Toluene	1	17 000	41 900	2.46
6	Toluene	0.5	10 100	17 700	1.75

^a)^ All reactions were performed under argon, ester formation was carried out in THF at 50 °C, and polymerization was performed at room temperature for 24 h.

^b)^ *M*_n_, *M*_w_, and *Đ* of polymer determined by SEC‐RI in THF calibrated with polystyrene standards at 35 °C.

**Figure 1:**
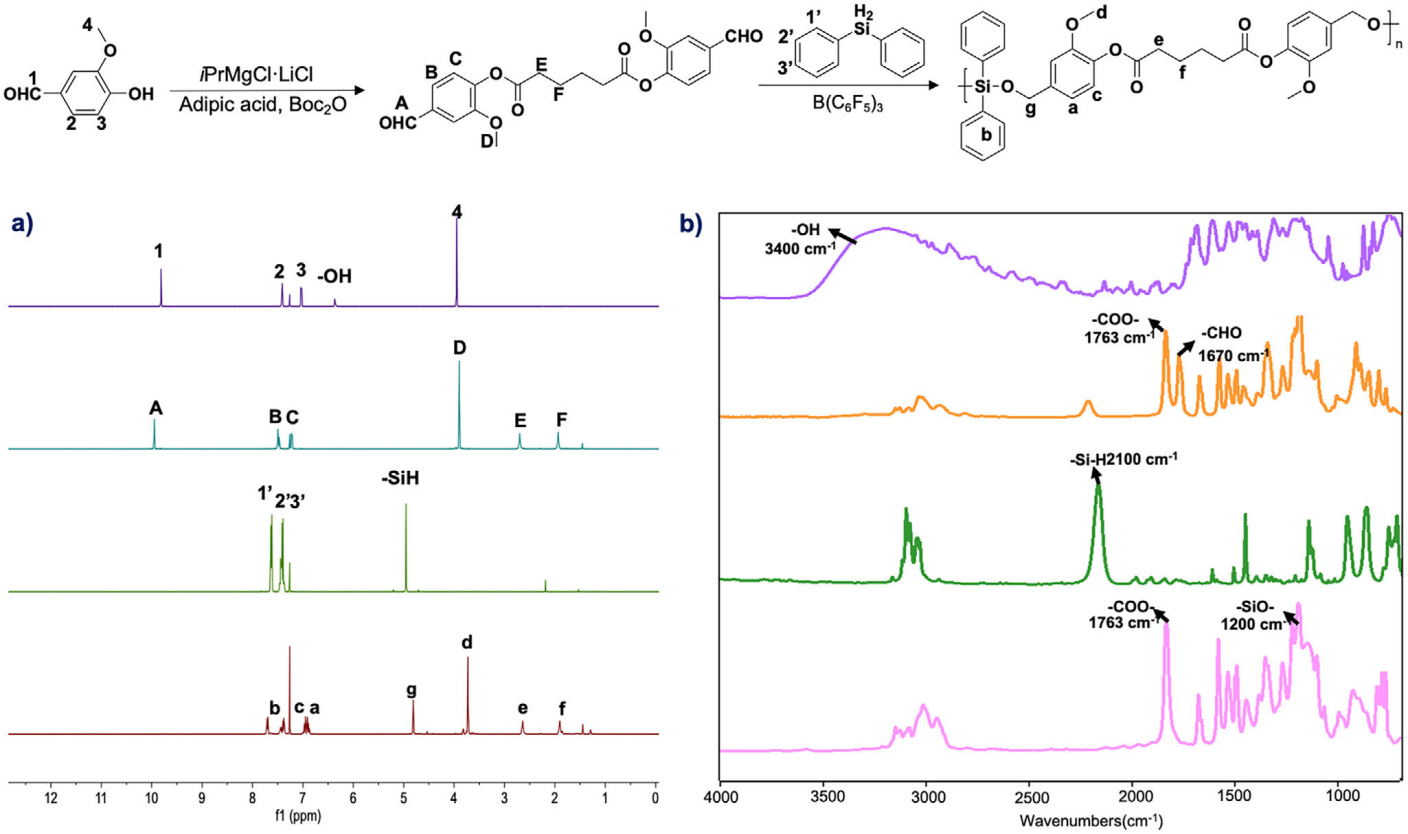
a) ^1^H NMR spectra (400 MHz, CDCl_3_) of vanillin, AA‐Va, Ph_2_SiH_2_, and poly(AA‐Va‐*co*‐Ph) (from top to bottom); b) FT‐IR spectra of Vanillin, AA‐Va, Ph_2_SiH_2_, and poly(AA‐Va‐*co*‐Ph) (from top to bottom).

The formation of the resulting PSE was confirmed by ^1^H and ^29^Si NMR, as well as FT‐IR spectroscopy (Figure 1). Complete disappearance of hydroxy (δ = 6.25 ppm in ^1^H NMR, ν = 3360 cm^−1^ in FT‐IR) and aldehyde (9.95 ppm in ^1^H NMR, 1700 cm^−1^ in FT‐IR) signals of vanillin indicated full conversion of the dialdehyde precursor. In addition, the absence of Si─H signals (4.93 ppm in ^1^H NMR, 2300 cm^−1^ in FT‐IR) coupled with the emergence of a methylene proton resonance at *δ* = 4.81 ppm and a strong ester carbonyl absorption band at *ν* = 1763 cm^−1^ confirmed successful ester formation.^[^ ^36^ ^]^ These results demonstrate the high compatibility between the magnesium catalyst used for the ester formation and the boron‐based catalyst for hydrosilylation, validating the efficiency of the multicatalytic one‐pot approach for PSE synthesis (Scheme S1).

To optimize the reaction conditions, the influence of various solvents was systematically investigated. Reactions conducted in THF and anisole showed no conversion, likely due to the poor compatibility of the highly reactive Lewis acid catalyst B(C_6_F_5_)_3_ with ether functionalities (Table 3, Entries 2, 3).^[^ ^37^, ^38^, ^39^ ^]^ Considering both reactivity and safety, toluene was chosen as an alternative solvent. Under identical reaction conditions, polymerization proceeded in toluene, yielding a polymer with a lower molecular weight (*M*_w _= 4200 g mol**^−1^**, *Đ* = 1.75, Table 3, Entry 4). The observed reduction in molecular weight is likely attributable to the lower solubility of the monomer in toluene compared to dichloromethane. Limited solubility can reduce the effective conversion and induce a temporary stoichiometric imbalance in the liquid phase. This imbalance leads to an excess of functional groups, which consequently restricts chain growth, thereby limiting the achievable molecular weight. Optimizing the catalyst loading revealed that reducing the catalyst loading to 1 mol% significantly increased molecular weight (*M*_w_ = 41,900 g mol**^−1^**, Table 3, Entry 5), though with broader dispersity (*Đ* = 2.46), suggesting reduced control over the polymerization process. A further reduction in catalyst loading to 0.5 mol% yielded a more balanced outcome (*M*_w_ = 17,700 g mol**^−1^**, *Đ* = 1.75), indicating a trade‐off between molecular weight and polymerization control (Table 3, Entry 6). Optimized conditions were established by using toluene at room temperature for 24 h, with a molar ratio of [AA‐Va]:[Ph]:[B(C_6_F_5_)_3_] = 100:100:1.

**Figure 2:**
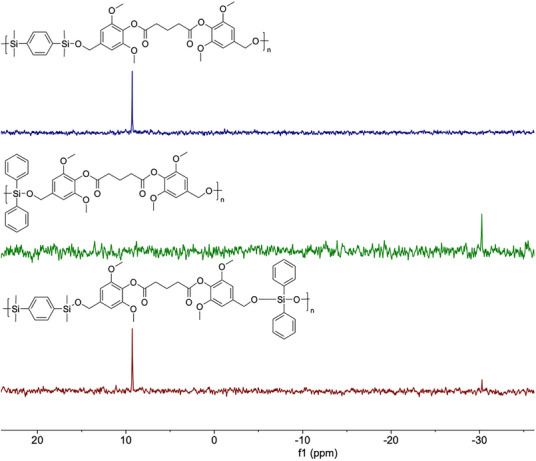
^29^Si NMR spectra (99 MHz, CDCl_3_) of poly(GA‐Sy‐*co*‐BDMSB), poly(GA‐Sy‐*co*‐Ph), and poly(GA‐Sy‐*co*‐BDMSB‐*r*‐Ph) (from top to bottom).

Following reaction optimization, the scope of the one‐pot polymerization was explored using a range of commercially available bis(hydrosilane) monomers [diphenylsilane (Ph), tetramethyldisiloxane (TMDS), and 1,4‐bis(dimethylsilyl)benzene (BDMSB)] in combination with three bio‐based diacids (GA, AA, PA) and two lignin‐derived aldehydes (vanillin, syringaldehyde) (Scheme 1). This modular approach enabled the synthesis of PSEs with well‐defined molecular architectures, as confirmed by NMR and FT‐IR spectroscopy (Figures S13–S92, S140–S147). The resulting polymers exhibited high molecular weights (*M*_w_ > 10,000 g mol**^−1^**, Table 4), demonstrating the efficiency and versatility of the method. To further expand structural diversity, the synthesis of copolymers was investigated using equimolar mixtures of different dihydrosilanes. For example, copolymerization of GA‐Sy with Ph_2_SiH_2_ and BDMSB yielded the expected copolymer with *M*_w_ = 25 100 g mol**^−1^** and *Đ* = 1.57 (Table 4, Entry 19). The presence of two distinct ^29^Si NMR signals at δ = 9.28, −30.27 ppm confirmed incorporation of both silane units into the polymer backbone (Figure 2). Similarly, poly(GA‐Sy‐*co*‐BDMSB‐*r*‐TMDS) was obtained using a binary mixture of hydrosilanes, yielding a copolymer with *M*_w_ = 28 600 g mol**^−1^** and *Đ* = 1.76 (Table 4, Entry 20).

**Table 4:** Thermal analyses of PSEs obtained in this study.^a)^

Entry	Polymer	*M*_n_ ^b)^ (g mol^−1^)	*M*_w_ ^b)^ (g mol^−1^)	*Đ* ^b)^	*T*_‐5%_ ^c)^ (°C)	*T*_‐50%_ ^c)^ (°C)	*T*_g_ ^c)^ (°C)
1	Poly(GA‐Va‐*co*‐Ph)	11 000	20 000	1.80	274	410	35
2	Poly(GA‐Va‐*co*‐BDMSB)	20 000	36 000	1.73	292	425	17
3	Poly(GA‐Va‐*co*‐TMDS)	14 200	22 200	1.58	225	363	2
4^d)^	Poly(GA‐Sy‐*co*‐Ph)	18 500	52 000	2.80	267	371	64
5	Poly(GA‐Sy‐*co*‐BDMSB)	12 900	22 100	1.70	282	383	54
6	Poly(GA‐Sy‐*co*‐TMDS)	20 600	36 030	1.74	263	369	16
7	Poly(AA‐Va‐*co*‐Ph)	17 000	41 900	2.46	298	405	26
8	Poly(AA‐Va‐*co*‐BDMSB)	23 000	38 000	1.64	306	414	4
9	Poly(AA‐Va‐*co*‐TMDS)	23 000	47 000	2.04	266	380	−13
10^d)^	Poly(AA‐Sy‐*co*‐Ph)	17 000	33 000	1.92	282	391	47
11	Poly(AA‐Sy‐*co*‐BDMSB)	11 900	22 000	1.85	300	387	44
12	Poly(AA‐Sy‐*co*‐TMDS)	8650	13 100	1.52	220	354	7
13	Poly(PA‐Va‐*co*‐Ph)	17 000	33 400	1.96	256	395	24
14	Poly(PA‐Va‐*co*‐BDMSB)	10 030	19 300	1.93	263	438	3
15	Poly(PA‐Va‐*co*‐TMDS)	12 400	26 000	2.09	240	391	−22
16^d)^	Poly(PA‐Sy‐*co*‐Ph)	9530	16 210	1.70	282	387	46
17	Poly(PA‐Sy‐*co*‐BDMSB)	17 700	33 200	1.86	277	391	40
18	Poly(PA‐Sy‐*co*‐TMDS)	20 000	35 000	1.75	229	354	3
19	Poly(GA‐Sy‐*co*‐BDMSB‐*r*‐Ph)	15 900	25 100	1.57	270	387	57
20	Poly(GA‐Sy‐*co*‐BDMSB‐*r*‐TMDS)	16 200	28 600	1.76	248	375	30

^a)^ All reactions were performed under argon, ester formation was carried out in THF at 50 °C, and polymerization was performed at room temperature.

^b)^ *M*_n_ *, M*_w_ *, and Đ* of polymer determined by SEC‐RI in THF calibrated with polystyrene standards at 35 °C.

^c)^ *T*_g_ of polymer determined by DSC on the second heating cycle (10 °C min**^−1^**, N_2_ flow). *T*_‐5%_ and *T*_‐50%_ of polymer determined by TGA (10 °C min**^−1^**, N_2_ flow).

^d)^ *T* = 70 °C.

### Thermal and Mechanical Properties of PSEs

**Figure 3:**
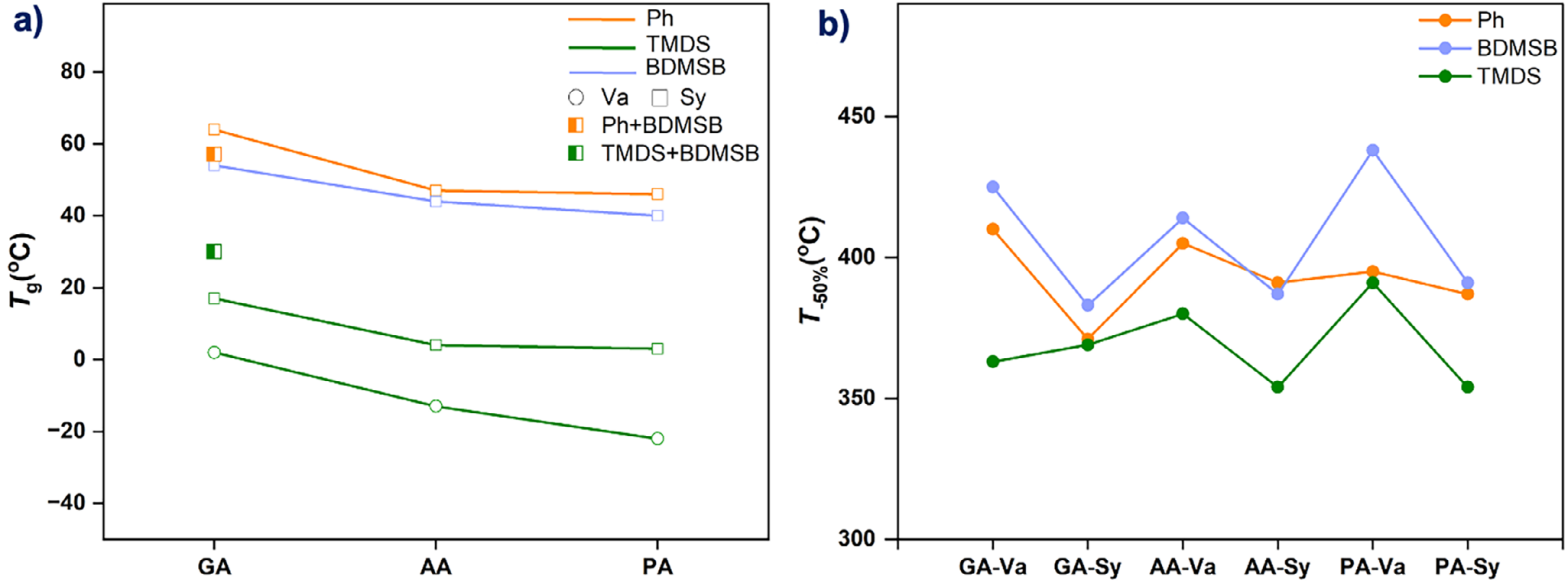
a) T_g_ and b) T_‐50%_ of PSEs in Table 4.

The thermal behavior of the synthesized PSEs was evaluated using differential scanning calorimetry (DSC) and thermogravimetric analysis (TGA), with results summarized in Table 4. All polymers exhibited glass transition temperatures (*T*_g_) ranging from −22 to 64 °C (Figure 3a), demonstrating the tunability of thermal properties through monomer selection. *T*_g_ values were found to be strongly dependent on the structure of the dihydrosilane precursors, with aromatic and rigid silane units yielding higher *T*_g_ values in the order: Ph > BDMSB > TMDS. This trend is likely attributable to the restricted rotational freedom of the Si–O–C linkages imposed by aryl‐enriched silane units, thereby enhancing chain rigidity and increasing *T*_g_.^[^ ^19^ ^]^

For example, copolymers derived from GA‐Va exhibited *T*_g_ values of 35 °C with Ph_2_SiH_2_ (Table 4, Entry 1), 17 °C with BDMSB (Table 4, Entry 2), and 2 °C with TMDS (Table 4, Entry 3). Statistical copolymerization enabled further fine‐tuning; poly(GA‐Sy‐*co*‐BDMSB‐*r*‐Ph) displayed a *T*_g_ at 57 °C (Table 4, Entry 19), which lies between the *T*_g_ values of poly(GA‐Sy‐*co*‐Ph) and poly(GA‐Sy‐*co*‐BDMSB) (Table 4, Entries 4, 5). Additionally, *T*_g_ decreased with increasing methylene units in the diacid backbone, reflecting enhanced flexibility of the alkyl segments. For Sy‐TMDS systems, extending the diacid chain length from glutaric acid to pimelic acid reduced *T*_g_ from 16 to 3 °C (Table 4, Entries 6, 12, 18). The choice of hydroxyaldehyde also impacted *T*_g_: syringaldehyde‐based copolymers with Ph_2_SiH_2_ exhibited a *T*_g_ of nearly 47 °C, compared to 26 °C for vanillin analogs (Table 4, Entries 7, 10). TGA analysis further highlighted the influence of comonomer structure on thermal stability. All PSEs exhibited excellent thermal resistance, with temperatures corresponding to 5% weight loss temperatures (*T*_‐5%_) ranging from 220 to 306 °C. The temperatures associated with 50% weight loss temperatures (*T*_‐50%_), which are less affected by early volatilization of residual water or solvent, were observed between 354 and 425 °C (Table 4).^[^ ^40^, ^41^ ^]^ Thermal stability followed the trend: BDMSB > Ph > TMDS (Figure 3b). These findings clearly demonstrate the importance of comonomer selection in tailoring thermal performance. This tunability in thermal behavior paves the way for the development of advanced PSEs with customizable properties, which is crucial for optimizing performance across diverse application domains.

**Figure 4:**
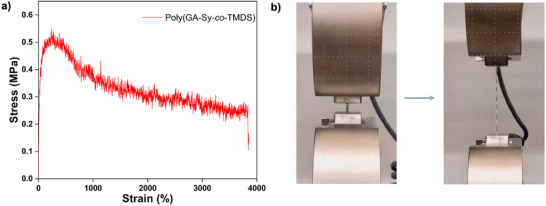
a) Stress–strain curve of poly(GA‐Sy‐*co*‐TMDS) and b) Tensile testing of zebra‐patterned poly(GA‐Sy‐*co*‐TMDS).

To evaluate the mechanical performance of the PSEs, preliminary uniaxial tensile tests were conducted to elucidate the deformation behavior of the resulting materials. Polymer samples were first dissolved in dichloromethane, cast onto Teflon‐coated molds, and subsequently processed into uniform, transparent films (Figure S138). Mechanical characterization was performed at ambient temperature using a universal testing machine (Instron 5968, USA) equipped with a 10 kN load cell, operating at a constant strain rate of 0.042 s^−1^. Both materials displayed an initial stress peak, followed by pronounced strain‐softening and an extended stress plateau (Figures 4a and S139), indicative of their distinctive deformation mechanisms.

Poly(GA‐Sy‐*co*‐TMDS) demonstrated remarkable extensibility, achieving an elongation at break of 3830% and an energy absorption capacity of 12.79 MJ m^−^^3^, approximately 24 times greater than that of poly(AA‐Va‐*co*‐Ph). Despite a reduction in Young's modulus, poly(GA‐Sy‐*co*‐TMDS) displayed superior tensile strength (0.52 MPa versus 0.36 MPa) (Table S2). During tensile loading, the zebra‐patterned poly(GA‐Sy‐*co*‐TMDS) deformed uniformly across the gauge section, and no evidence of strain localization, confirming homogeneous in‐plane stretching of the entire film (Figure 4b).

The extraordinary stretchability of poly(GA‐Sy‐*co*‐TMDS) can be rationalized by its molecular architecture, which combines a highly flexible siloxane backbone with dynamic silyl ether linkages. This structural motif enables efficient stress redistribution and mitigates strain localization during deformation. This synergistic interplay between backbone flexibility and dynamic connectivity provides an optimal balance of elasticity and toughness, distinguishing poly(GA‐Sy‐*co*‐TMDS) from conventional PDMS and TPU systems that rely on static crosslinks or hard segment reinforcement.^[^ ^42^ ^]^ These insights suggest broader design principles for ultra‐stretchable polymers: incorporating highly flexible backbones with dynamic, stress‐adaptive linkages can unlock unprecedented combinations of extensibility, toughness, and processability.

### Degradability of PSEs

PSEs are known to undergo degradation due to the presence of Si─O─C linkages in their backbone. A common method for evaluating PSE degradation involves dissolving the polymer in an organic solvent followed by treatment with a nucleophilic agent, such as methanol or aqueous HCl.^[^ ^16^, ^28^ ^]^ However, the incorporation of ester functionalities introduces additional hydrolytic sensitivity, necessitating efficient degradation strategies to enable timely polymer recycling and reuse. Methanesulfonic acid, a biodegradable Brønsted acid,^[^ ^43^ ^]^ has been reported as an efficient catalyst for the degradation of PSE‐*b*‐PLA systems, offering a sustainable pathway for polymer breakdown.^[^ ^44^ ^]^

**Figure 5:**
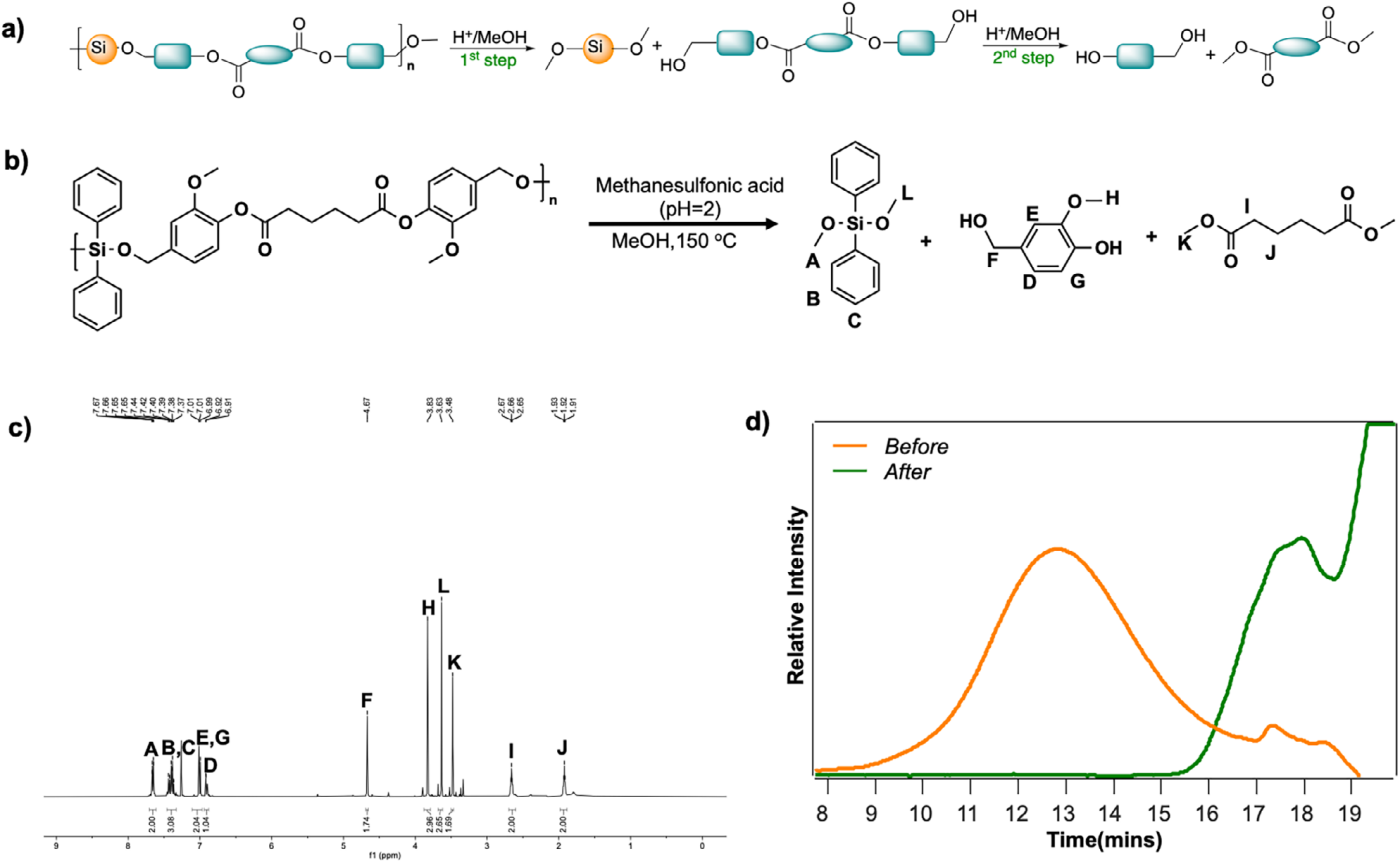
a) Representative stepwise degradation routes of poly(silyl ether)s b) Degradation study of poly(AA‐Va‐co‐Ph) showing the reaction conditions c) ^1^H NMR spectrum (400 MHz, CDCl_3_) of the crude mixture after the acid‐catalyzed methanolysis of poly(AA‐VA‐*co*‐Ph) d) SEC‐RI traces before and after the acid‐catalyzed methanolysis of poly(AA‐Va‐*co*‐Ph).

To evaluate the degradability of ester‐functionalized PSEs, poly(AA‐Va‐*co*‐Ph) was selected as a model system due to its backbone containing both silyl ether and ester linkages. Degradation was initiated via Brønsted acid‐catalyzed methanolysis using methanesulfonic acid. Under acidic alcoholysis conditions, the process proceeded through two sequential steps: protonation of the Si–O–C linkage followed by nucleophilic attack of methanol at the silicon center, leading to a rapid cleavage of Si─O─C bonds and yielding bis(alkoxysilyl) and bis(hydroxyalkyl)‐alkyldicarboxylate intermediates. Subsequently, transesterification of the ester groups yielded dialkyl esters and diols (Figure 5a).^[^ ^18^, ^19^, ^45^ ^]^ Remarkably, the polymer exhibited a rapid molecular weight reduction, with approximately 82% within 1 h and complete degradation within 6 h (Figure S133). Early degradation products included bis(hydroxyalkyl)‐alkyldicarboxylates and dimethoxydiphenylsilane, which further converted into dimethyl adipate and 4‐(hydroxymethyl)‐2‐methoxyphenol (Figure 5b). FT‐IR spectroscopy confirmed the degradation pathway, with a shift in the carbonyl stretching band from *ν* = 1760 cm^−1^ to *ν* = 1692 cm^−1^, indicating ester cleavage and formation of carboxylic acid groups (Figure S134). The appearance of a hydroxyl band at *ν* = 3440 cm^−1^ further supported the breakdown of the polymer backbone. Complementary ^1^H and ^13^C NMR analyses (Figure S135) confirmed the identity of the degradation products.

**Figure 6:**
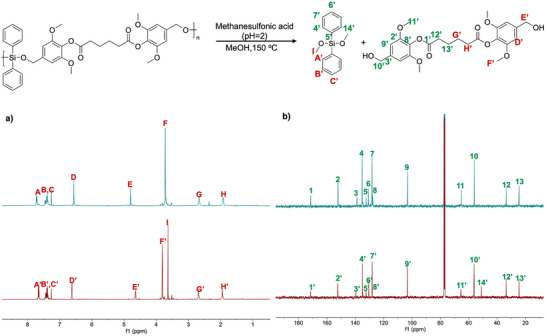
a) ^1^H NMR spectrum (400 MHz, CDCl_3_), b) ^13^C{^1^H} NMR spectrum (101 MHz, CDCl_3_) of the crude mixture resulting from methanolysis of poly(AA‐Sy‐*co*‐Ph) after 6 h.

Interestingly, while poly(AA‐Va‐*co*‐Ph) underwent complete degradation, poly(AA‐Sy‐*co*‐Ph) followed a distinct pathway. During its breakdown, the characteristic PSE signal at *δ* = 3.72 ppm in the ^1^H NMR spectrum disappeared, while two new methoxy resonances emerged at *δ* = 3.83 and 3.63 ppm (Figure 6a). Correspondingly, the ^13^C NMR spectrum revealed two methoxy carbon signals at *δ* = 65.55 and 51.09 ppm (Figure 6b), indicating preferential cleavage of the Si─O─C bond, while the ester group remained largely intact. Even after prolonged reaction times, ester hydrolysis was incomplete, likely due to steric hindrance imposed by the syringyl substituent (Figure S136).^[^ ^18^, ^19^ ^]^ Poly(AA‐Va‐*co*‐Ph) also underwent hydrolysis under basic conditions at room temperature (Figure S137).

Methanolysis of poly(silyl ether)s yields diols and dialkoxysilanes, which are key intermediates for chemical recycling. Recent studies show closed‐loop repolymerization via silicon acetal metathesis and siloxane polycondensation, producing polymers with properties comparable to virgin materials.^[^ ^46^, ^47^, ^48^ ^]^ In addition, diols enable the synthesis of renewable polyurethanes,^[^ ^49^ ^]^ thermosets,^[^ ^50^ ^]^ and polysiloxanes.^[^ ^51^ ^]^ These findings underscore the importance of monomer design and molecular architecture in governing degradation pathways and kinetics, and highlight the potential of poly(silyl ether)s as sustainable polymers with programmed degradability.

## Conclusion

A multicatalytic platform that integrates magnesium‐catalyzed esterification with borane‐mediated hydrosilylation was successfully employed to synthesize ester‐functionalized poly(silyl ether)s under mild conditions. The proposed one‐pot strategy significantly broadens the structural diversity of bio‐based PSEs, enabling precise modulation of thermal behavior and chemical stability. Importantly, preliminary mechanical characterization revealed exceptional extensibility and energy absorption in selected copolymers, underscoring the potential of this architecture for flexible and high‐performance applications. Furthermore, the incorporation of degradable linkages facilitates efficient chemical recycling via acid‐ and base‐catalyzed depolymerization, enabling recovery of monomeric building blocks and reinforcing circular economy principles. These findings highlight the promise of multicatalytic design in advancing sustainable polymer technologies that combine durability, tunability, and programmed end‐of‐life recyclability.

## Conflict of Interests

The authors declare no conflict of interest.

## Supporting information



Supporting Information

## Data Availability

The data that support the findings of this study are available in the Supporting Information of this article.
