# Supplementary material for: Multicatalytic Access to Renewable Poly(Silyl Ether)s with Tunable Properties
Source: Angew Chem Int Ed Engl. 2026 Jan 14;65(8):e21137. doi: 10.1002/anie.202521137 (PMC12910142; doi:10.1002/anie.202521137)
Supplement: Supplementary file 1 — Supporting Information [file ANIE-65-e21137-s001.pdf]

Supporting information

Multicatalytic Access to Renewable Poly(silyl ether)s with Tunable Properties

Fan Yang, Fan Sun, Christophe M. Thomas*

Table of Contents

Experimental section 3

General procedure for the Ester-formation procedure 5

General procedure for the one-pot synthesis of bio-based polysilyethers(PSEs) 5

General procedure for the degradation of partially bio-based poly(silyl ether)s 6

Structural characterization of bio-based ester monomers 7

Structural characterization of bio-based poly(silyl ether)s 19

Fourier transform infrared spectroscopy (FTIR) 79

SEC-RI traces of the synthesis of bio-based PSEs 89

Thermal performance of bio-based PSEs 99

Degradation of PSEs 107

Mechanical properties of PSEs 111

Experimental section

Materials and methods

All manipulations requiring a dry atmosphere were performed under a purified argon atmosphere using standard Schlenk techniques or in a glovebox. Solvents for synthesis (toluene, THF, DCM, *n*-pentane) were freshly distilled from Na/benzophenone or calcium hydride under argon and degassed thoroughly by freeze-thaw-vacuum cycles prior to use.

Di-*tert*-butyl dicarbonate (Boc_2O, Fluorochem), vanillin (99%, Thermo Scientific), syringaldehyde (98%, TCI), glutaric acid (99%, Sigma-Aldrich), adipic acid (99%, Sigma-Aldrich), pimelic acid (98%, Alfa Aesar), magnesium dichloride ($MgCl_2$, 99%, Alfa Aesar), isopropylmagnesium chloride lithium chloride complex solution (*i*PrMgCl·LiCl, 1.3 M in THF, Sigma-Aldrich tris(pentafluorophenyl)borane ($B(C_6F_5)_3$, 99%, TCI), sodium (Na, Sigma-Aldrich), chloroform-*d* ($CDCl_3$, 99.5% D, Eurisotop), hydrochloric acid (HCl, 37%, VWR), chloroform-*d* ($CDCl_3$, 99.5% D, Eurisotop), acetone-d_6 (99.9% D, Eurisotop).

Diphenylsilane (Ph, 99.04%, BLD Pharmatech), 1,1,3,3-tetramethyldisiloxane (TMDS, 97%, TCI) and 1,4-bis(dimethylsilyl)benzene (BDMSB, 98.85%, BLD Pharmatech) were freshly distilled, degassed and stored under argon prior to use. Anisole (99%, Alfa Aesar) were dried over sodium, and then freshly distilled prior to use. Toluene, dichloromethane (DCM, CH_2Cl_2), tetrahydrofuran (THF), and pentane were dried over sodium and distilled under argon prior to use or taken under argon from a solvent purification system (SPS).

Nuclear Magnetic Resonance (NMR) Spectroscopy: NMR spectra were recorded on Bruker Avance-400 and Avance-Neo 500 spectrometers at Chimie ParisTech. ^{1}H, ^{13}C and ^{29}Si chemical shifts are reported in ppm versus $SiMe_4$ and were determined by reference to the residual solvent peaks for ^{1}H and ^{13}C NMR and to the chemical shift of TMS (0 ppm) used as external reference

for ^{29}Si NMR. Assignment of signals was performed from multinuclear 1D (^{1}H, ^{13}C) and 2D (COSY, HMQC, HMBC, DOSY) NMR experiments.

Diffuse reflectance Fourier transform infrared: measurements were carried out on a Thermo Fisher Nicolet IS 20 FT-IR spectrometers equipped with a Harrick Praying Mantis device.

Gel permeation chromatography (GPC): Size exclusion chromatography (SEC) of polymers was performed in THF at 35 °C using an Agilent 1260 Infinity Series GPC (ResiPore 3 µm, 300 x 7.5 mm, 1.0 mL/min, RI (PL-GPC 220) and Light scattering detectors) at Chimie ParisTech. When using the RI detector, the number of average molecular masses (M_n) and polydispersity index ($Đ$) of the polymers were calculated with reference to a universal calibration vs. polystyrene standards (limits M_w = 200 to 400,000 g/mol).

Differential scanning calorimetry (DSC): The glass transition temperature (T_g) of polymer was determined by differential scanning calorimeter (DSC25, TA Instrument, USA) a nitrogen flow of 25 mL/min. Samples (1-5 mg) were sealed in aluminum Tzero hermetic pans and lids using a Tzero sample press (TA Instruments, USA).[1] To erase thermal history of polymer, a DSC program with heat/cool/heat cycle was used: the first heating was done from 40 ºC to 220 ºC at 10 ºC/min; then the cooling was done from 220 ºC to -80 ºC at -10 ºC/min; the final heating was done from -80 ºC to 220 ºC at 10 ºC/min.

Thermogravimetric analysis (TGA): The thermal stability was evaluated by thermogravimetric analyzer (TGA55, TA Instruments, USA). Samples of 1-5 mg were heated from 40 ºC to 400 (or 550) ºC at a heating rate of 10 ºC/min under nitrogen flow rate of 25 mL/min. $T_{d,-5\%}$ and $T_{d,-50\%}$ of polymers was defined as the thermal degradation temperature corresponding to 5% and 50% weight lost, respectively.

---

1 https://www.tainstruments.com/pdf/literature/TN071_REV-B-DSC_Sample_Pan_Guide.pdf (accessed September 2025).

Tensile Testing: The polymers were dissolved in DCM and cast onto a teflon-coated mold. After allowing the solvent to evaporate naturally for 12 h at room temperature. The polymer films were carefully peeled from the dog bone shape mold. Uniaxial tensile tests were then conducted at room temperature using a universal testing machine (Instron 5968, USA) equipped with a 10 kN load cell, operating at a constant strain rate of 0.042 s^{-1}.

General procedure for the Ester-formation procedure

A solution of the acid, the bio-based alcohol, Boc_2O (coupling reagent), and [Mg] (4.0 mol% catalyst) in freshly distilled THF was stirred in a Schlenk tube and heated at 50 °C for the indicated time under argon. Unless otherwise noted: [Alcohol] = 2.0 × [Acid] and [Boc_2O] = 2.2 × [Acid] in THF. After the reaction, the solvent was removed under reduced pressure. The residue was filtered and washed with hot *n*-pentane to afford the monomer, which was analyzed by NMR spectroscopy.

General procedure for the one-pot synthesis of bio-based polysilyethers(PSEs)

The general procedure for the one-pot synthesis of partially bio-based poly(silyl ether)s involves combining an ester formation with a subsequent polymerization step. To begin, within the ester-formation step, a solution of acid, alcohol, Boc_2O as coupling reagent, [Mg] as catalyst in dry THF was stirred in a Schlenk tube and heated at 50 ºC for a determined reaction time. All reactions were performed under argon, with [-OH] : [Boc_2O] : [-COOH] = 2 : 2.4 : 2, and a catalyst loading of 4 mol%. After complete conversion of the alcohol determined by ^{1}H NMR spectroscopy, the solution mixture was evaporated to remove *t*-BuOH. Then, dihydrosilane (1.0 eq. to diacid), 0.5-1 mol% $B(C_6F_5)_3$ as catalyst and toluene were added to this Schlenk tube, stirred at set temperature for 24 h. After the polymerization, the solvent was evaporated, and the crude product was dissolved in minimum amount of DCM then precipitate with an excess of cold *n*-

pentane. Finally, the product was dried under vacuum. The resulting poly(silyl ether)s were consistently stored in Schlenk tubes under argon.

As a representative example for the synthesis of poly(AA-Va-*co*-Ph):

A solution of adipic acid (141 mg, 1.0 mmol), vanillin (304 mg, 2.0 mmol), Boc_2O (523.2 mg, 2.4 mmol) and *i*PrMgCl·LiCl (3.8 mg, 40 μmol) as a catalyst in dry THF (1 mL) were stirred in a 25 mL Schlenk tube and heated at 50 °C for 5 h. After complete conversion of the -CHO determined by ^1H NMR spectroscopy, the solution mixture was evaporated to remove the *t*-BuOH. Then, diphenyl silane (184.3 mg, 1.0 mmol), $B(C_6F_5)_3$ (5 mg, 10 μmol) and toluene (0.5 mL) were added to the reaction mixture. The solution was stirred at set temperature for 24 h. After the polymerization, the toluene was evaporated, and the crude product was dissolved in minimum amount of DCM then precipitate with an excess of cold pentane (10 mL). Finally, the product of poly(AA-Va-*co*-Ph) was dried under vacuum.

General procedure for the degradation of partially bio-based poly(silyl ether)s

Under atmospheric pressure, 20 mg of the target polymer was introduced into a dry Schlenk tube and dissolved in 2.0 mL of methanol. Methanesulfonic acid was then added, and the reaction mixture was stirred at 150 °C. For intermediate analyses, the Schlenk tube was cooled to room temperature, carefully vented by gradual opening of the PTFE valve, and small aliquots were regularly taken, dried under vacuum, and characterized by SEC using refractive index detection. The tube was subsequently resealed and reheated to 150 °C to resume the reaction. Refractive index detection was selected over light scattering due to the latter's requirement for precisely known polymer concentrations and purification for accurate measurements. Results are reported as relative reductions in weight-average molecular weight (M_w), mitigating inaccuracies associated

with RI calibration against polystyrene standards.

Structural characterization of bio-based ester monomers

GA-Va

^{1}H NMR (400 MHz, Chloroform-*d*) δ 9.96 (s, 2H), 7.52 – 7.47 (m, 4H), 7.23 (d, *J* = 7.8 Hz, 2H), 3.91 (s, 6H), 2.81 (t, *J* = 7.2 Hz, 4H), 2.24 (t, *J* = 7.2 Hz, 2H). **^{13}C{^{1}H} NMR (101 MHz, Chloroform-*d*)** δ 191.13, 170.46, 152.02, 145.00, 135.41, 124.91, 123.49, 110.92, 56.18, 32.85, 20.28.

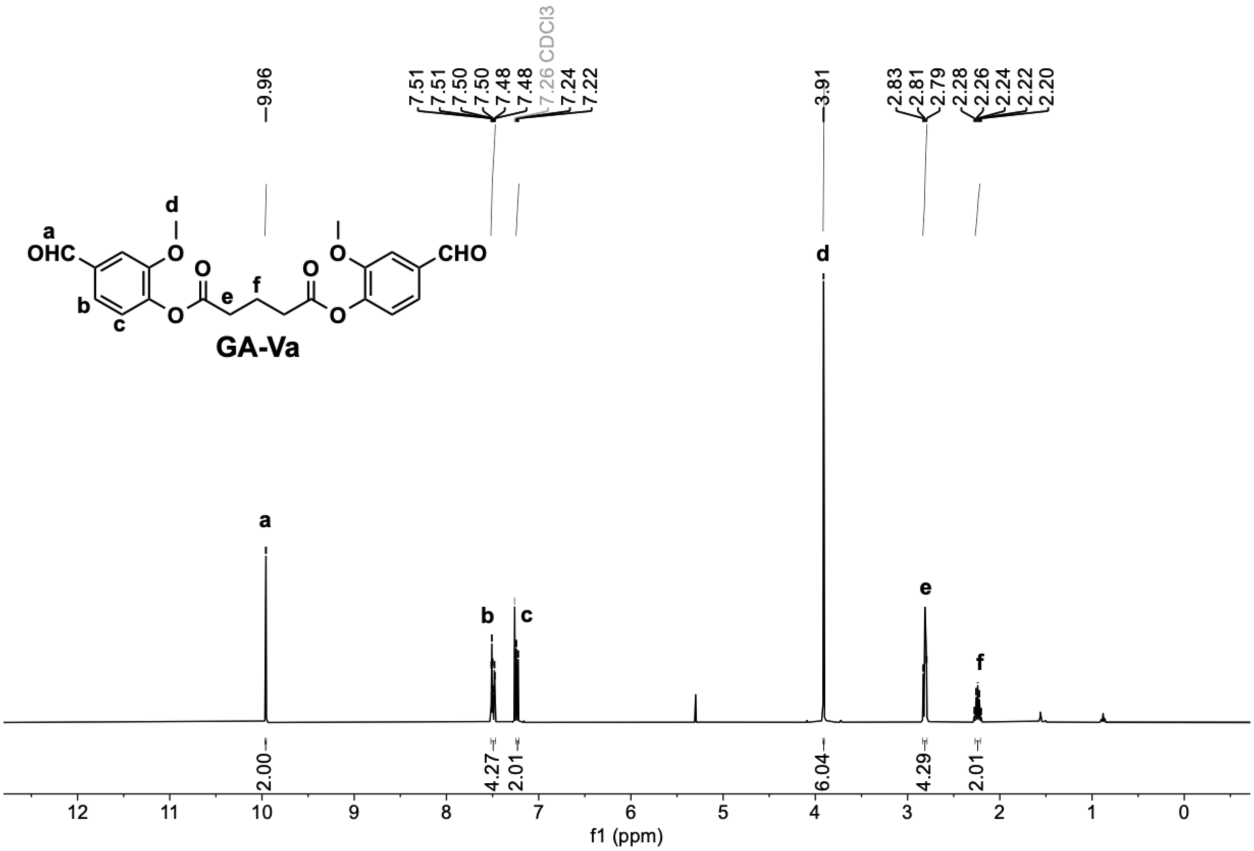

Figure S1 ^{1}H NMR spectrum (400 MHz, $CDCl_3$) of **GA-Va**.

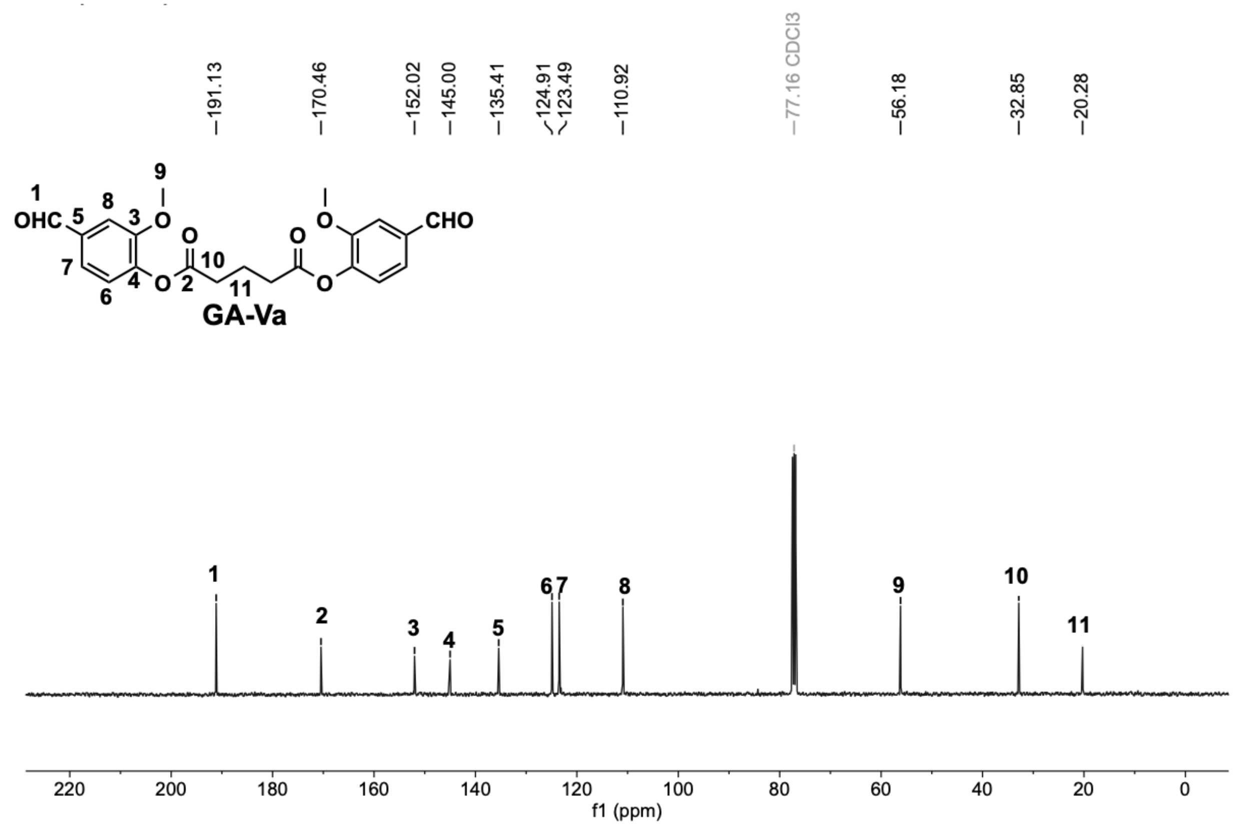

Figure S2 $^{13}C\{^{1}H\}$ NMR spectrum (101 MHz, $CDCl_3$) of **GA-Va.**

GA-Sy

^{1}H NMR (400 MHz, Chloroform-*d*) δ 9.91 (s, 2H), 7.16 (s, 4H), 3.91 (s, 12H), 2.84 (t, *J* = 7.3 Hz, 4H), 2.26 (p, *J* = 7.3 Hz, 2H). **^{13}C{^{1}H} NMR (101 MHz, Chloroform-*d*)** δ 191.15, 170.26, 153.00, 134.47, 133.90, 106.19, 56.44, 32.77, 20.61.

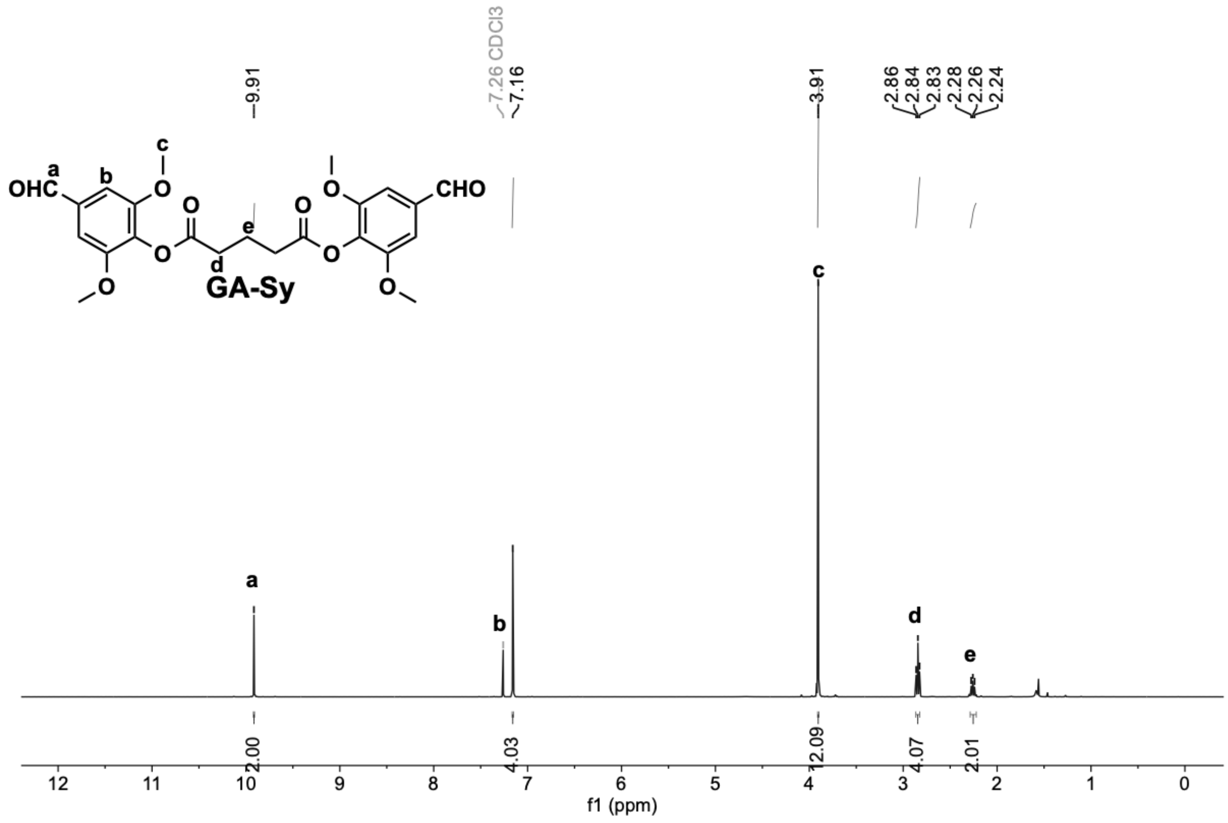

Figure S3 ^{1}H NMR spectrum (400 MHz, $CDCl_3$) of **GA-Sy**.

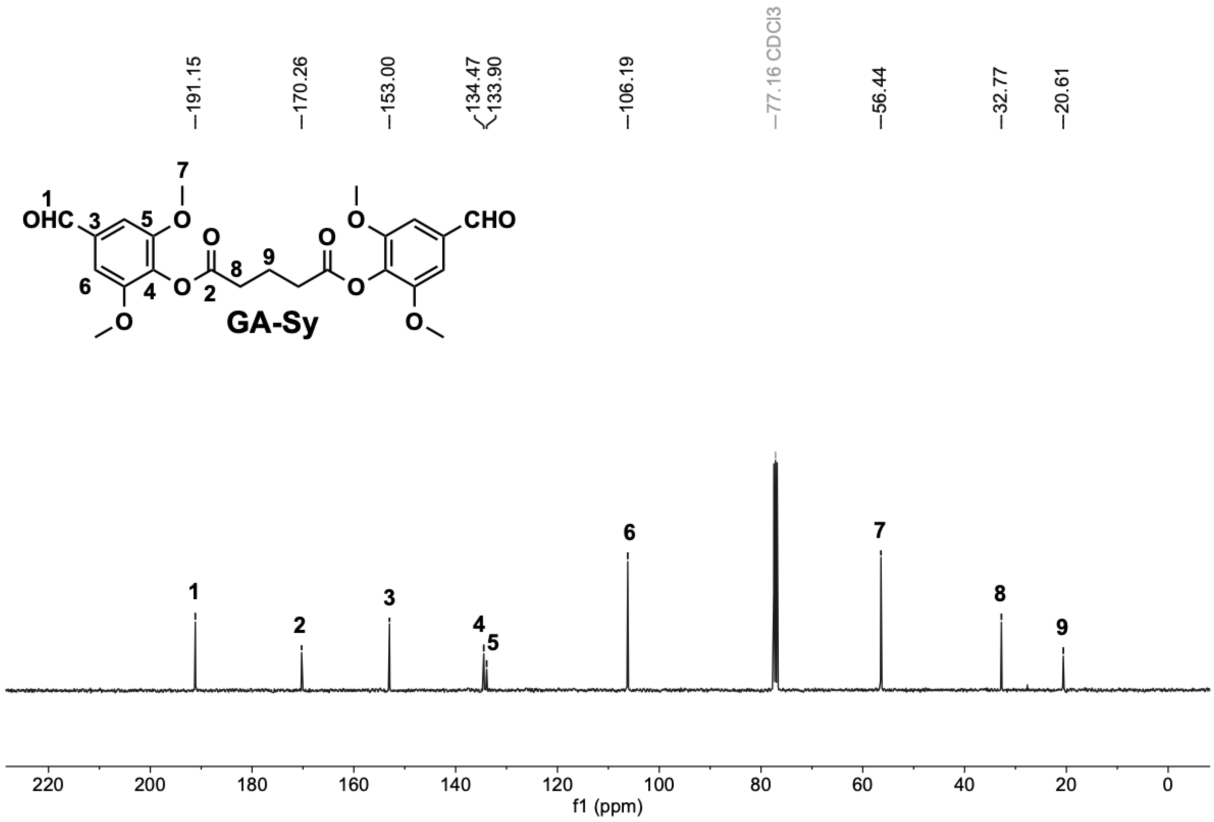

Figure S4 $^{13}C\{^{1}H\}$ NMR spectrum (101 MHz, $CDCl_3$) of **GA-Sy.**

AA-Va

^{1}H NMR (400 MHz, Chloroform-*d*) δ 9.95 (s, 2H), 7.51 – 7.46 (m, 4H), 7.22 (d, *J* = 7.9 Hz, 2H), 3.89 (s, 6H), 2.69 (s, 4H), 1.93 (s, 4H). **^{13}C{^{1}H} NMR (101 MHz, Chloroform-*d*)** δ 191.15, 170.81, 152.08, 145.08, 135.37, 124.90, 123.52, 110.93, 56.18, 33.69, 24.31.

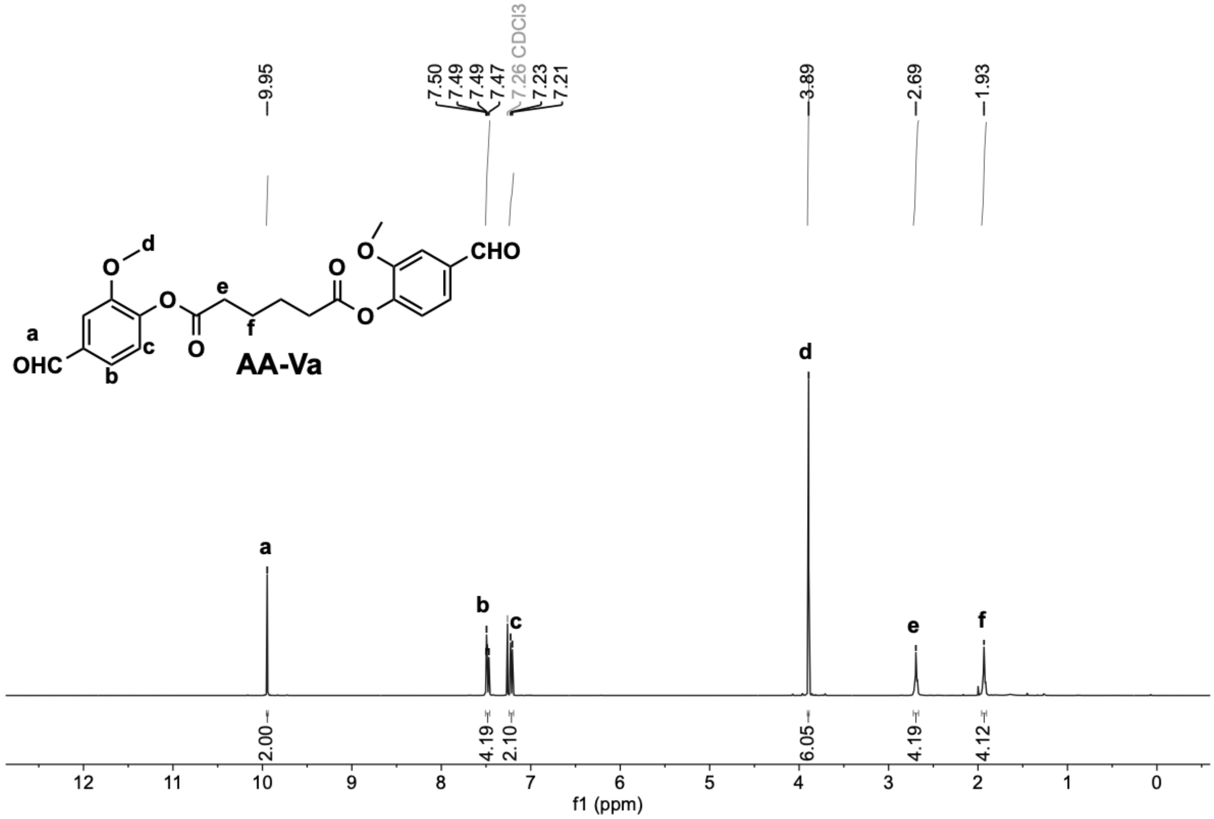

Figure S5 ^{1}H NMR spectrum (400 MHz, $CDCl_3$) of **AA-Va**.

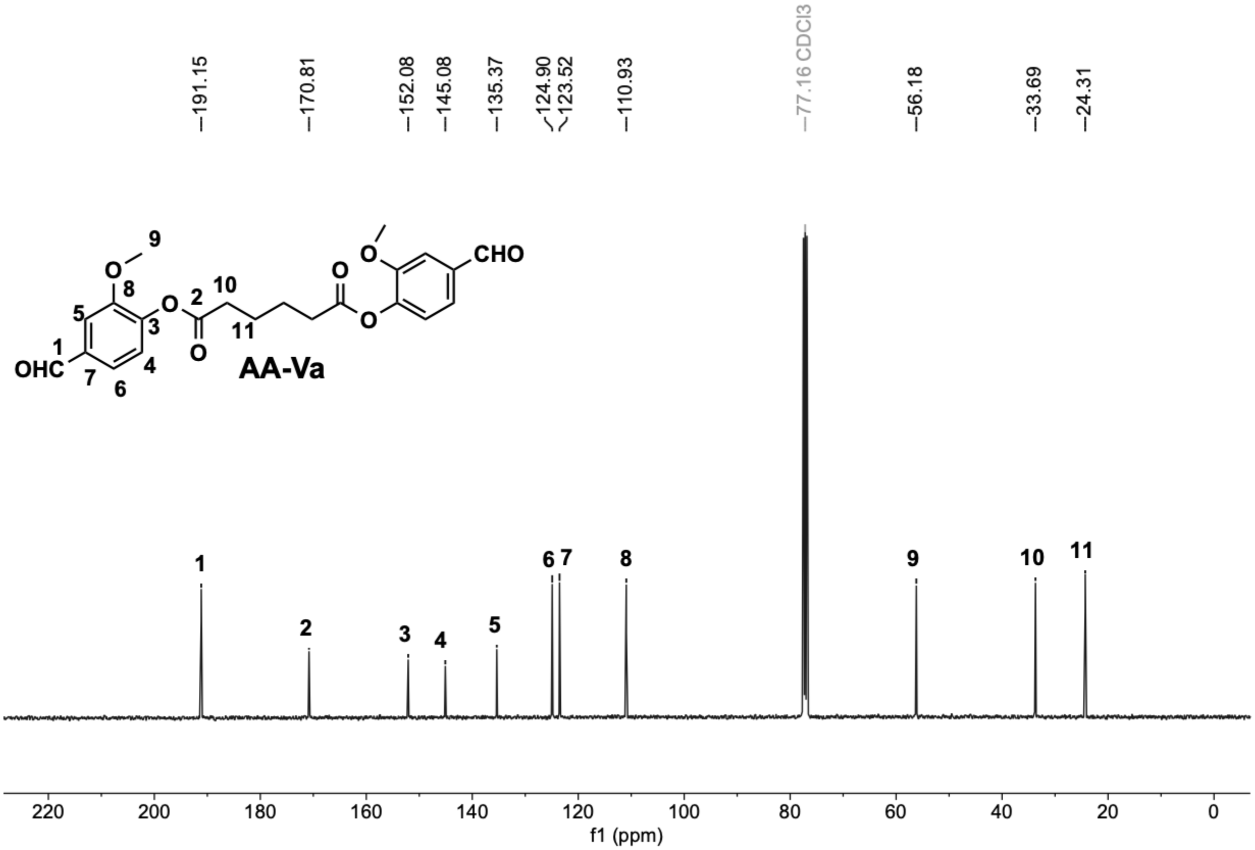

Figure S6 $^{13}C\{^{1}H\}$ NMR spectrum (101 MHz, $CDCl_3$) of **AA-Va.**

AA-Sy

^{1}H NMR (400 MHz, Chloroform-*d*) δ 9.91 (s, 2H), 7.15 (s, 4H), 3.89 (s, 13H), 2.72 (q, *J* = 4.4, 5.9 Hz, 4H), 1.96 (p, *J* = 3.3 Hz, 4H). **^{13}C{^{1}H} NMR (101 MHz, Chloroform-*d*)** δ 191.17, 170.60, 153.04, 134.45, 133.97, 106.20, 56.45, 33.58, 24.41.

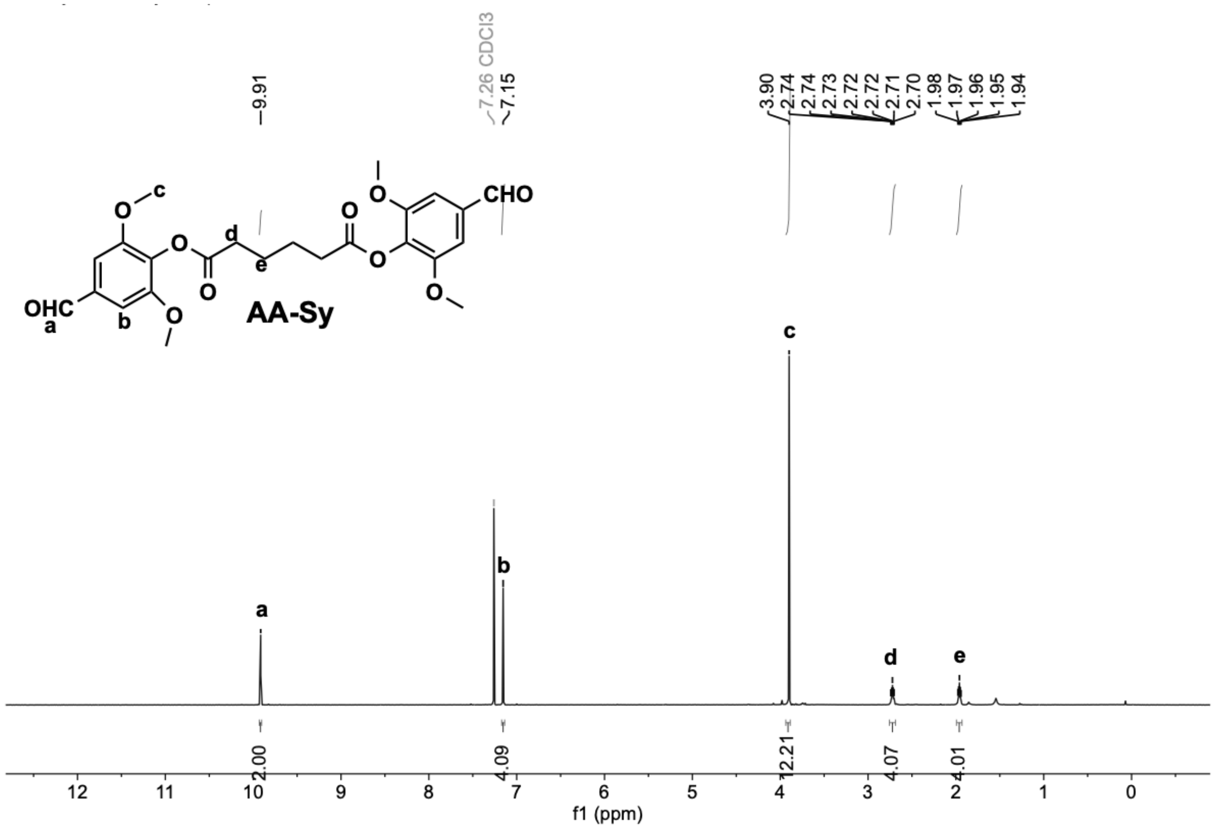

Figure S7 ^{1}H NMR spectrum (400 MHz, $CDCl_3$) of **AA-Sy**.

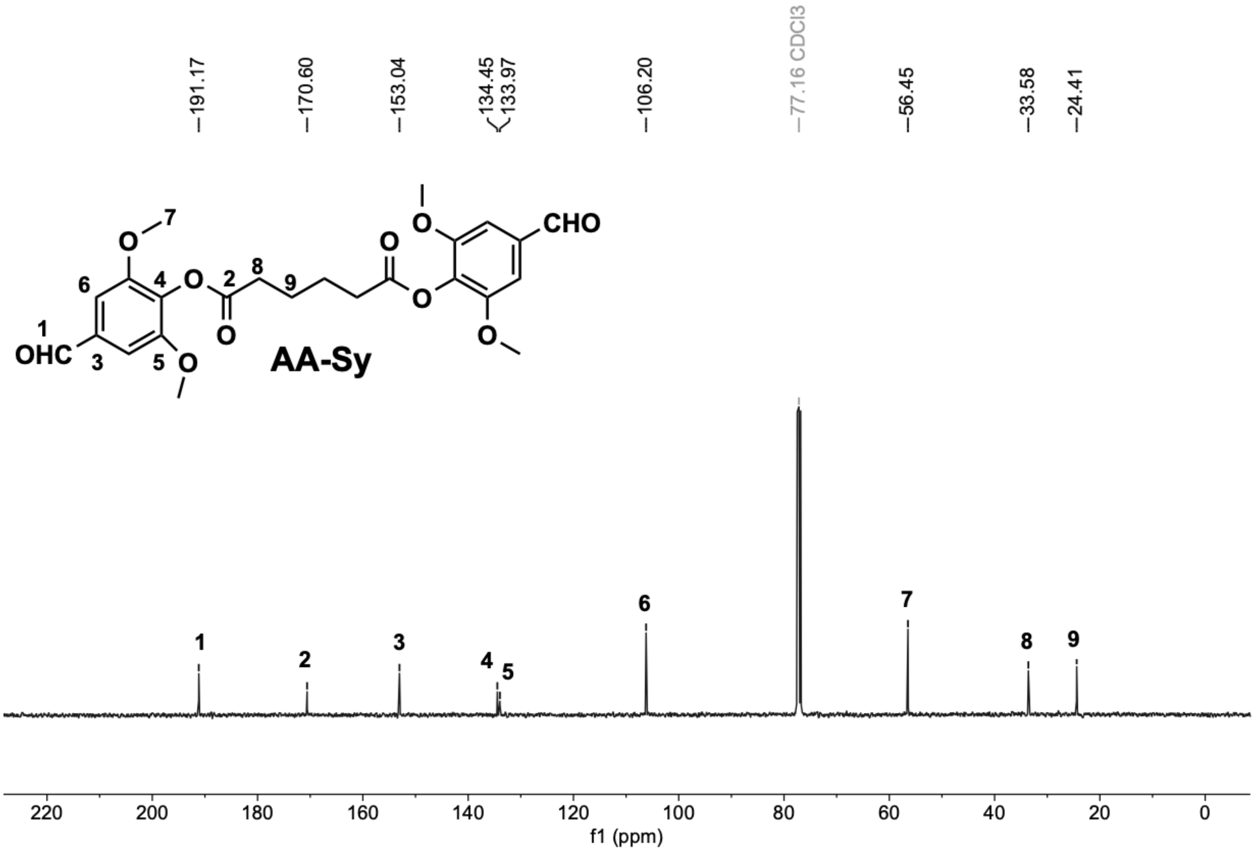

Figure S8 $^{13}C\{^{1}H\}$ NMR spectrum (101 MHz, $CDCl_3$) of **AA-Sy.**

PA-Va

^{1}H NMR (400 MHz, Chloroform-*d*) δ 9.95 (s, 2H), 7.51 – 7.45 (m, 4H), 7.21 (d, *J* = 7.9 Hz, 2H), 3.89 (s, 6H), 2.66 (t, *J* = 7.4 Hz, 4H), 1.92 – 1.80 (m, 4H), 1.61 (d, *J* = 7.1 Hz, 2H). **^{13}C{^{1}H} NMR (101 MHz, Chloroform-*d*)** δ 191.14, 171.02, 152.08, 145.10, 135.31, 124.86, 123.52, 110.91, 56.18, 33.85, 28.42, 24.61.

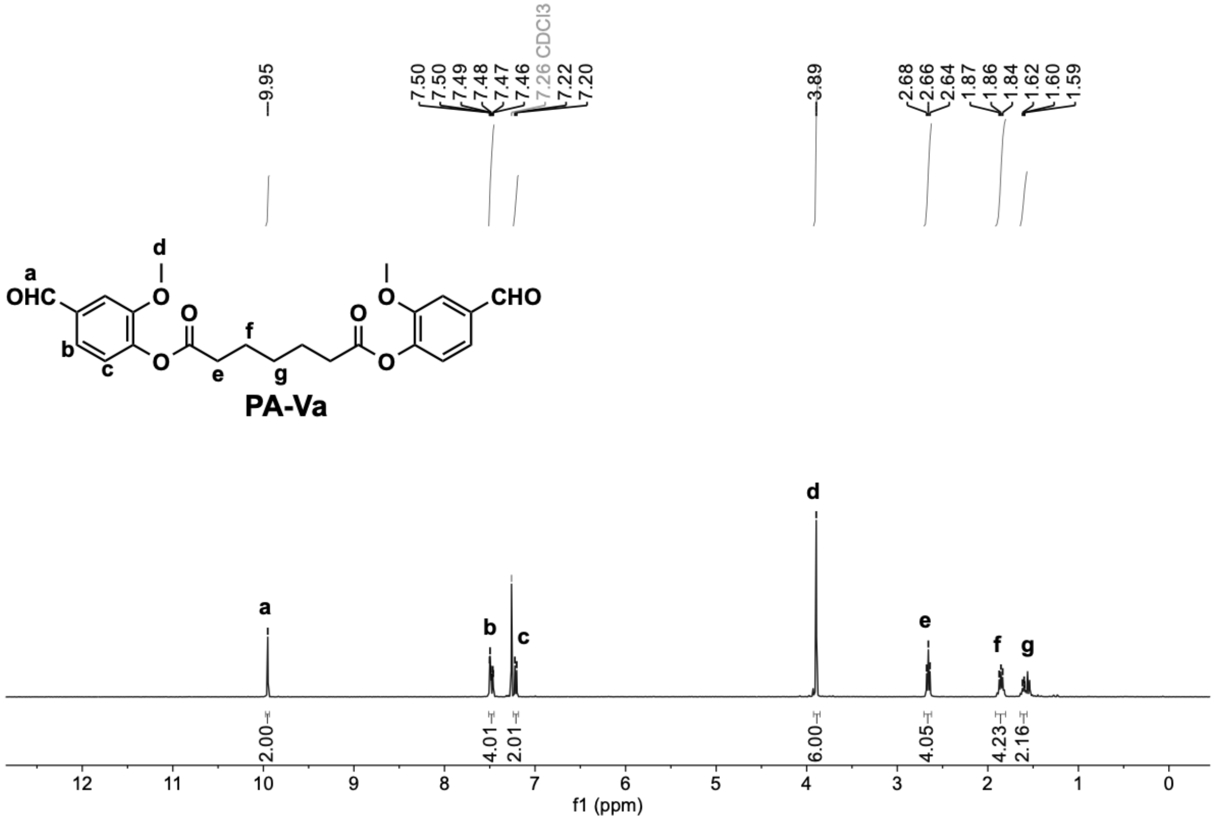

Figure S9 ^{1}H NMR spectrum (400 MHz, $CDCl_3$) of **PA-Va**.

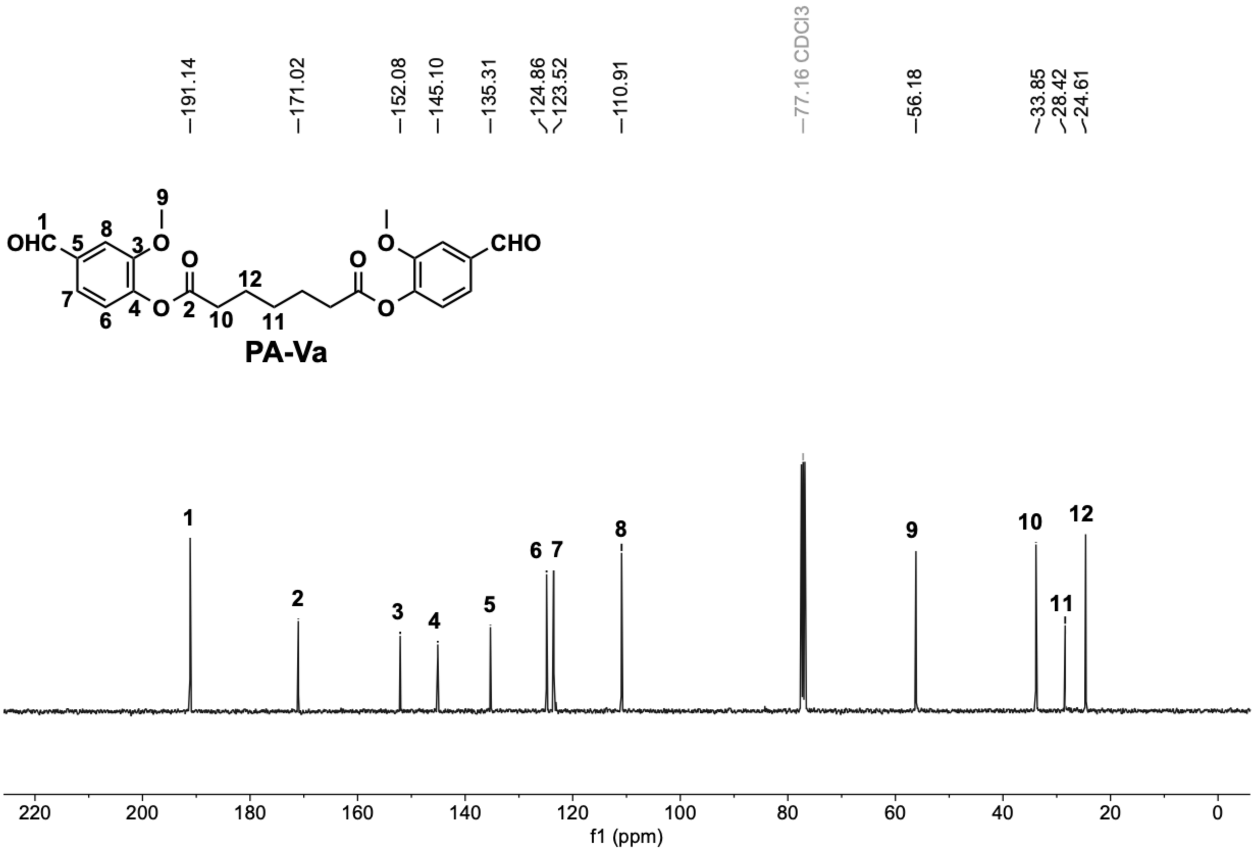

Figure S10 $^{13}C\{^1H\}$ NMR spectrum (101 MHz, $CDCl_3$) of **PA-Va.**

PA-Sy

^{1}H NMR (400 MHz, Chloroform-*d*) δ 9.91 (s, 2H), 7.15 (s, 4H), 3.89 (s, 12H), 2.68 (t, J = 7.3 Hz, 4H), 1.89 – 1.83 (m, 4H), 1.66 – 1.60 (m, 2H). **^{13}C{^{1}H} NMR (101 MHz, Chloroform-*d*)** δ 191.17, 170.78, 153.03, 134.41, 133.99, 106.19, 56.46, 33.78, 28.40, 24.76.

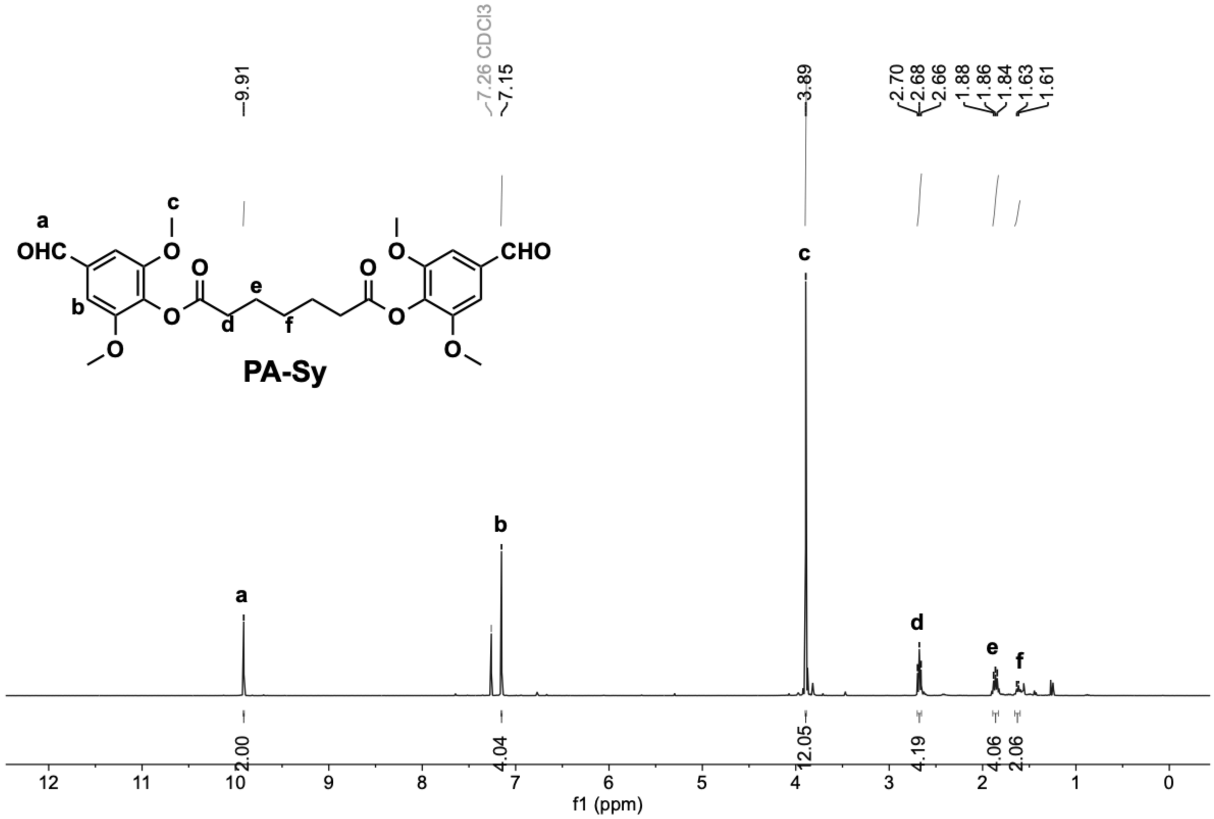

Figure S11 ^{1}H NMR spectrum (400 MHz, $CDCl_3$) of **PA-Sy**.

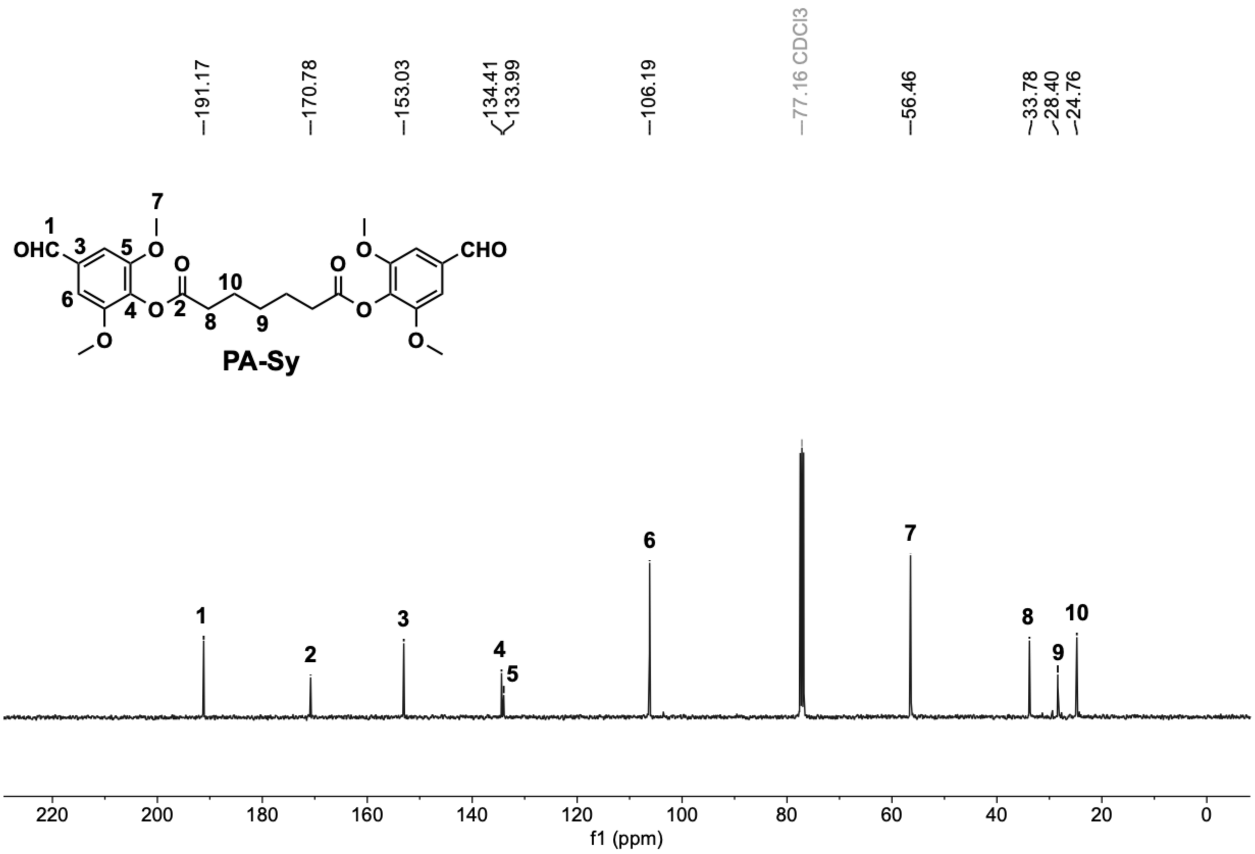

Figure S12 $^{13}C\{^{1}H\}$ NMR spectrum (101 MHz, $CDCl_3$) of **PA-Sy**.

Structural characterization of bio-based poly(silyl ether)s

Poly(GA-Va-co-Ph)

^{1}H NMR (400 MHz, Chloroform-*d*) δ 7.73 – 7.69 (m, 5H), 7.38 (dd, *J* = 6.4, 7.9 Hz, 6H), 6.97 (d, *J* = 8.0 Hz, 2H), 6.92 – 6.88 (m, 4H), 4.82 (s, 4H), 3.74 (s, 6H), 2.75 (t, *J* = 7.2 Hz, 4H), 2.20 (t, *J* = 7.2 Hz, 2H). **^{13}C{^{1}H} NMR (101 MHz, Chloroform-*d*)** δ 171.28, 151.04, 139.36, 138.88, 135.09, 132.24, 130.76, 128.18, 122.61, 118.75, 110.82, 64.87, 55.86, 33.01, 20.54. **^{29}Si NMR (99 MHz, Chloroform-*d*)** δ -30.25.

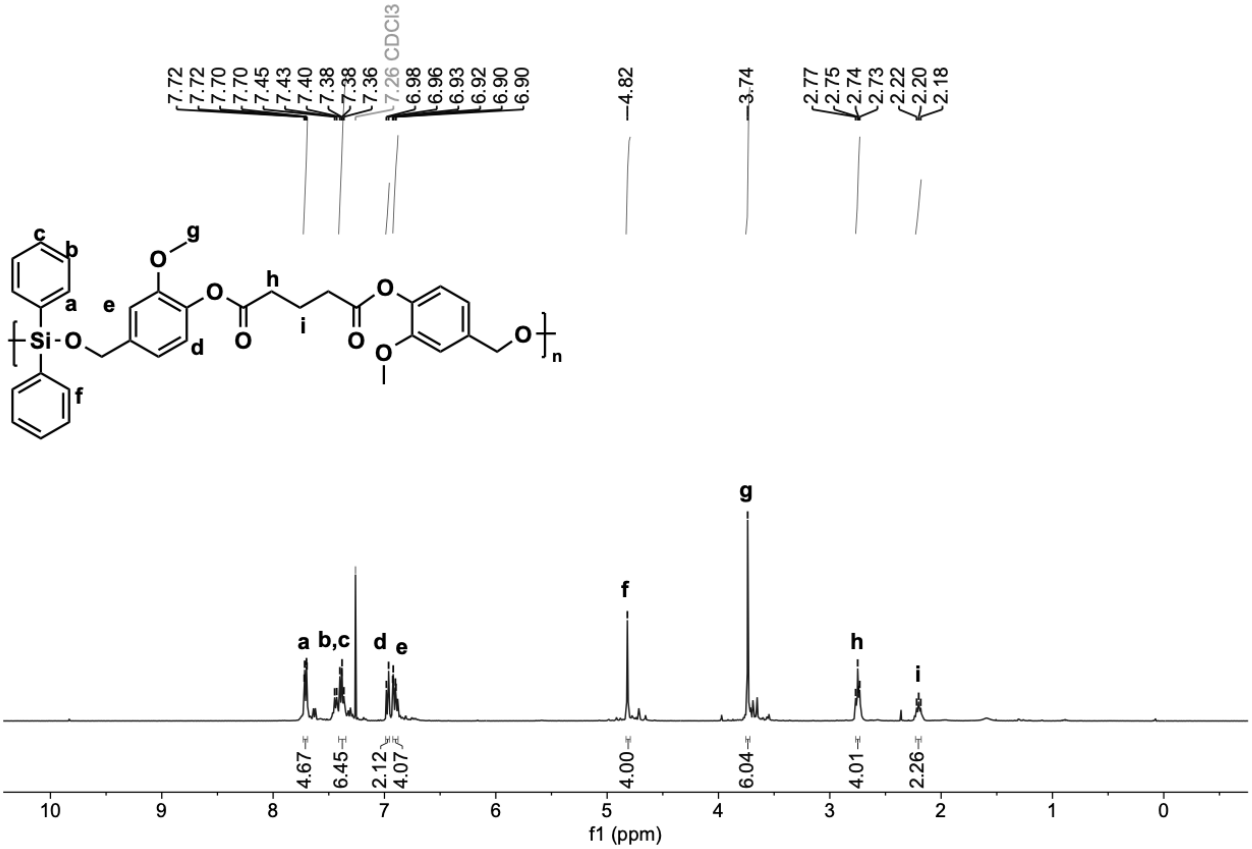

Figure S13 ^{1}H NMR spectrum (400 MHz, $CDCl_3$) of **poly(GA-Va-*co*-Ph)**.

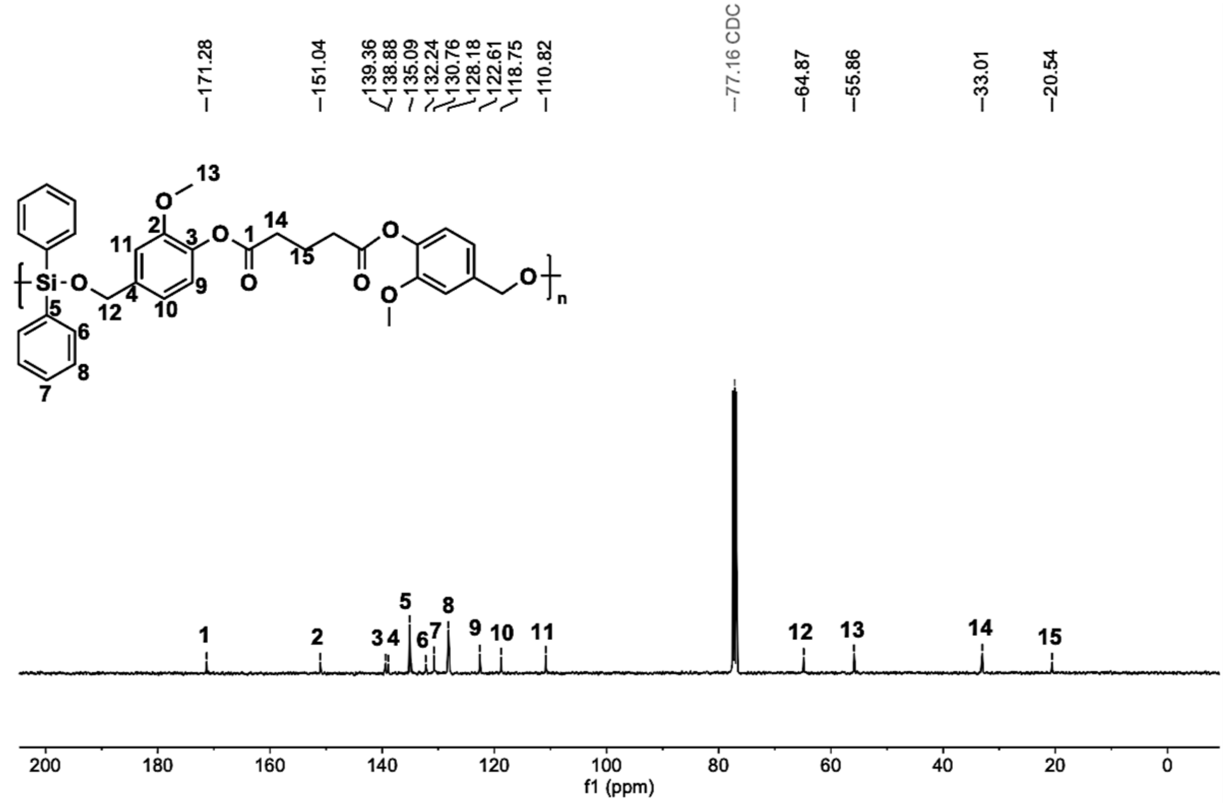

Figure S14 $^{13}C\{^{1}H\}$ NMR spectrum (101 MHz, $CDCl_3$) of **poly(GA-Va-*co*-Ph)**.

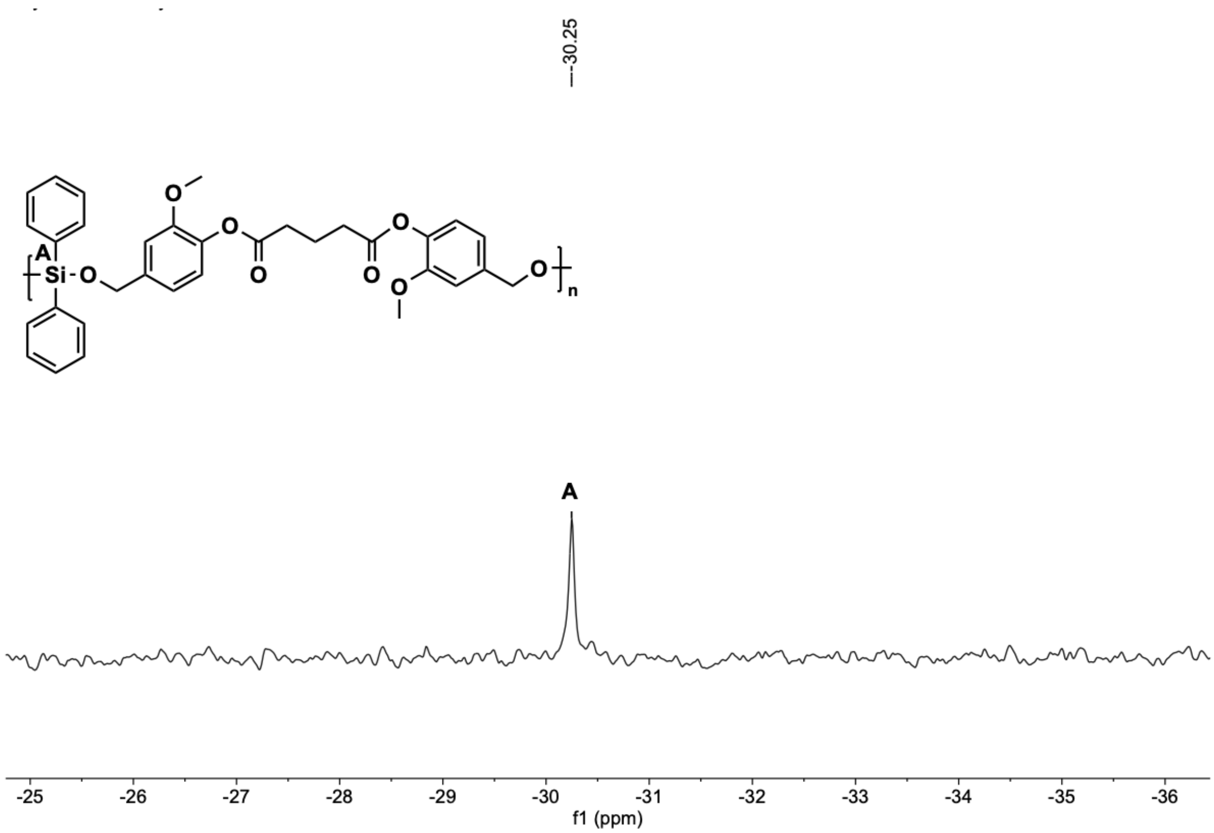

Figure S15 ^{29}Si NMR spectrum (99 MHz, $CDCl_3$) of **poly(GA-Va-*co*-Ph)**.

Poly(GA-Va-co-BDMSB)

^{1}H NMR (400 MHz, Chloroform-*d*) δ 7.61 (s, 4H), 6.98 – 6.92 (m, 4H), 6.84 (s, 2H), 4.68 (s, 4H), 3.78 (s, 6H), 2.75 (t, *J* = 7.2 Hz, 4H), 2.20 (t, *J* = 7.2 Hz, 2H), 0.42 (s, 12H). **^{13}C{^{1}H} NMR (101 MHz, Chloroform-*d*)** δ 171.32, 151.04, 139.84, 139.26, 138.81, 133.07, 122.54, 118.64, 110.77, 64.87, 55.88, 33.01, 20.53, -1.59. **^{29}Si NMR (99 MHz, Chloroform-*d*)** δ 9.21.

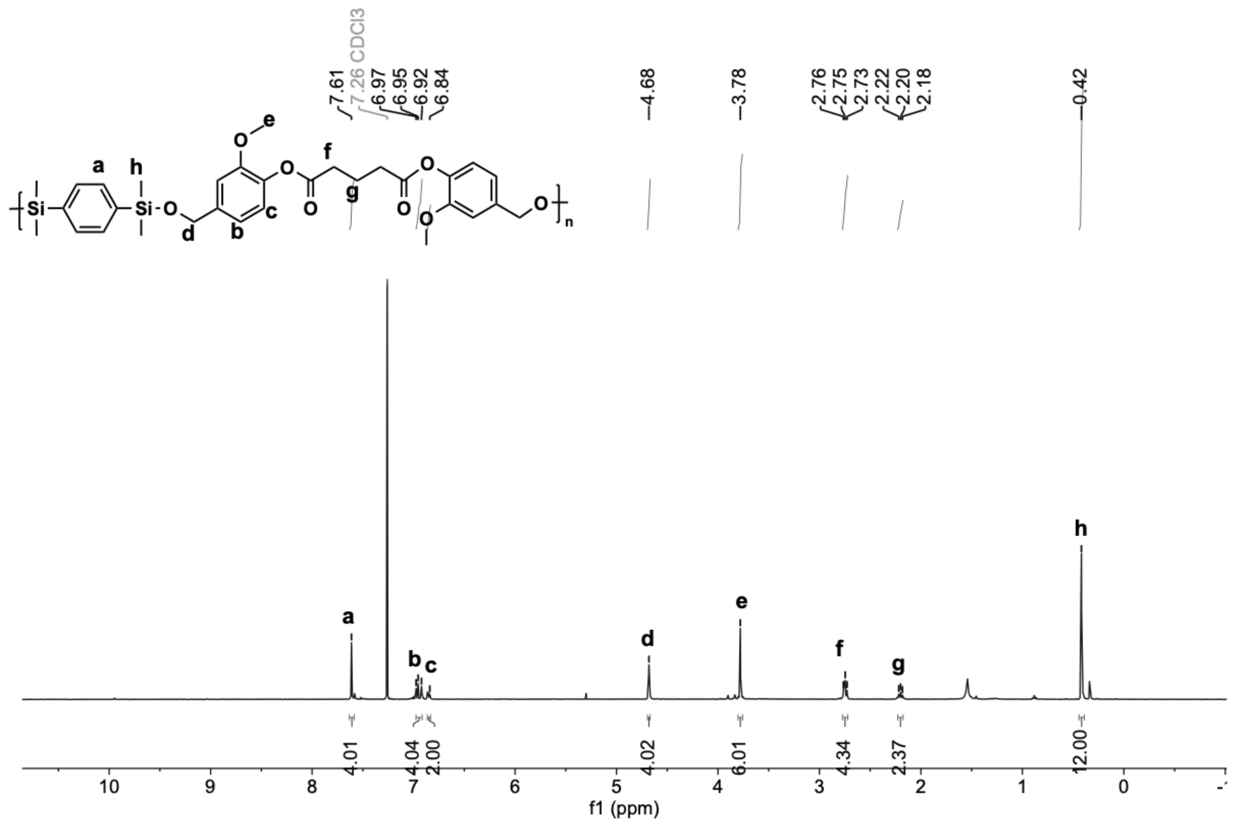

Figure S16 ^{1}H NMR spectrum (400 MHz, $CDCl_3$) of **poly(GA-Va-*co*-BDMSB)**.

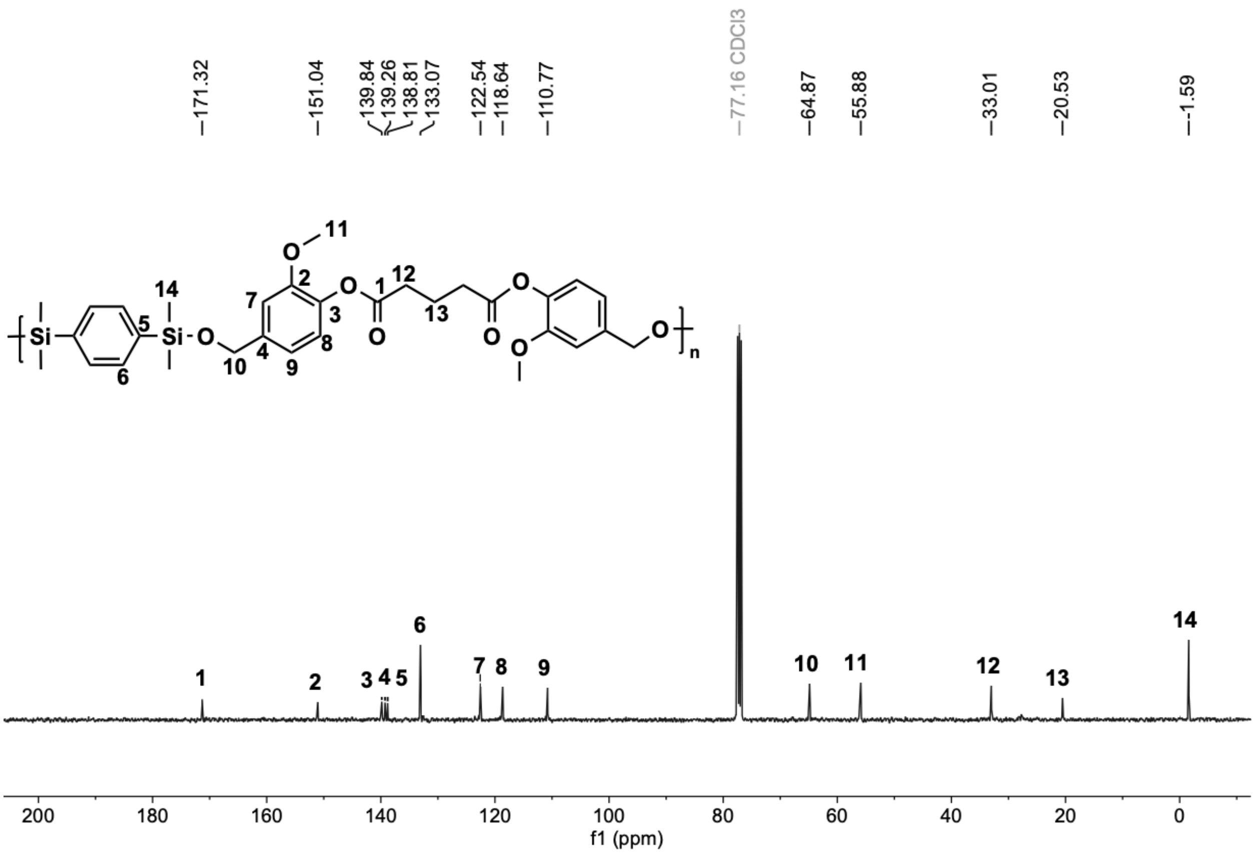

Figure S17 $^{13}C\{^{1}H\}$ NMR spectrum (101 MHz, $CDCl_3$) of **poly(GA-Va-*co*-BDMSB)**.

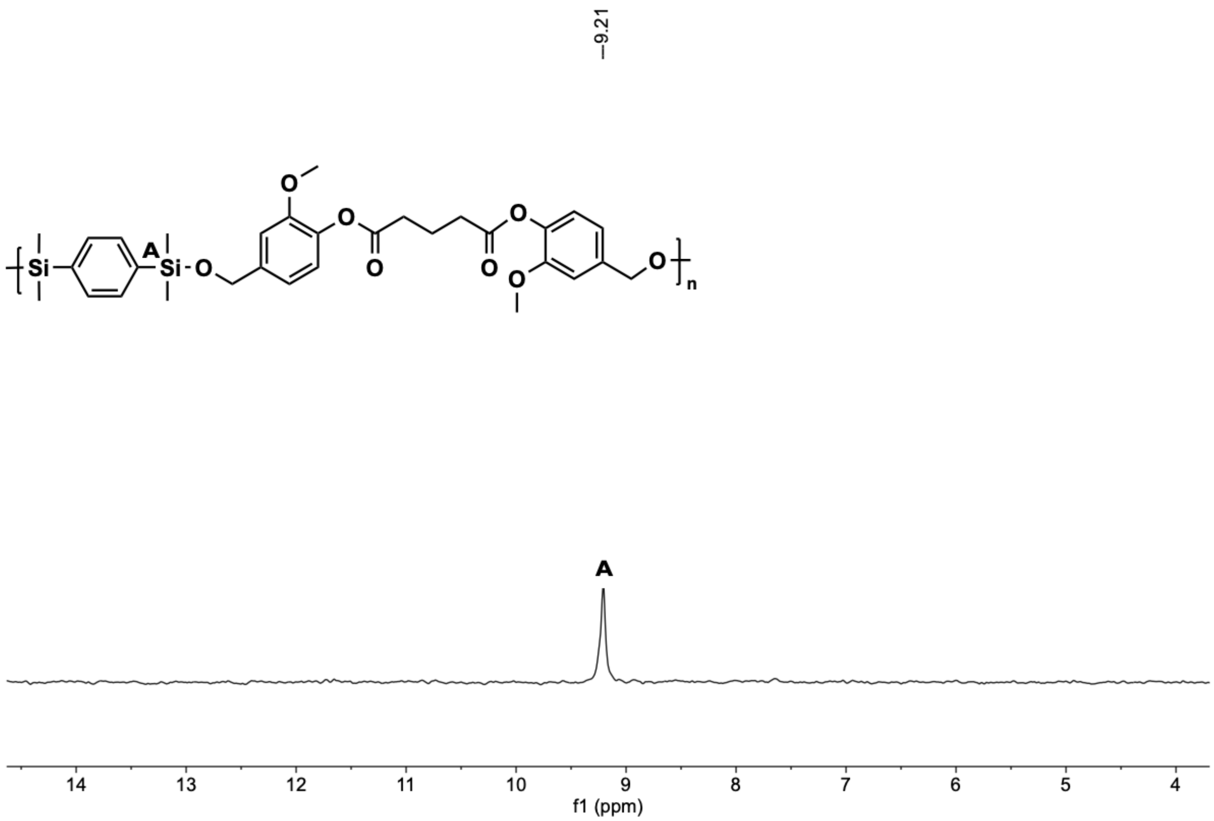

Figure S18 ^{29}Si NMR spectrum (99 MHz, $CDCl_3$) of **poly(GA-Va-*co*-BDMSB)**.

Poly(GA-Va-co-TMDS)

^{1}H NMR (400 MHz, Chloroform-*d*) δ 7.04 – 6.93 (m, 4H), 6.87 (dd, *J* = 1.9, 8.1 Hz, 2H), 4.74 (s, 4H), 3.81 (s, 6H), 2.75 (t, *J* = 7.3 Hz, 4H), 2.21 (t, *J* = 7.3 Hz, 2H), 0.16 (s, 12H). **^{13}C{^{1}H} NMR (101 MHz, Chloroform-*d*)** δ 7.04 – 6.93 (m, 4H), 6.87 (dd, *J* = 1.9, 8.1 Hz, 2H), 4.74 (s, 4H), 3.81 (s, 6H), 2.75 (t, *J* = 7.3 Hz, 4H), 2.21 (t, *J* = 7.3 Hz, 2H), 0.16 (s, 12H). **^{29}Si NMR (99 MHz, Chloroform-*d*)** δ -10.69.

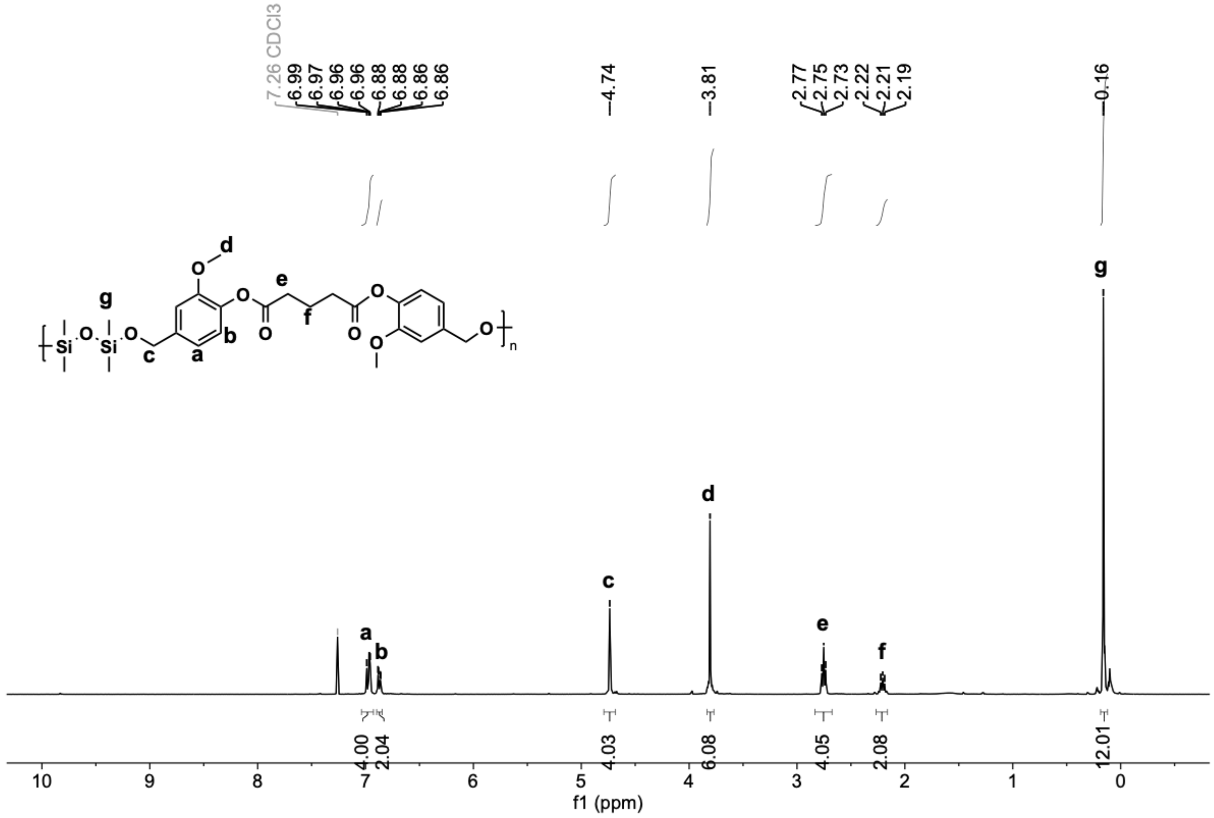

Figure S19 ^{1}H NMR spectrum (400 MHz, $CDCl_3$) of **poly(GA-Va-*co*-TMDS)**.

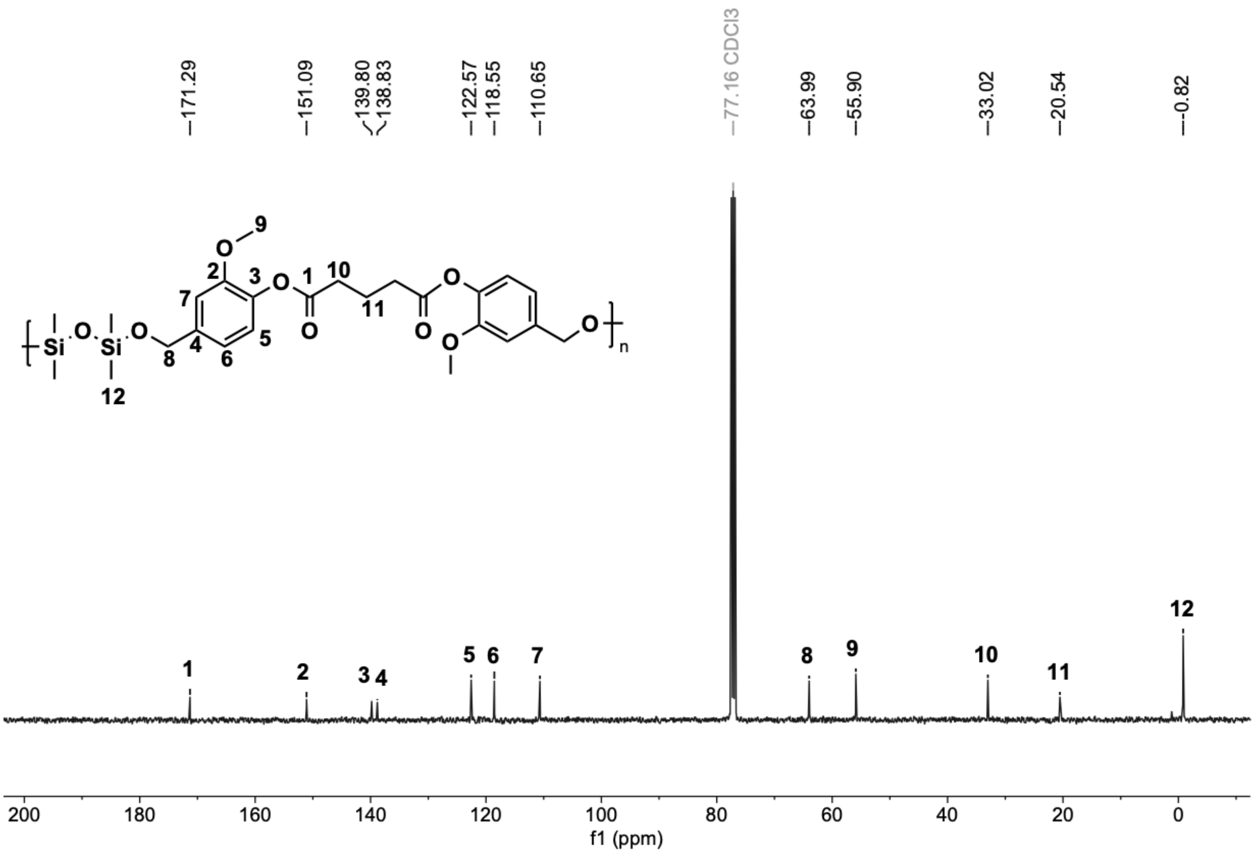

Figure S20 $^{13}C\{^1H\}$ NMR spectrum (101 MHz, $CDCl_3$) of **poly(GA-Va-*co*-TMDS)**.

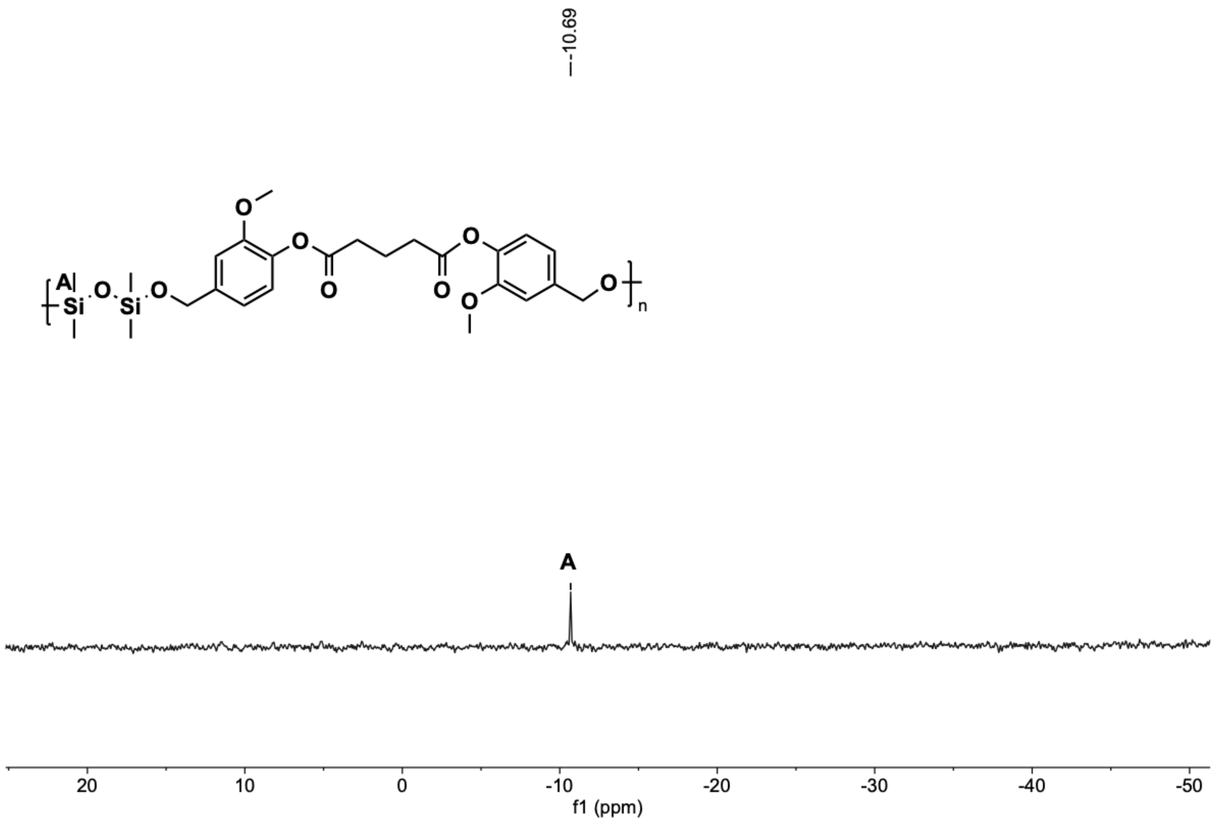

Figure S21 ^{29}Si NMR spectrum (99 MHz, $CDCl_3$) of **poly(GA-Va-*co*-TMDS)**.

Poly(GA-Sy-co-Ph)

^{1}H NMR (400 MHz, Chloroform-*d*) δ 7.78 – 7.67 (m, 1H), 7.52 – 7.37 (m, 1H), 6.56 (s, 1H), 4.79 (s, 1H), 3.72 (s, 3H), 2.82 – 2.71 (m, 1H), 2.22 (dd, J = 5.0, 9.7 Hz, 1H). **^{13}C{^{1}H} NMR (101 MHz, Chloroform-*d*)** δ 171.07, 152.17, 138.84, 135.09, 132.31, 130.79, 128.19, 127.69, 103.13, 65.27, 56.13, 32.94, 20.82. **^{29}Si NMR (99 MHz, Chloroform-*d*)** δ -30.25.

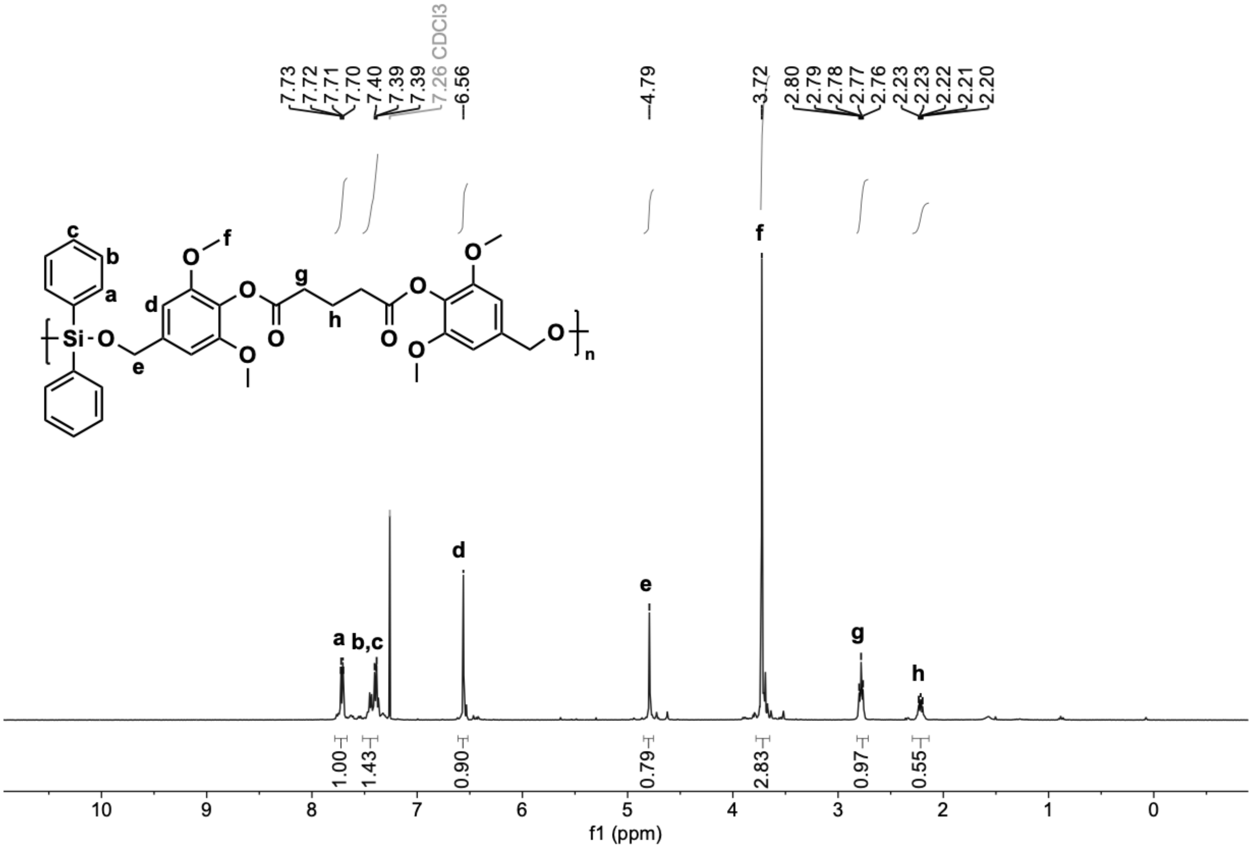

Figure S22 ^{1}H NMR spectrum (400 MHz, $CDCl_3$) of **poly(GA-Sy-*co*-Ph)**.

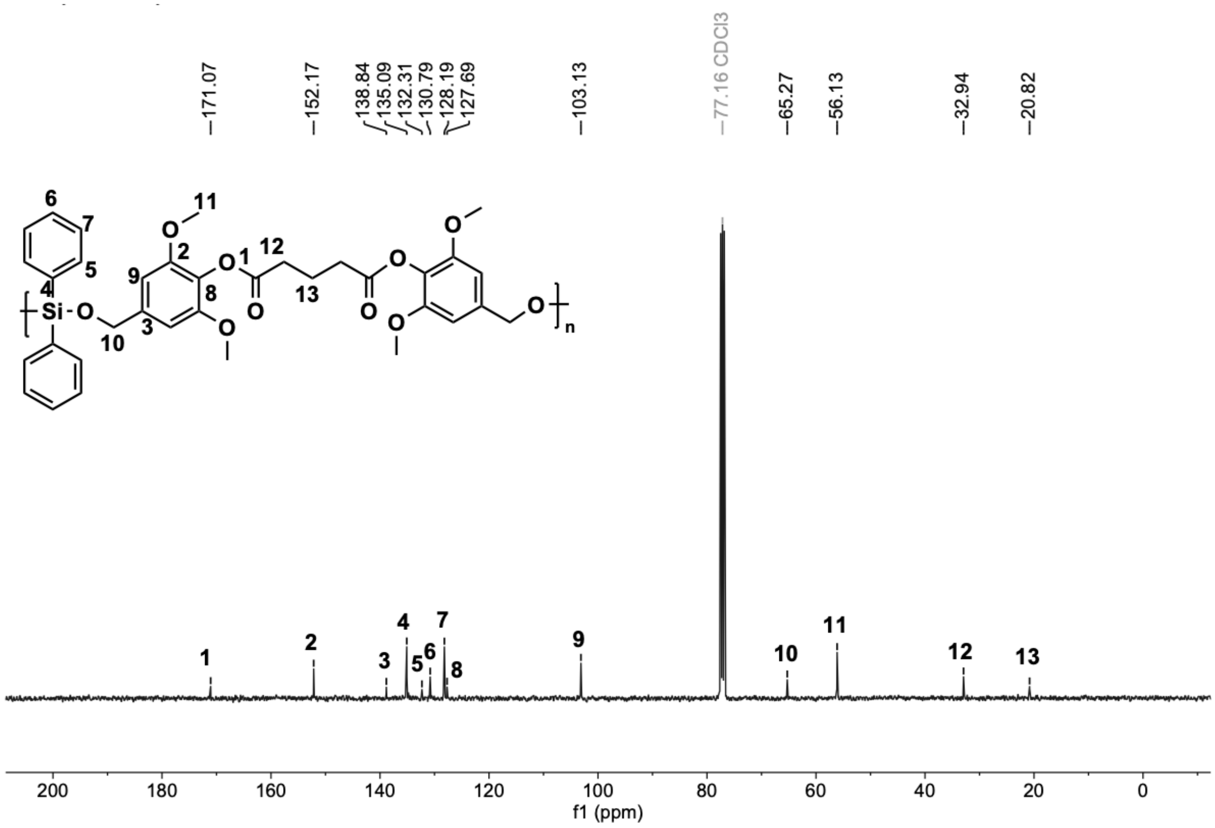

Figure S23 $^{13}C\{^{1}H\}$ NMR spectrum (101 MHz, $CDCl_3$) of **poly(GA-Sy-*co*-Ph).**

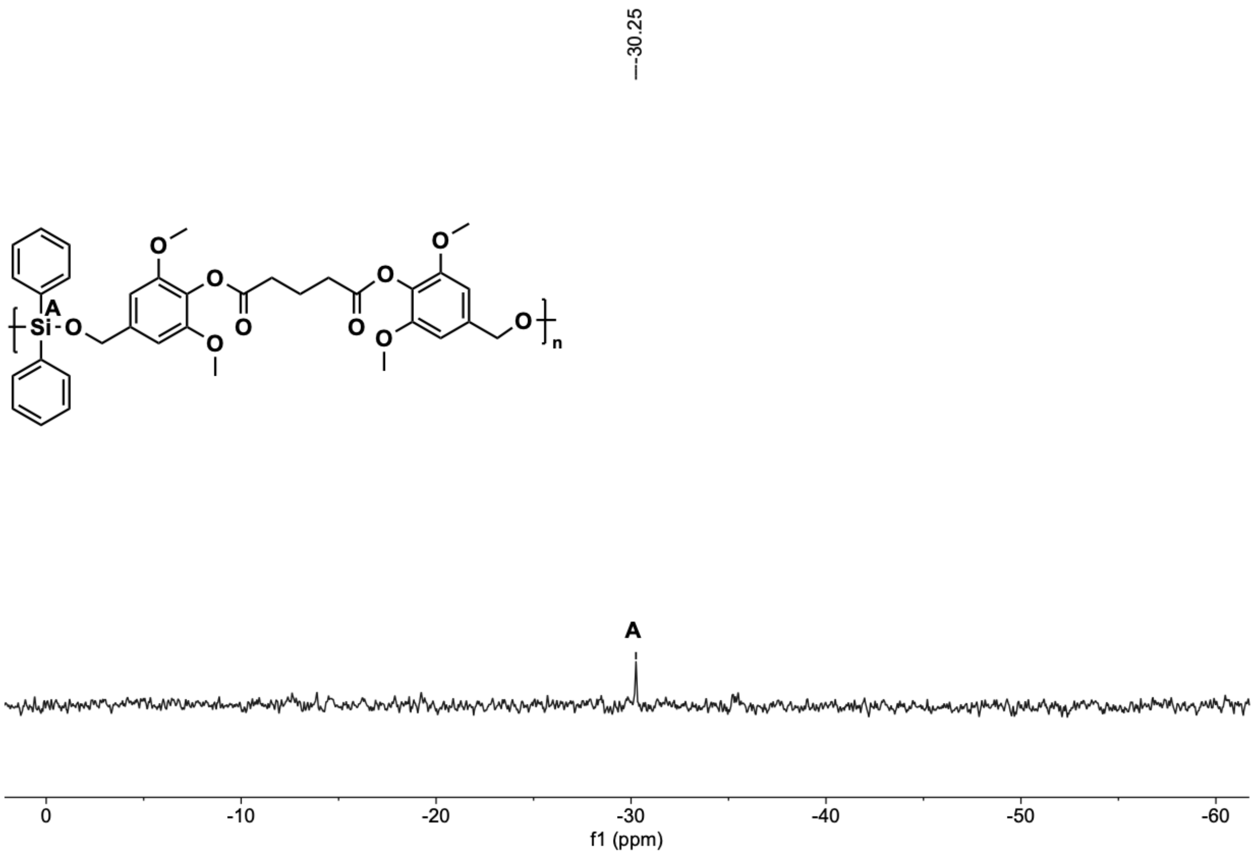

Figure S24 ^{29}Si NMR spectrum (99 MHz, $CDCl_3$) of **poly(GA-Sy-*co*-Ph)**.

Poly(GA-Sy-co-BDMSB)

^{1}H NMR (400 MHz, Chloroform-*d*) δ 7.62 (s, 4H), 6.54 (s, 4H), 4.67 (s, 4H), 3.77 (s, 12H), 2.79 (t, J = 7.2 Hz, 4H), 2.22 (t, J = 7.3 Hz, 2H), 0.43 (s, 12H). **^{13}C{^{1}H} NMR (101 MHz, Chloroform-*d*)** δ 171.13, 152.14, 139.29, 133.08, 127.64, 103.01, 65.24, 56.15, 32.94, 20.82, -1.58. **^{29}Si NMR (99 MHz, Chloroform-*d*)** δ 9.29.

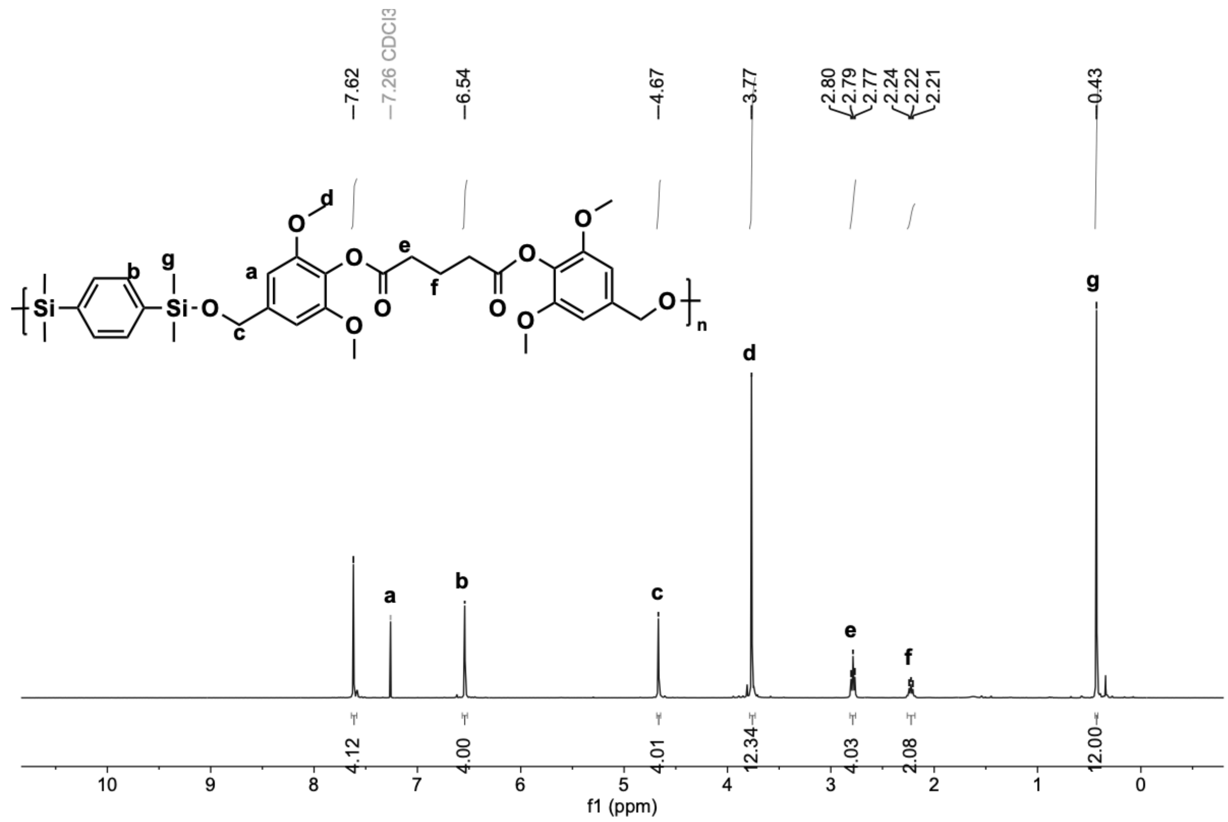

Figure S25 ^{1}H NMR spectrum (400 MHz, $CDCl_3$) of **poly(GA-Sy-*co*-BDMSB)**.

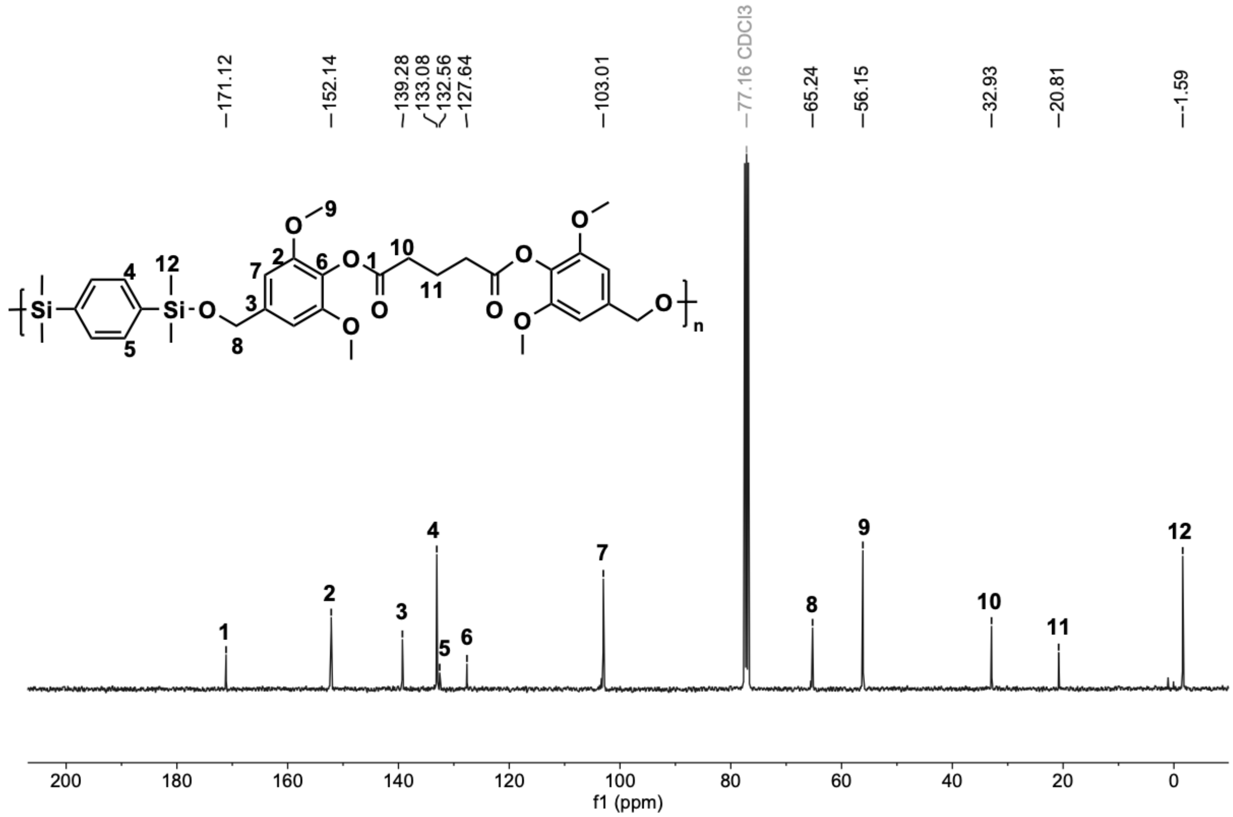

Figure S26 $^{13}C\{^{1}H\}$ NMR spectrum (101 MHz, $CDCl_3$) of **poly(GA-Sy-*co*-BDMSB)**.

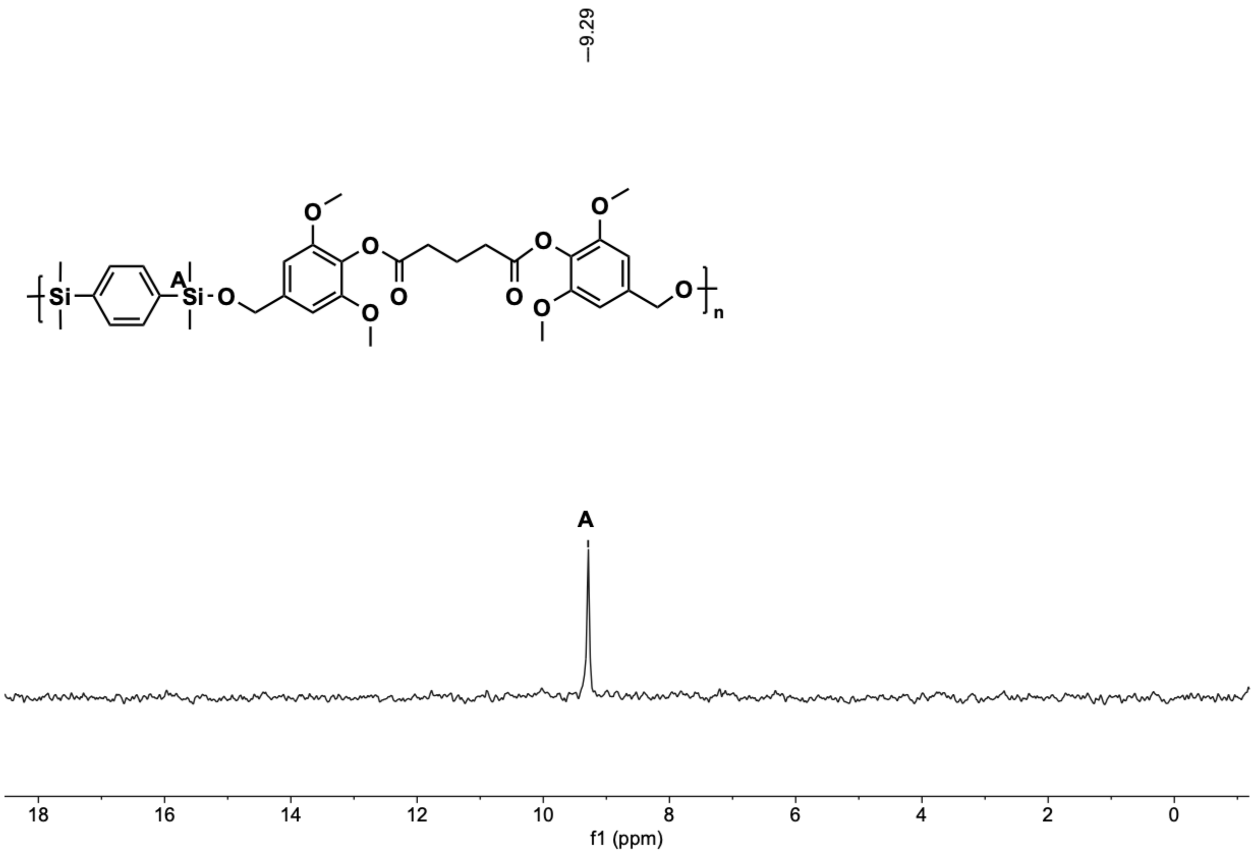

Figure S27 ^{29}Si NMR spectrum (99 MHz, $CDCl_3$) of **poly(GA-Sy-*co*-BDMSB)**.

Poly(GA-Sy-co-TMDS)

^{1}H NMR (400 MHz, Chloroform-*d*) δ 6.58 (s, 4H), 4.72 (s, 4H), 3.79 (s, 12H), 2.79 (t, *J* = 7.3 Hz, 4H), 2.23 (dd, *J* = 6.2, 8.3 Hz, 2H), 0.18 (s, 12H). **^{13}C{^{1}H} NMR (101 MHz, Chloroform-*d*)** δ 171.09, 152.19, 139.27, 127.64, 102.82, 64.28, 56.16, 32.94, 20.81. **^{29}Si NMR (99 MHz, Chloroform-*d*)** δ -10.59.

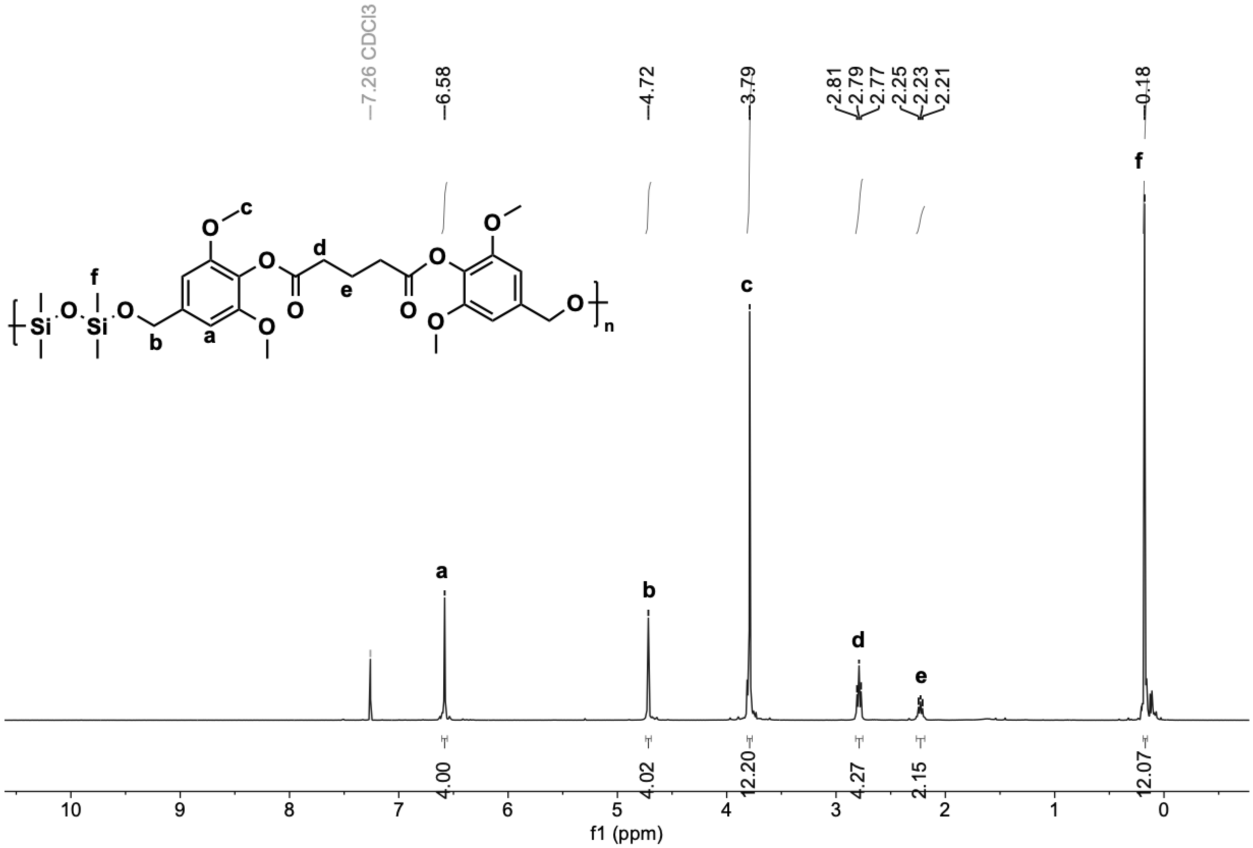

Figure S28 ^{1}H NMR spectrum (400 MHz, $CDCl_3$) of **poly(GA-Sy-*co*-TMDS)**.

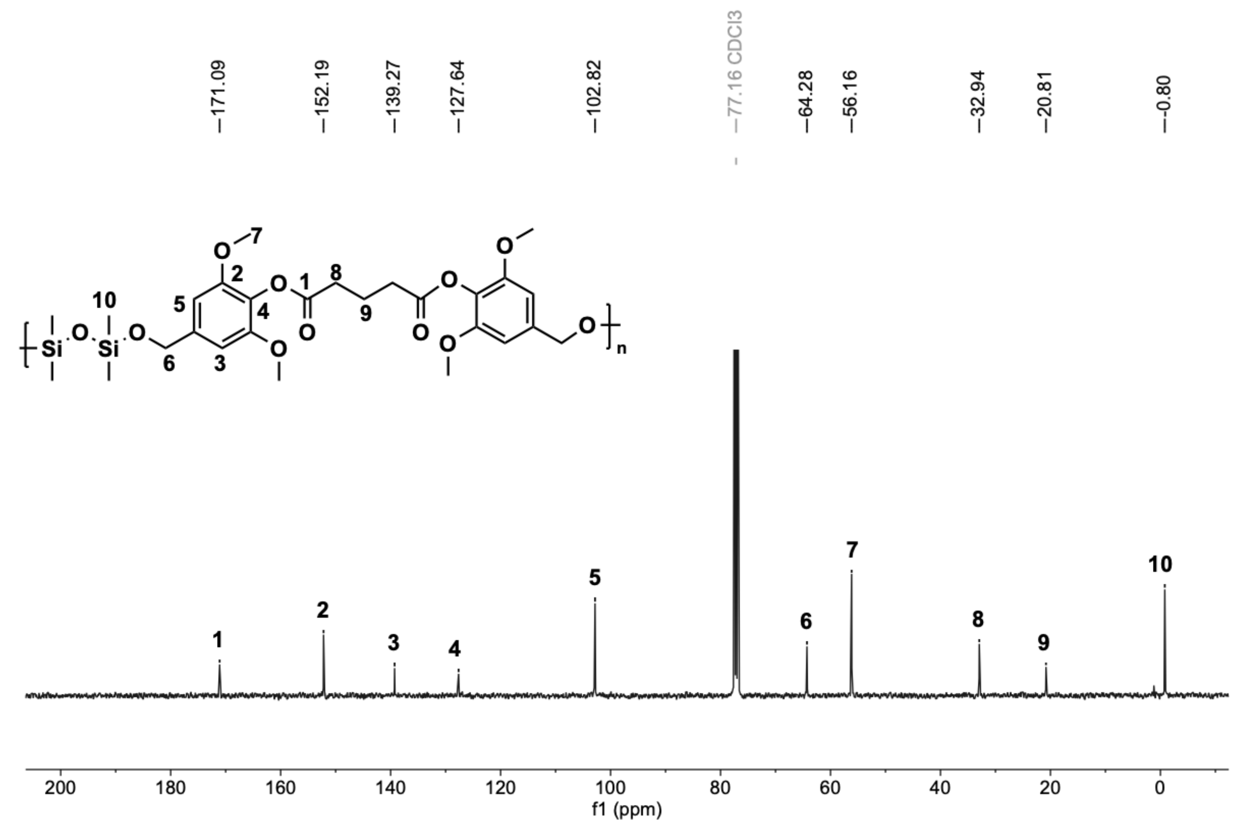

Figure S29 $^{13}C\{^{1}H\}$ NMR spectrum (101 MHz, $CDCl_3$) of **poly(GA-Sy-*co*-TMDS)**.

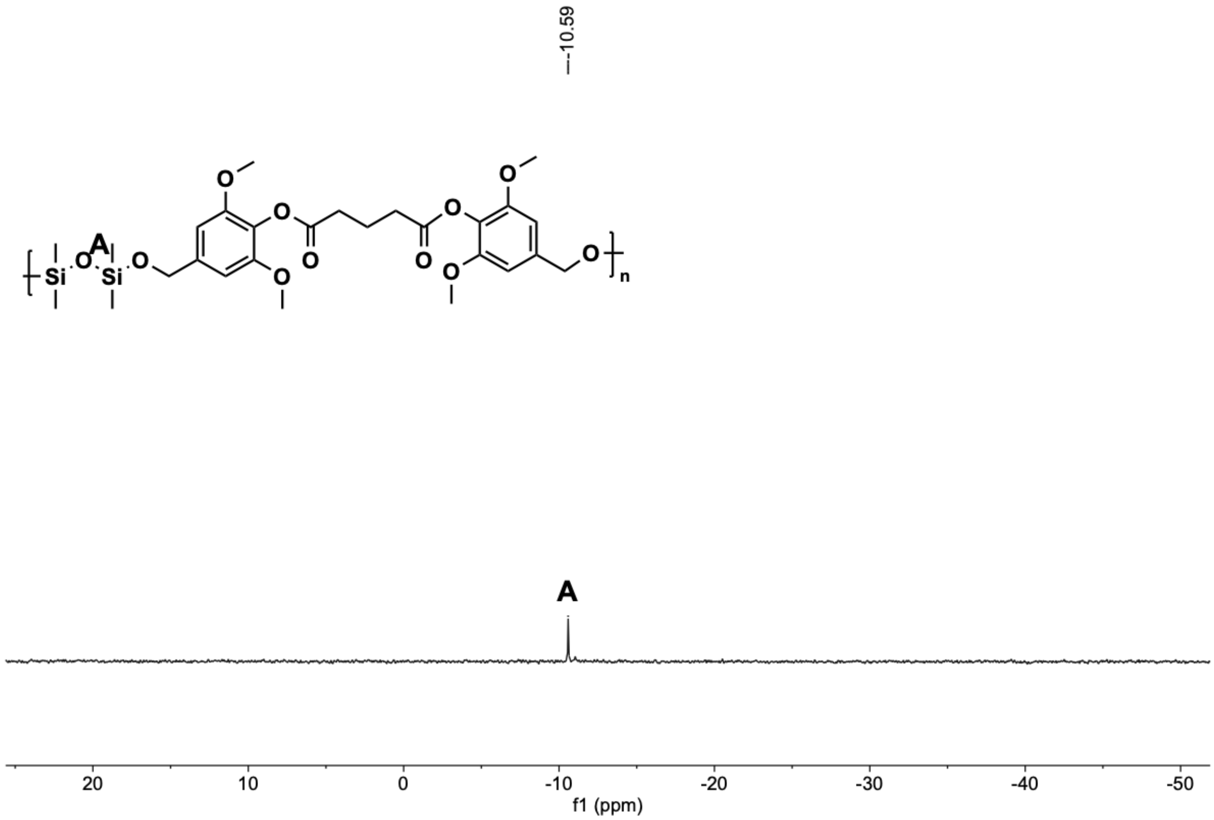

Figure S30 ^{29}Si NMR spectrum (99 MHz, $CDCl_3$) of **poly(GA-Sy-*co*-TMDS)**.

Poly(AA-Va-co-Ph)

^{1}H NMR (400 MHz, Chloroform-*d*) δ 7.74 - 7.68 (m, 4H), 7.45 - 7.36 (m, 6H), 6.99 - 6.88 (m, 6H), 4.81 (s, 4H), 3.73 (s, 6H), 2.64 (s, 5H), 1.90 (s, 4H). **^{13}C{^{1}H} NMR (101 MHz, Chloroform-*d*)** δ 171.59, 151.08, 139.32, 138.91, 135.10, 132.25, 130.75, 128.17, 122.64, 118.75, 110.82, 64.88, 55.87, 33.78, 24.50. **^{29}Si NMR (99 MHz, Chloroform- *d*)** δ -30.29.

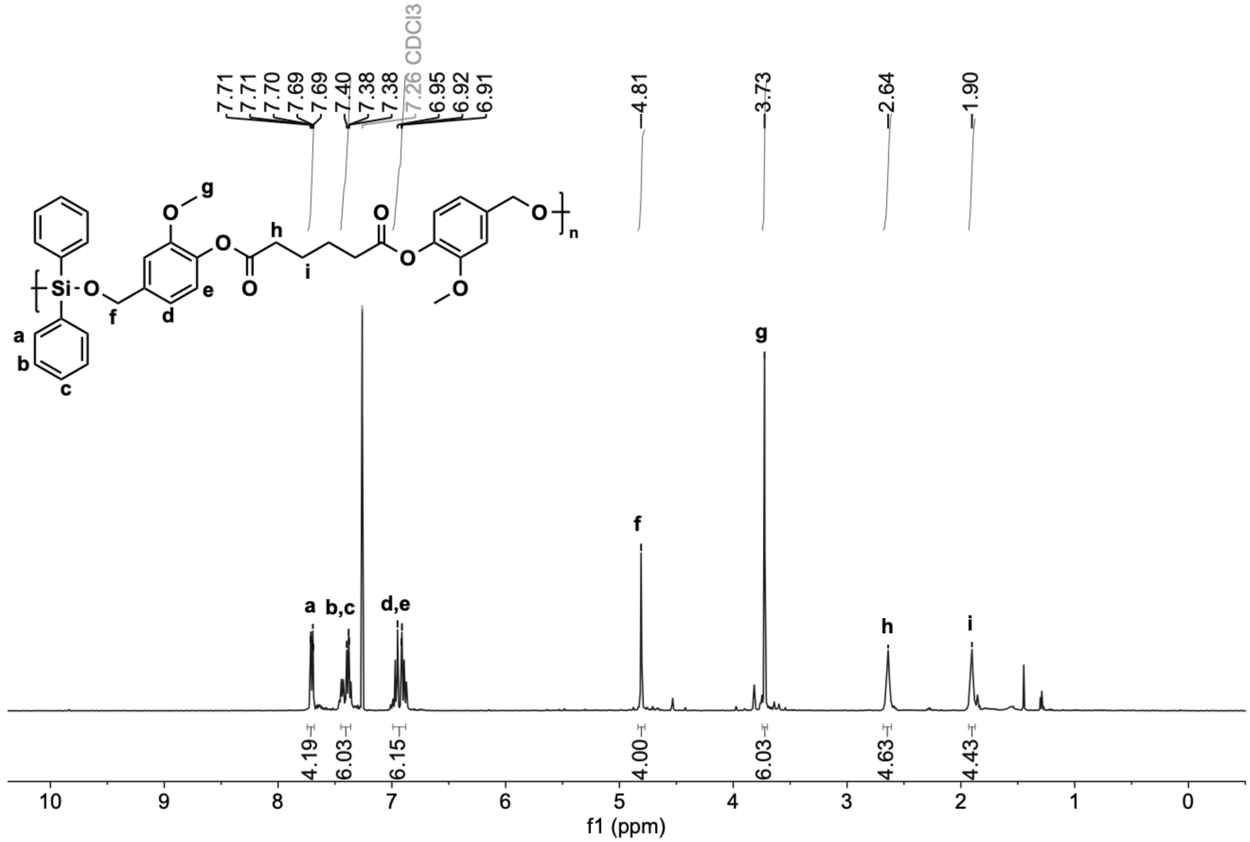

Figure S31 ^{1}H NMR spectrum (400 MHz, $CDCl_3$) of **poly(AA-Va-*co*-Ph)**.

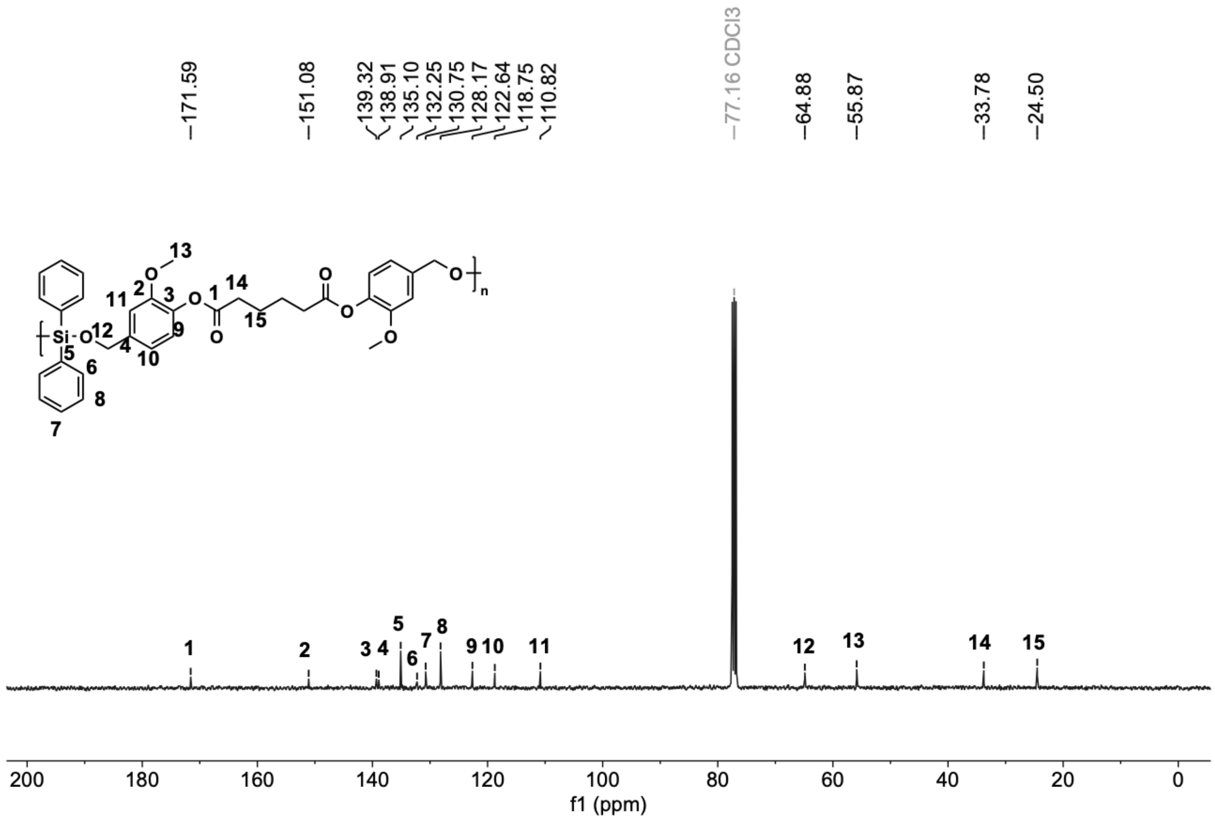

Figure S32 $^{13}C\{^{1}H\}$ NMR spectrum (101 MHz, $CDCl_3$) of **poly(AA-Va-*co*-Ph)**.

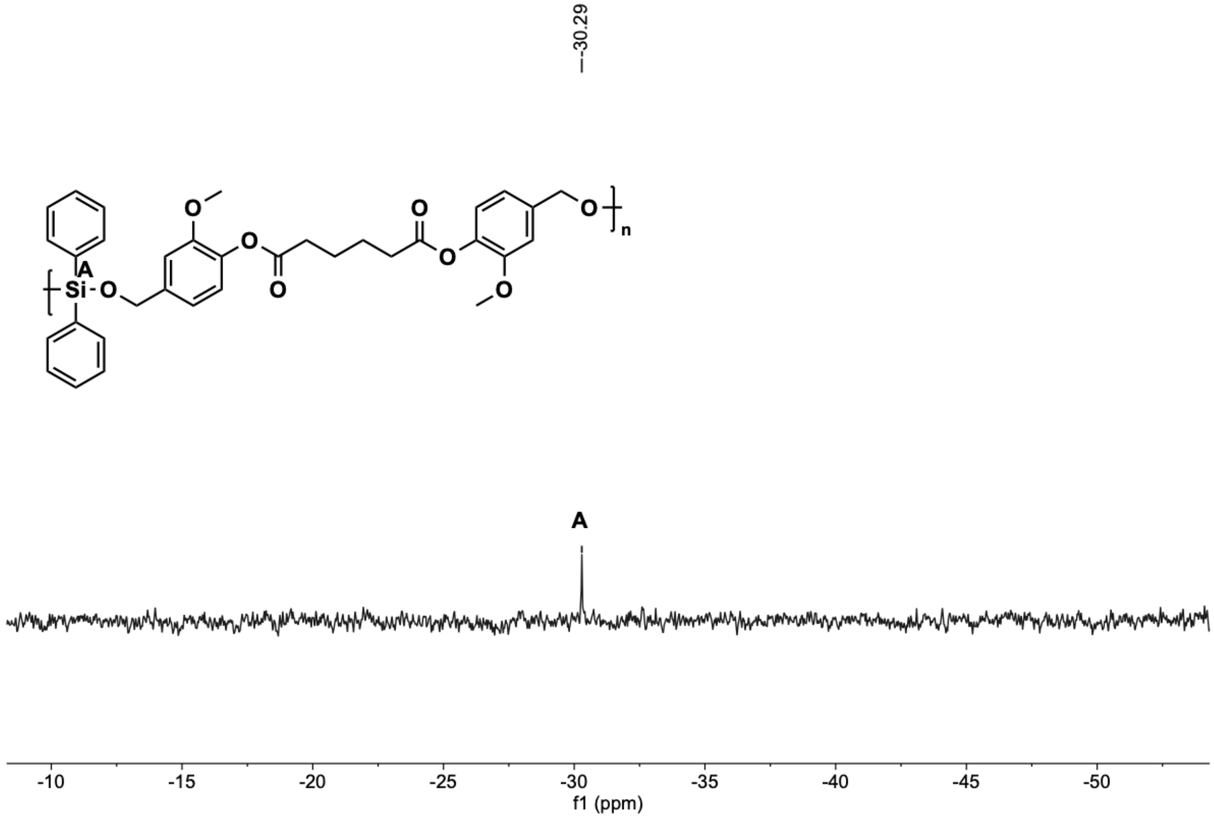

Figure S33 ^{29}Si NMR spectrum (99 MHz, $CDCl_3$) of **poly(AA-Va-*co*-Ph)**.

Poly(AA-Va-co-BDMSB)

^{1}H NMR (400 MHz, Chloroform-*d*) δ 7.54 (s, 4H), 7.03 – 6.85 (m, 6H), 4.68 (s, 4H), 3.83 (s, 6H), 2.66 (s, 4H), 1.92 (s, 4H), 0.33 (s, 12H). **^{13}C{^{1}H} NMR (101 MHz, Chloroform-*d*)** δ 171.61, 151.35, 140.99, 139.98, 139.26, 132.38, 122.86, 119.12, 111.14, 65.25, 55.97, 33.79, 24.50, 1.05. **^{29}Si NMR (99 MHz, Chloroform-*d*)** δ -1.27.

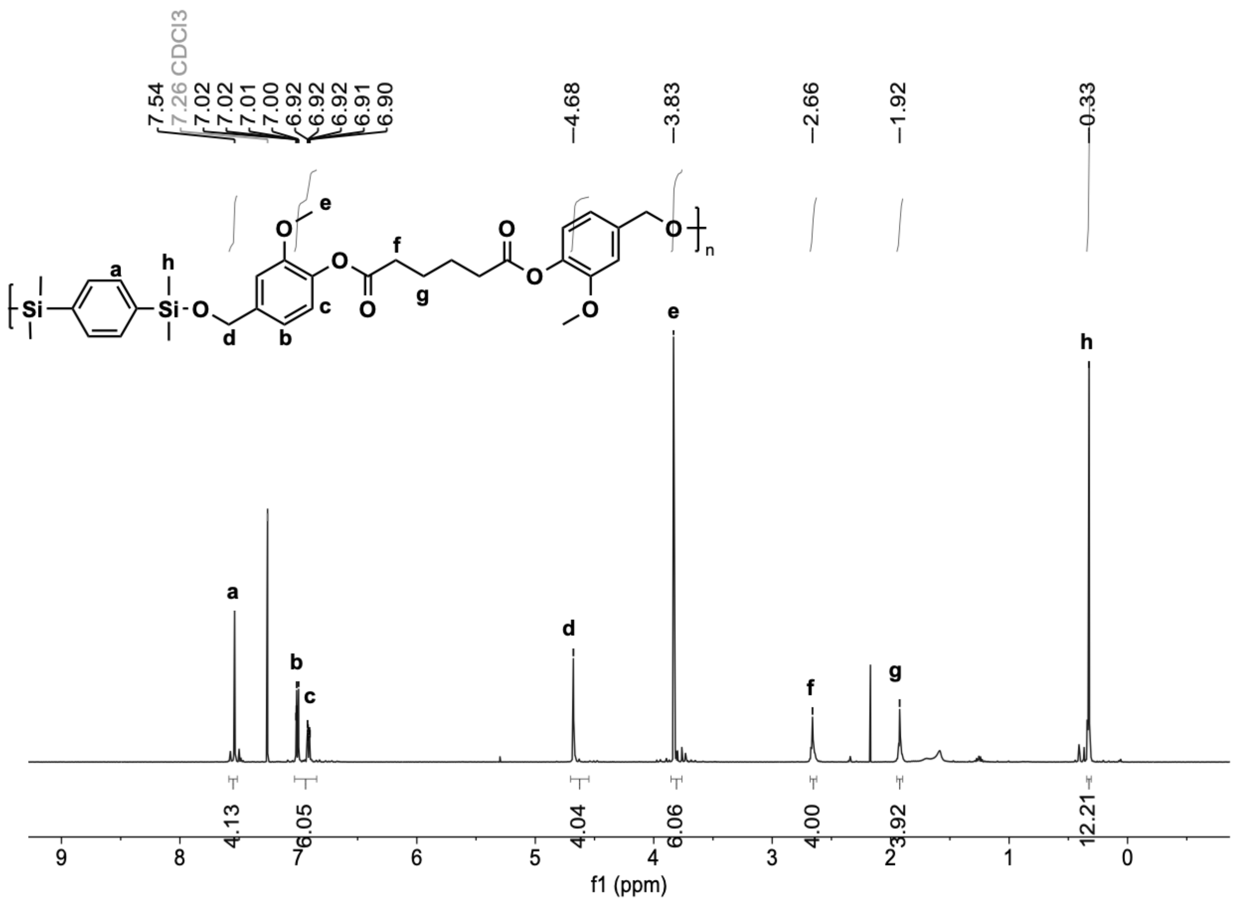

Figure S34 ^{1}H NMR spectrum (400 MHz, $CDCl_3$) of **poly(AA-Va-*co*-BDMSB)**.

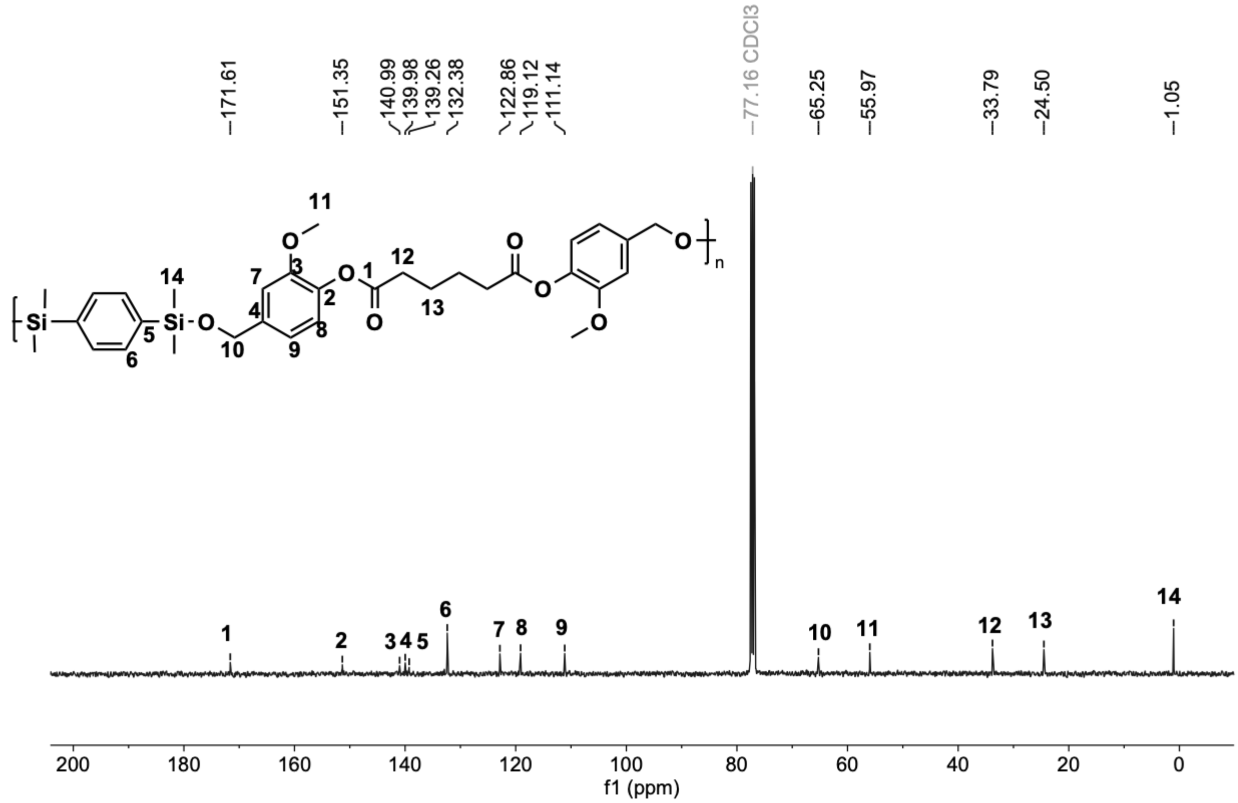

Figure S35 $^{13}C\{^{1}H\}$ NMR spectrum (101 MHz, $CDCl_3$) of **poly(AA-Va-*co*-BDMSB)**.

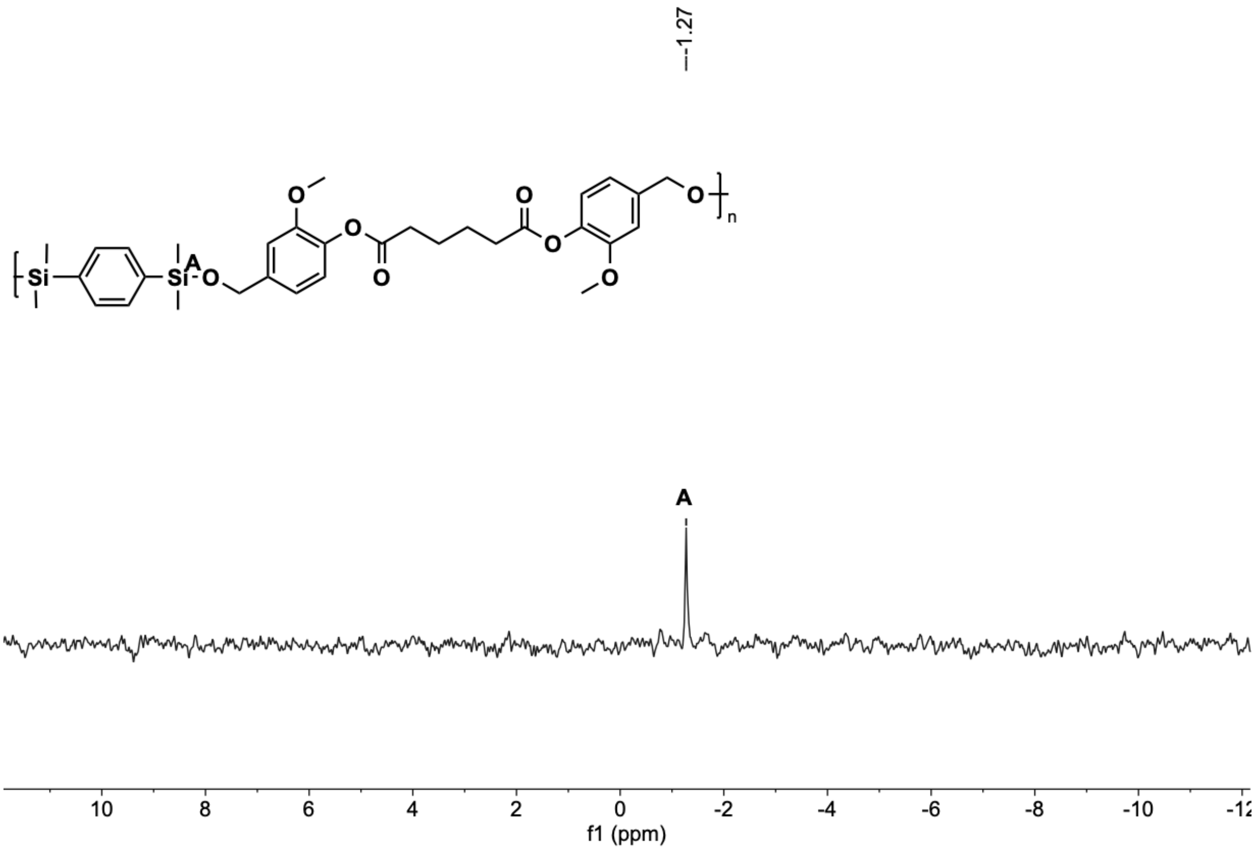

Figure S36 ^{29}Si NMR spectrum (99 MHz, $CDCl_3$) of **poly(AA-Va-*co*-BDMSB)**.

Poly(AA-Va-co-TMDS)

^{1}H NMR (400 MHz, Chloroform-*d*) δ 7.03 - 6.81 (m, 6H), 4.73 (s, 4H), 3.80 (s, 6H), 2.74 - 2.58 (m, 4H), 1.91 (d, *J* = 3.3 Hz, 4H), 0.16 (s, 12H). **^{13}C{^{1}H} NMR (101 MHz, Chloroform-*d*)** δ 171.60, 151.11, 139.73, 138.83, 122.57, 118.53, 110.63, 63.98, 55.89, 33.77,24.48. **^{29}Si NMR (99 MHz, Chloroform-*d*)** δ -10.70.

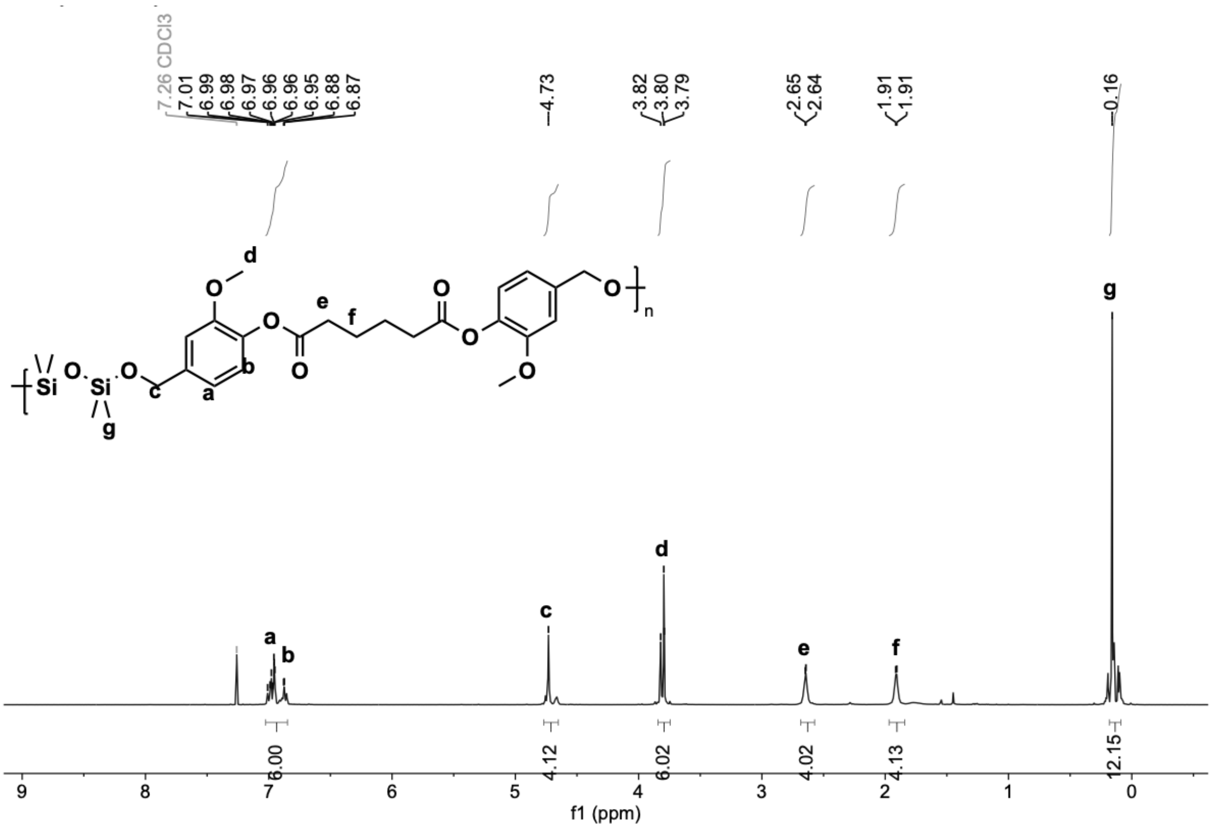

Figure S37 ^{1}H NMR spectrum (400 MHz, $CDCl_3$) of **poly(AA-Va-*co*-TMDS)**.

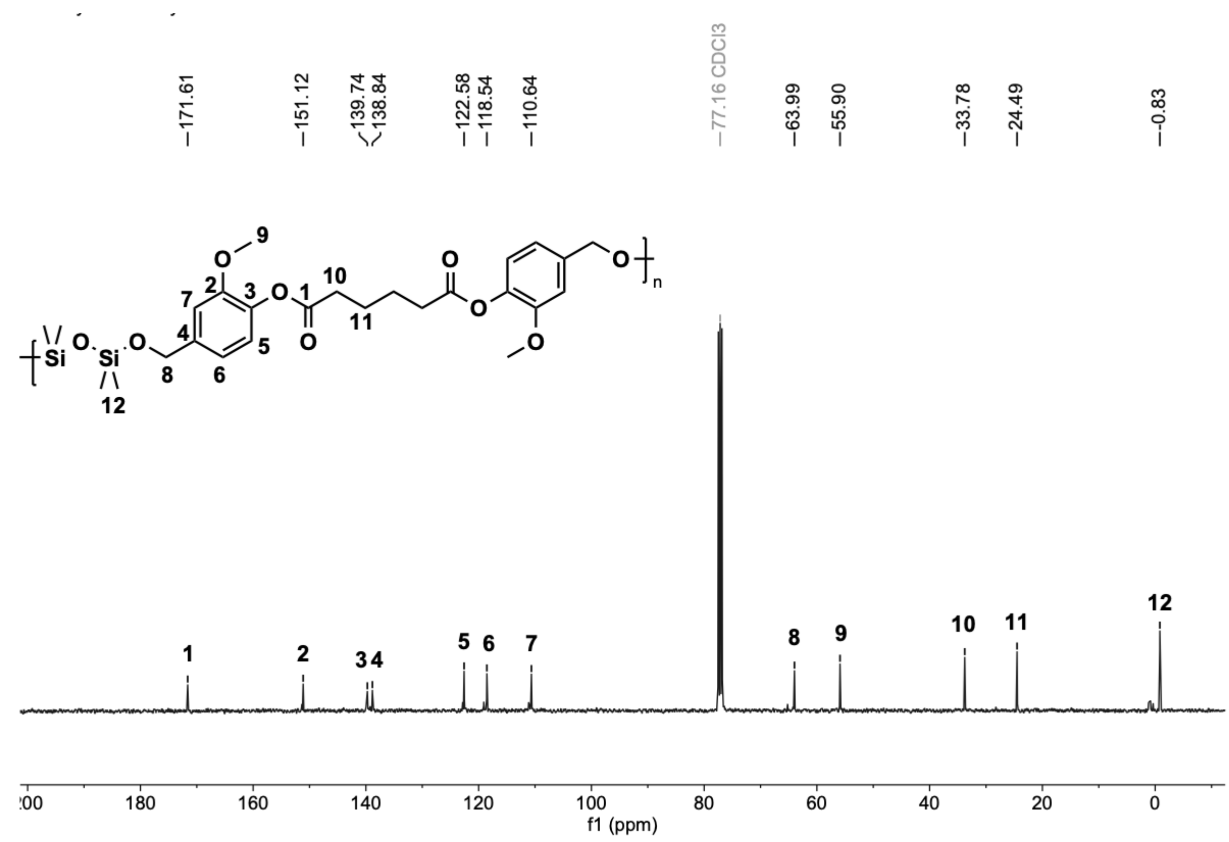

Figure S38 $^{13}C\{^{1}H\}$ NMR spectrum (101 MHz, $CDCl_3$) of **poly(AA-Va-*co*-TMDS)**.

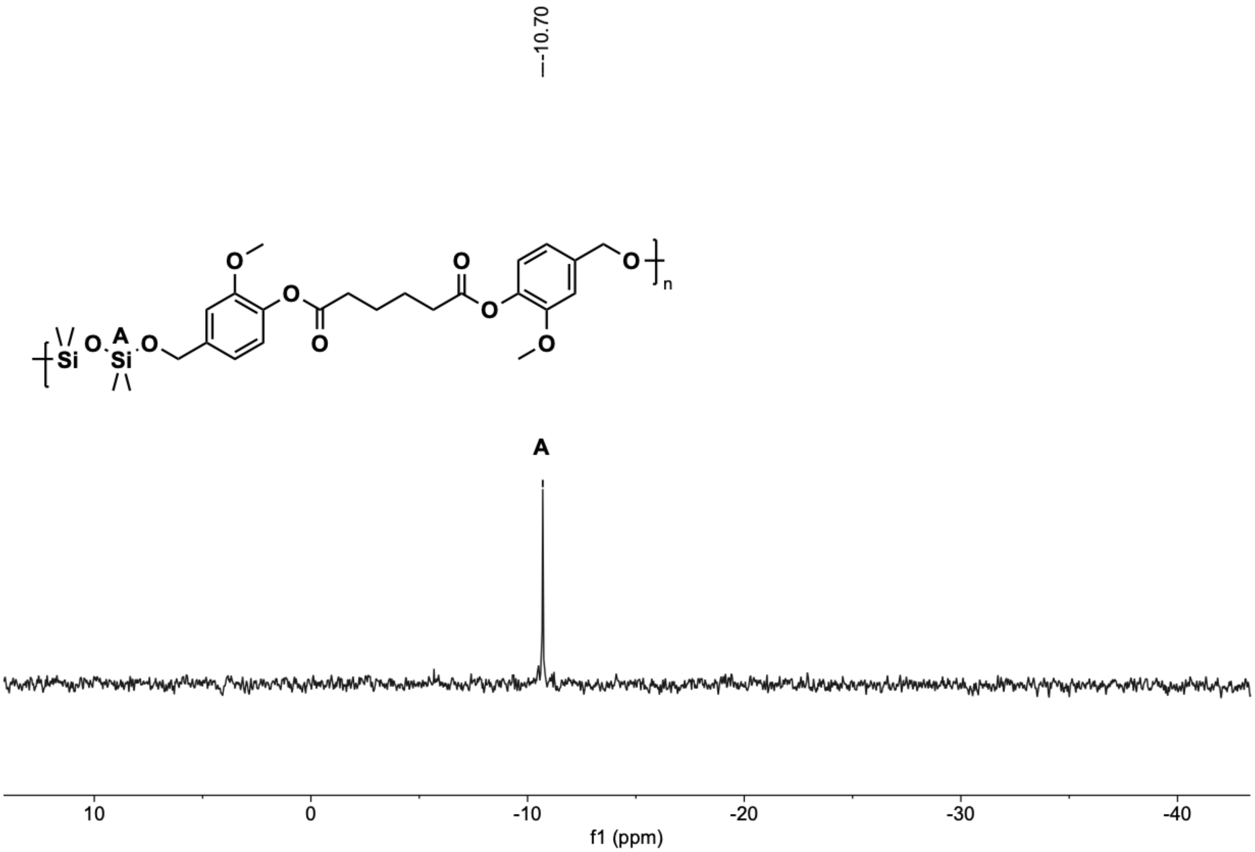

Figure S39 ^{29}Si NMR spectrum (99 MHz, $CDCl_3$) of **poly(AA-Va-*co*-TMDS)**.

Poly(AA-Sy-co-Ph)

^{1}H NMR (400 MHz, Chloroform-*d*) δ 7.75 – 7.67 (m, 4H), 7.45 – 7.35 (m, 6H), 6.56 (s, 4H), 4.80 (s, 4H), 3.72 (s, 12H), 2.67 (t, *J* = 3.0 Hz, 4H), 1.98 – 1.87 (m, 4H). **^{13}C{^{1}H} NMR (101 MHz, Chloroform-*d*)** δ 171.38, 152.17, 138.79, 135.08, 132.30, 130.78, 128.18, 127.69, 103.12, 65.27, 56.12, 33.63, 24.53. **^{29}Si NMR (99 MHz, Chloroform-*d*)** δ -30.24.

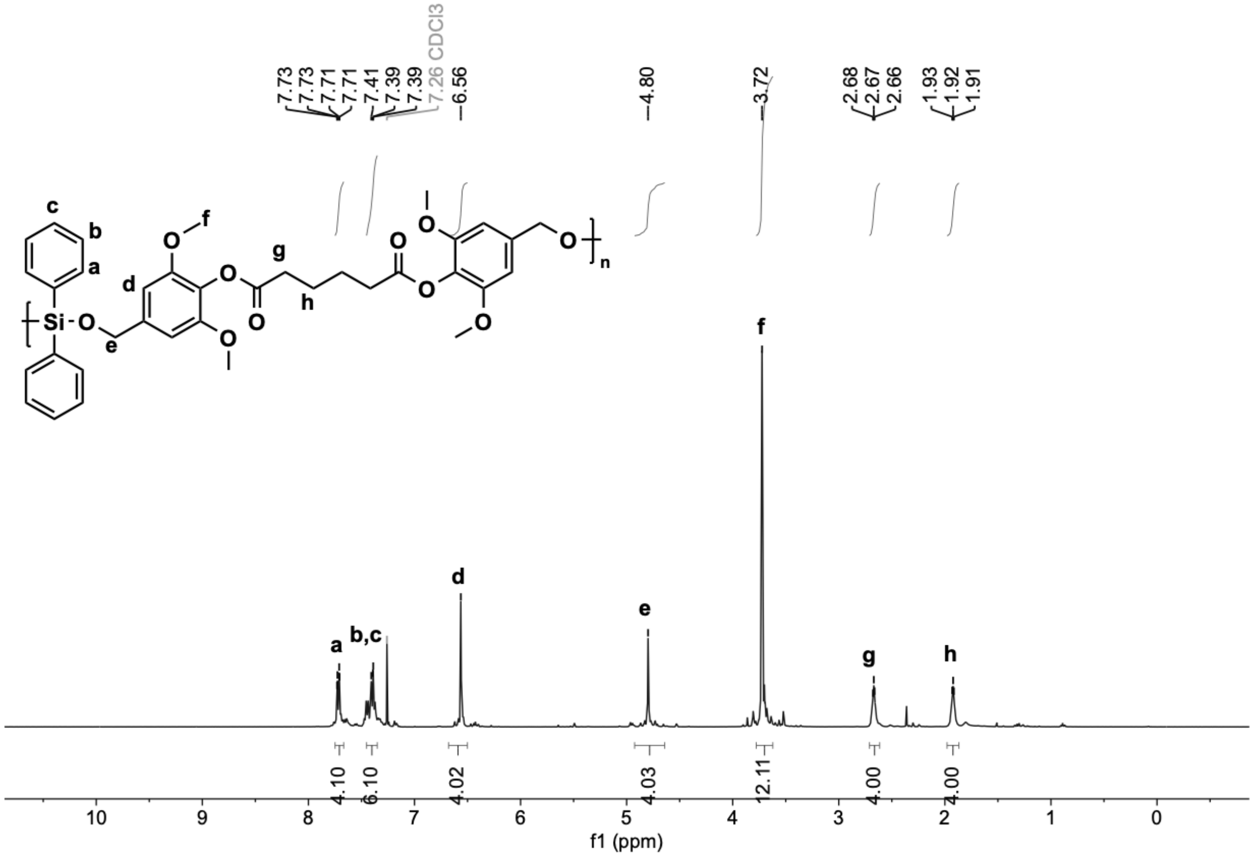

Figure S40 ^{1}H NMR spectrum (400 MHz, $CDCl_3$) of **poly(AA-Sy-*co*-Ph)**.

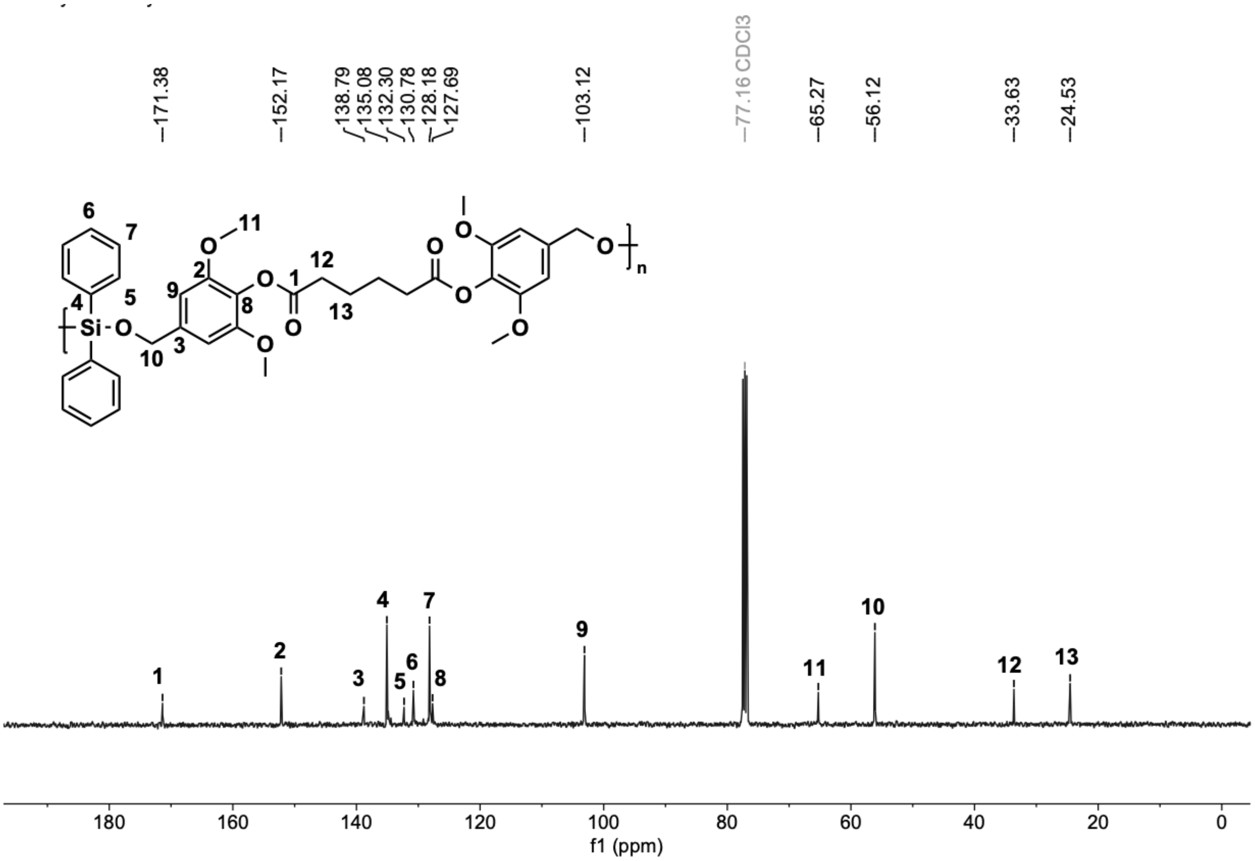

Figure S41 $^{13}C\{^1H\}$ NMR spectrum (101 MHz, $CDCl_3$) of **poly(AA-Sy-*co*-Ph)**.

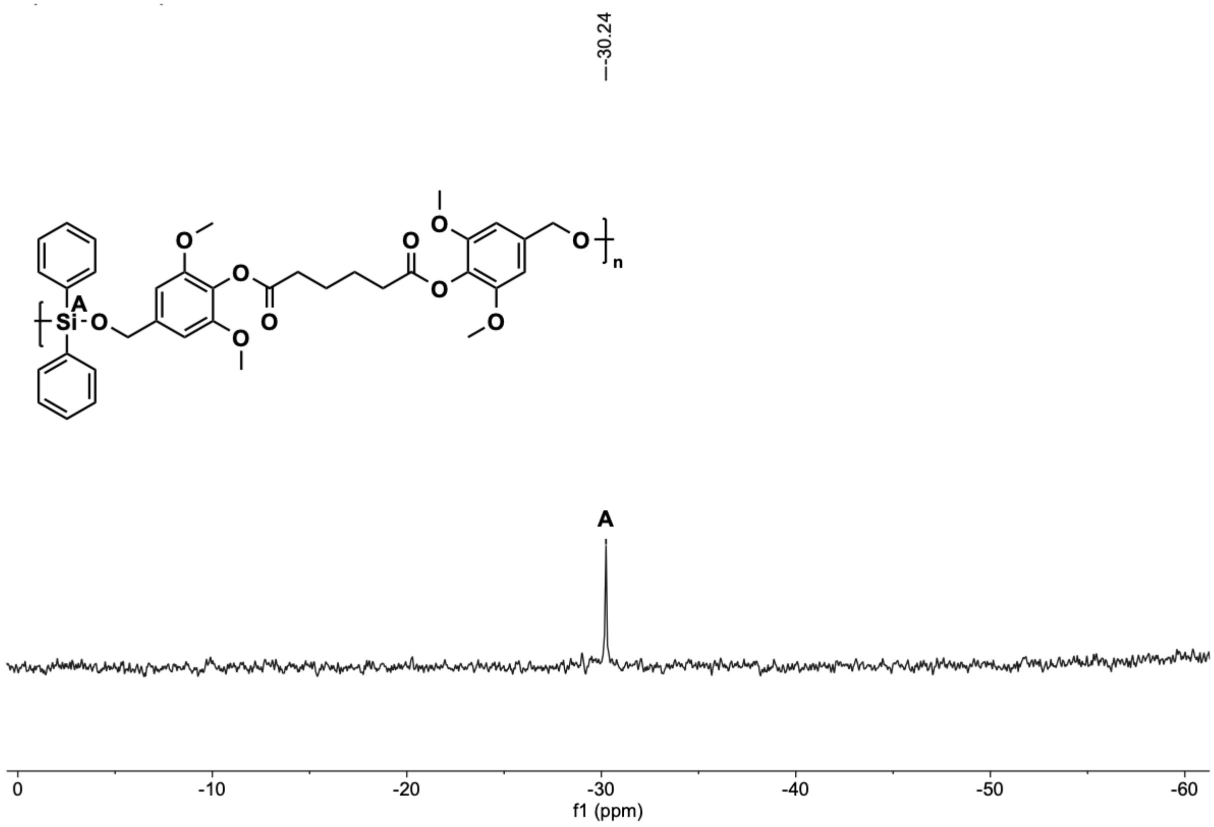

Figure S42 ^{29}Si NMR spectrum (99 MHz, $CDCl_3$) of **poly(AA-Sy-*co*-Ph)**.

Poly(AA-Sy-co-BDMSB)

^{1}H NMR (400 MHz, Chloroform-*d*) δ 7.60 (d, *J* = 11.8 Hz, 4H), 6.54 (s, 4H), 4.67 (s, 4H), 3.76 (s, 12H), 2.68 (d, *J* = 6.2 Hz, 4H), 1.93 (q, *J* = 3.4 Hz, 4H), 0.43 (d, *J* = 2.2 Hz, 12H). **^{13}C{^{1}H} NMR (101 MHz, Chloroform-*d*)** δ 171.35, 152.14, 139.20, 133.58, 133.05, 127.62, 102.98, 65.23, 56.12, 33.63, 24.54, -1.61. **^{29}Si NMR (99 MHz, Chloroform-*d*)** δ 9.27.

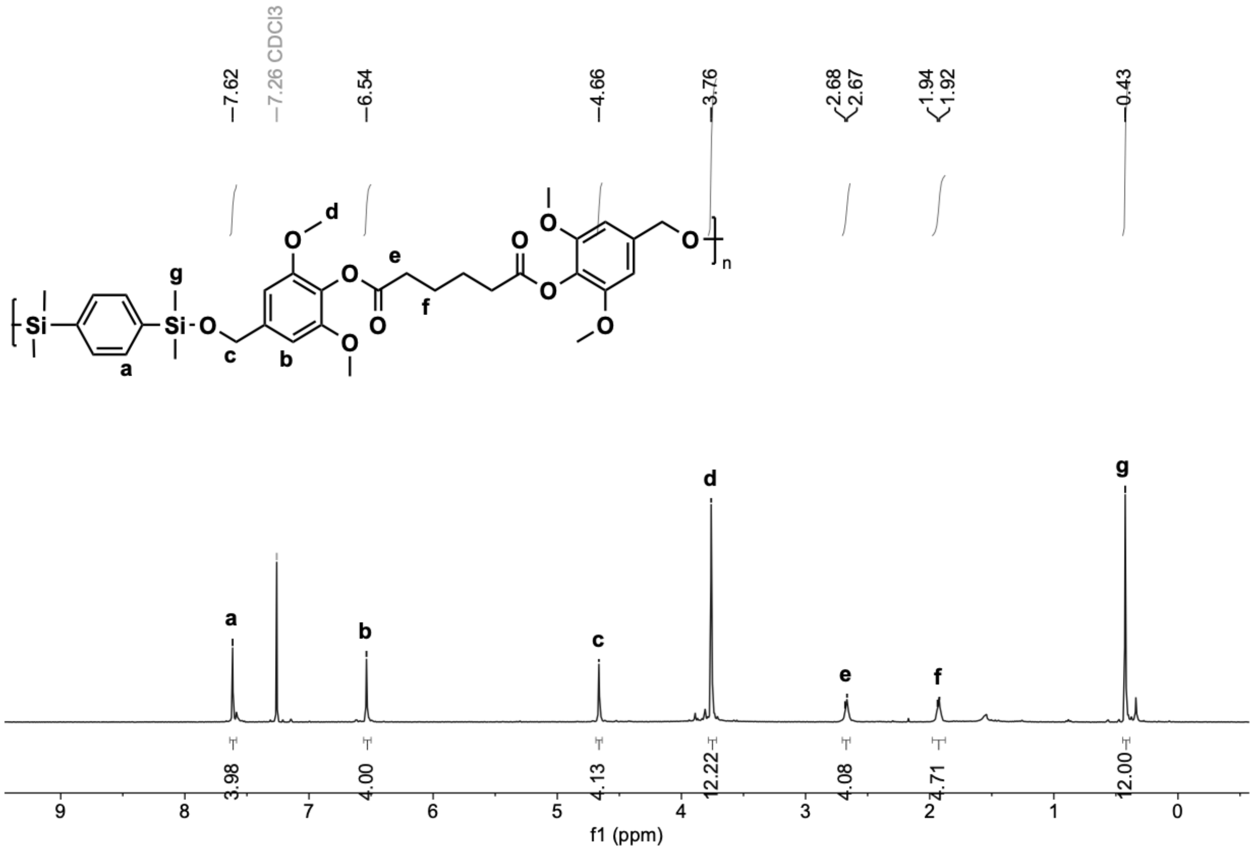

Figure S43 ^{1}H NMR spectrum (400 MHz, $CDCl_3$) of **poly(AA-Sy-*co*-BDMSB)**.

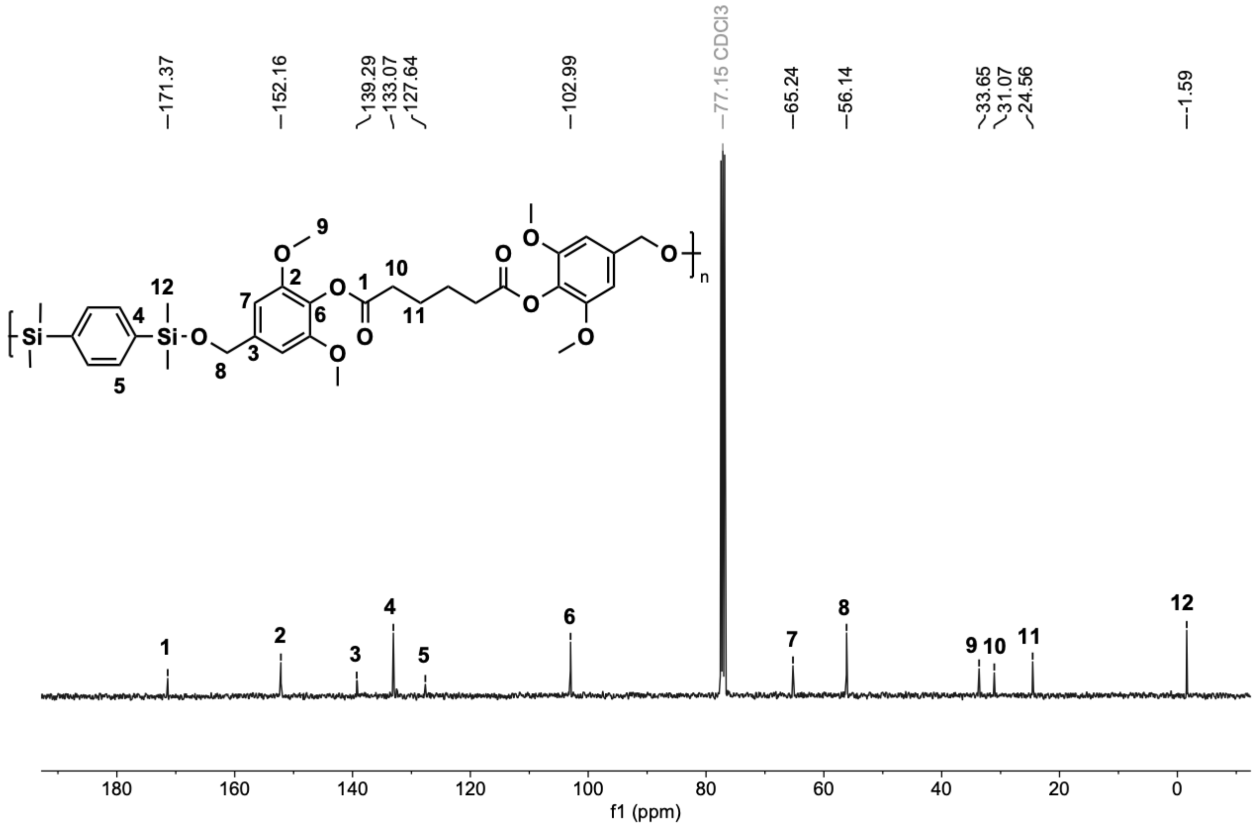

Figure S44 $^{13}C\{^{1}H\}$ NMR spectrum (101 MHz, $CDCl_3$) of **poly(AA-Sy-*co*-BDMSB)**.

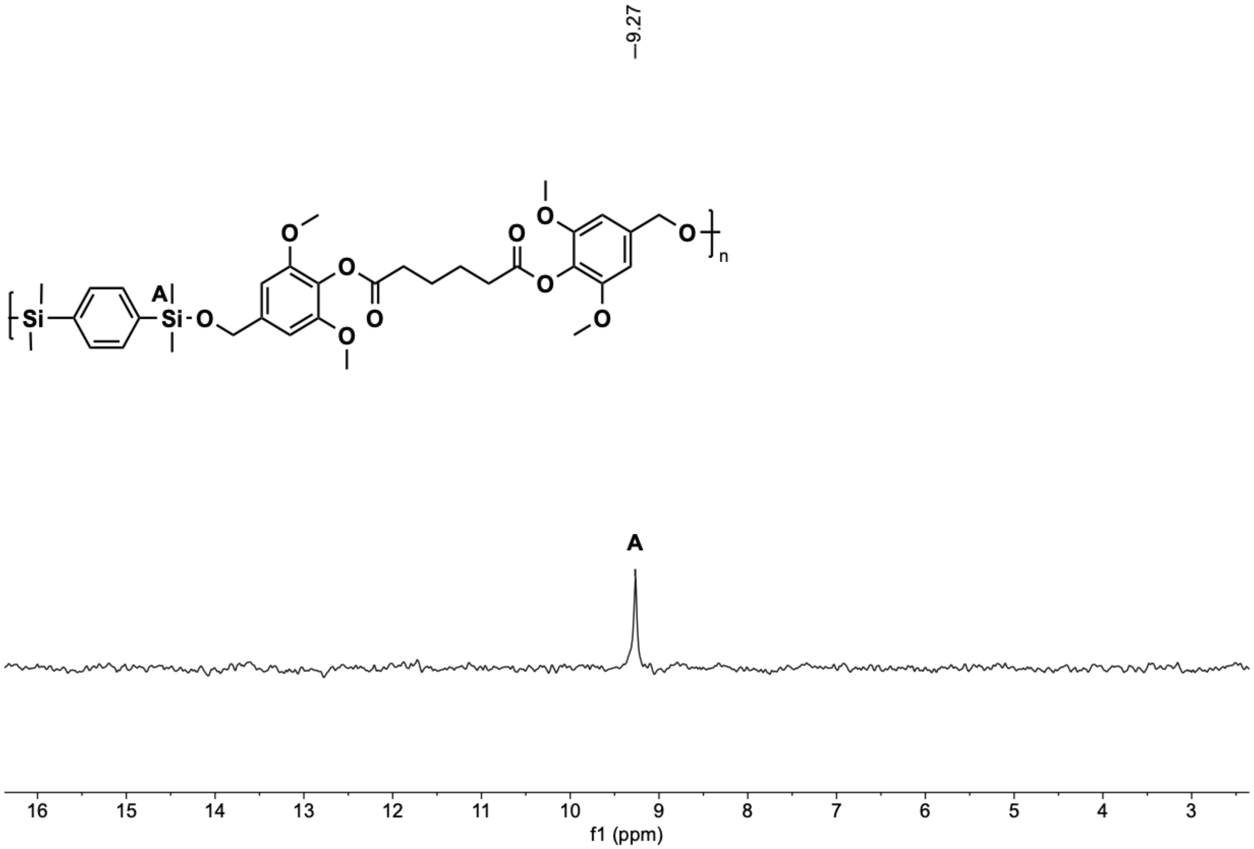

Figure S45 ^{29}Si NMR spectrum (99 MHz, $CDCl_3$) of **poly(AA-Sy-*co*-BDMSB)**.

Poly(AA-Sy-co-TMDS)

^{1}H NMR (400 MHz, Chloroform-*d*) δ 6.58 (s, 4H), 4.71 (s, 4H), 3.78 (s, 12H), 2.67 (p, *J* = 3.9 Hz, 4H), 1.93 (dd, *J* = 3.6, 6.3 Hz, 4H), 0.18 (s, 12H). **^{13}C{^{1}H} NMR (101 MHz, Chloroform-*d*)** δ δ 171.38, 152.20, 139.23, 127.64, 102.81, 64.28, 56.15, 33.64, 24.55. **^{29}Si NMR (99 MHz, Chloroform-*d*)** δ -10.59.

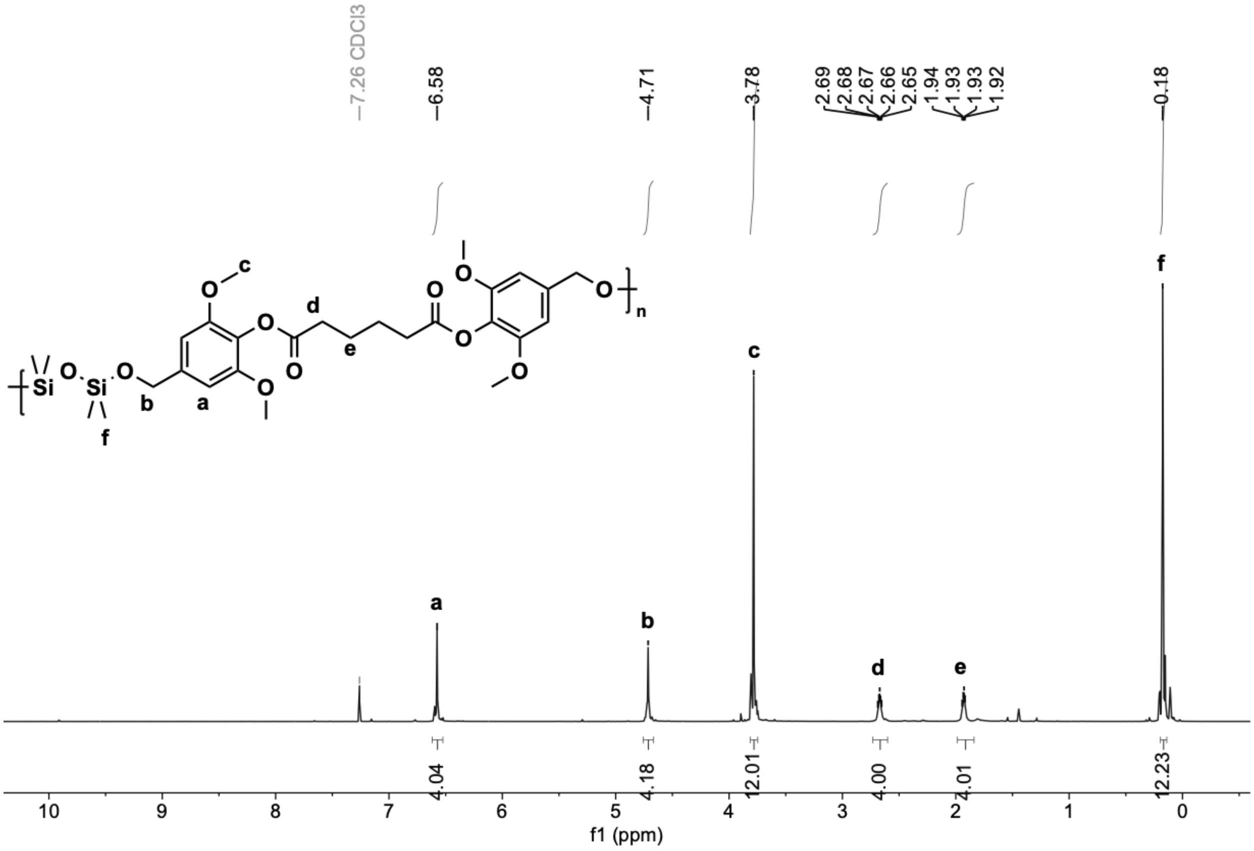

Figure S46 ^{1}H NMR spectrum (400 MHz, $CDCl_3$) of **poly(AA-Sy-*co*-TMDS)**.

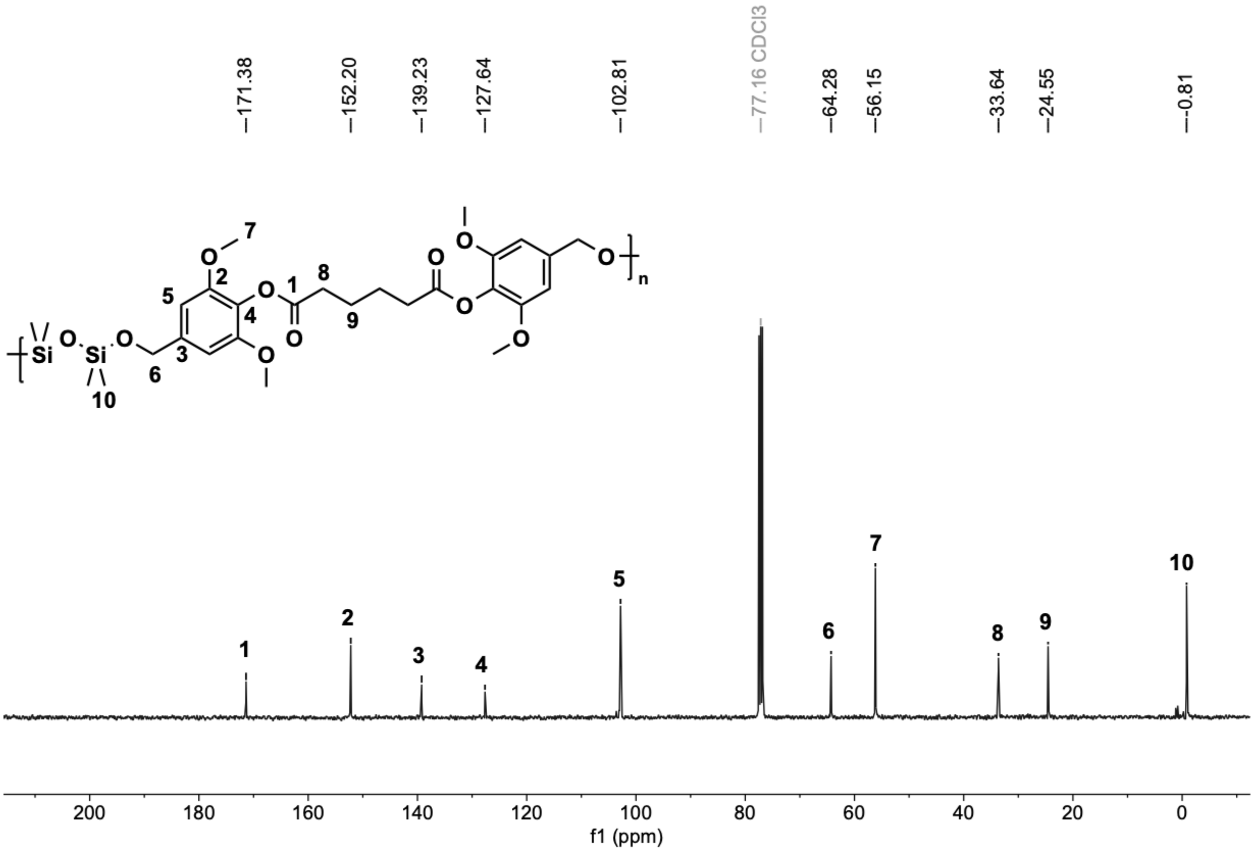

Figure S47 $^{13}C\{^{1}H\}$ NMR spectrum (101 MHz, $CDCl_3$) of **poly(AA-Sy-*co*-TMDS)**.

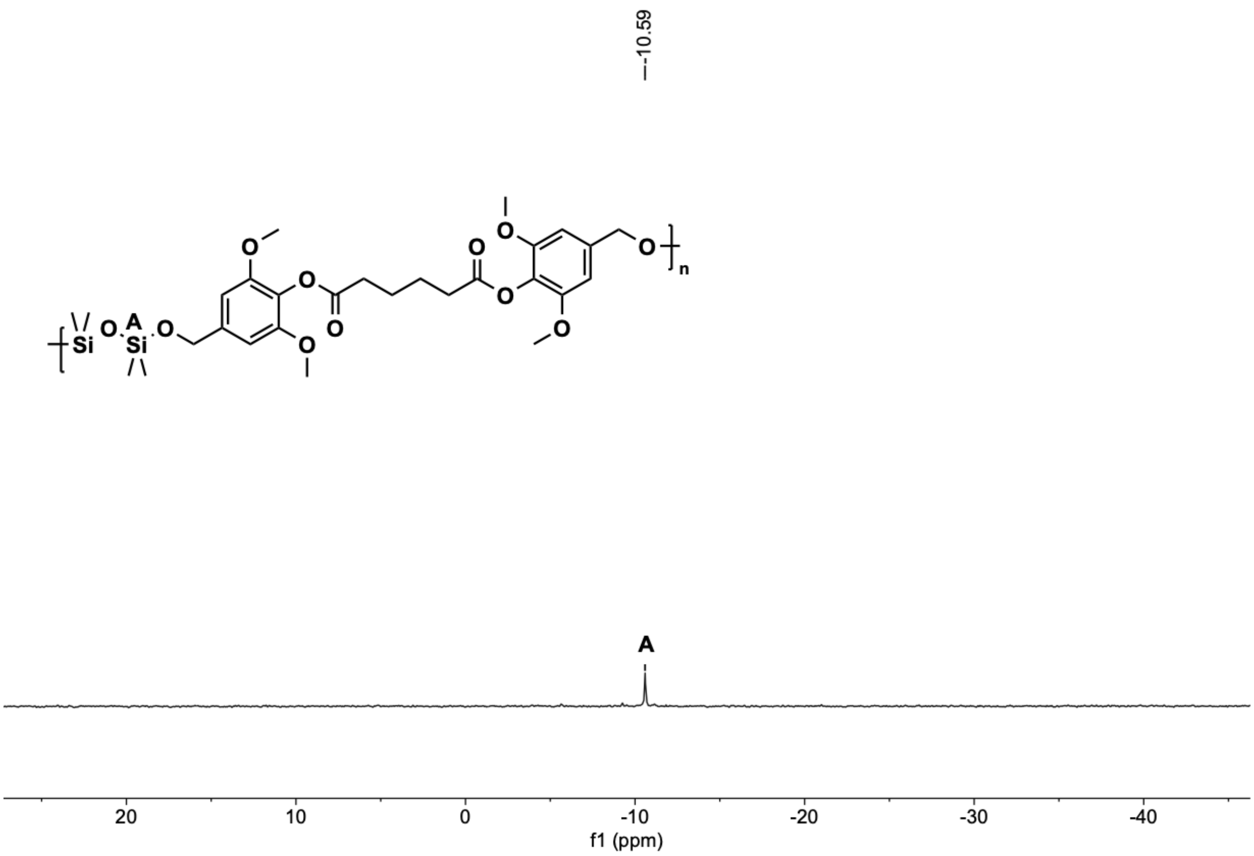

Figure S48 ^{29}Si NMR spectrum (99 MHz, $CDCl_3$) of **poly(AA-Sy-co-TMDS)**.

Poly(PA-Va-co-Ph)

^{1}H NMR (400 MHz, Chloroform-*d*) δ 7.73 – 7.68 (m, 4H), 7.46 – 7.36 (m, 6H), 6.98 – 6.85 (m, 6H), 4.81 (s, 4H), 3.73 (s, 6H), 2.60 (t, *J* = 7.4 Hz, 4H), 1.83 (t, *J* = 7.7 Hz, 4H), 1.58 (d, *J* = 7.4 Hz, 2H). **^{13}C{^{1}H} NMR (101 MHz, Chloroform-*d*)** δ 171.83, 151.09, 139.29, 138.93, 135.10, 132.26, 130.76, 128.17, 122.66, 118.76, 110.84, 64.88, 55.90, 33.98, 28.58, 24.80. **^{29}Si NMR (99 MHz, Chloroform-*d*)** δ -30.25.

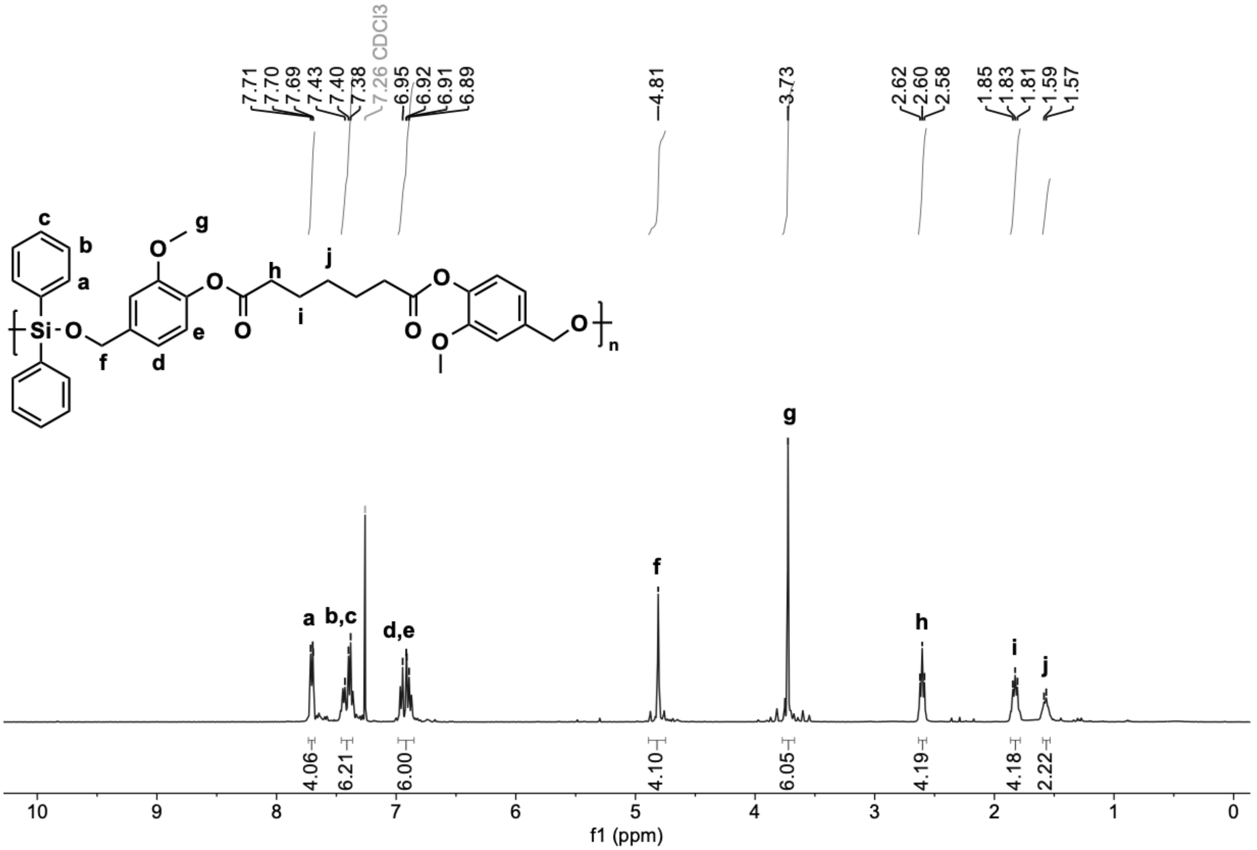

Figure S49 ^{1}H NMR spectrum (400 MHz, $CDCl_3$) of **poly(PA-Va-*co*-Ph)**.

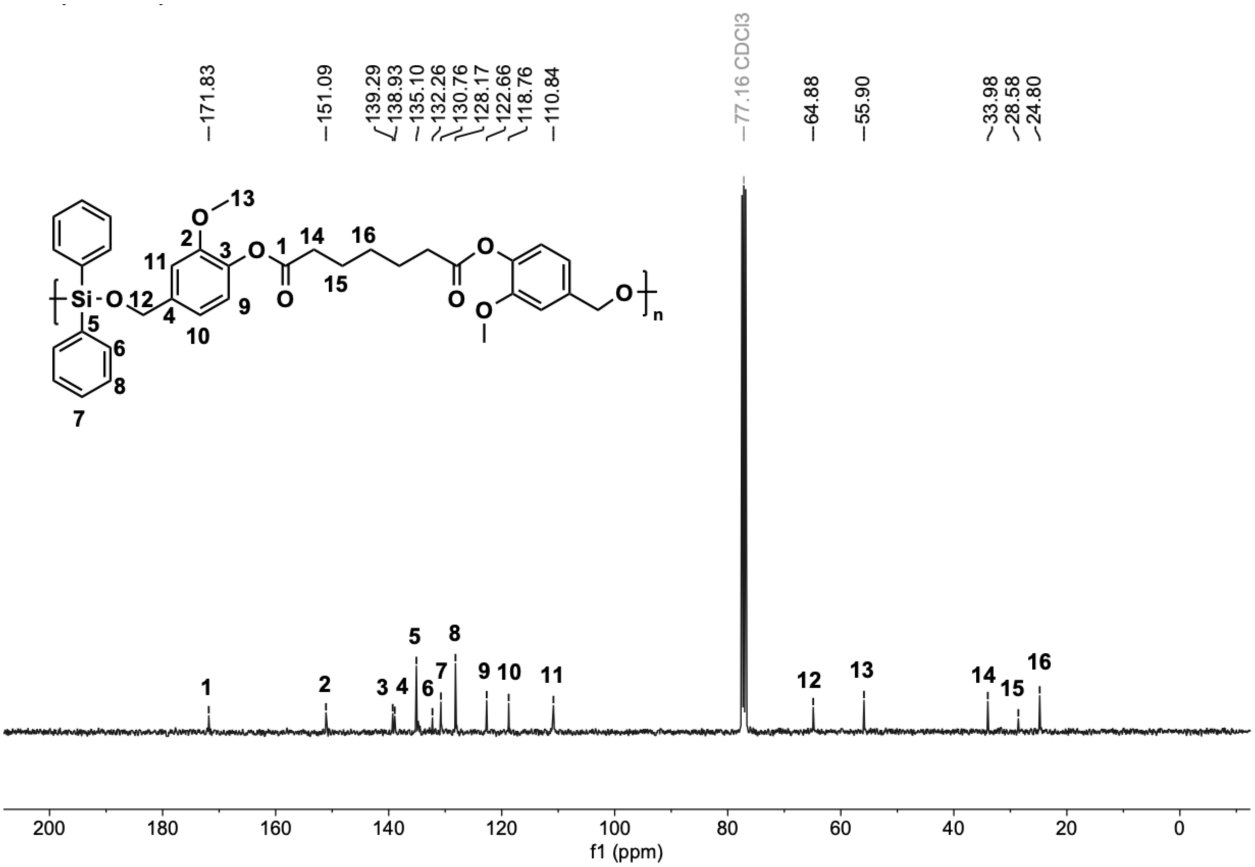

Figure S50 $^{13}C\{^{1}H\}$ NMR spectrum (101 MHz, $CDCl_3$) of **poly(PA-Va-*co*-Ph)**.

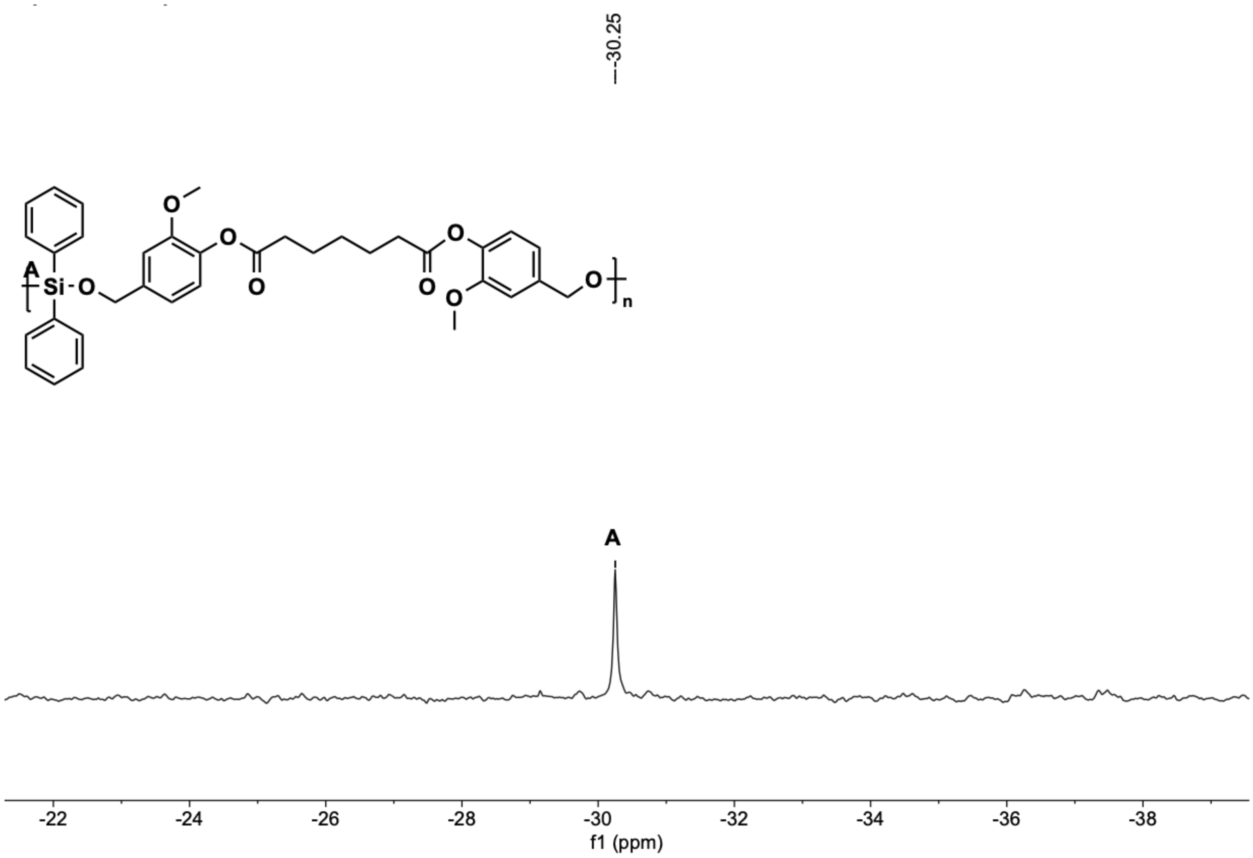

Figure S51 ^{29}Si NMR spectrum (99 MHz, $CDCl_3$) of **poly(PA-Va-*co*-Ph)**.

Poly(PA-Va-co-BDMSB)

^{1}H NMR (400 MHz, Chloroform-*d*) δ 7.61 (s, 4H), 6.97 – 6.90 (m, 4H), 6.84 (dd, *J* = 1.9, 8.2 Hz, 2H), 4.68 (s, 4H), 3.77 (s, 6H), 2.60 (t, *J* = 7.4 Hz, 5H), 1.82 (t, *J* = 7.7 Hz, 4H), 1.56 (d, *J* = 10.8 Hz, 4H), 0.42 (s, 12H). **^{13}C{^{1}H} NMR (101 MHz, Chloroform-*d*)** δ 171.84, 151.10, 139.74, 139.27, 138.86, 133.07, 122.59, 118.65, 110.79, 64.88, 55.91, 33.98, 28.58, 24.80, -1.59. **^{29}Si NMR (99 MHz, Chloroform-*d*)** δ 9.19.

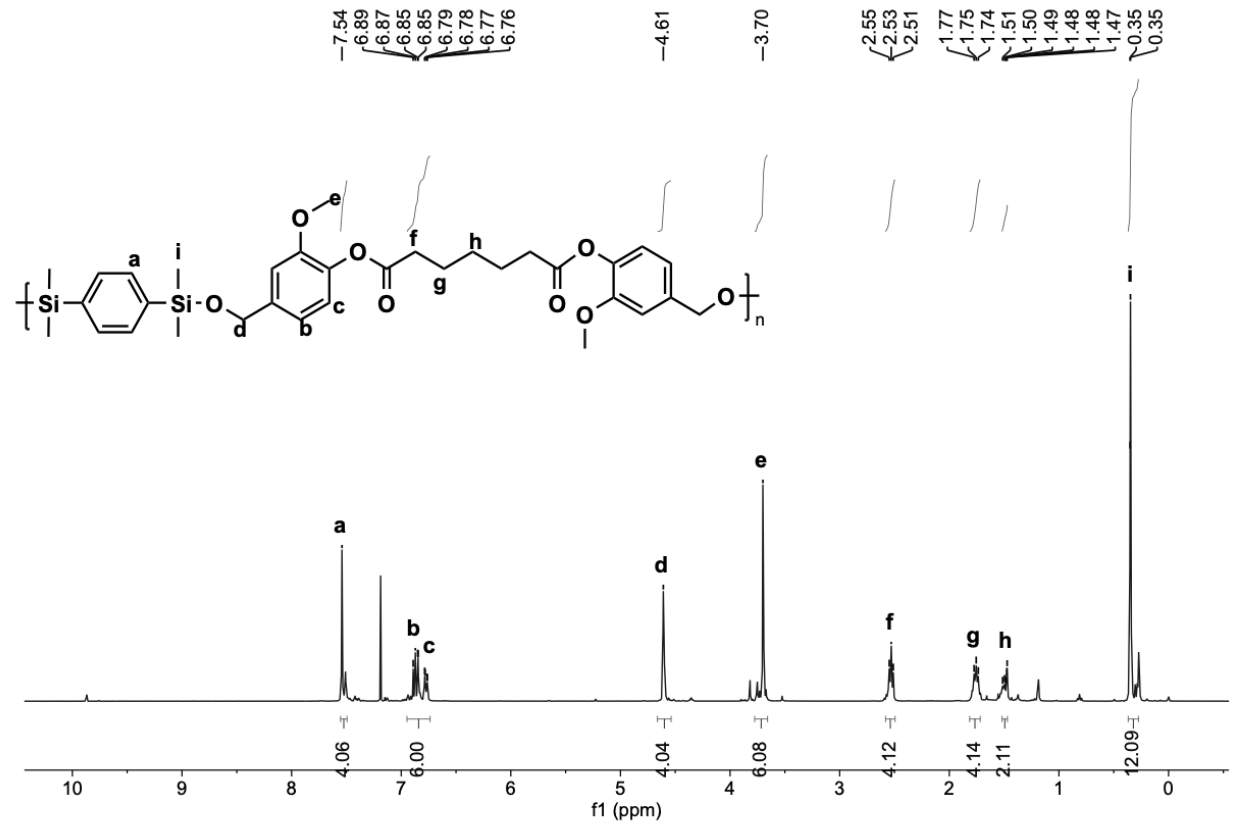

Figure S52 ^{1}H NMR spectrum (400 MHz, $CDCl_3$) of **poly(PA-Va-*co*-BDMSB).**

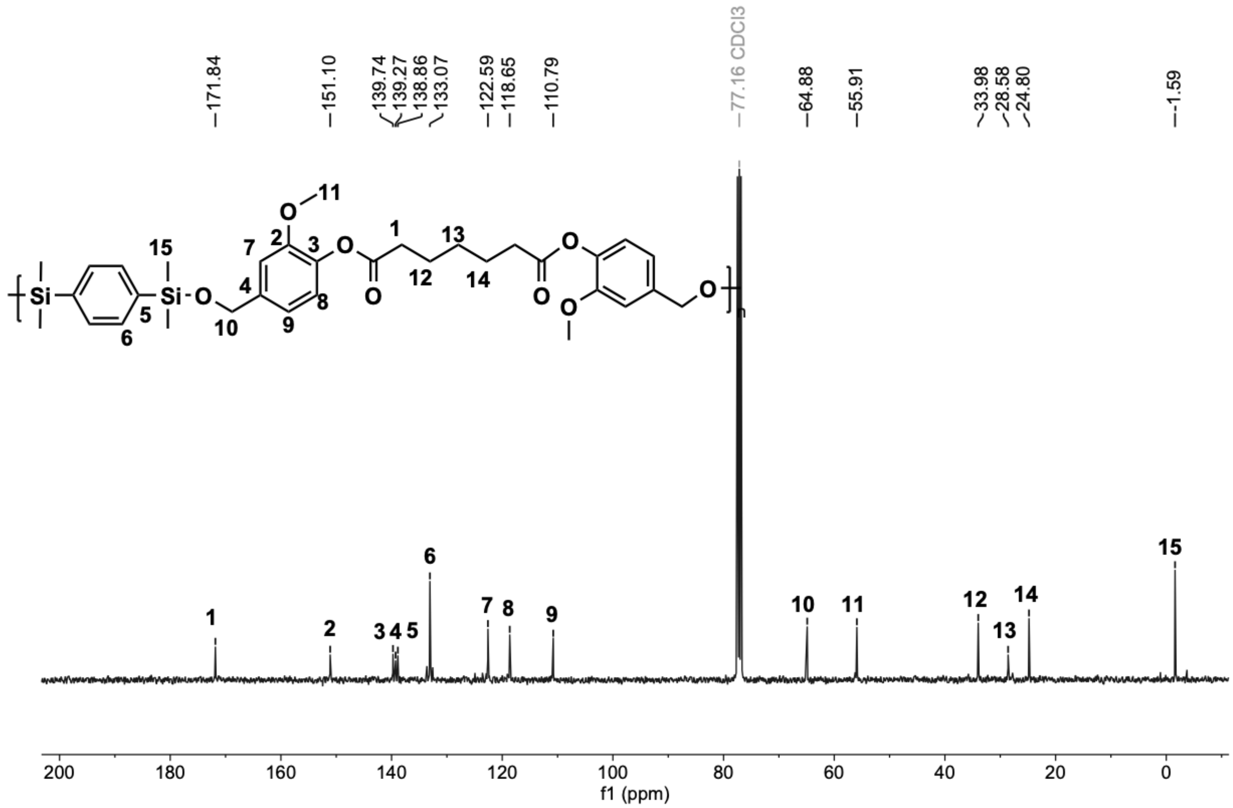

Figure S53 $^{13}C\{^1H\}$ NMR spectrum (101 MHz, $CDCl_3$) of **poly(PA-Va-*co*-BDMSB).**

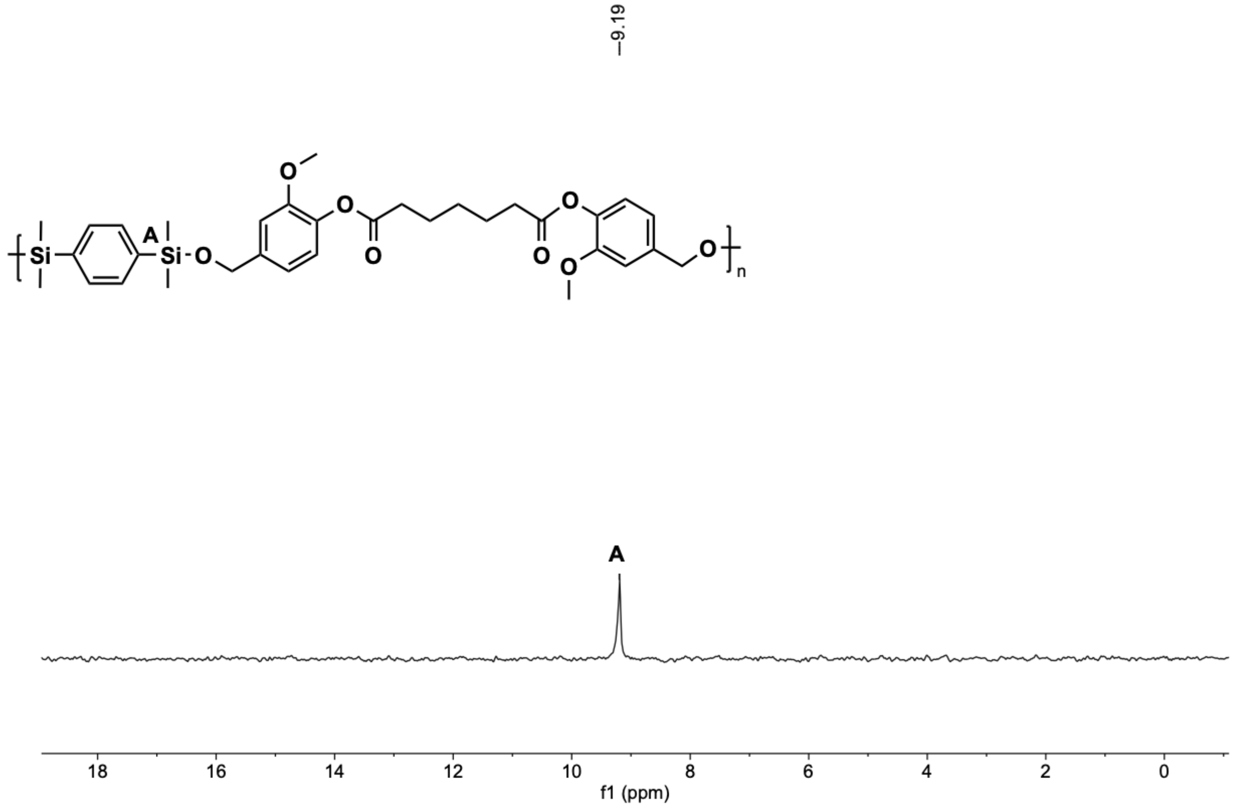

Figure S54 ^{29}Si NMR spectrum (99 MHz, $CDCl_3$) of **poly(PA-Va-*co*-BDMSB).**

Poly(PA-Va-co-TMDS)

^{1}H NMR (400 MHz, Chloroform-*d*) δ 6.96 (d, *J* = 8.1 Hz, 4H), 6.89 – 6.84 (m, 2H), 4.73 (s, 4H), 3.80 (s, 6H), 2.60 (t, *J* = 7.4 Hz, 4H), 1.87 – 1.79 (m, 4H), 1.56 (d, *J* = 10.0 Hz, 2H), 0.16 (s, 12H). **^{13}C{^{1}H} NMR (101 MHz, Chloroform-*d*)** δ 171.82, 151.13, 139.70, 138.86, 122.60, 118.54, 110.65, 63.98, 55.93, 33.97, 28.58, 24.79. **^{29}Si NMR (99 MHz, Chloroform-*d*)** δ -10.71.

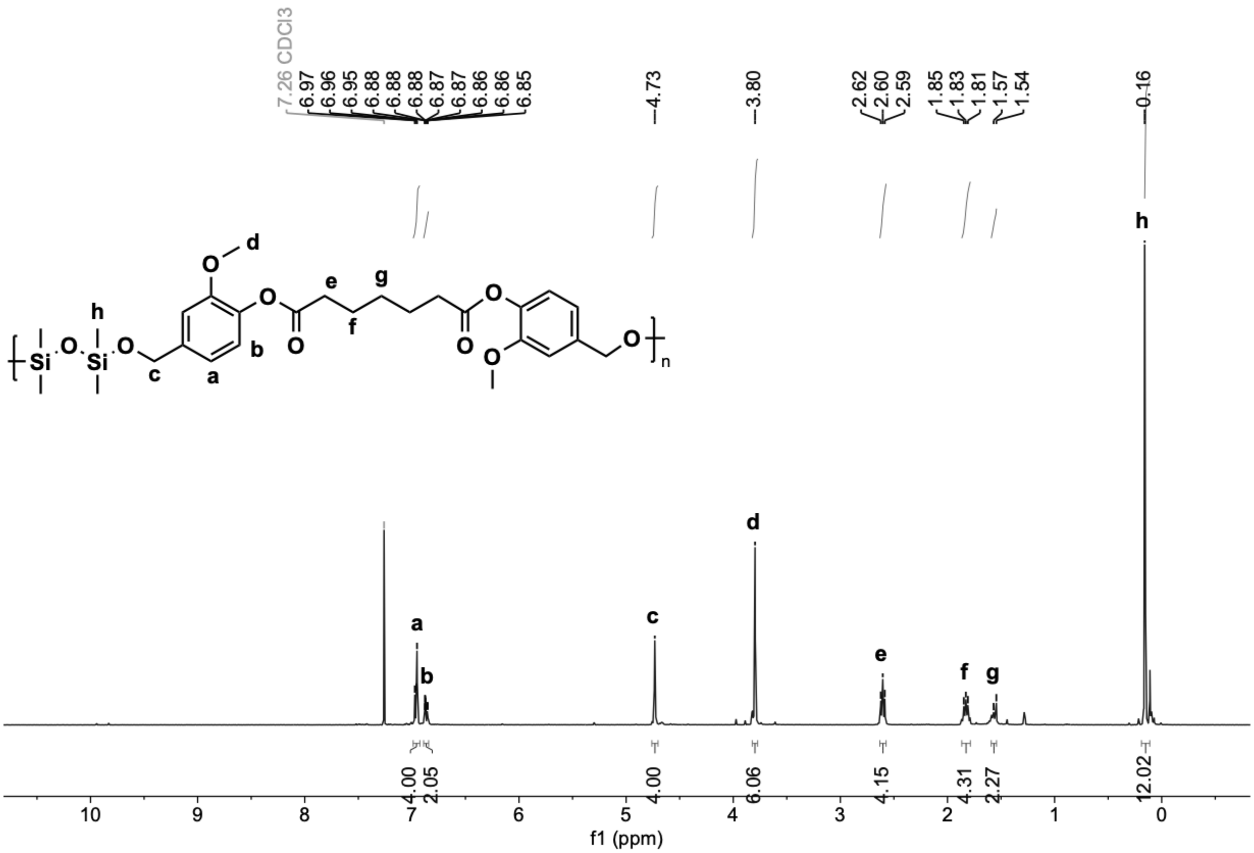

Figure 55 ^{1}H NMR spectrum (400 MHz, $CDCl_3$) of **poly(PA-Va-*co*-TMDS).**

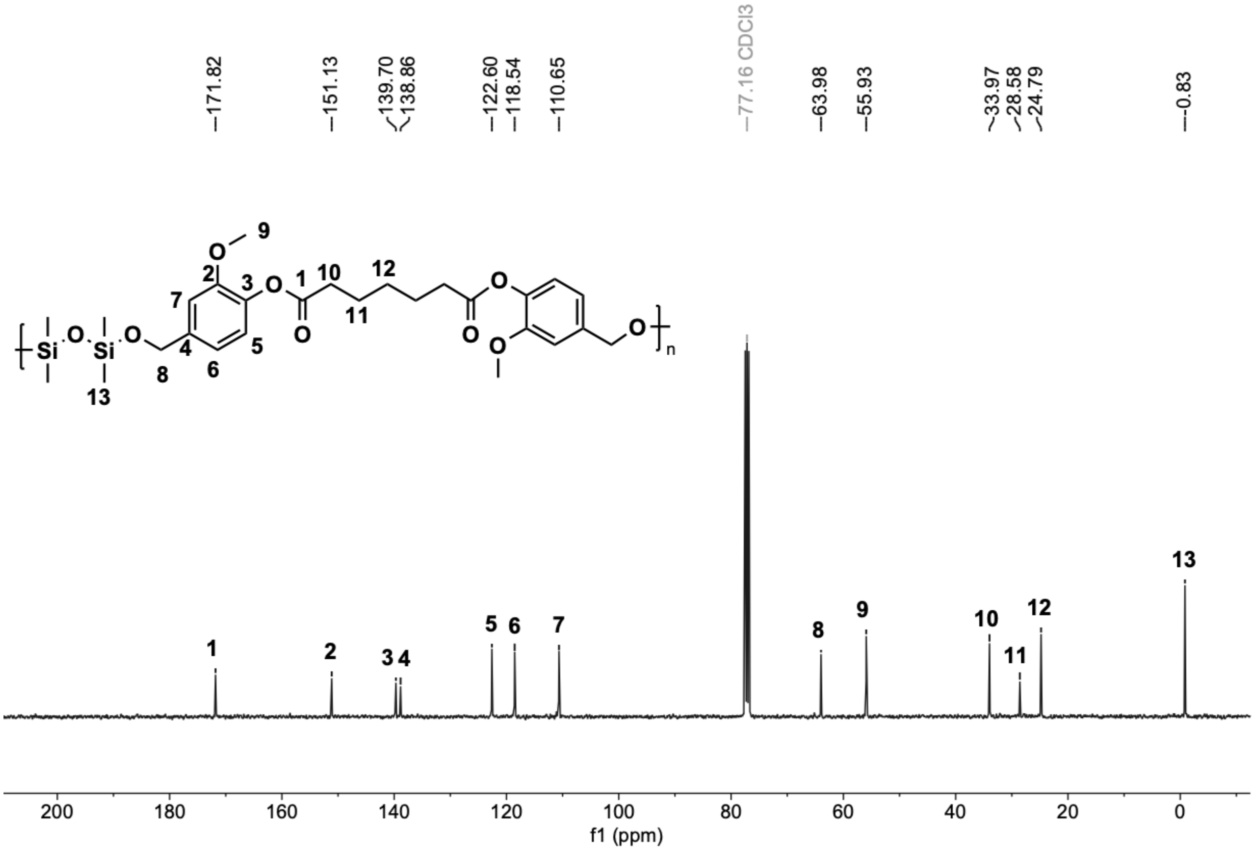

Figure S56 $^{13}C\{^{1}H\}$ NMR spectrum (101 MHz, $CDCl_3$) of **poly(PA-Va-*co*-TMDS).**

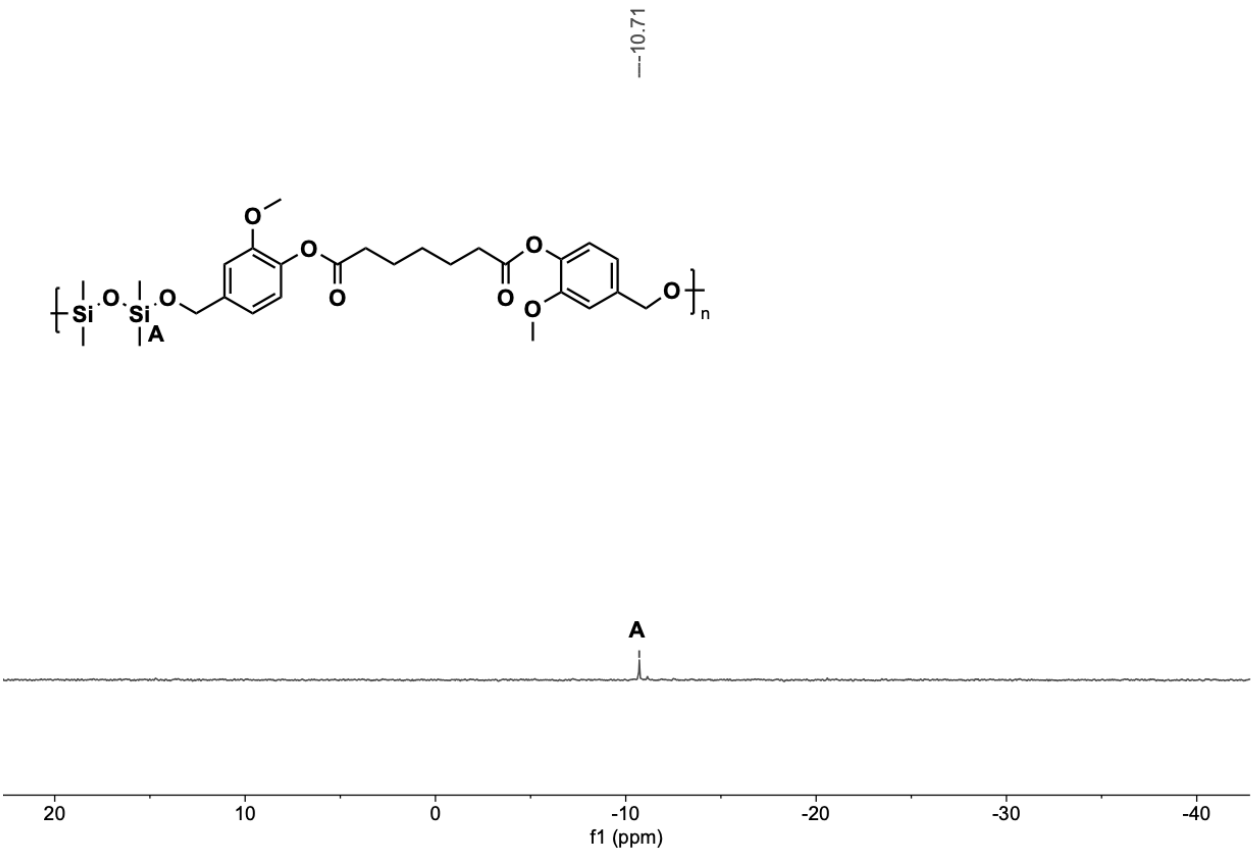

Figure S57 ^{29}Si NMR spectrum (99 MHz, $CDCl_3$) of **poly(PA-Va-*co*-TMDS).**

Poly(PA-Sy-co-Ph)

^{1}H NMR (400 MHz, Chloroform-*d*) δ 7.76 – 7.68 (m, 4H), 7.47 – 7.37 (m, 6H), 6.57 (s, 4H), 4.80 (s, 4H), 3.73 (s, 12H), 2.64 (t, *J* = 7.4 Hz, 4H), 1.84 (t, *J* = 7.7 Hz, 4H), 1.60 (dd, *J* = 3.0, 7.3 Hz, 2H). **^{13}C{^{1}H} NMR (101 MHz, Chloroform-*d*)** δ 171.54, 152.18, 138.76, 135.07, 132.30, 130.77, 128.17, 127.71, 103.14, 65.27, 56.15, 33.85, 28.49, 24.89. **^{29}Si NMR (99 MHz, Chloroform-*d*)** δ -30.25.

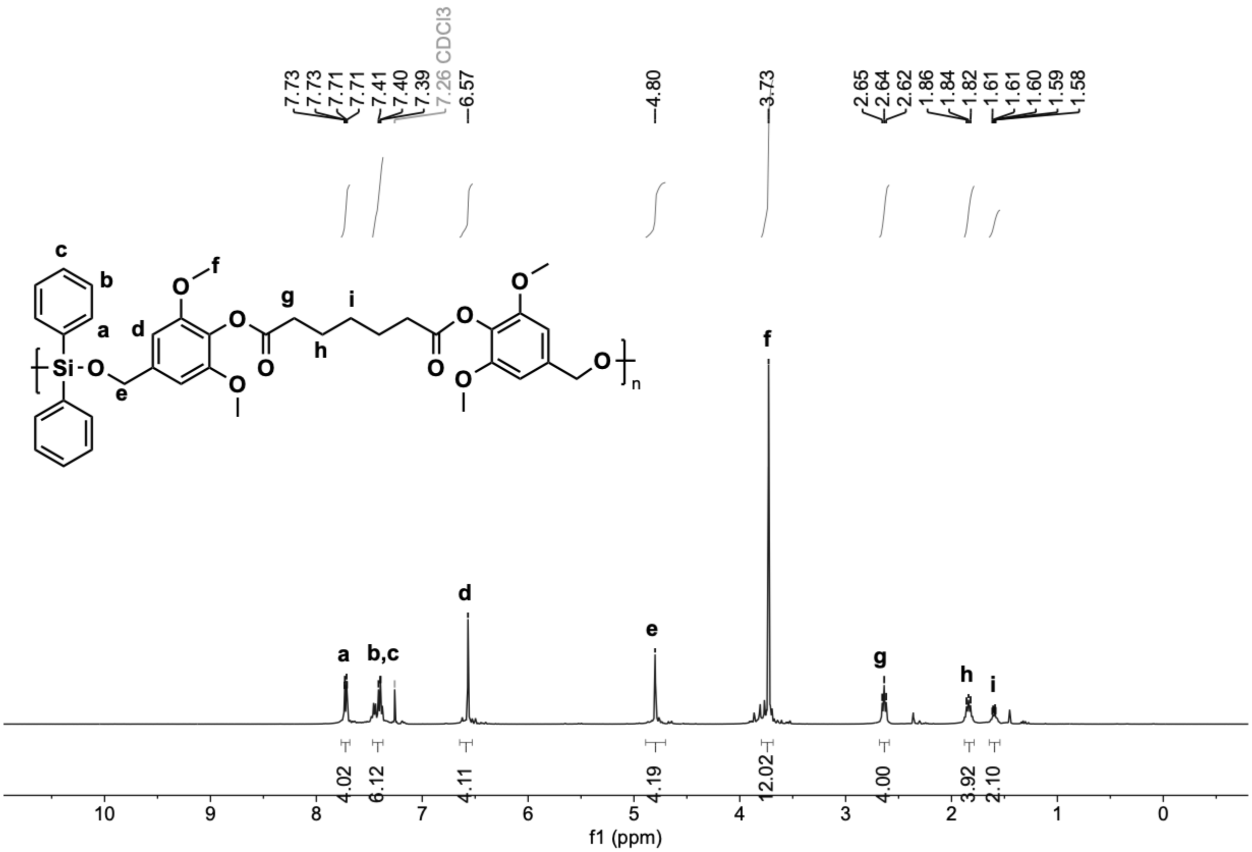

Figure S58 ^{1}H NMR spectrum (400 MHz, $CDCl_3$) of **poly(PA-Sy-*co*-Ph)**.

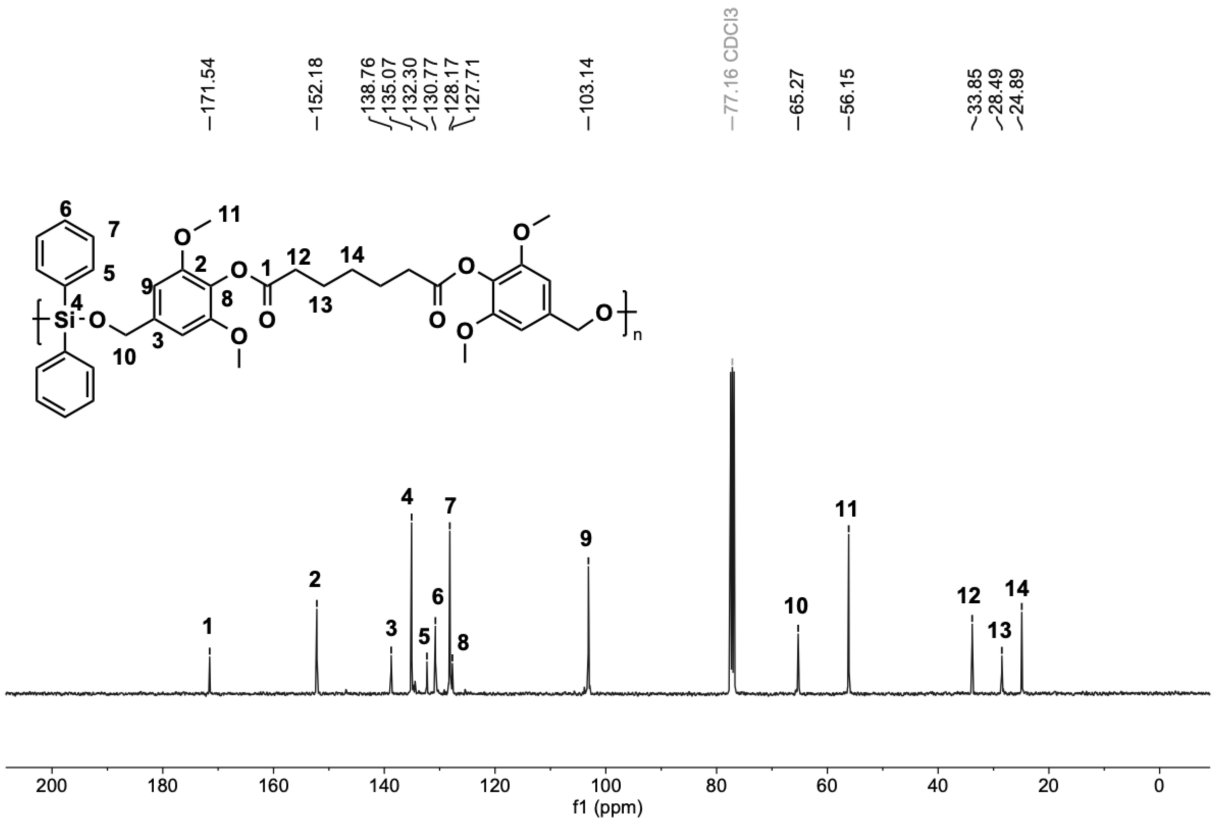

Figure S59 $^{13}C\{^{1}H\}$ NMR spectrum (101 MHz, $CDCl_3$) of **poly(PA-Sy-*co*-Ph)**.

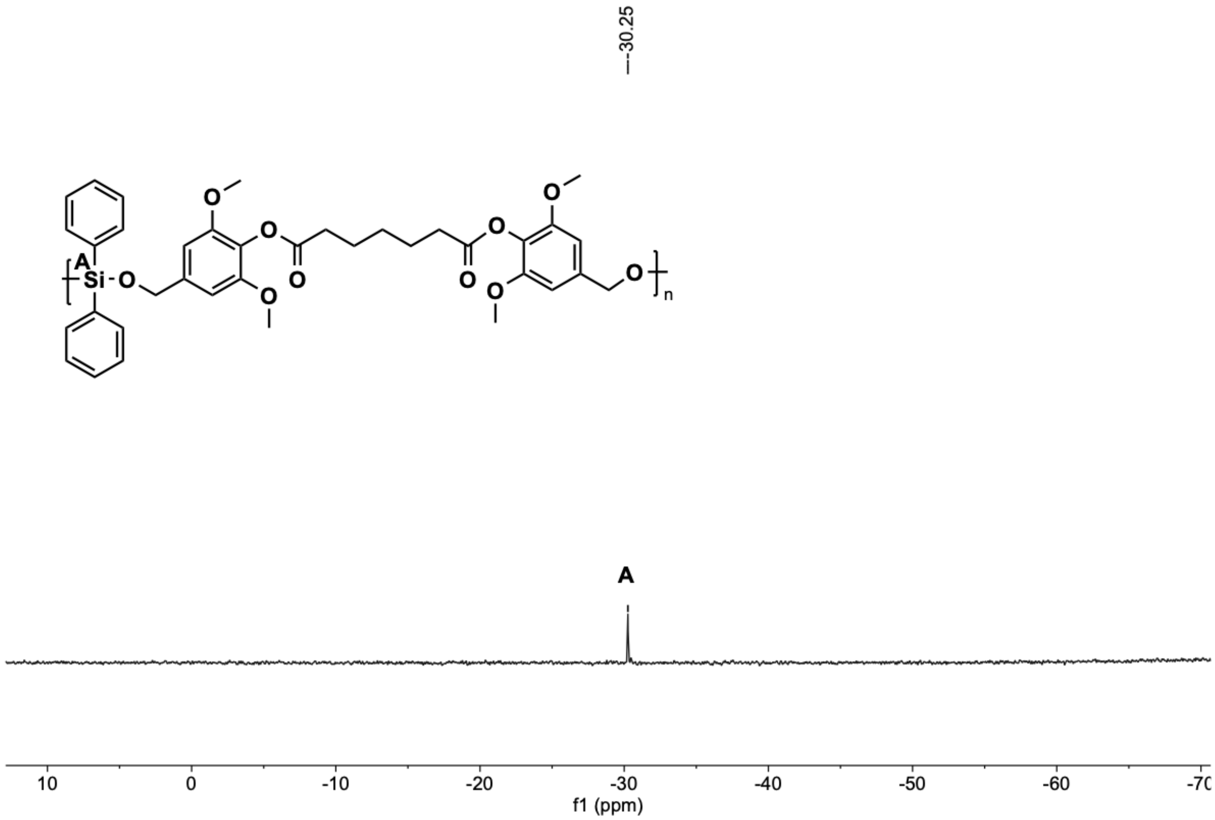

Figure S60 ^{29}Si NMR spectrum (99 MHz, $CDCl_3$) of **poly(PA-Sy-*co*-Ph)**.

Poly(PA-Sy-co-BDMSB)

^{1}H NMR (400 MHz, Chloroform-*d*) δ 7.61 (s, 4H), 6.98 – 6.92 (m, 4H), 6.84 (s, 2H), 4.68 (s, 4H), 3.78 (s, 6H), 2.75 (t, *J* = 7.2 Hz, 4H), 2.20 (t, *J* = 7.2 Hz, 2H), 0.42 (s, 12H). **^{13}C{^{1}H} NMR (101 MHz, Chloroform-*d*)** δ 171.32, 151.04, 139.84, 139.26, 138.81, 133.07, 122.54, 118.64, 110.77, 64.87, 55.88, 33.01, 20.53, -1.59. **^{29}Si NMR (99 MHz, Chloroform-*d*) δ 9.27.**

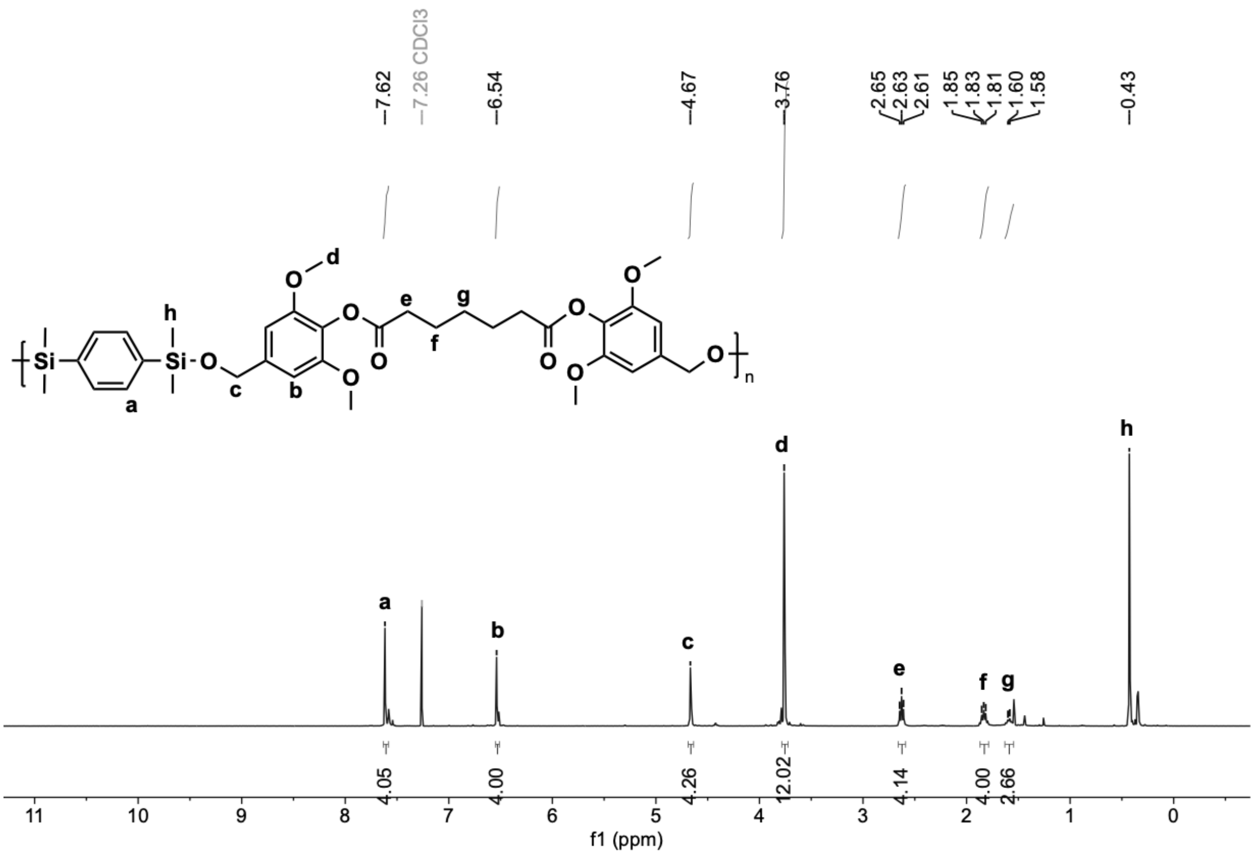

Figure S61 ^{1}H NMR spectrum (400 MHz, $CDCl_3$) of **poly(PA-Sy-*co*-BDMSB).**

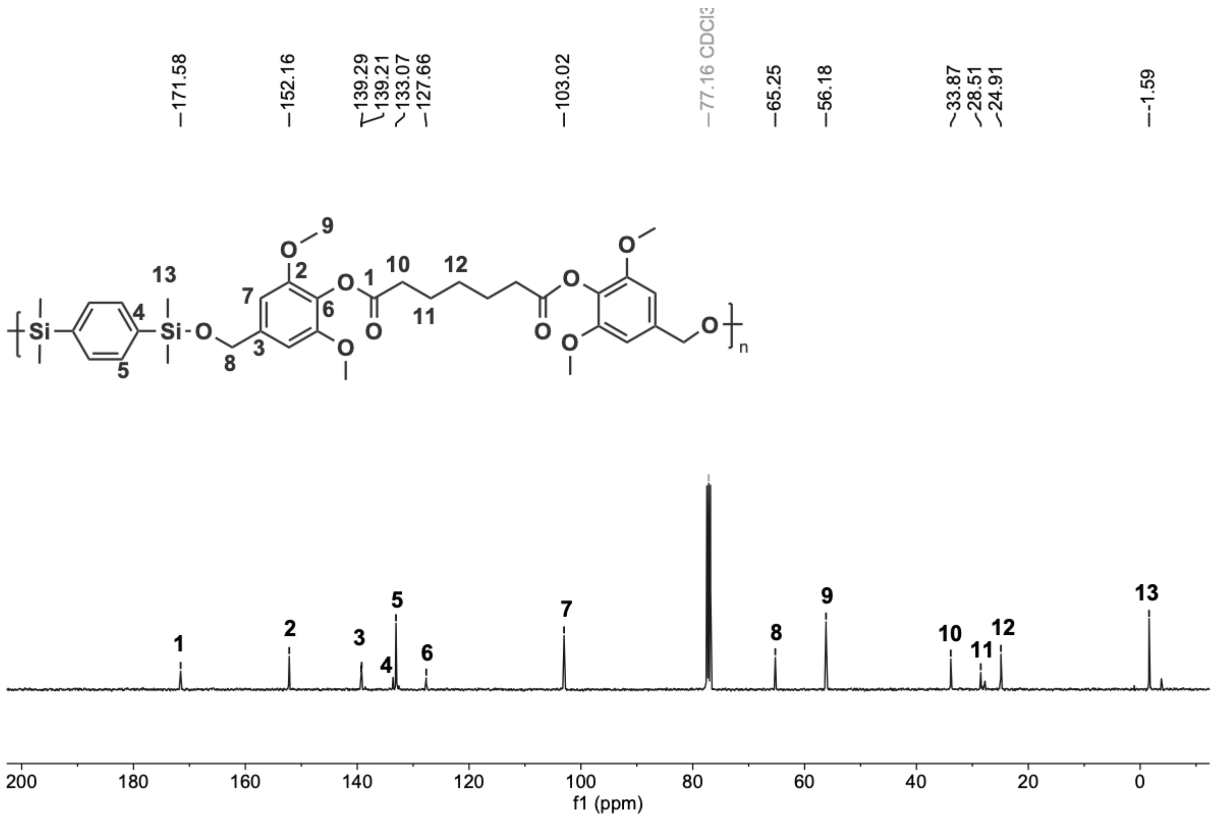

Figure S62 $^{13}C\{^{1}H\}$ NMR spectrum (101 MHz, $CDCl_3$) of **poly(PA-Sy-co-BDMSB).**

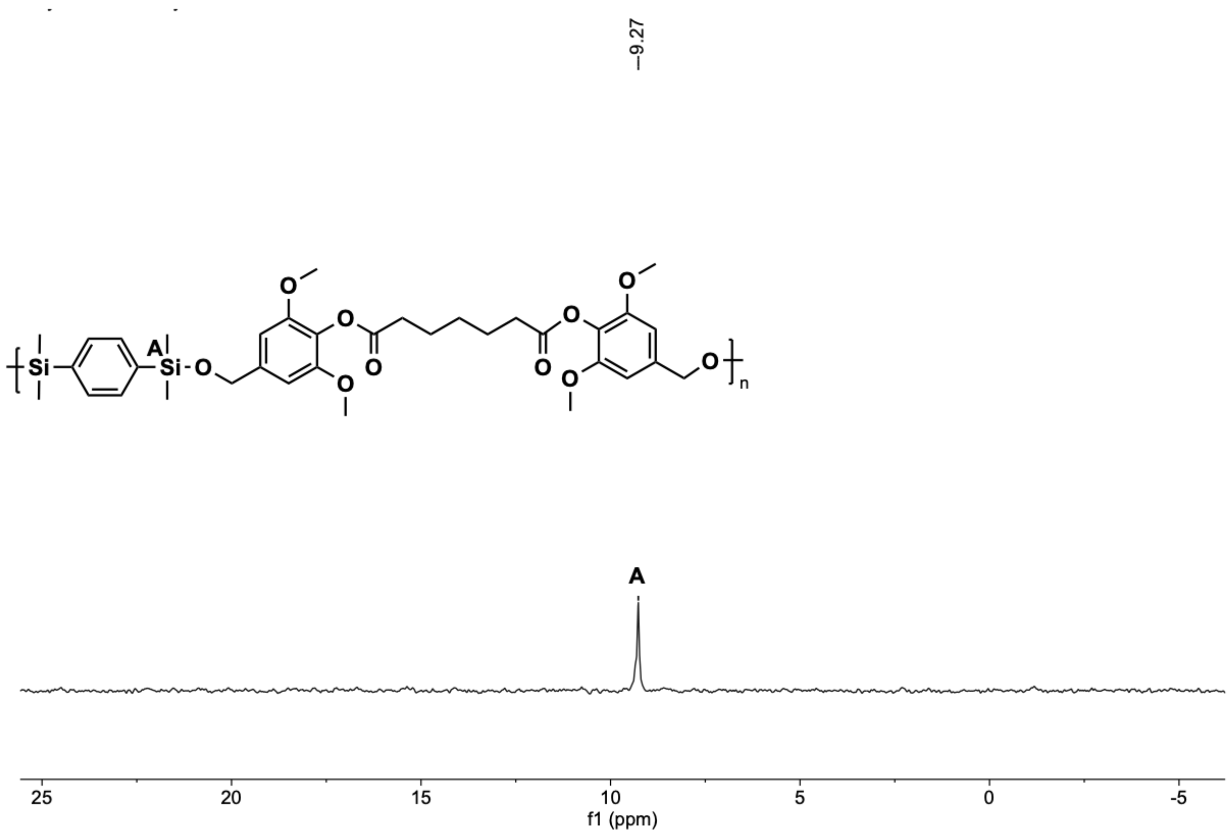

Figure S63 ^{29}Si NMR spectrum (99 MHz, $CDCl_3$) of **poly(PA-Sy-*co*-BDMSB).**

Poly(PA-Sy-co-TMDS)

^{1}H NMR (400 MHz, Chloroform-*d*) δ 6.96 (d, *J* = 8.1 Hz, 4H), 6.89 – 6.84 (m, 2H), 4.73 (s, 4H), 3.80 (s, 6H), 2.60 (t, *J* = 7.4 Hz, 4H), 1.87 – 1.79 (m, 4H), 1.56 (d, *J* = 10.0 Hz, 2H), 0.16 (s, 12H). **^{13}C{^{1}H} NMR (101 MHz, Chloroform-*d*)** δ 171.82, 151.13, 139.70, 138.86, 122.60, 118.54, 110.65, 63.98, 55.93, 33.97, 28.58, 24.79. **^{29}Si NMR (99 MHz, Chloroform-*d*)** δ -10.6.

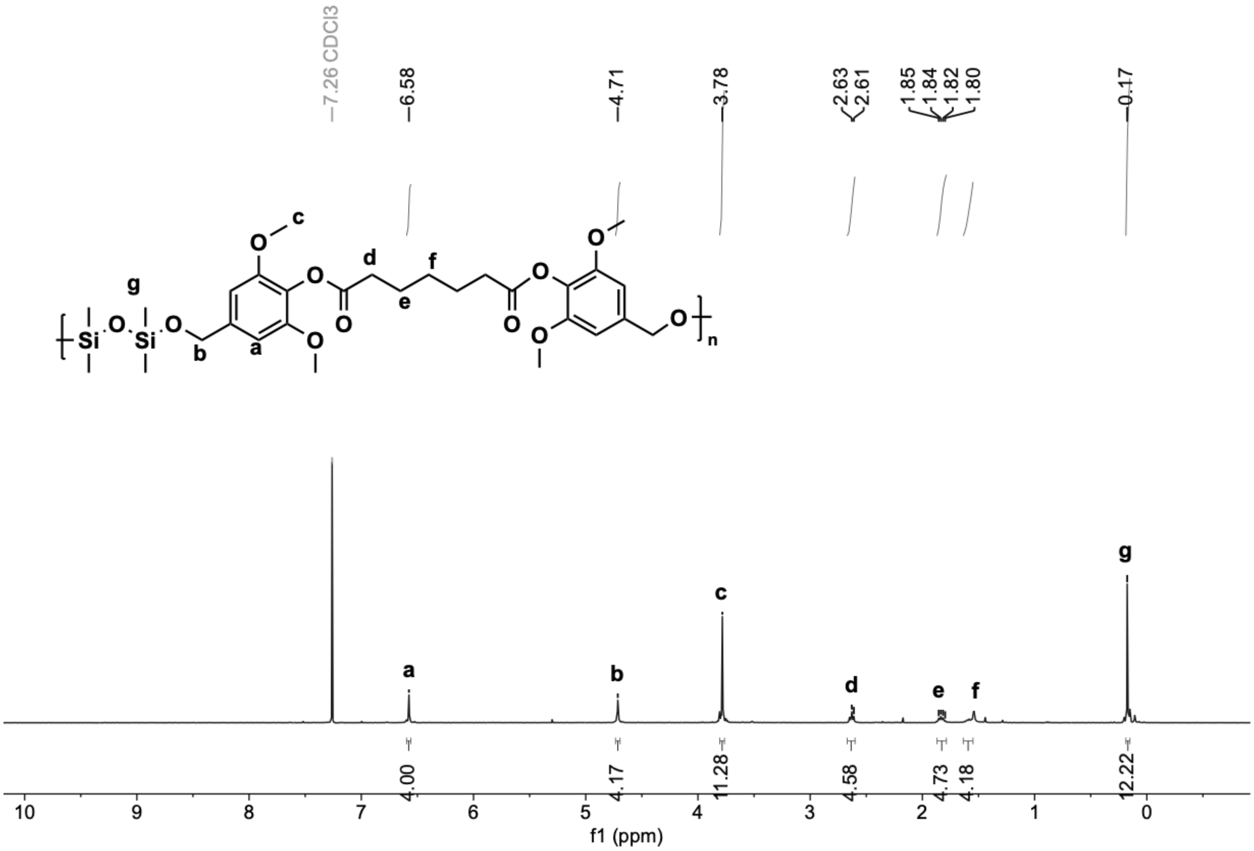

Figure S64 ^{1}H NMR spectrum (400 MHz, $CDCl_3$) of **poly(PA-Sy-*co*-TMDS).**

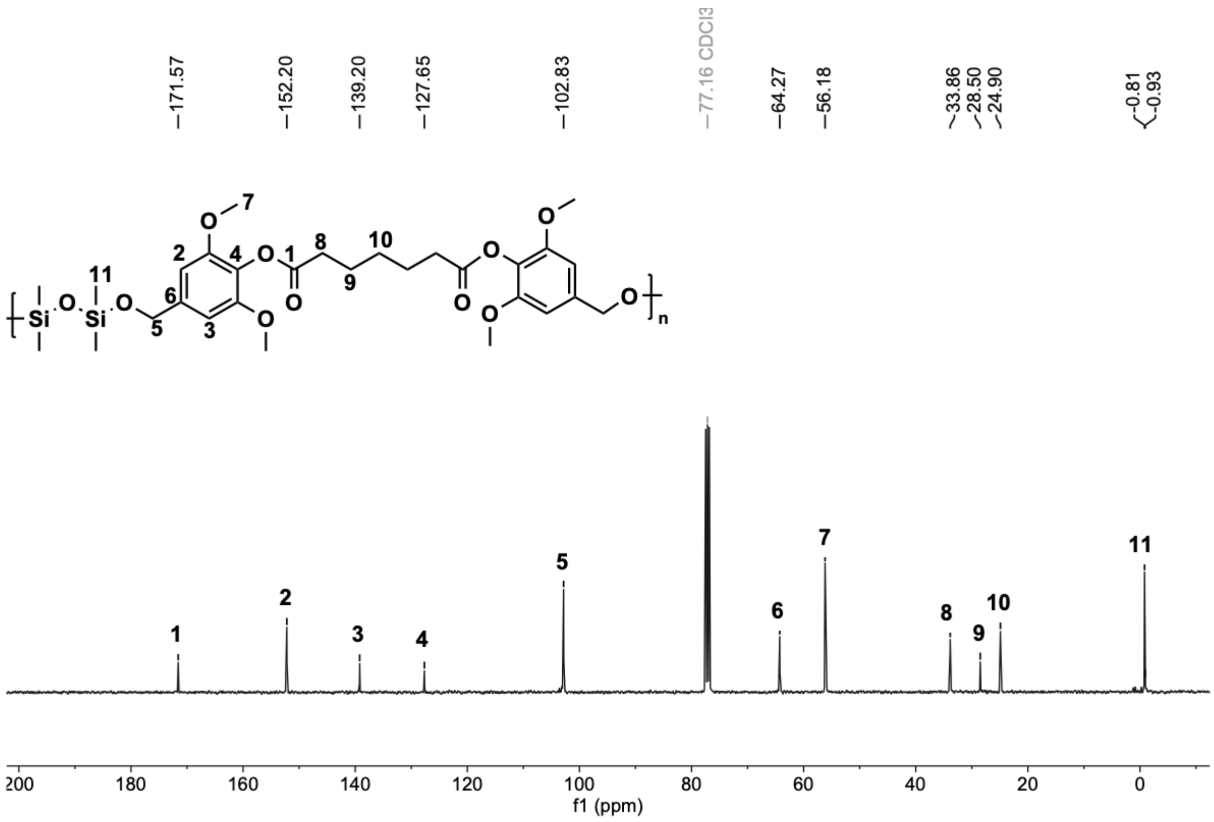

Figure S65 $^{13}C\{^{1}H\}$ NMR spectrum (101 MHz, $CDCl_3$) of **poly(PA-Sy-*co*-TMDS).**

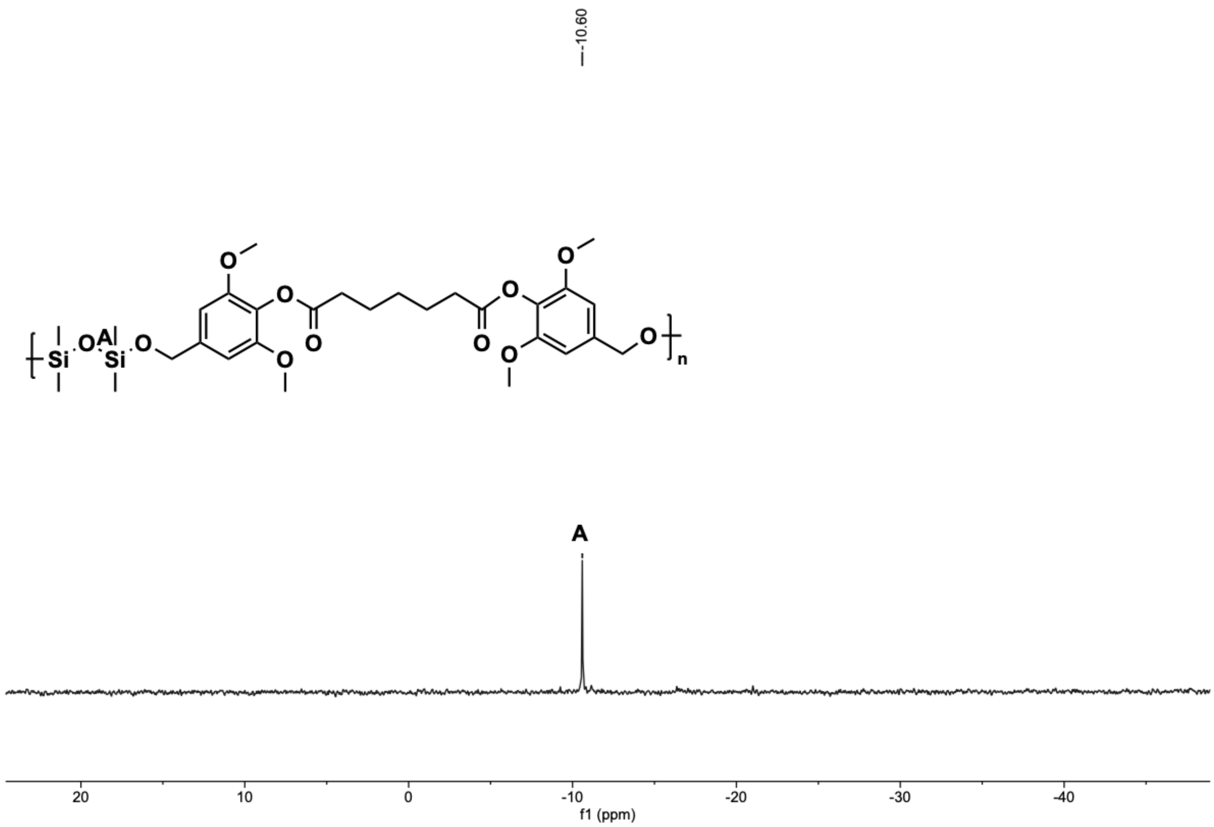

Figure S66 ^{29}Si NMR spectrum (99 MHz, $CDCl_3$) of **poly(PA-Sy-*co*-TMDS).**

Poly(GA-Sy-co-BDMSB-r-Ph)

^{1}H NMR (400 MHz, Chloroform-*d*) δ 7.74 – 7.70 (m, 2H), 7.62 (s, 4H), 7.50 – 7.31 (m, 6H), 6.57 (s, 2H), 6.55 (s, 4H), 4.80 (s, 2H), 4.67 (s, 4H), 3.77 (s, 12H), 3.73 (s, 6H), 2.79 (t, *J* = 7.2 Hz, 7H), 2.29 – 2.19 (m, 4H), 0.43 (s, 12H). **^{13}C{^{1}H} NMR (101 MHz, Chloroform-*d*)** δ 171.11, 152.14, 139.28, 135.08, 133.08, 130.78, 128.18, 127.69, 127.63, 103.13, 103.01, 65.24, 56.14, 32.93, 20.81, -1.59. **^{29}Si NMR (99 MHz, Chloroform-*d*)** δ 9.28, -30.27.

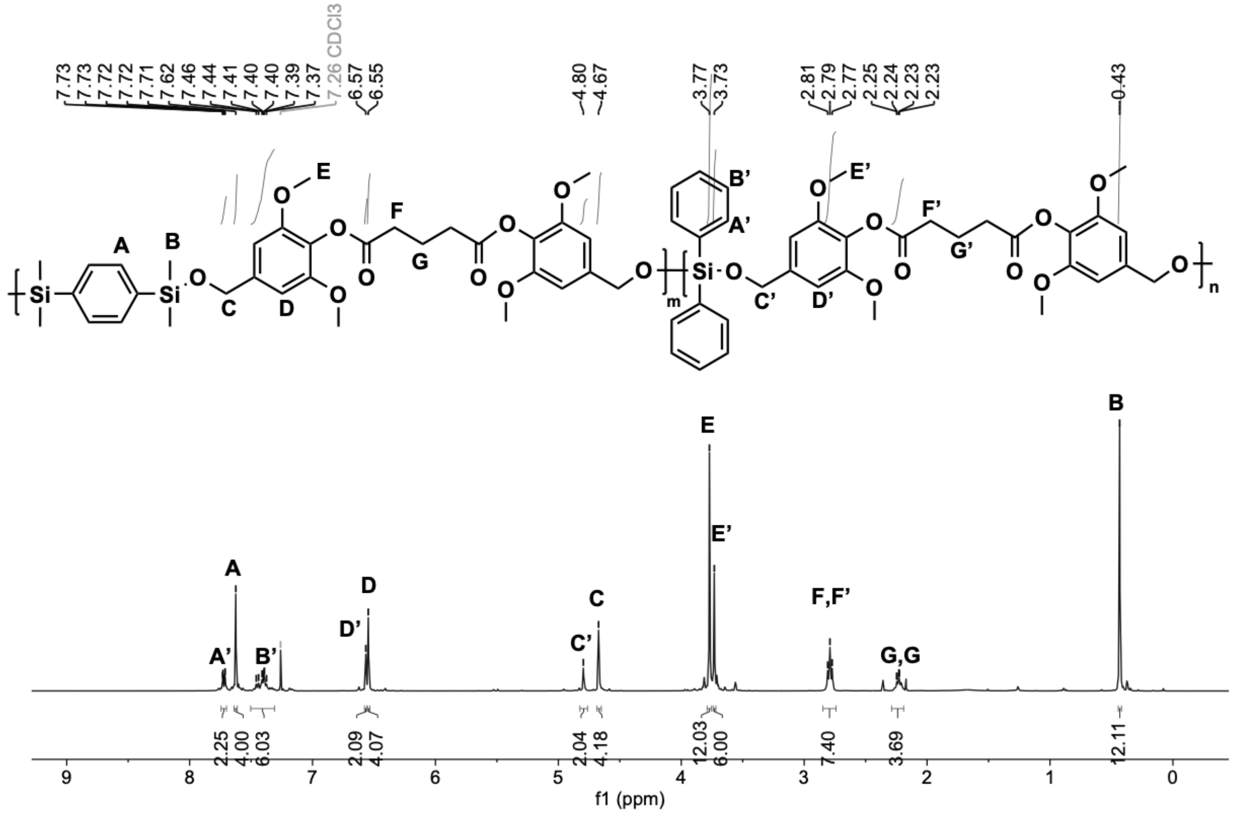

Figure S67 ^{1}H NMR spectrum (400 MHz, $CDCl_3$) of **poly(GA-Sy-*co*-BDMSB-*r*-Ph)**.

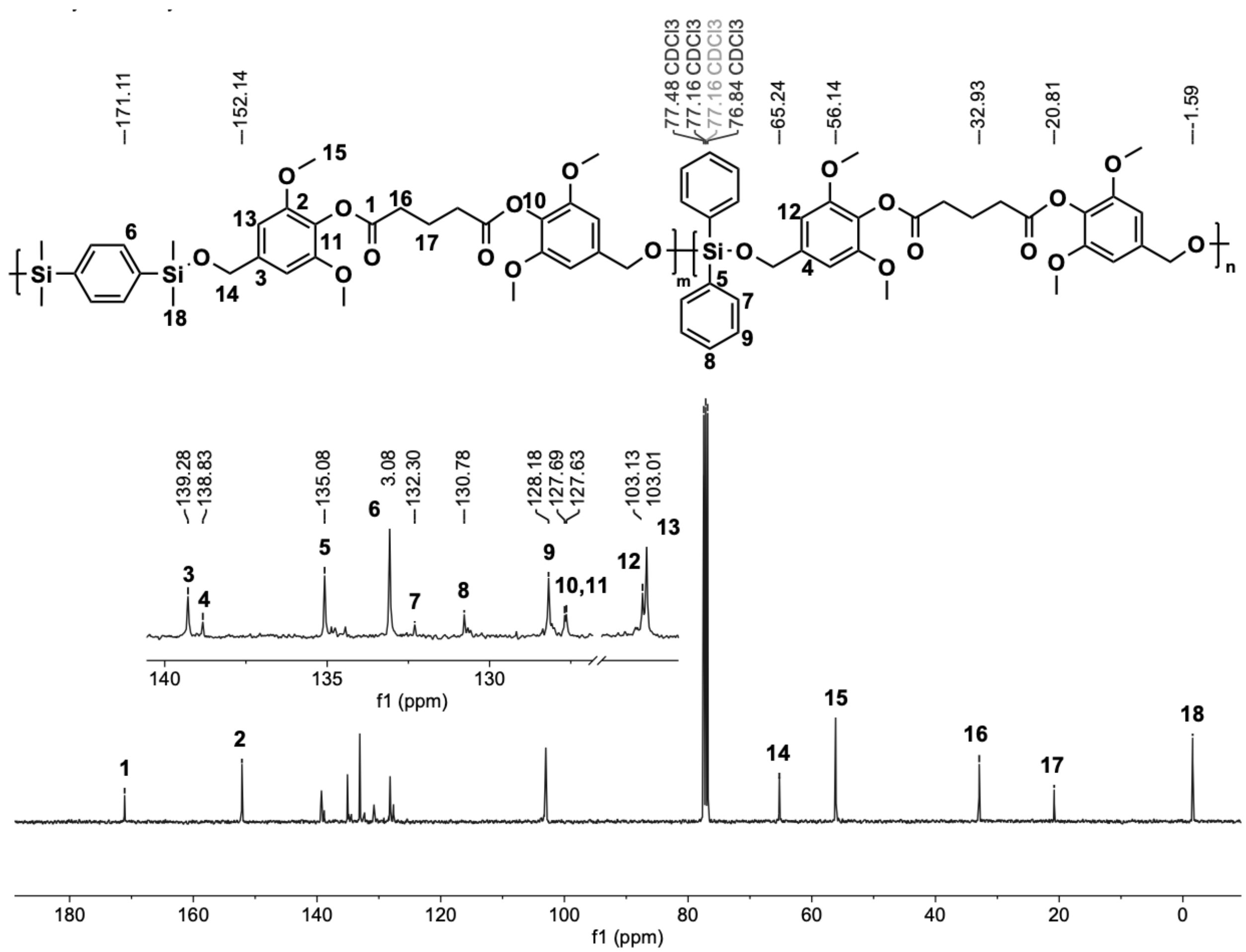

Figure S68 $^{13}C\{^{1}H\}$ NMR spectrum (101 MHz, $CDCl_3$) of **poly(GA-Sy-*co*-BDMSB-*r*-Ph)**.

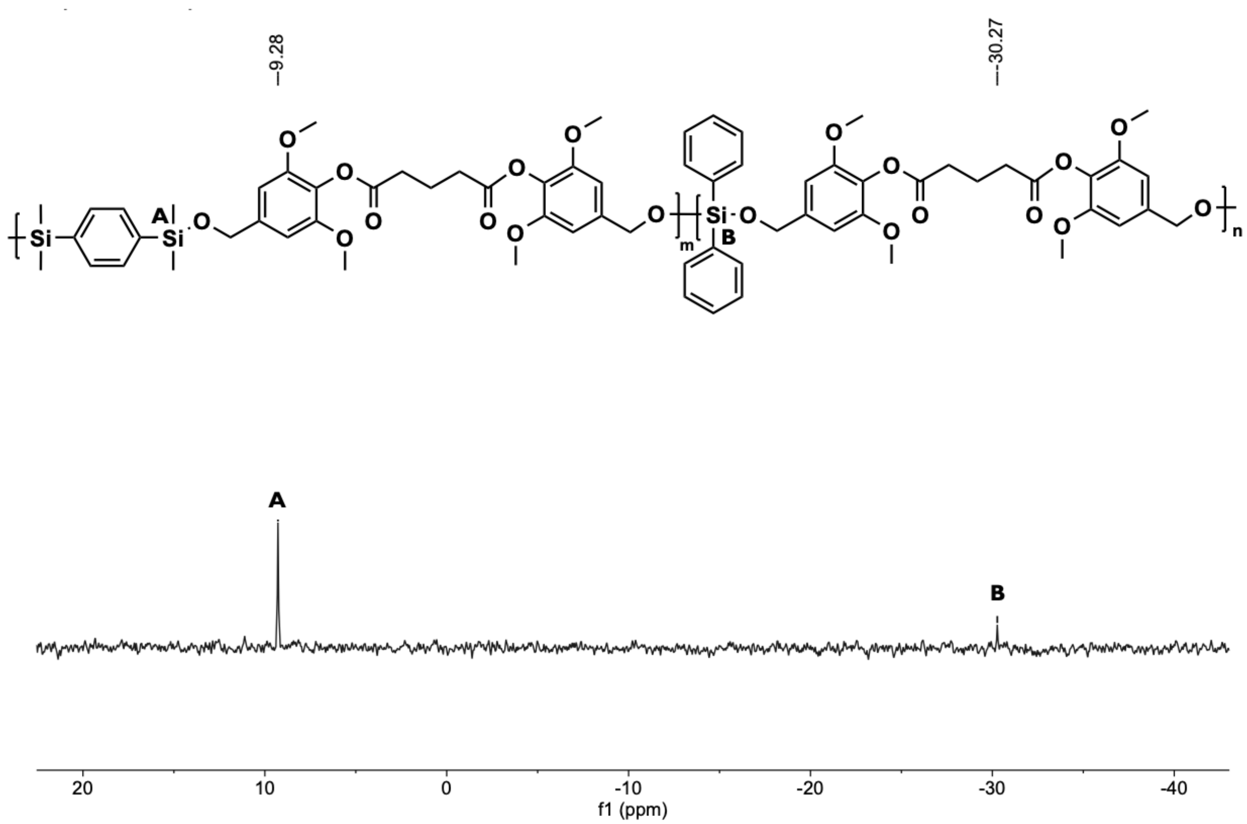

Figure S69 ^{29}Si NMR spectrum (99 MHz, $CDCl_3$) of **poly(GA-Sy-*co*-BDMSB-*r*-Ph)**.

Poly(GA-Sy-co-BDMSB-r-TMDS)

^{1}H NMR (400 MHz, Chloroform-*d*) δ 7.62 (s, 4H), 6.58 (s, 5H), 6.54 (s, 5H), 4.72 (s, 4H), 4.67 (s, 5H), 3.79 (s, 13H), 3.77 (s, 12H), 2.79 (t, *J* = 7.3 Hz, 10H), 2.24 (d, *J* = 7.3 Hz, 6H), 0.43 (s, 12H), 0.18 (s, 12H). **^{13}C{^{1}H} NMR (101 MHz, Chloroform-*d*)** δ 171.10, 152.20, 152.15, 139.28, 133.08, 132.55, 127.65, 103.02, 102.83, 65.24, 64.28, 56.16, 32.94, 20.82, -1.58. **^{29}Si NMR (99 MHz, Chloroform-*d*)** δ 9.29, -10.58.

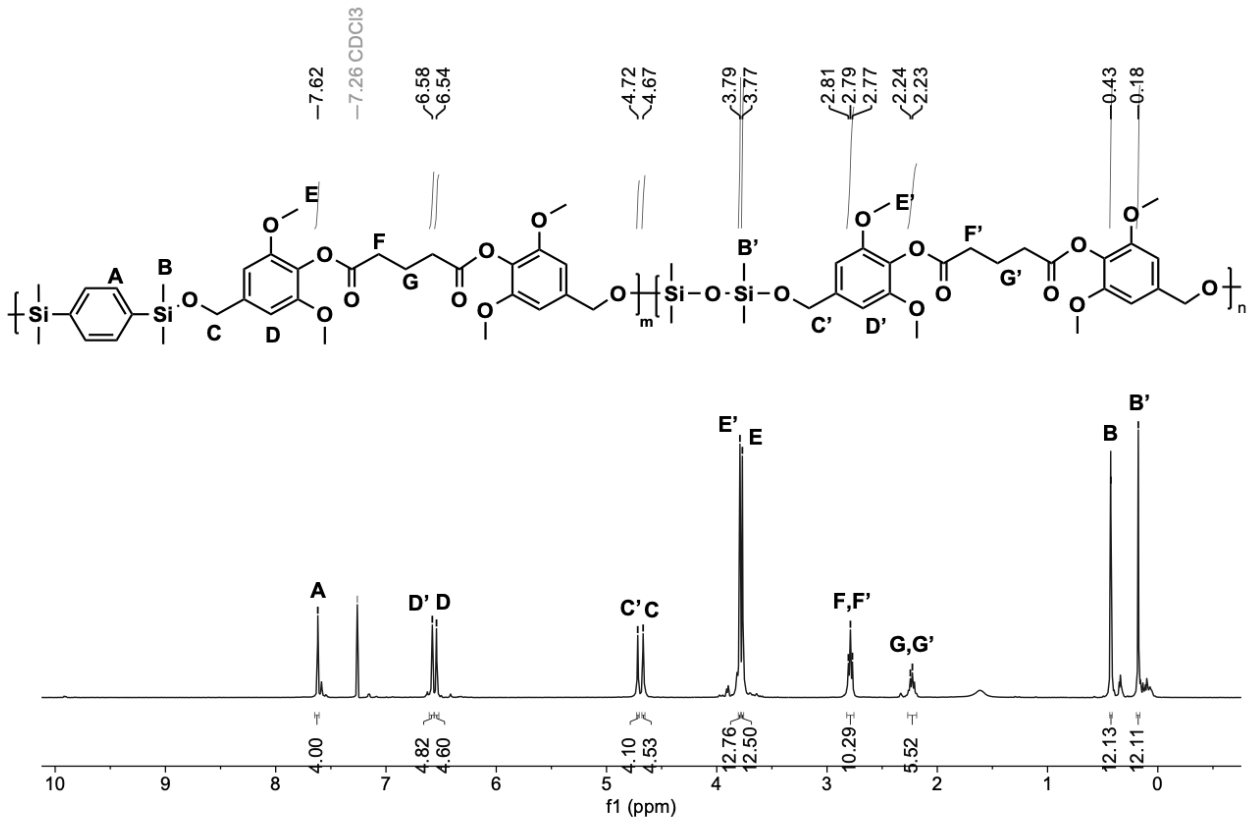

Figure S70 ^{1}H NMR spectrum (400 MHz, $CDCl_3$) of **poly(GA-Sy-*co*-BDMSB-*r*-TMDS)**.

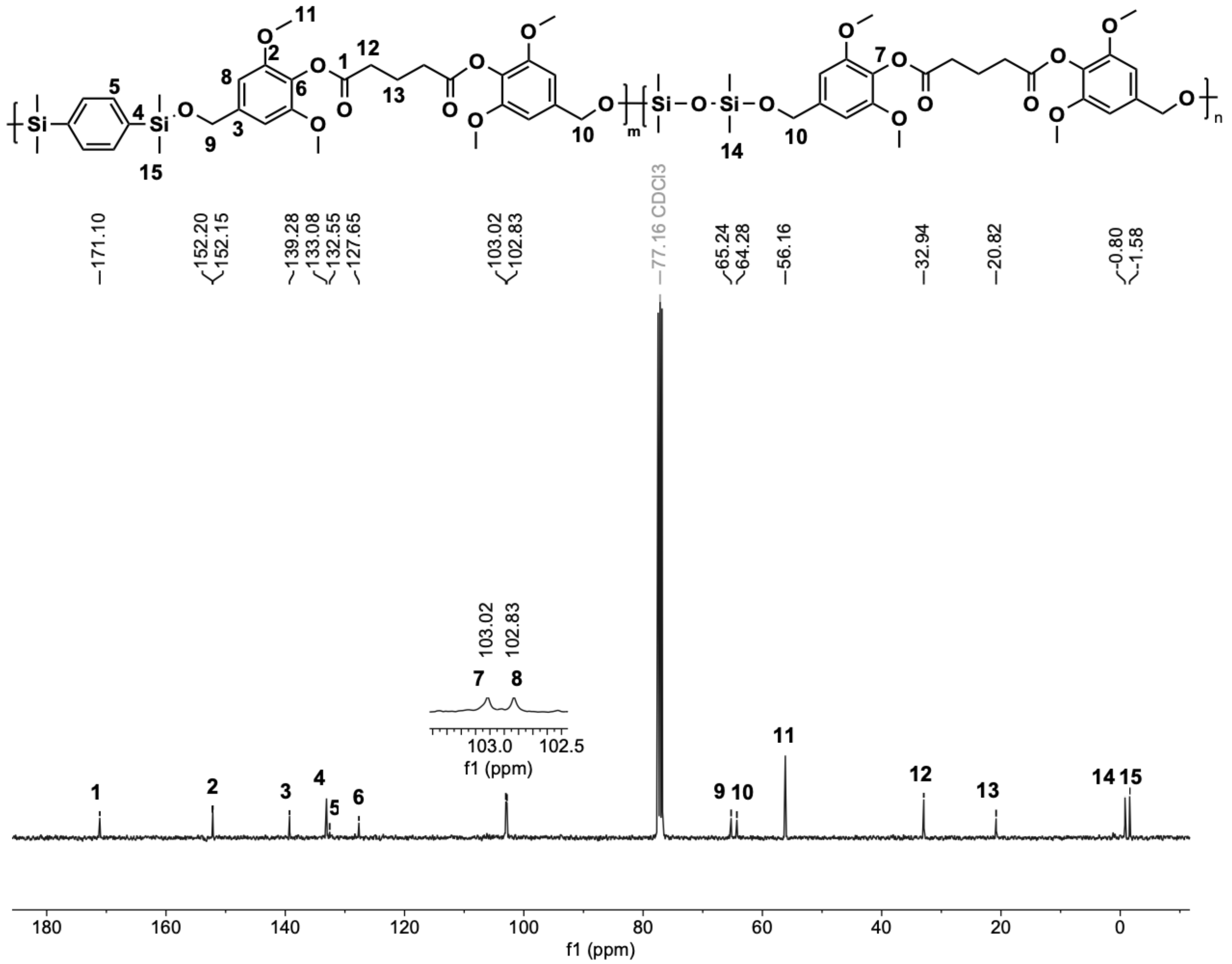

Figure S71 $^{13}C\{^1H\}$ NMR spectrum (101 MHz, $CDCl_3$) of **poly(GA-Sy-*co*-BDMSB-*r*-TMDS)**.

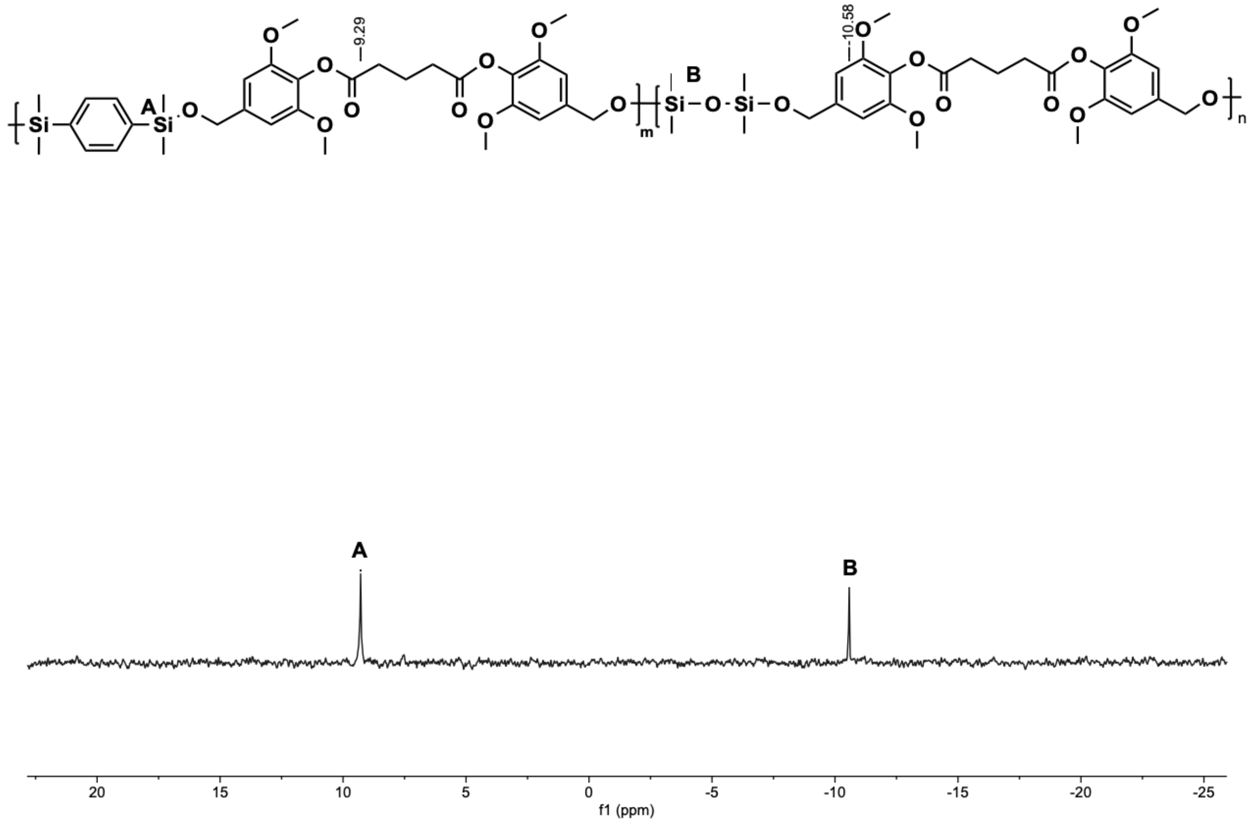

Figure S72 ^{29}Si NMR spectrum (99 MHz, $CDCl_3$) of **poly(GA-Sy-*co*-BDMSB-*r*-TMDS)**.

Fourier transform infrared spectroscopy (FTIR)

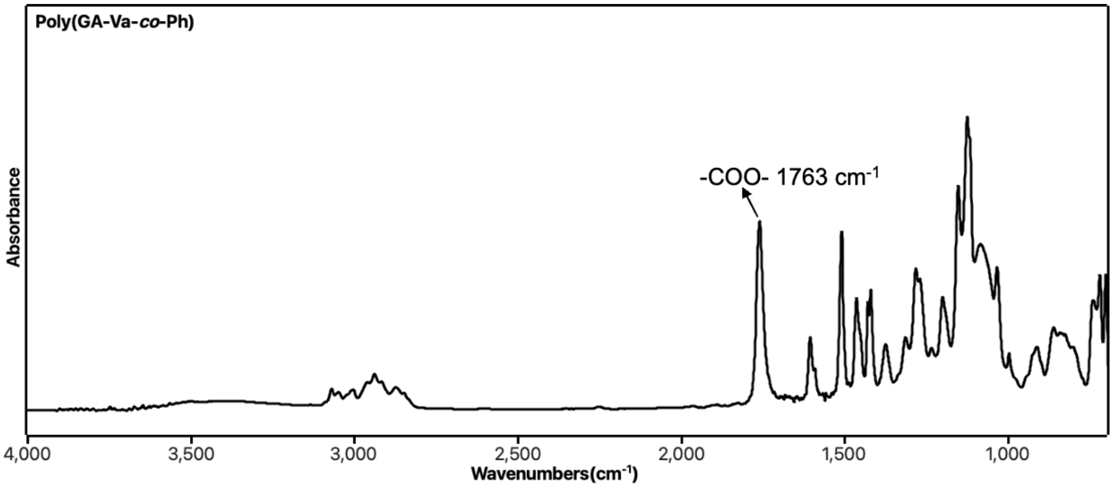

Figure S73 FT-IR spectrum of **poly(GA-Va-*co*-Ph)**.

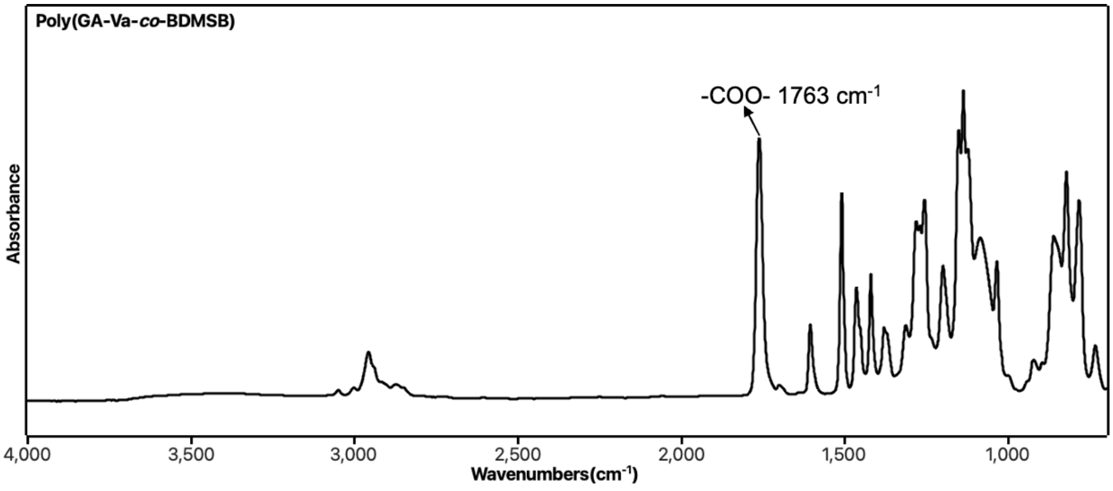

Figure S74 FT-IR spectrum of **poly(GA-Va-*co*-BDMSB)**.

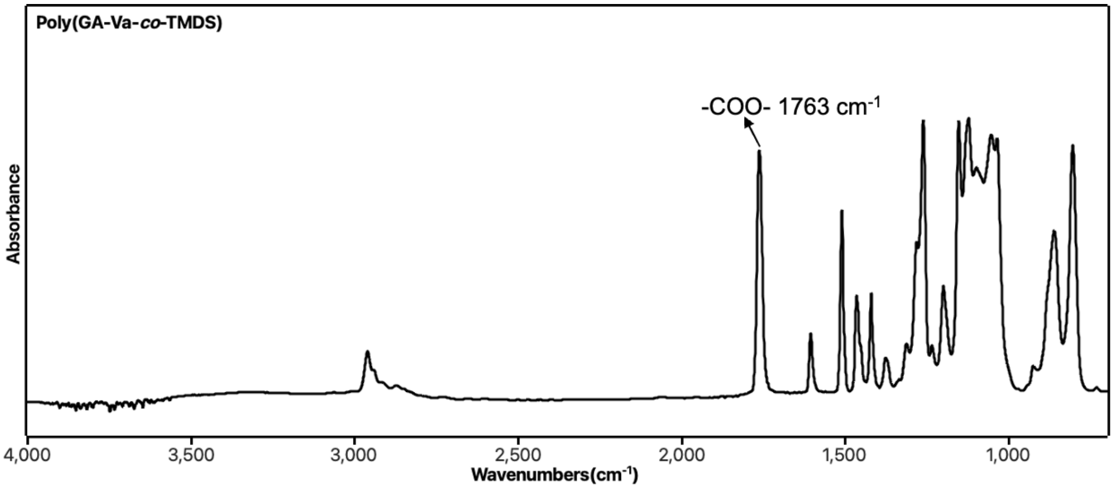

Figure S75 FT-IR spectrum of **poly(GA-Va-*co*-TMDS)**.

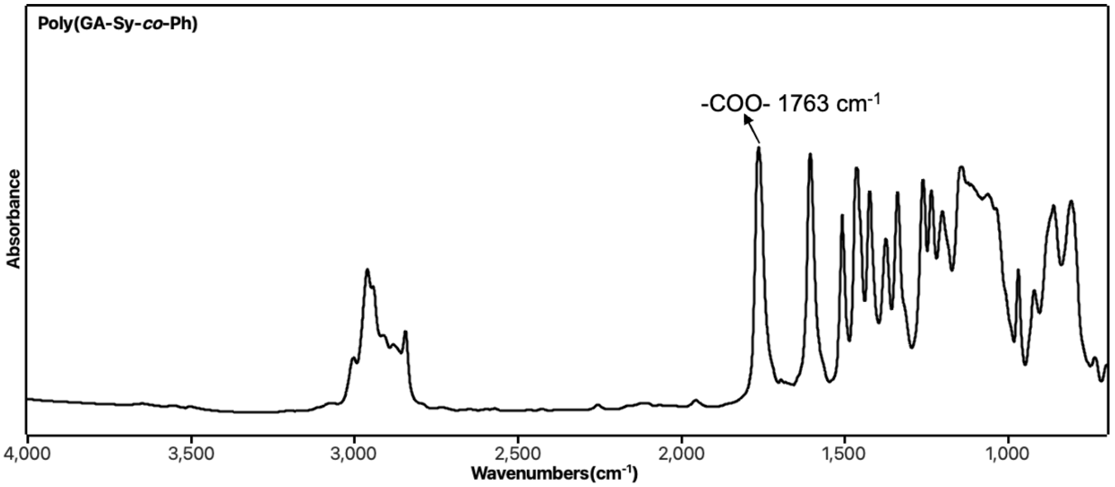

Figure S76 FT-IR spectrum of **poly(GA-Sy-*co*-Ph)**.

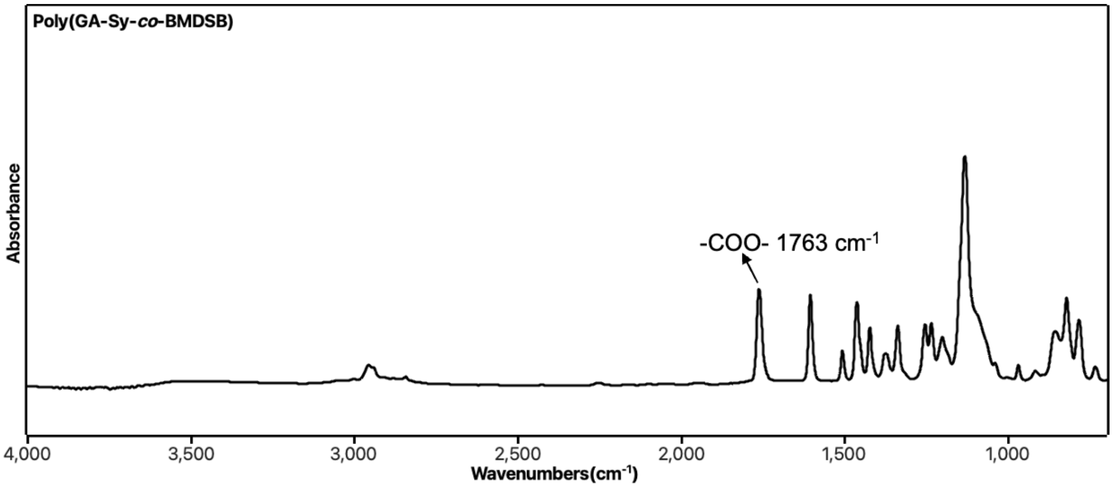

Figure S77 FT-IR spectrum of **poly(GA-Sy-co-BDMSB)**.

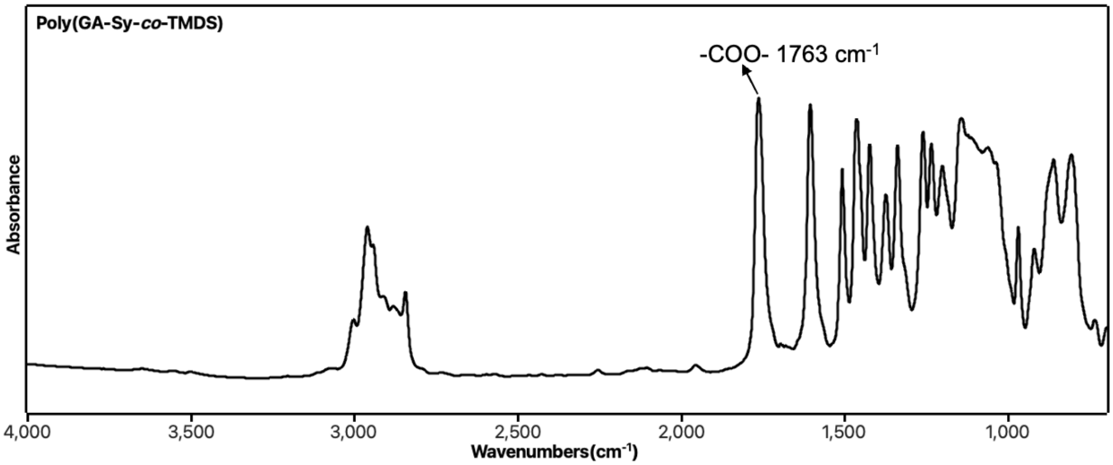

Figure S78 FT-IR spectrum of **poly(GA-Sy-*co*-TMDS)**.

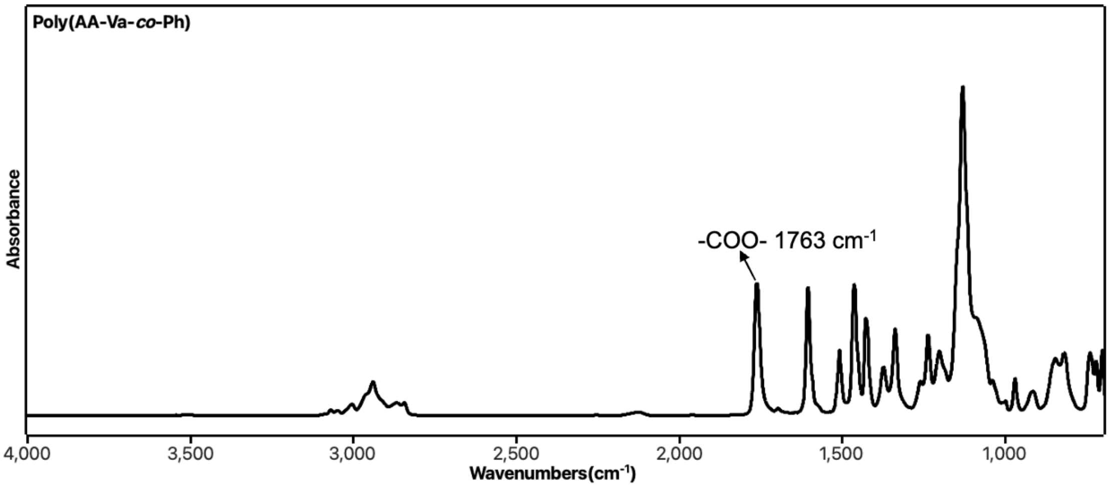

Figure S79 FT-IR spectrum of **poly(AA-Va-co-Ph)**.

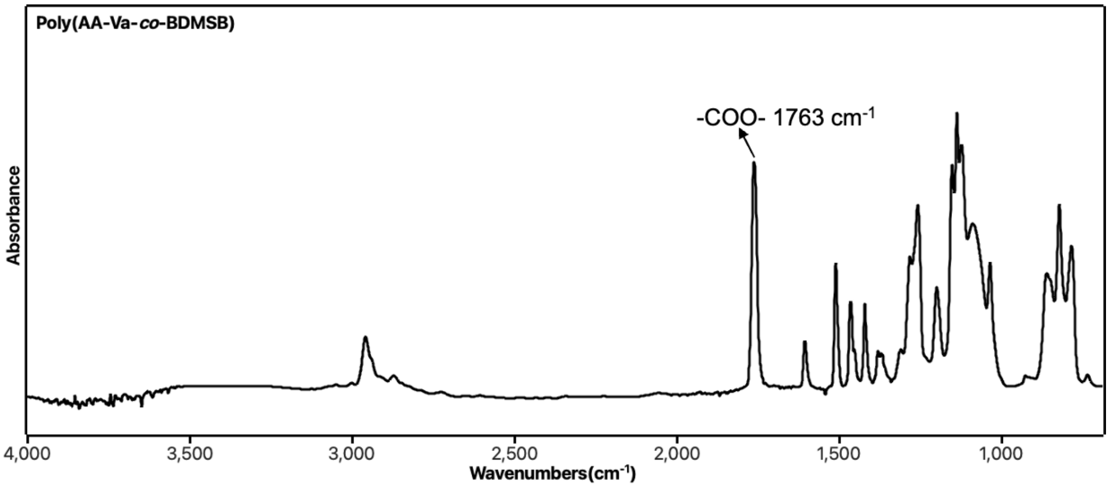

Figure S80 FT-IR spectrum of **poly(AA-Va-*co*-BDMSB)**.

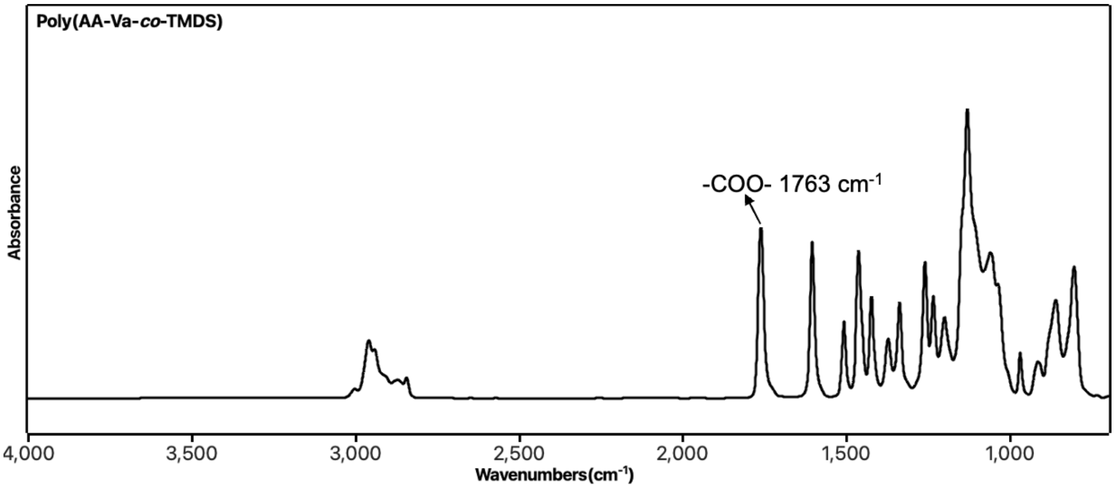

Figure S81 FT-IR spectrum of **poly(AA-Va-co-TMDS)**.

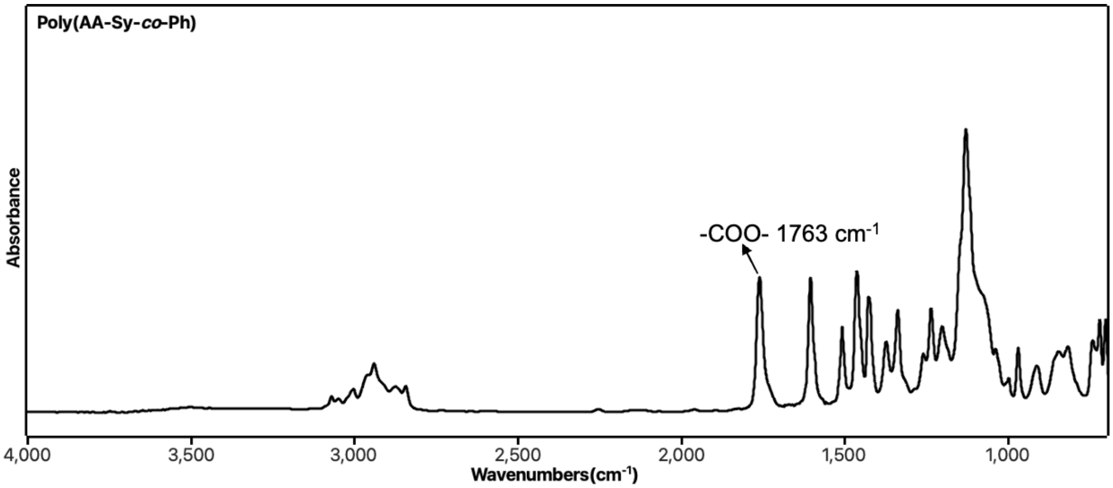

Figure S82 FT-IR spectrum of **poly(AA-Sy-*co*-Ph)**.

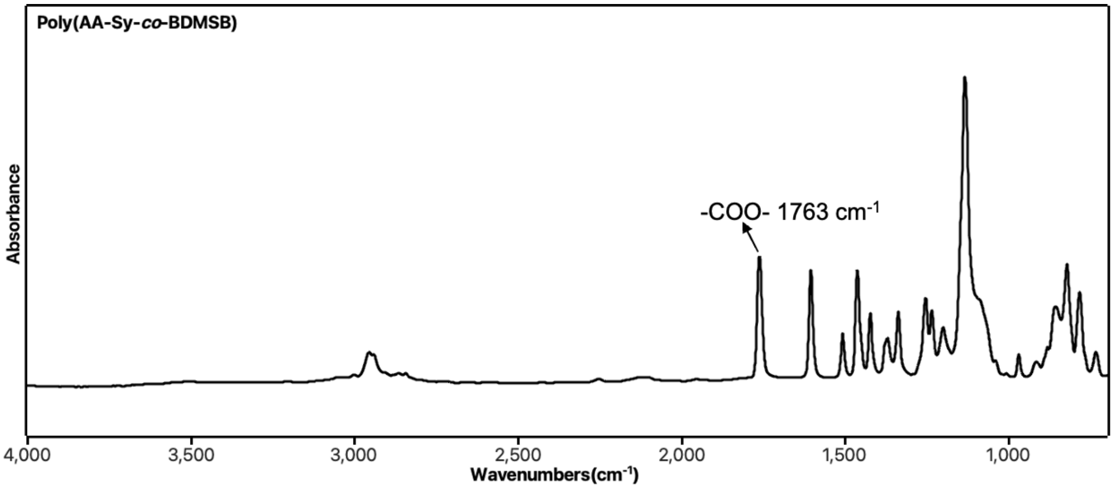

Figure S83 FT-IR spectrum of **poly(AA-Sy-*co*-BDMSB)**.

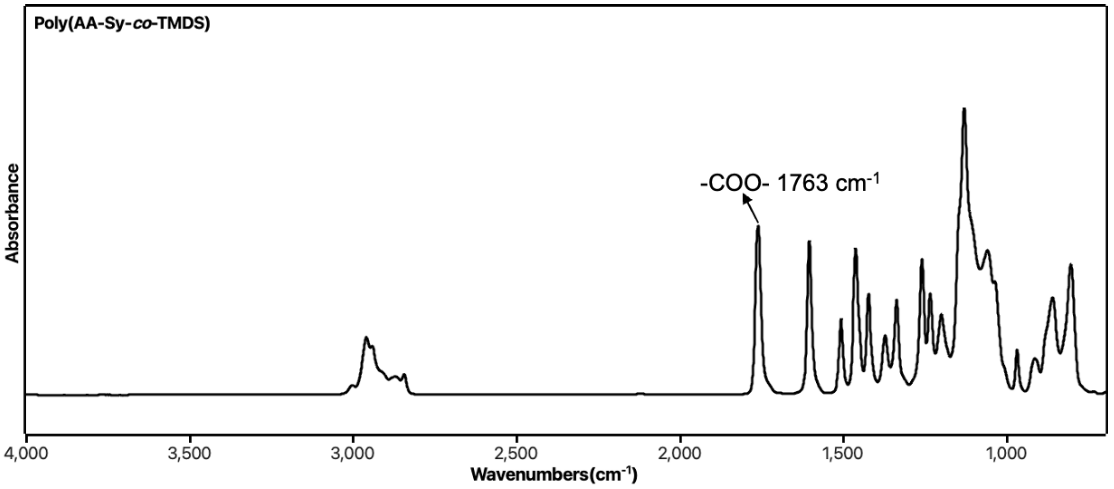

Figure S84 FT-IR spectrum of **poly(AA-Sy-*co*-TMDS)**.

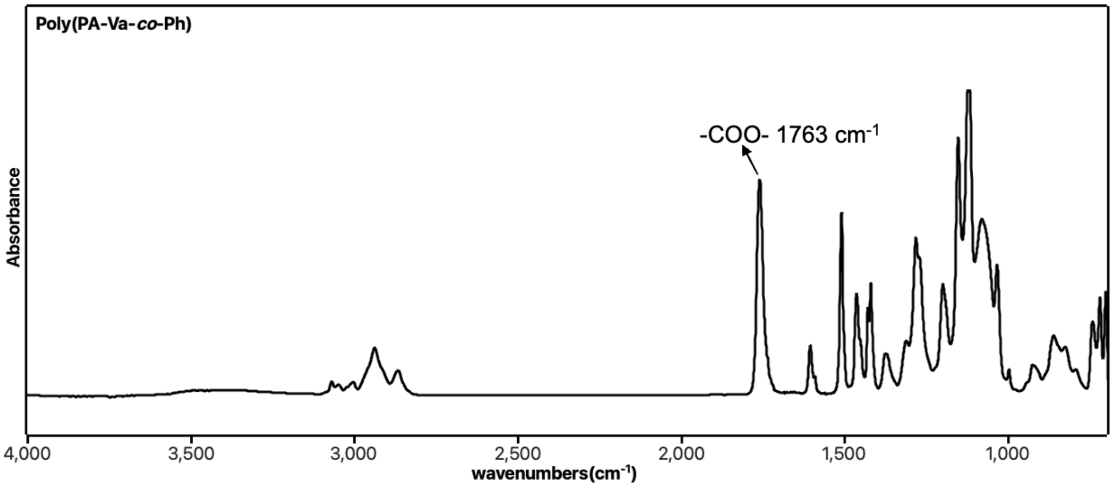

Figure S85 FT-IR spectrum of **poly(PA-Va-co-Ph)**.

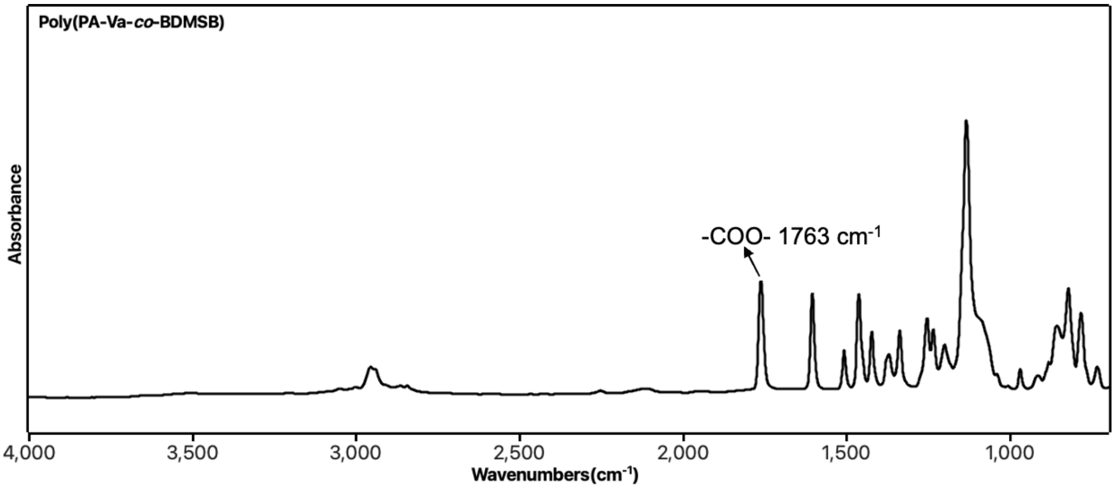

Figure S86 FT-IR spectrum of **poly(PA-Va-*co*-BDMSB)**.

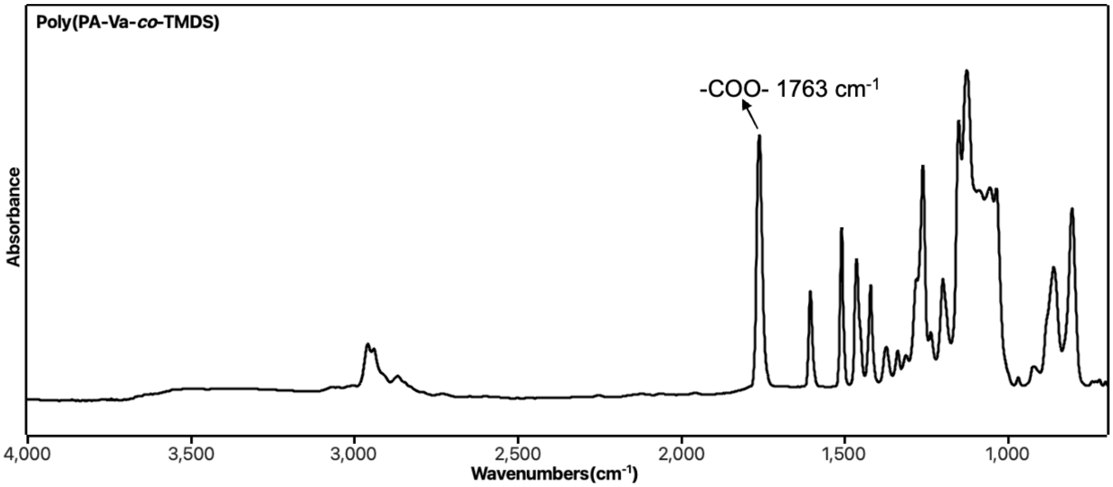

Figure S87 FT-IR spectrum of **poly(PA-Va-*co*-TMDS)**.

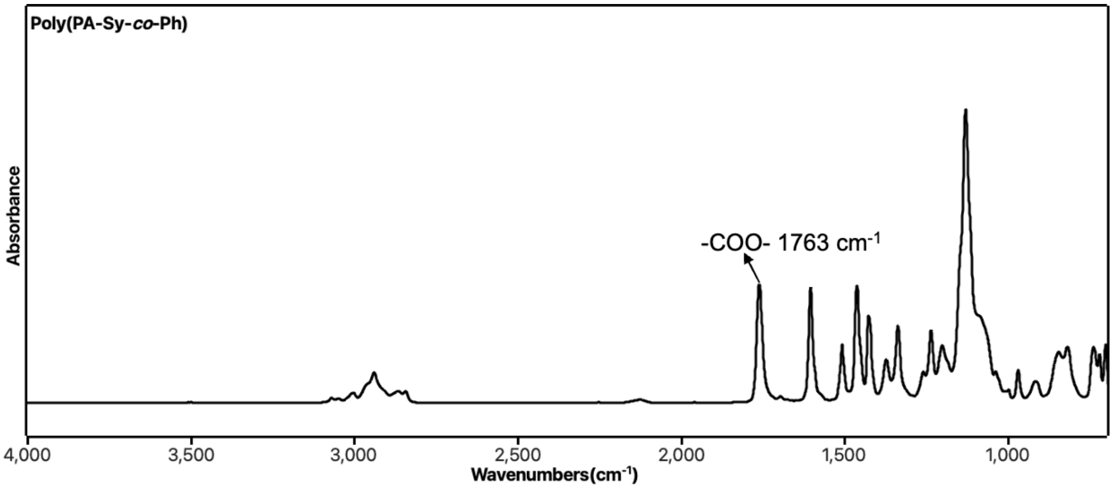

Figure S88 FT-IR spectrum of **poly(PA-Sy-*co*-Ph)**.

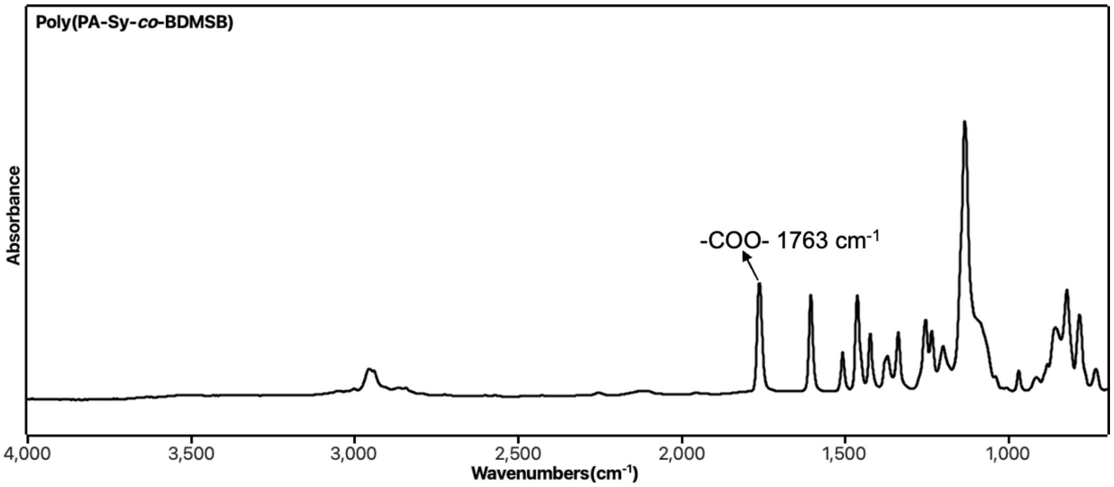

Figure S89 FT-IR spectrum of **poly(PA-Sy-*co*-BDMSB)**.

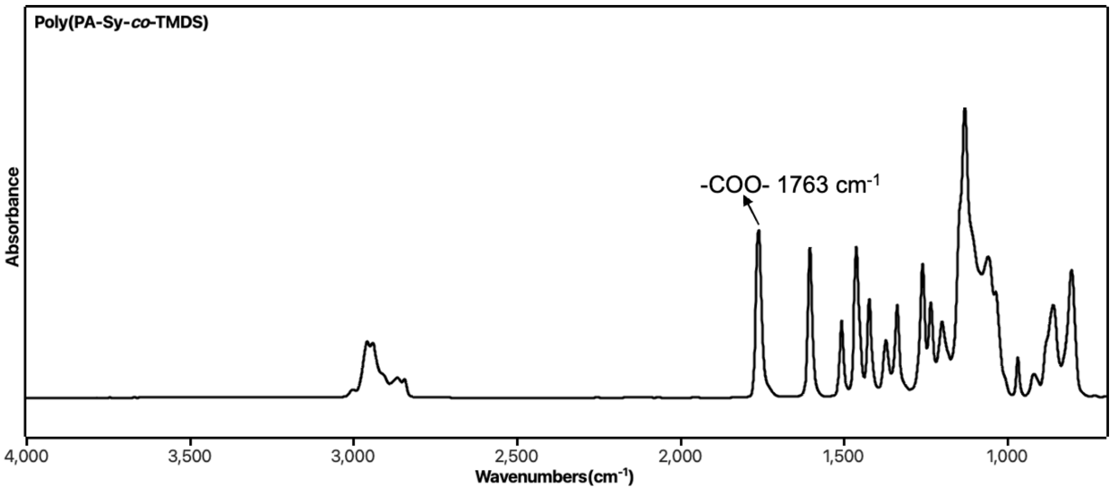

Figure S90 FT-IR spectrum of **poly(PA-Sy-*co*-TMDS)**.

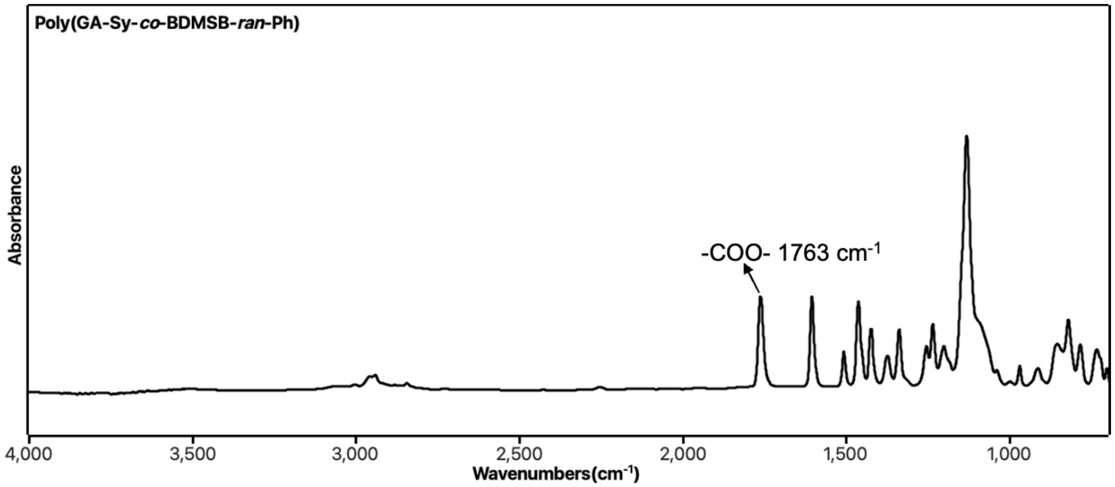

Figure S91 FT-IR spectrum of **poly(GA-Sy-co-BDMSB-*r*-Ph)**.

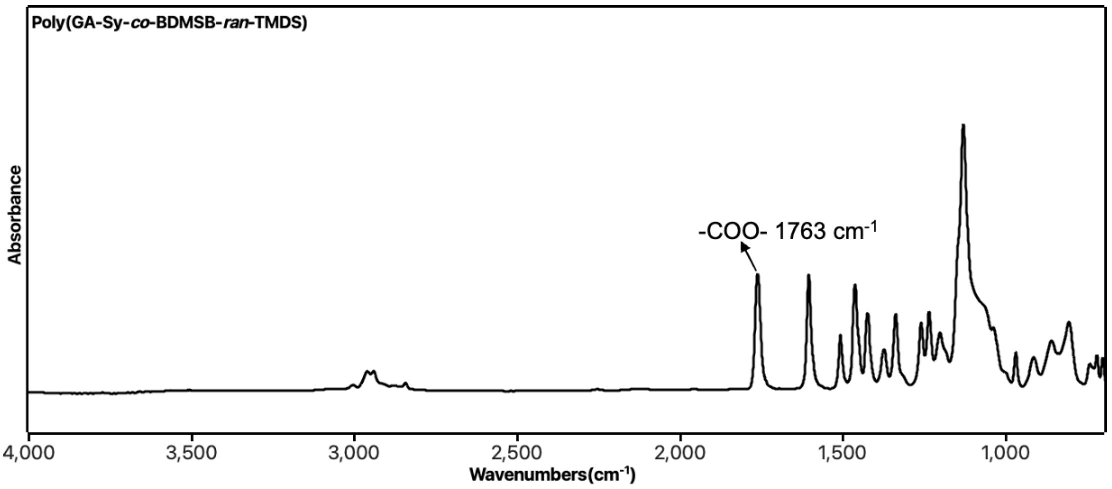

Figure S92 FT-IR spectrum of **poly(GA-Sy-*co*-BDMSB-*r*-TMDS)**.

SEC-RI traces of the synthesis of bio-based PSEs

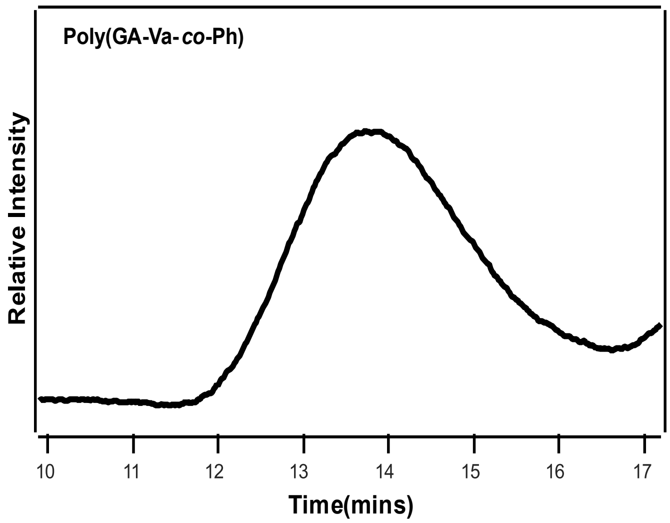

Figure S93 SEC-RI traces of **poly(GA-Va-*co*-Ph)**.

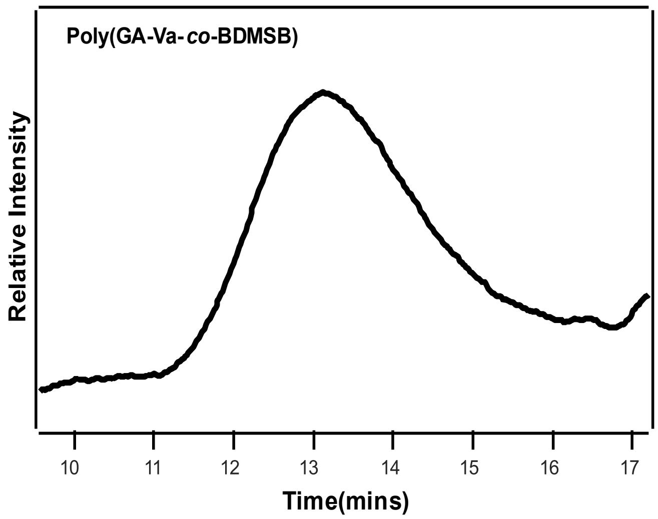

Figure S94 SEC-RI traces of **poly(GA-Va-*co*-BDMSB)**.

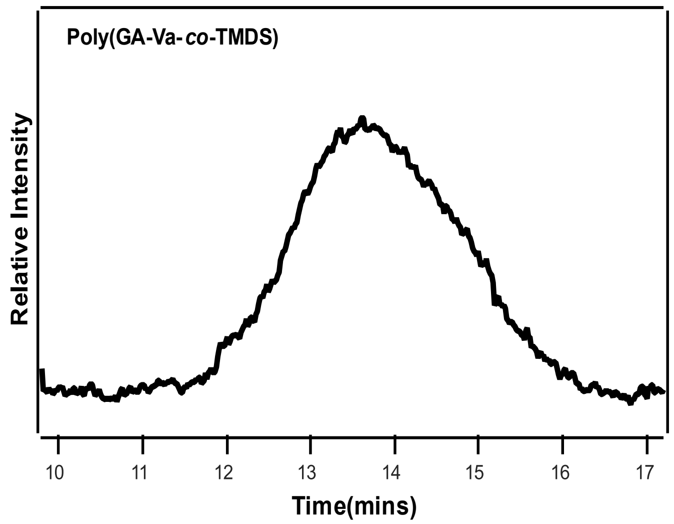

Figure S95 SEC-RI traces of **poly(GA-Va-*co*-TMDS)**.

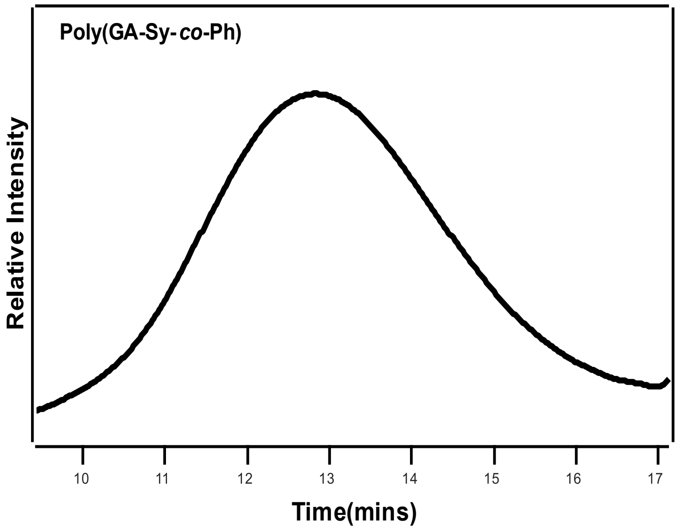

Figure S96 SEC-RI traces of **poly(GA-Sy-*co*-Ph)**.

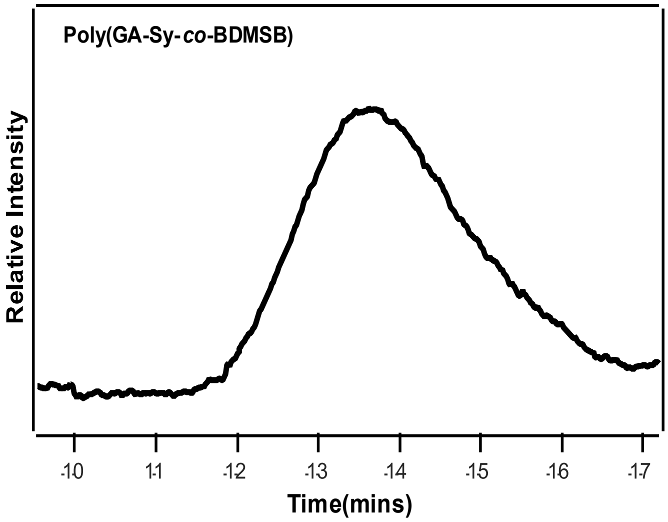

Figure S97 SEC-RI traces of **poly(GA-Sy-*co*-BDMSB)**.

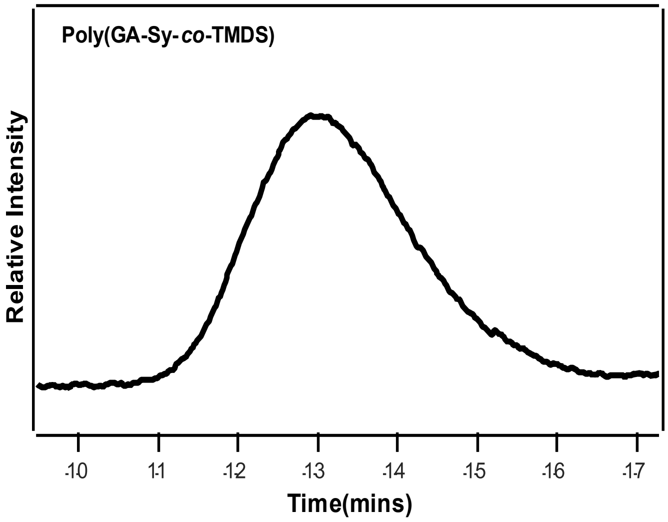

Figure S98 SEC-RI traces of **poly(GA-Sy-*co*-TMDS)**.

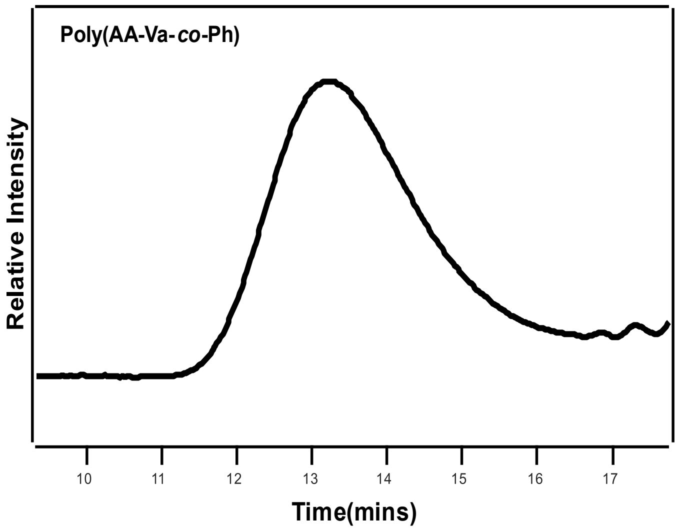

Figure S99 SEC-RI traces of **poly(AA-Va-*co*-Ph)**.

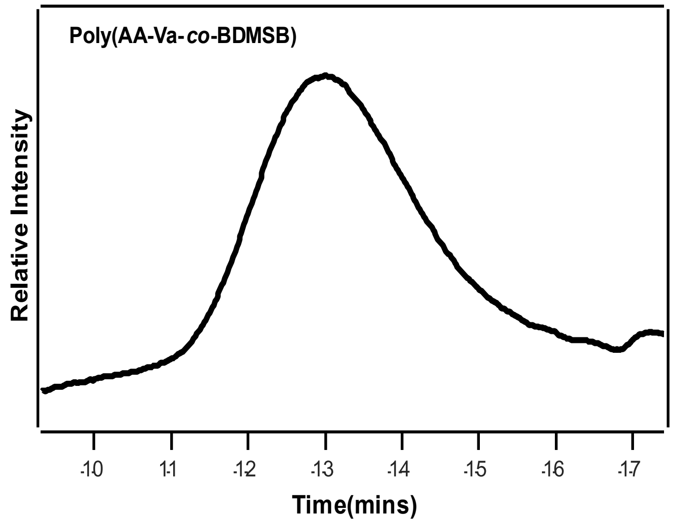

Figure S100 SEC-RI traces of **poly(AA-Va-*co*-BDMSB)**.

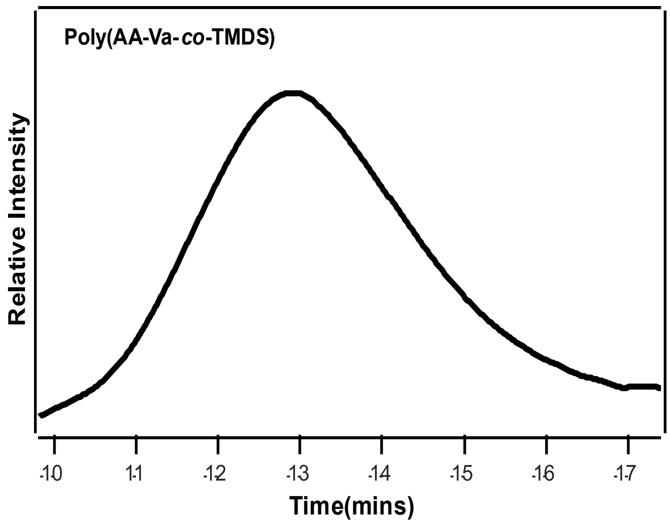

Figure S101 SEC-RI traces of **poly(AA-Va-*co*-TMDS)**.

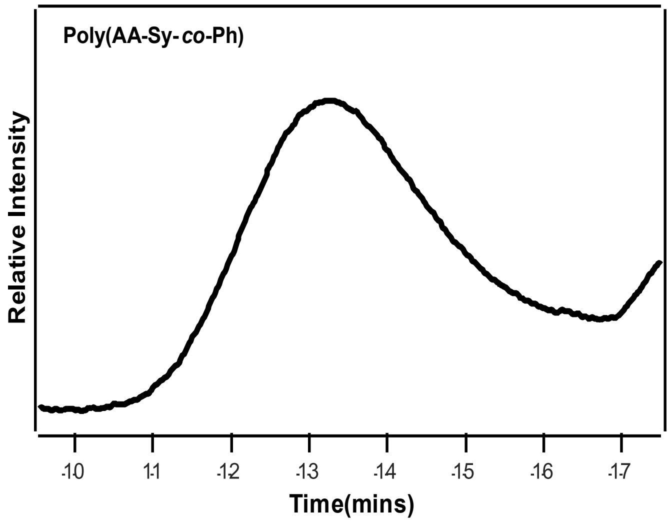

Figure S102 SEC-RI traces of **poly(AA-Sy-*co*-Ph)**.

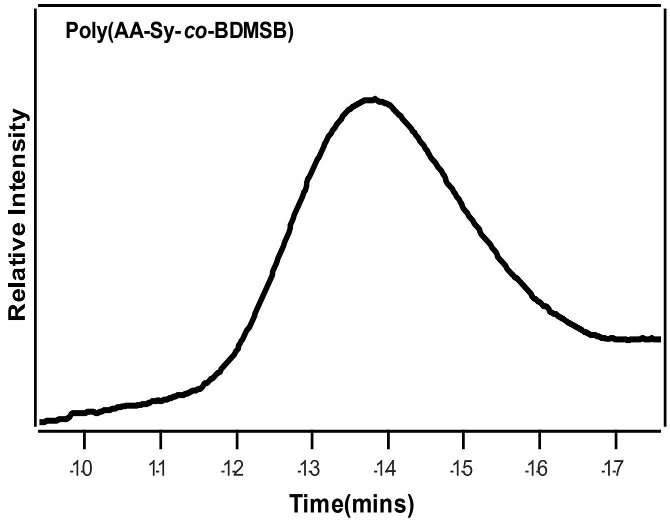

Figure S103 SEC-RI traces of **poly(AA-Sy-*co*-BDMSB)**.

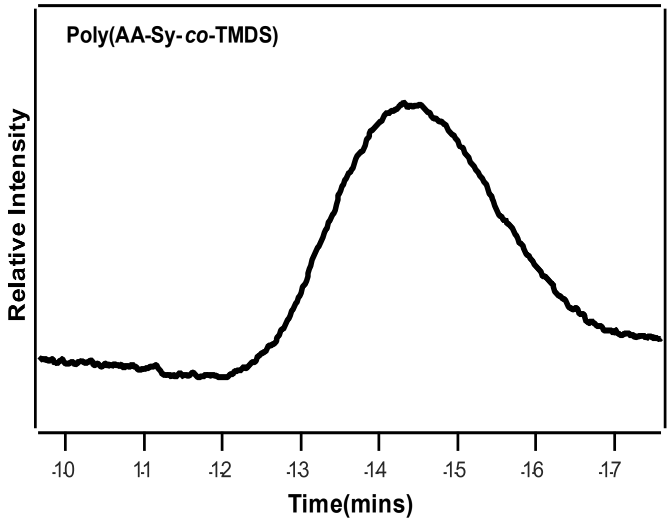

Figure S104 SEC-RI traces of **poly(AA-Sy-*co*-TMDS)**.

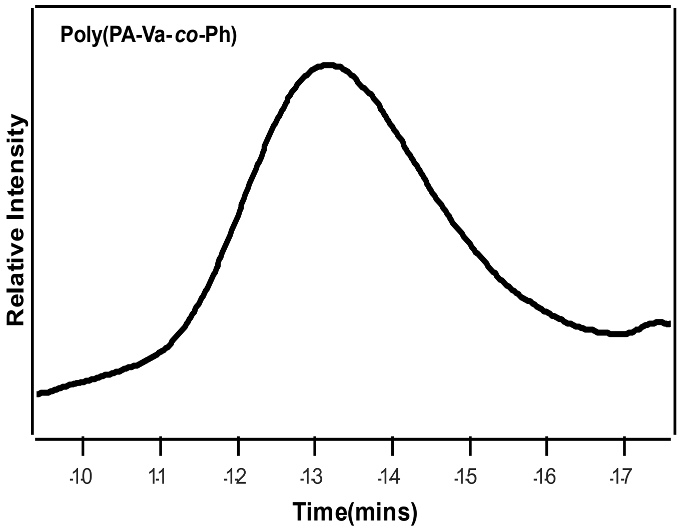

Figure S105 SEC-RI traces of **poly(PA-Va-*co*-Ph)**.

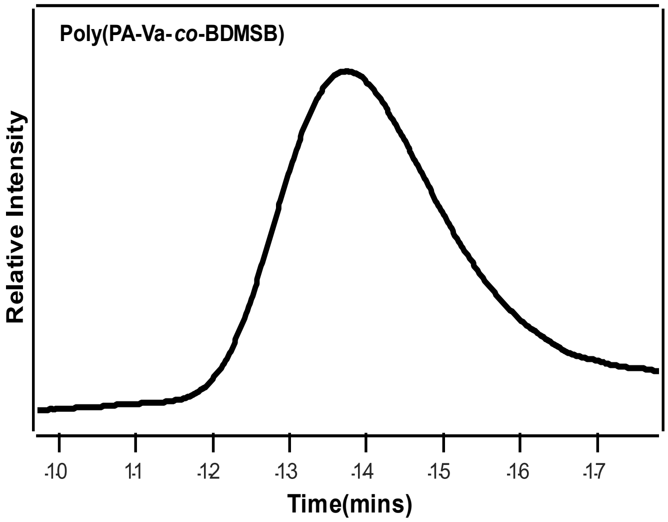

Figure S106 SEC-RI traces of **poly(PA-Va-*co*-BDMSB)**.

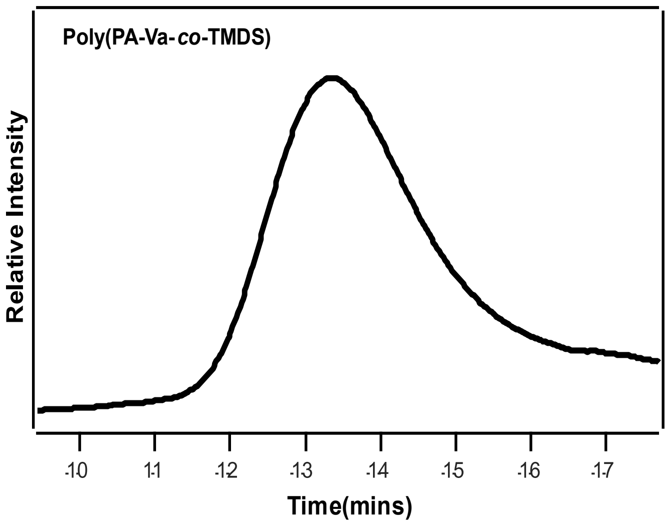

Figure S107 SEC-RI traces of **poly(PA-Va-*co*-TMDS)**.

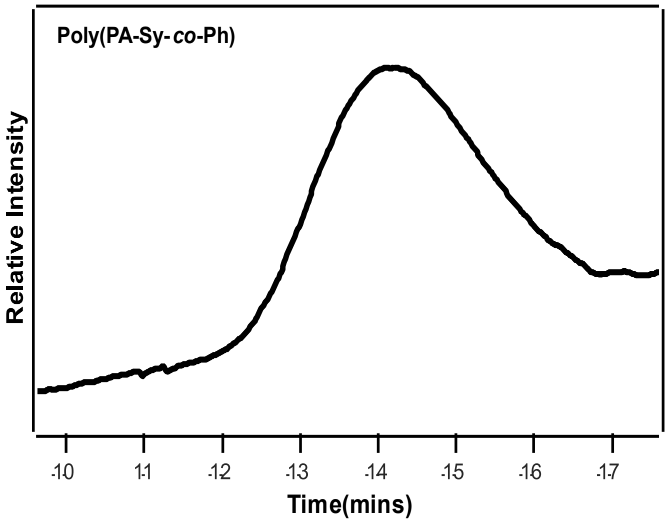

Figure S108 SEC-RI traces of **poly(PA-Sy-*co*-Ph)**.

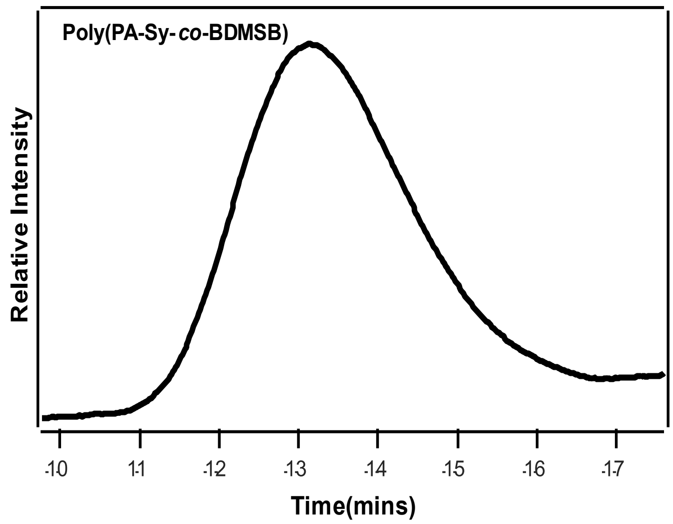

Figure S109 SEC-RI traces of **poly(PA-Sy-*co*-BDMSB)**.

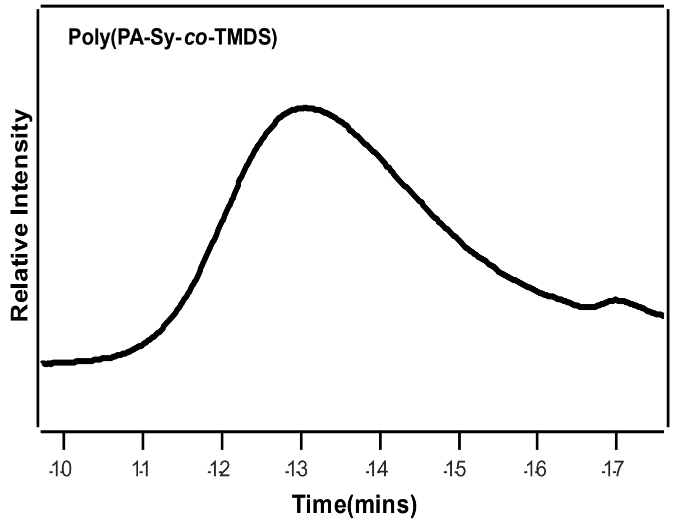

Figure S110 SEC-RI traces of **poly(PA-Sy-*co*-TMDS)**.

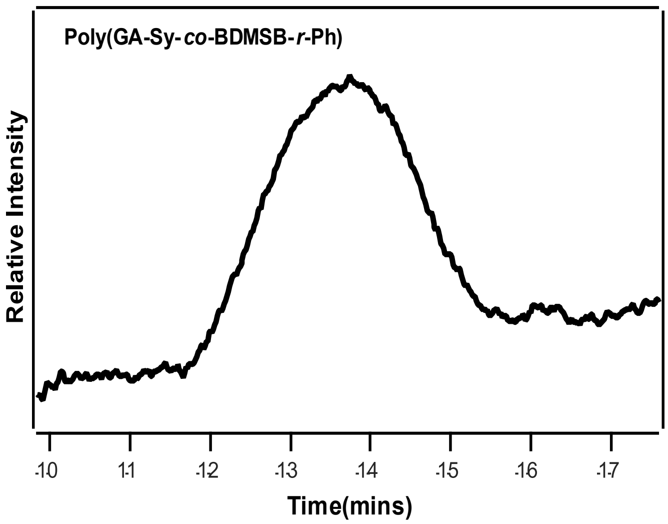

Figure S111 SEC-RI traces of **poly(GA-Sy-*co*-BDMSB-*r*-Ph)**.

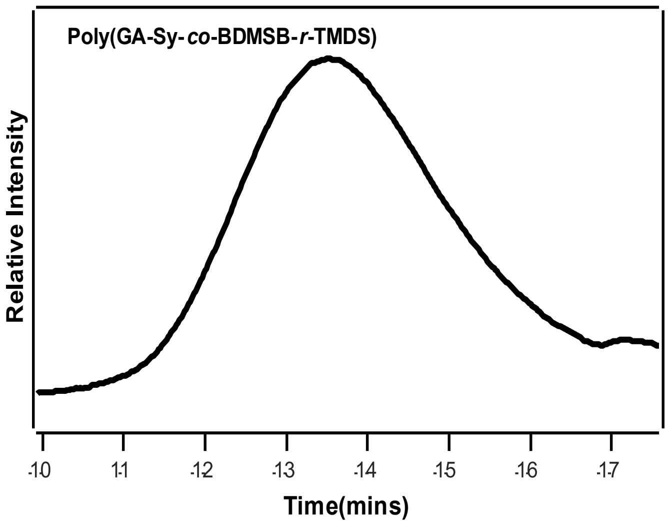

Figure S112 SEC-RI traces of **poly(GA-Sy-*co*-BDMSB-*r*-TMDS)**.

Thermal performance of bio-based PSEs

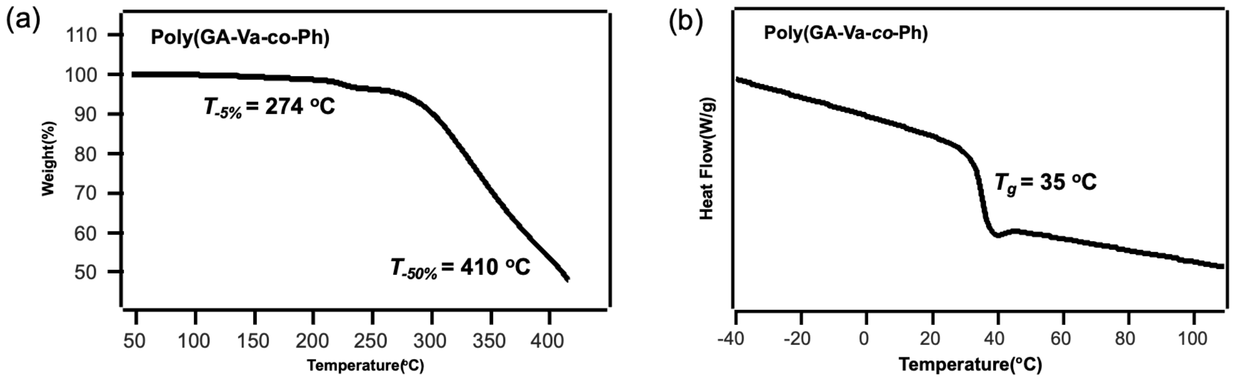

Figure S113 (a) TGA thermogram and (b) DSC curve of **poly(GA-Va-*co*-Ph)**.

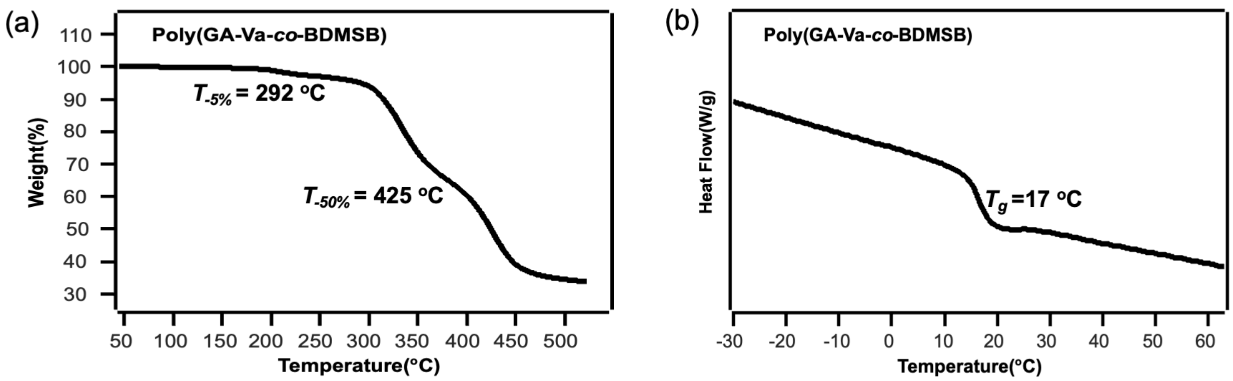

Figure S114 (a) TGA thermogram and (b) DSC curve of **poly(GA-Va-*co*-BDMSB)**.

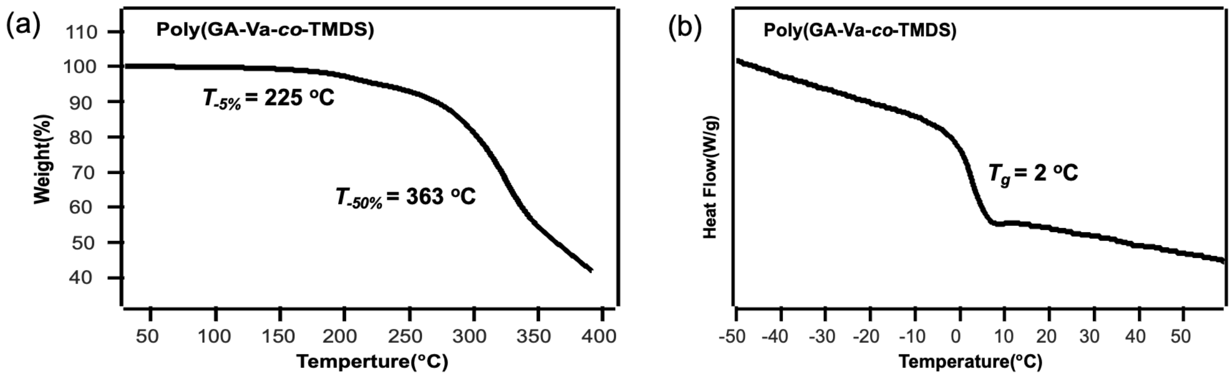

Figure S115 (a) TGA thermogram and (b) DSC curve of **poly(GA-Va-*co*-TMDS)**.

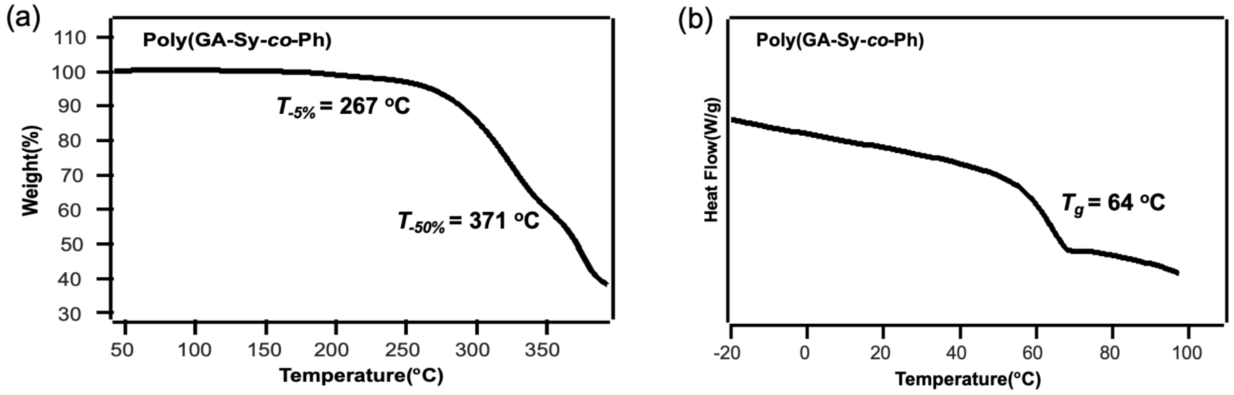

Figure S116 (a) TGA thermogram and (b) DSC curve of **poly(GA-Sy-*co*-Ph)**.

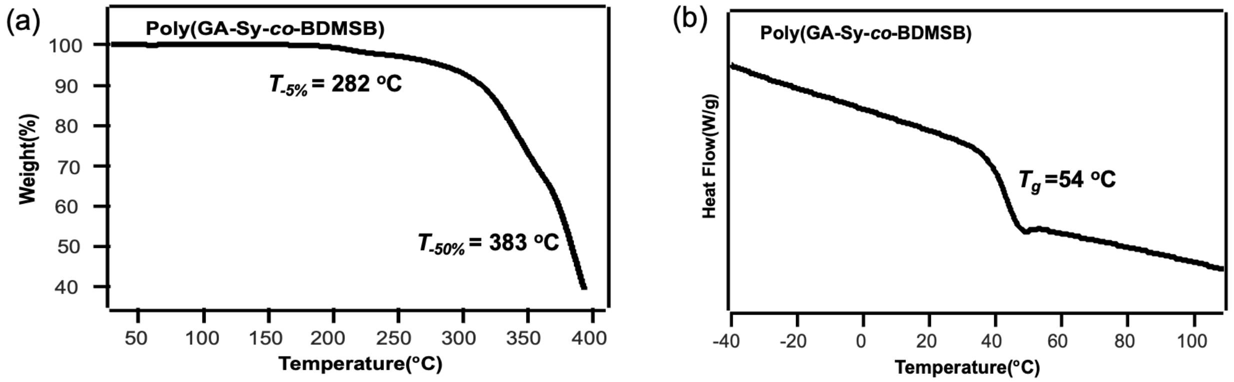

Figure S117 (a) TGA thermogram and (b) DSC curve of **poly(GA-Sy-*co*-BDMSB)**.

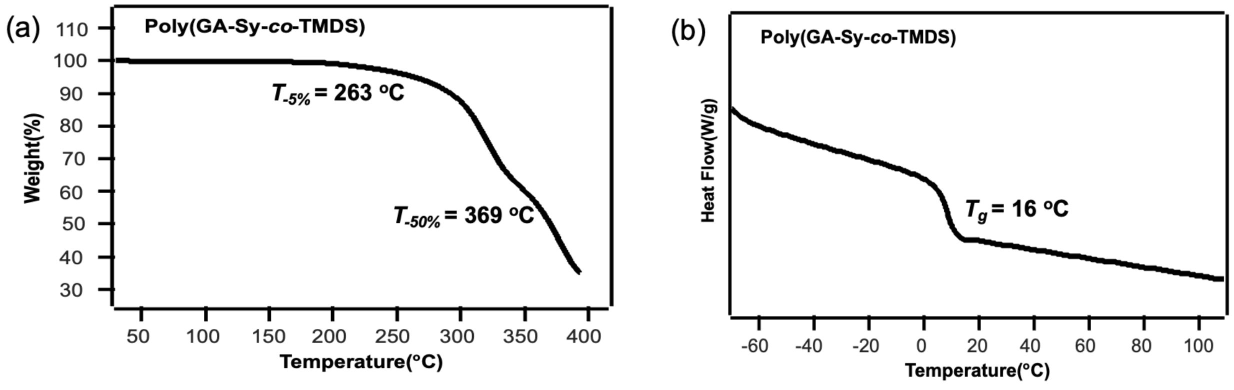

Figure S118 (a) TGA thermogram and (b) DSC curve of **poly(GA-Sy-*co*-TMDS)**.

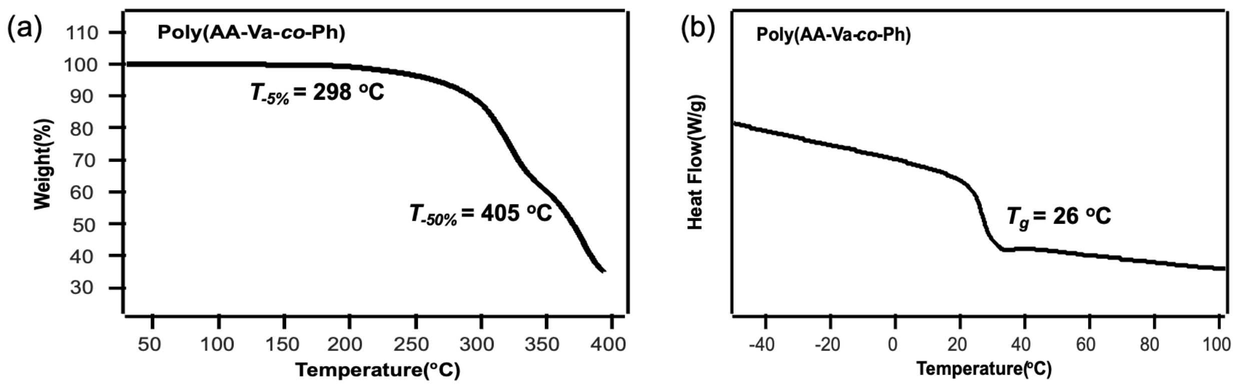

Figure S119 (a) TGA thermogram and (b) DSC curve of **poly(AA-Va-*co*-Ph)**.

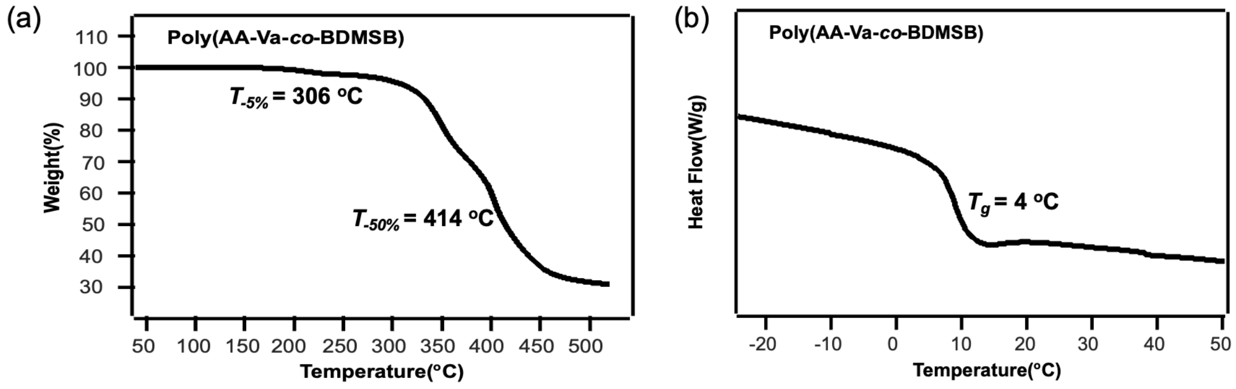

Figure S120 (a) TGA thermogram and (b) DSC curve of **poly(AA-Va-*co*-BDMSB)**.

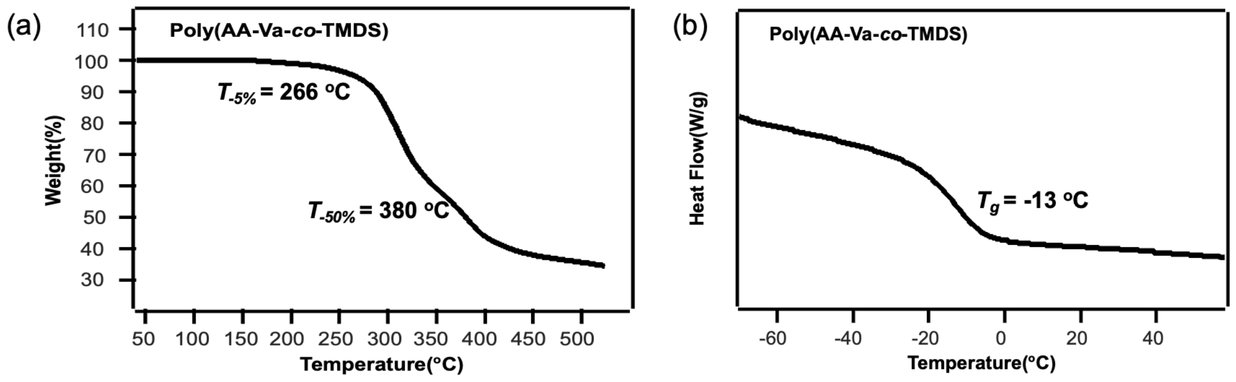

Figure S121 (a) TGA thermogram and (b) DSC curve of **poly(AA-Va-*co*-TMDS)**.

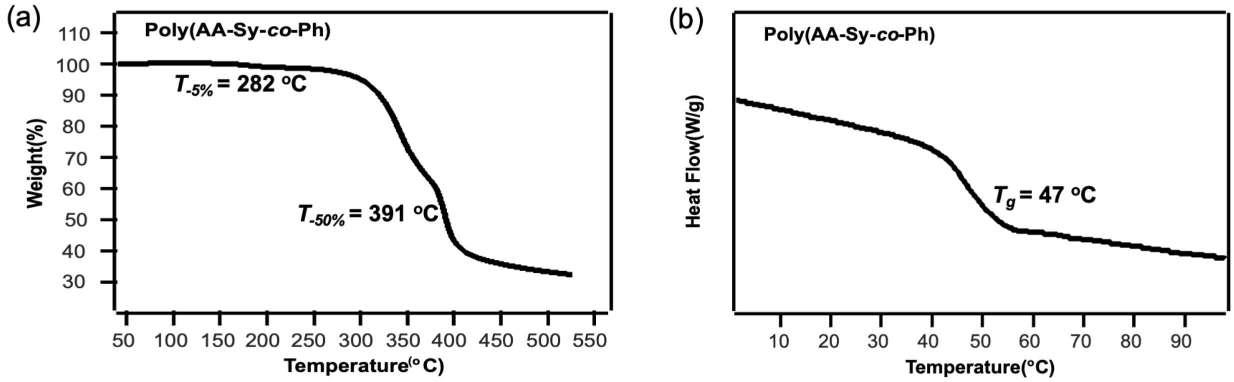

Figure S122 (a) TGA thermogram and (b) DSC curve of **poly(AA-Sy-*co*-Ph)**.

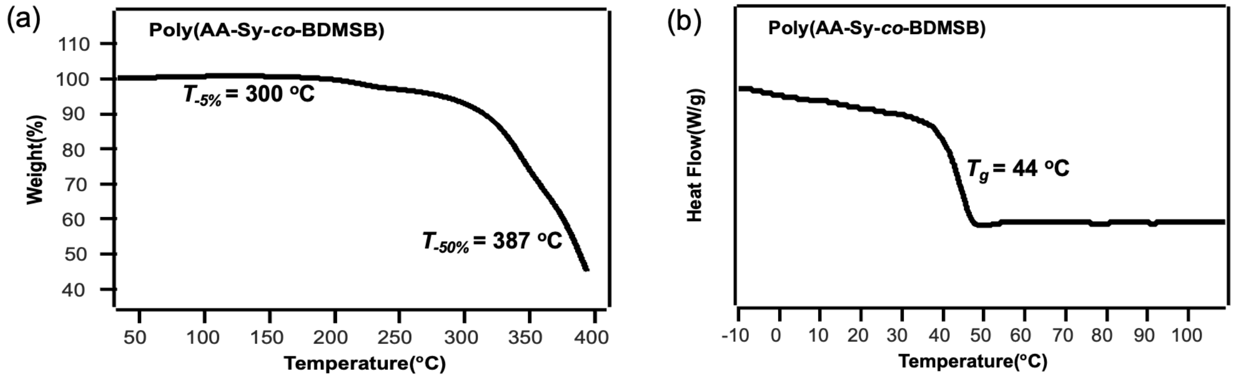

Figure S123 (a) TGA thermogram and (b) DSC curve of **poly(AA-Sy-*co*-BDMSB)**.

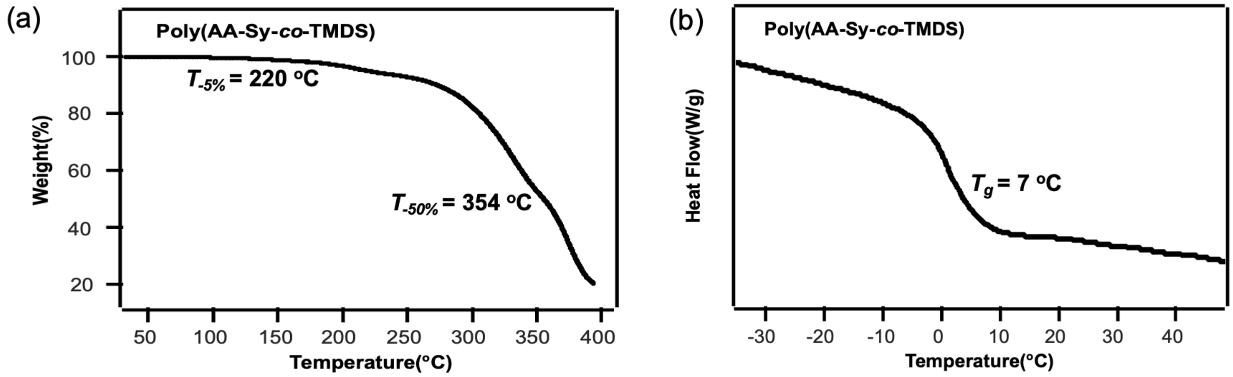

Figure S124 (a) TGA thermogram and (b) DSC curve of **poly(AA-Sy-*co*-TMDS)**.

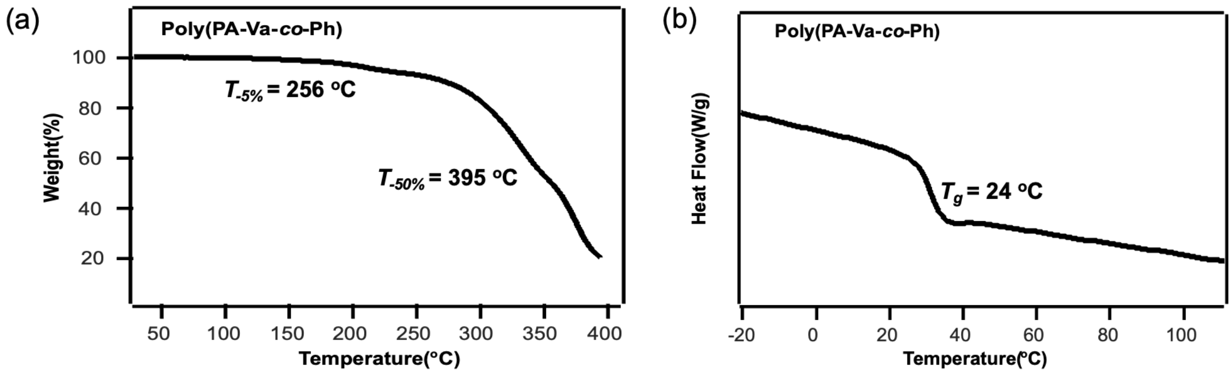

Figure S125 (a) TGA thermogram and (b) DSC curve of **poly(PA-Va-*co*-Ph)**.

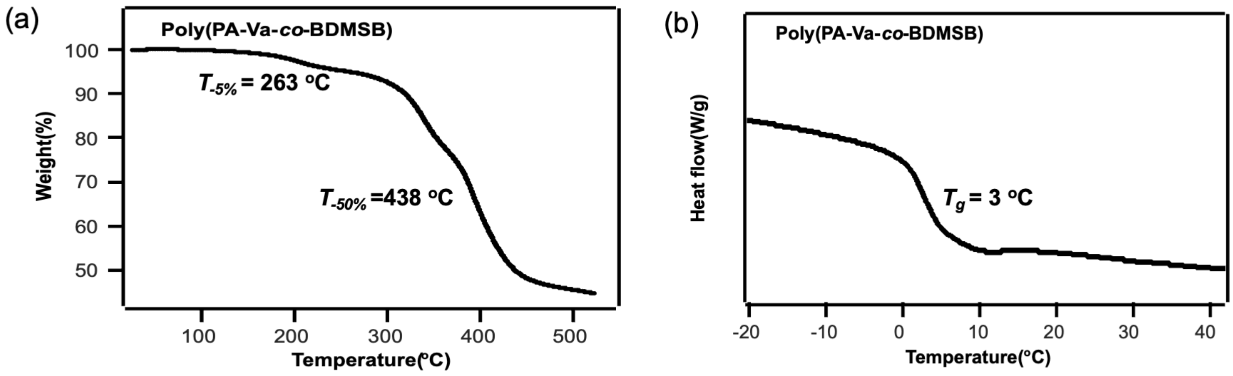

Figure S126 (a) TGA thermogram and (b) DSC curve of **poly(PA-Va-*co*-BDMSB)**.

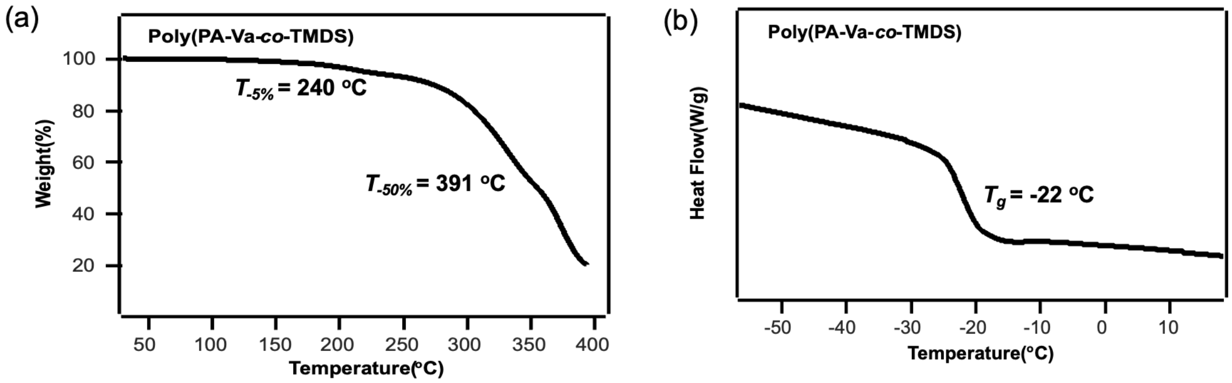

Figure S127 (a) TGA thermogram and (b) DSC curve of **poly(PA-Va-*co*-TMDS)**.

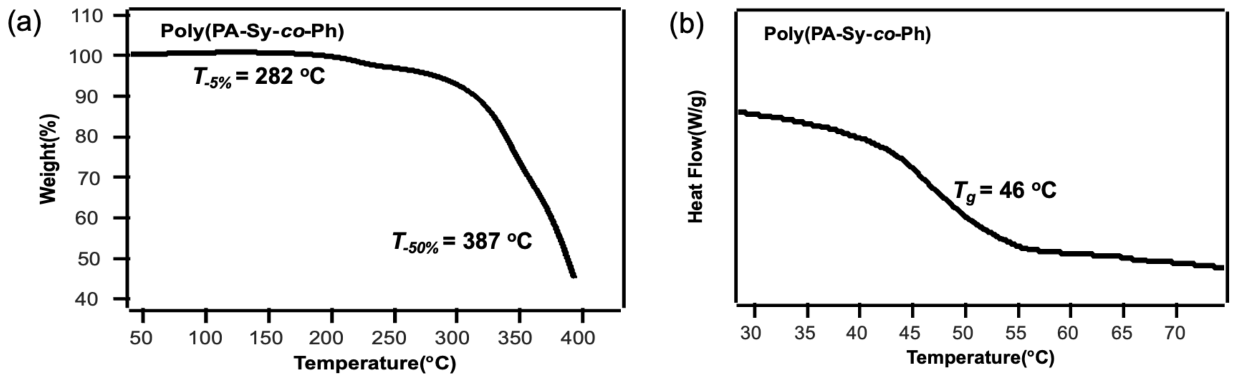

Figure S128 (a) TGA thermogram and (b) DSC curve of **poly(PA-Sy-*co*-Ph)**.

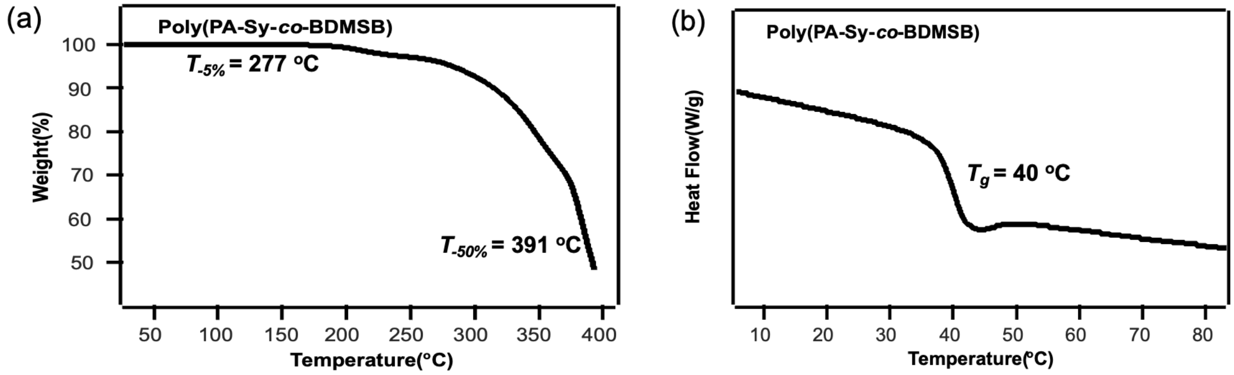

Figure S129 (a) TGA thermogram and (b) DSC curve of **poly(PA-Sy-*co*-BDMSB)**.

17

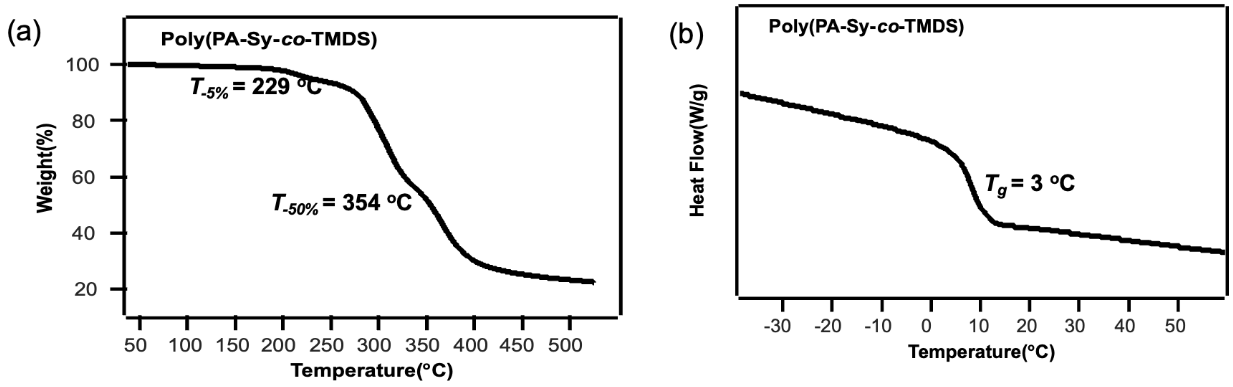

Figure S130 (a) TGA thermogram and (b) DSC curve of **poly(PA-Sy-*co*-TMDS)**.

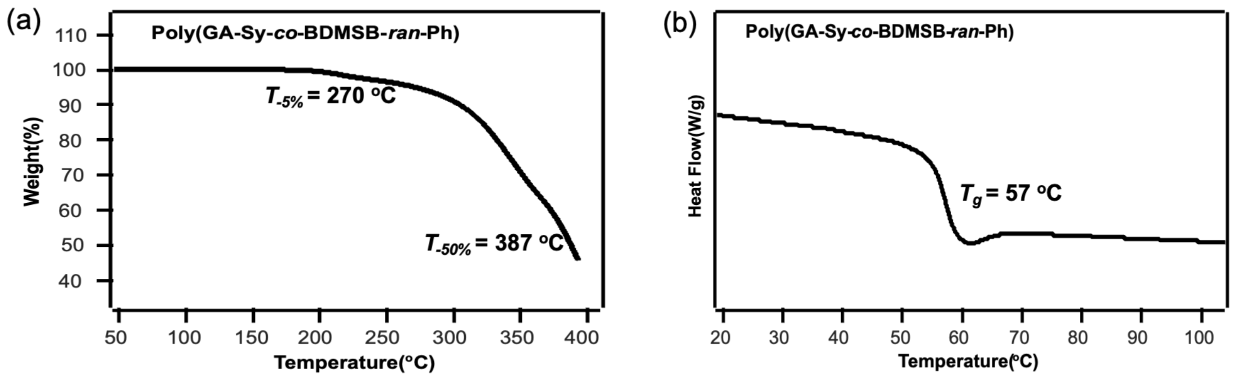

Figure S131 (a) TGA thermogram and (b) DSC curve of **poly(GA-Sy-*co*-BDMSB-*r*-Ph)**.

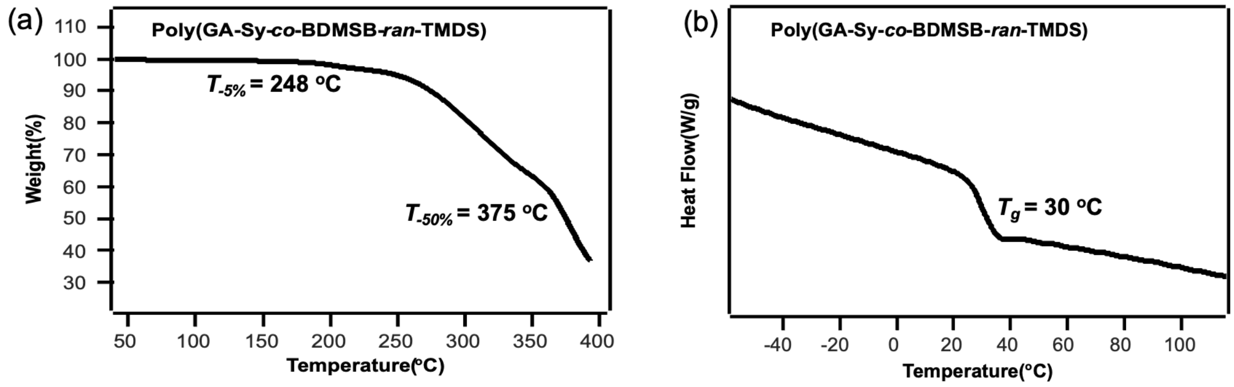

Figure S132 (a) TGA thermogram and (b) DSC curve of **poly(GA-Sy-*co*-BDMSB-*r*-TMDS)**.

Table S1 Catalytic synthesis of PSEs.[a]

Entry	Polymer	Time[b] (h)	Isolated yields(%)	M_n[c] (g/mol)	M_w[c] (g/mol)	$Đ$[c]
1	Poly(GA-Va-*co*-Ph)	2	67	11000	20000	1.80
2	Poly(GA-Va-*co*-BDMSB)	0.25	73	20000	36000	1.73
3	Poly(GA-Va-*co*-TMDS)	0.5	81	14200	22200	1.58
4[d]	Poly(GA-Sy-*co*-Ph)	4	83	18500	52000	2.80
5	Poly(GA-Sy-*co*-BDMSB)	0.25	70	12900	22100	1.70
6	Poly(GA-Sy-*co*-TMDS)	0.5	75	20600	36030	1.74
7	Poly(AA-Va-*co*-Ph)	2	82	17000	41900	2.46
8	Poly(AA-Va-*co*-BDMSB)	0.08	71	23000	38000	1.64
9	Poly(AA-Va-*co*-TMDS)	0.25	84	23000	47000	2.04
10[d]	Poly(AA-Sy-*co*-Ph)	4	88	17000	33000	1.92
11	Poly(AA-Sy-*co*-BDMSB)	0.5	81	11900	22000	1.85
12	Poly(AA-Sy-*co*-TMDS)	2	73	8650	13100	1.52
13	Poly(PA-Va-*co*-Ph)	4	80	17000	33400	1.96
14	Poly(PA-Va-*co*-BDMSB)	0.25	85	10030	19300	1.93
15	Poly(PA-Va-*co*-TMDS)	0.5	71	12400	26000	2.09
16[d]	Poly(PA-Sy-*co*-Ph)	4	67	9530	16210	1.70
17	Poly(PA-Sy-*co*-BDMSB)	0.5	73	17700	33200	1.86
18	Poly(PA-Sy-*co*-TMDS)	2	78	20000	35000	1.75
19	Poly(GA-Sy-*co*-BDMSB-*r*-Ph)	2	76	15900	25100	1.57
20	Poly(GA-Sy-*co*-BDMSB-*r*-TMDS)	1	73	16200	28600	1.76

[a] All reactions were performed under argon, ester formation was carried out in THF at 50 °C, and polymerization was performed at room temperature for 24 h. [b] The time required for the completion of CHO. [c] M_n, M_w, $Đ$ of polymer determined by SEC-RI in THF calibrated with polystyrene standards at 35 °C. [d] T = 70 °C.

Degradation of PSEs

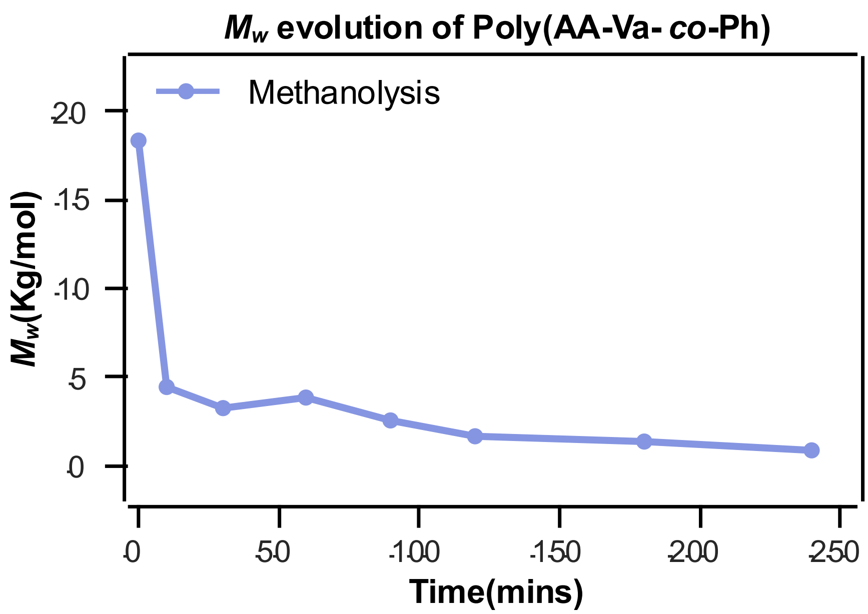

Figure S133 Evolution of the relative M_w of the **poly(AA-Va-*co*-Ph)** over time, depending on the reaction conditions.

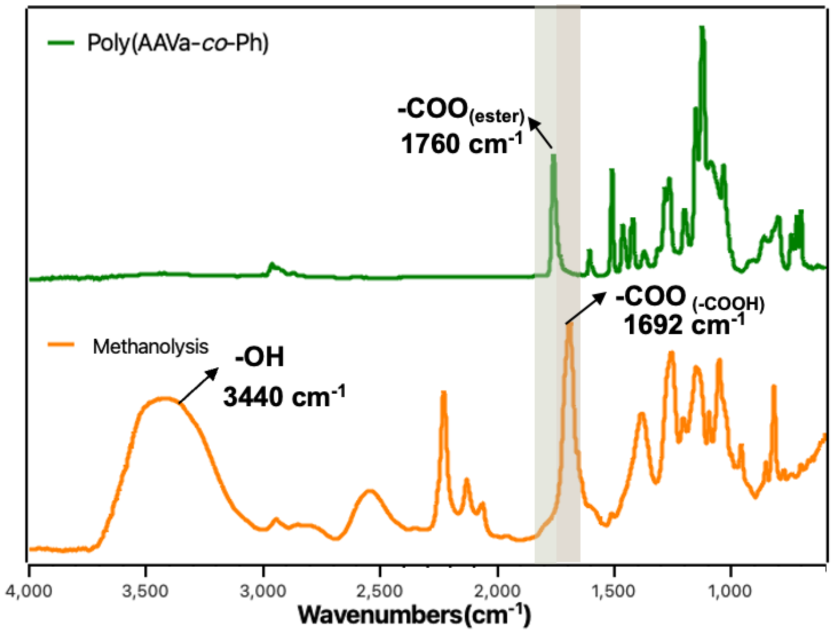

Figure S134 FT-IR spectrum of the crude mixture resulting from methanolysis of **poly(AA-Va-*co*-Ph).**

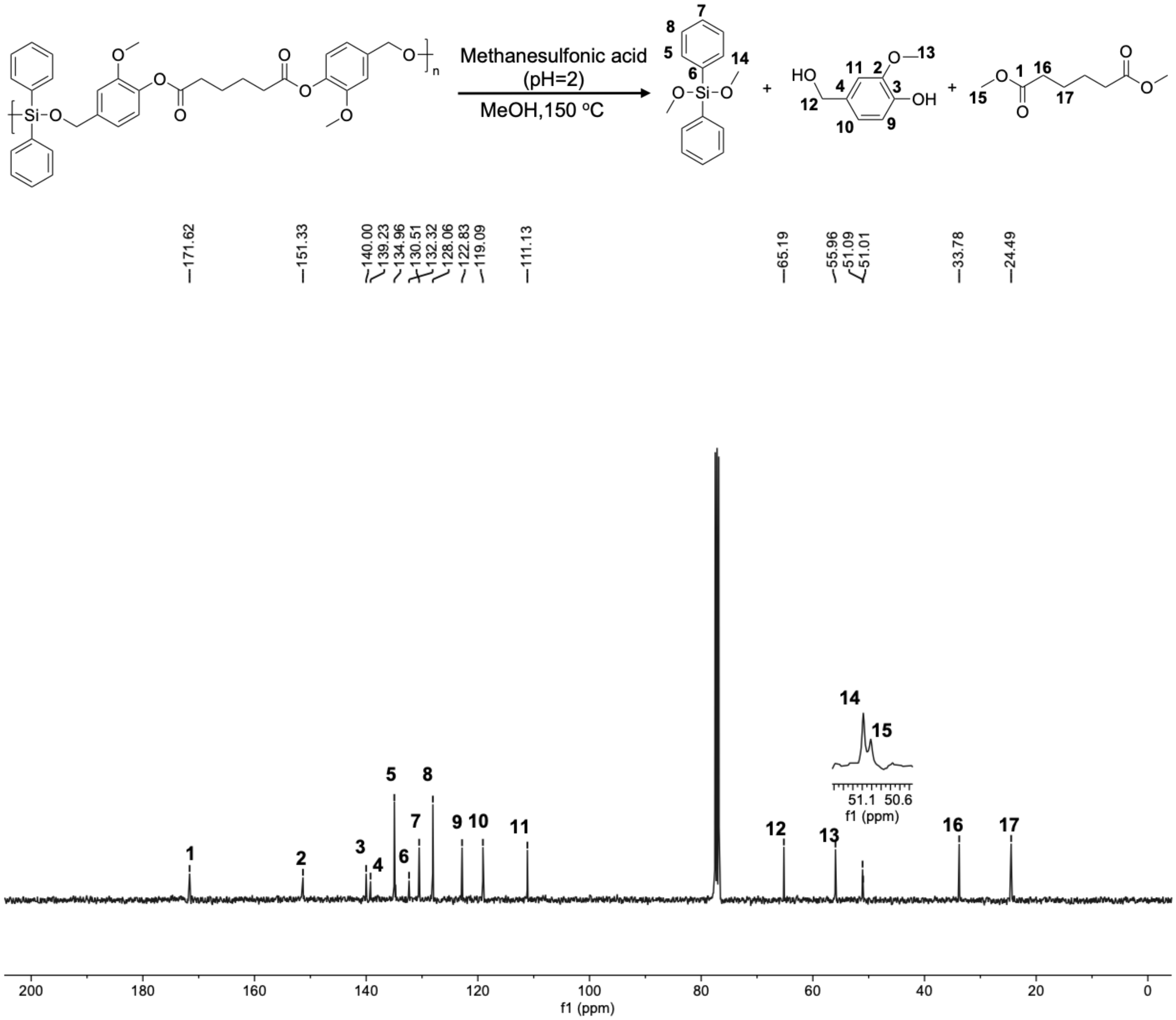

Figure S135 $^{13}C\{^{1}H\}$ NMR spectrum (101 MHz, $CDCl_3$) of degradation products of **poly(AA-Va-*co*-Ph)** after methanolysis.

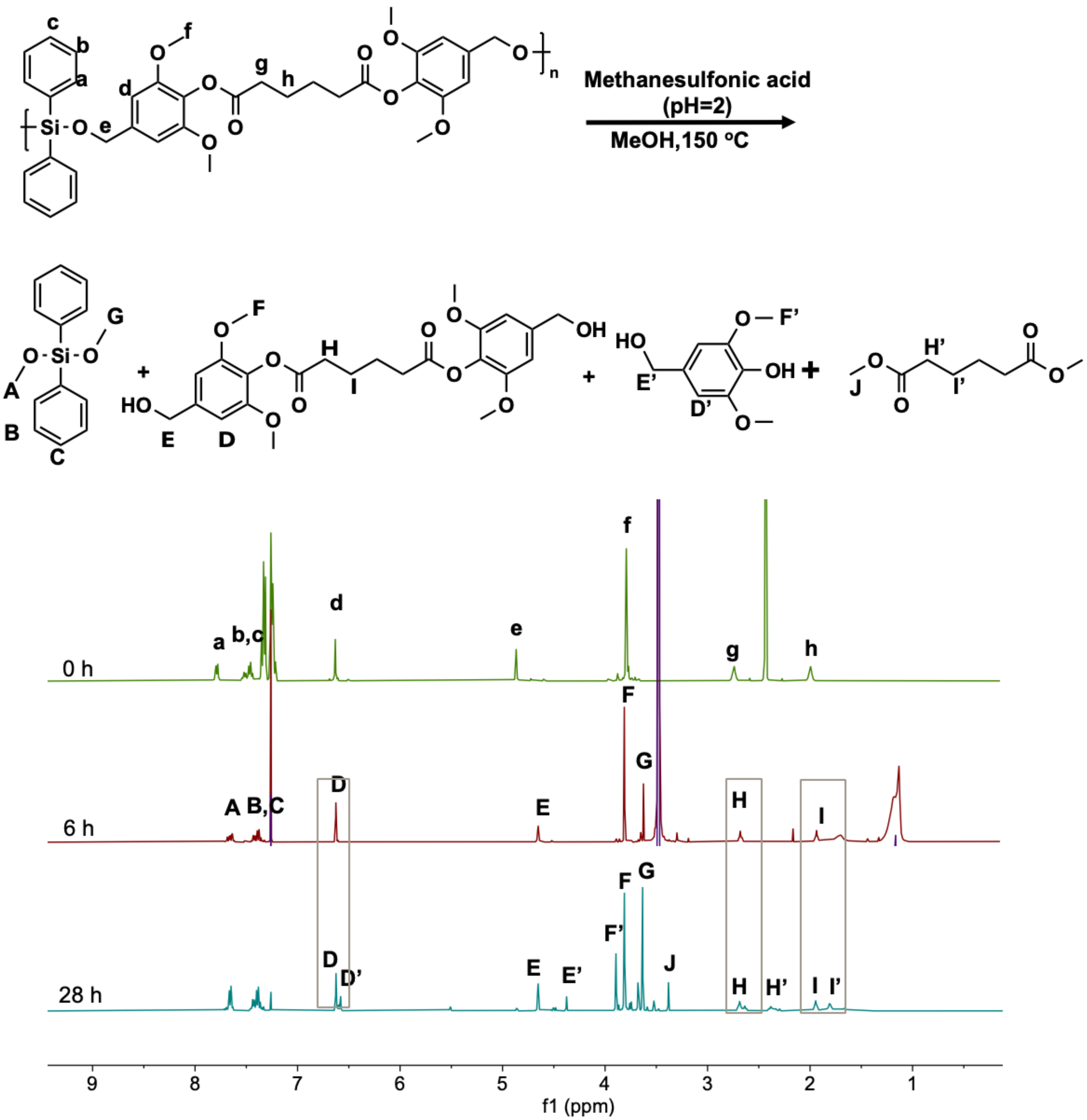

Figure S136 Acid-catalyzed methanolysis of **poly(AA-Sy-*co*-Ph)** and ^{1}H NMR spectrum (400 MHz, $CDCl_3$) of the crude mixture after 28 h.

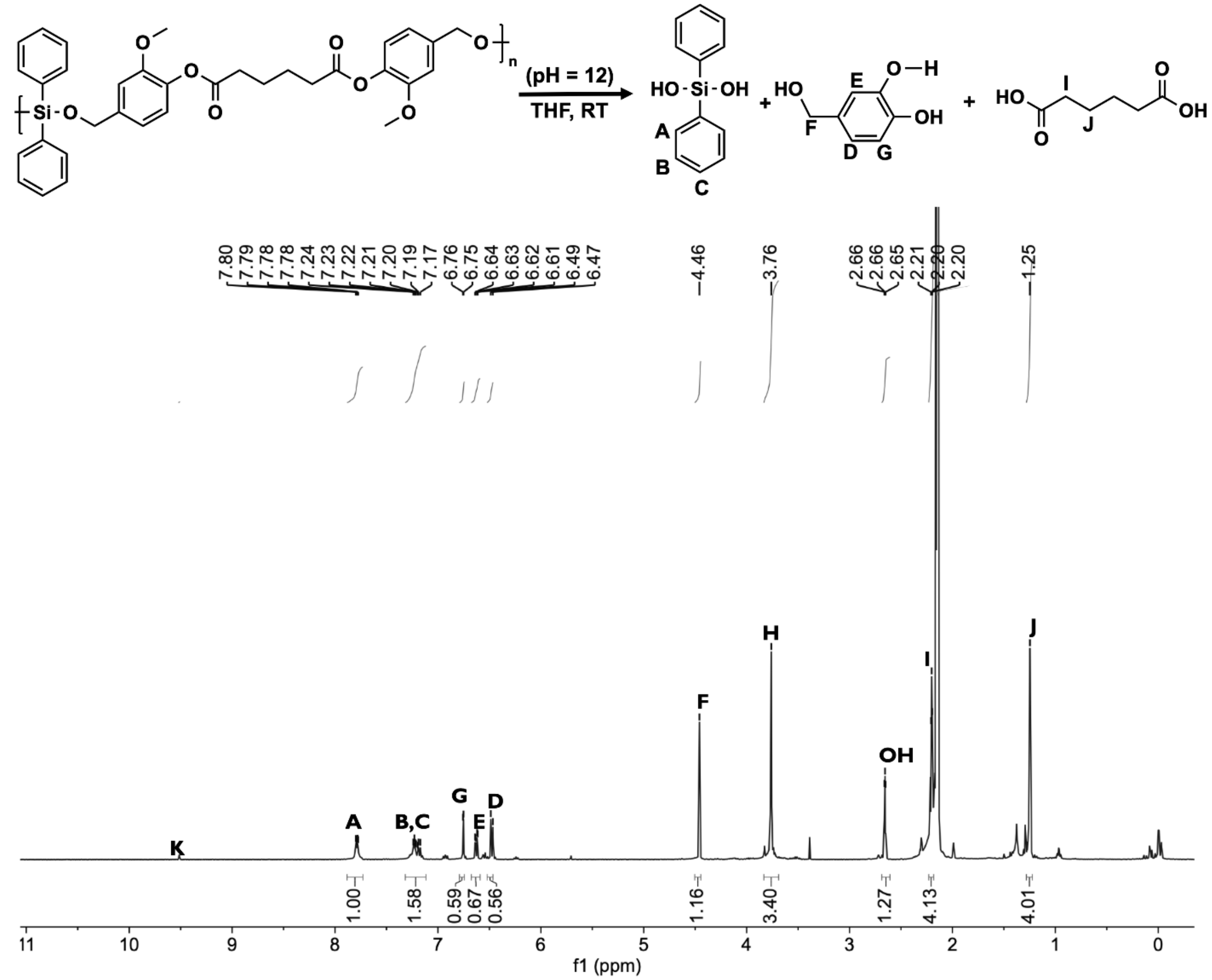

Figure S137 Base-catalyzed hydrolysis of **poly(AA-Va-*co*-Ph)** and ^{1}H NMR spectrum (400 MHz, Acetone-d_6) of the crude mixture after 7 days.

Mechanical properties of PSEs

Table S2 Summary of mechanical properties[a]

Sample	T_g[b] (°C)	$T_{-5\%}$[b] (°C)	M_w[c] (g/mol)	$Đ$[c]	E[d] [MPa]	Tensile strength [MPa]	ε[e] (%)	Toughness[f] [MJ/m^3]
Poly(GA-Sy-*co*-TMDS)	19	270	37000	1.8	0.15	0.52	3830	12.79
Poly(AA-Va-*co*-Ph)	27	269	23000	1.8	3.50	0.36	230	0.53

[a] Tensile tests were conducted at a strain rate of 15 mm/min, at 25 °C. [b] T_g of polymer determined by DSC on the second heating cycle (10 °C/min, N_2 flow). $T_{-5\%}$ of the polymer is determined by TGA (10 °C/min, N_2 flow). [c] M_w and $Đ$ of polymer determined by SEC-RI in THF calibrated with polystyrene standards at 35 °C. [d] Young's modulus. [e] Maximum engineering strain-at-break. [f] Calculated by integration of the area under stress-strain curves.

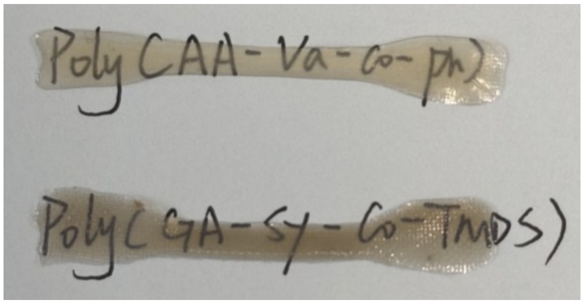

Figure S138 Pictures of **poly(GA-Sy-*co*-TMDS)** and **poly(AA-Va-*co*-Ph)**.

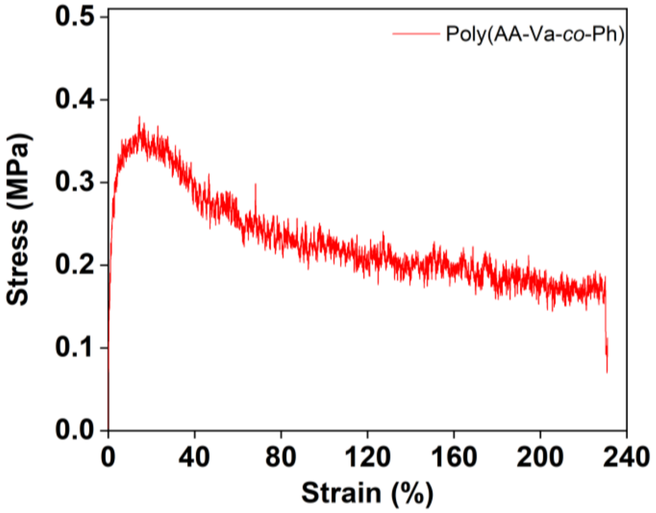

Figure S139 Stress-strain curve of **poly(AA-Va-*co*-Ph)**.

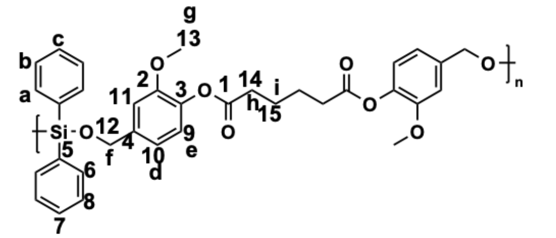

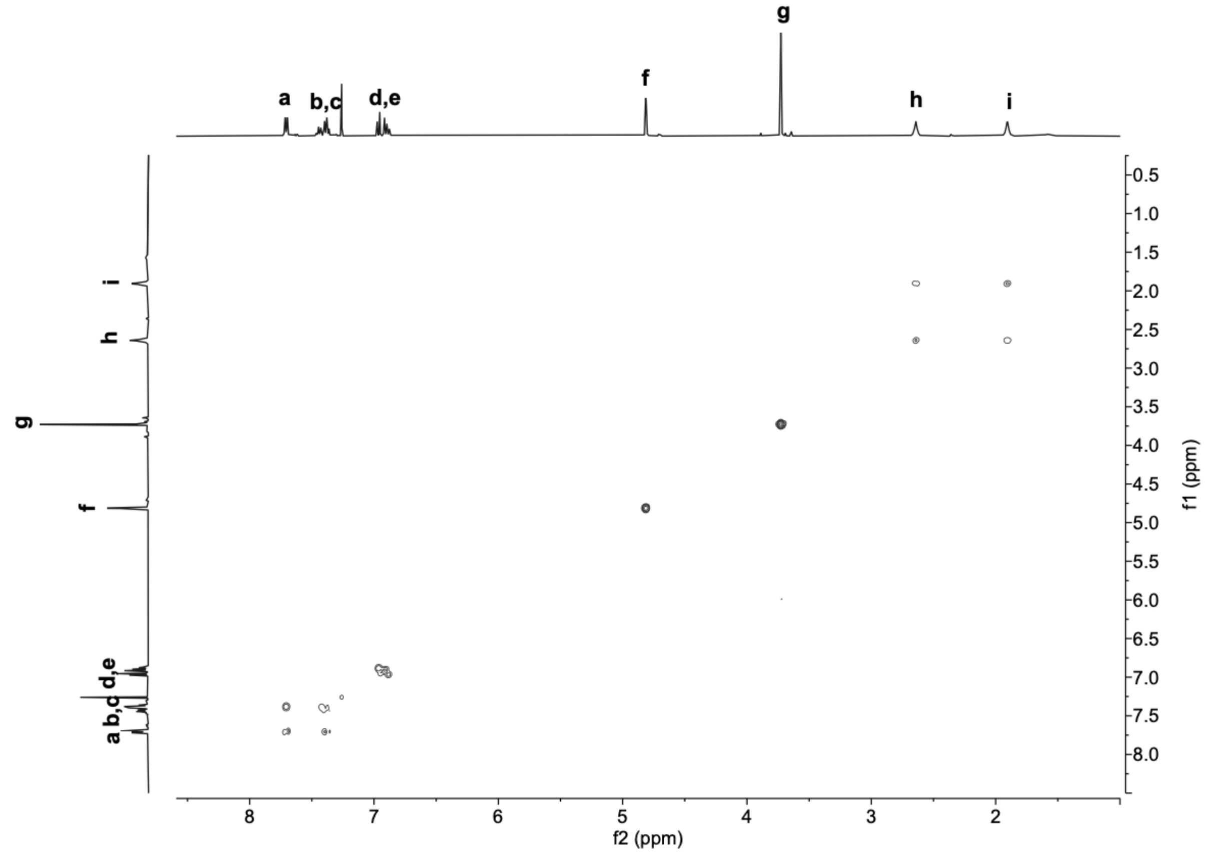

Figure S140 COSY NMR spectrum (400 MHz, $CDCl_3$) of **poly(AA-Va-*co*-Ph)** (Table S1).

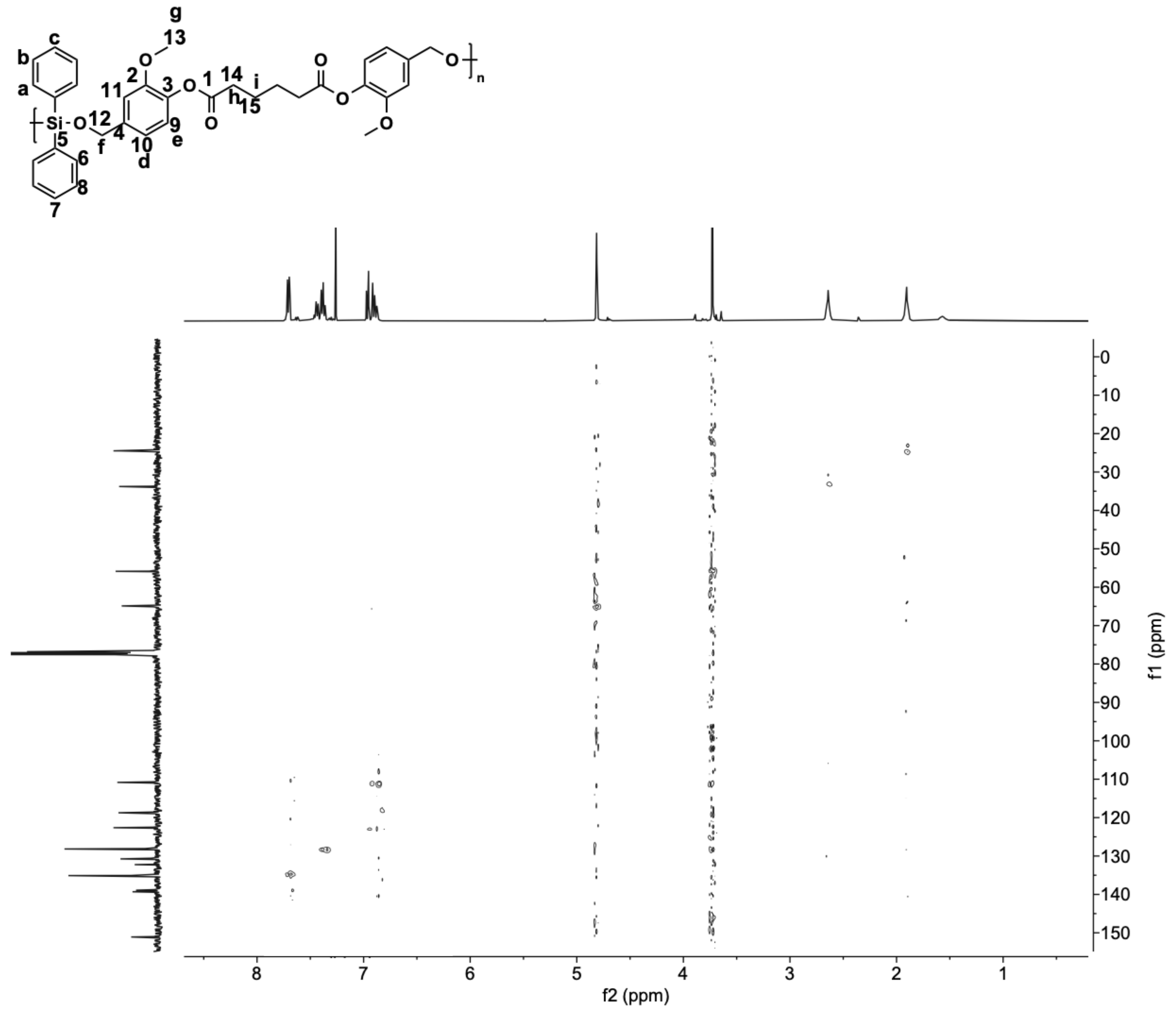

Figure S141 HSQC NMR spectrum (400 MHz, $CDCl_3$) of **poly(AA-Va-*co*-Ph)** (Table S1).

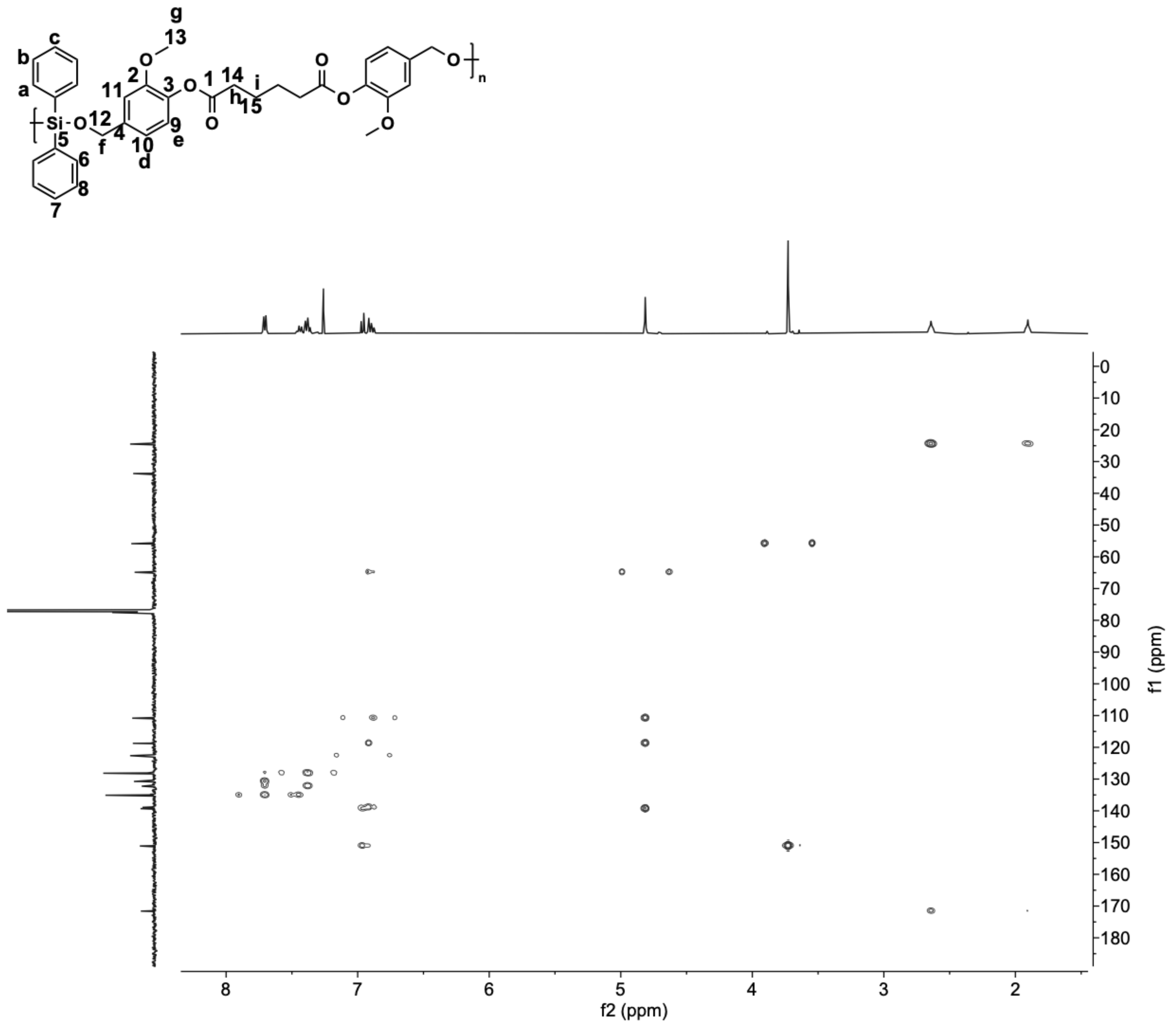

Figure S142 HMBC NMR spectrum (400 MHz, $CDCl_3$) of **poly(AA-Va-*co*-Ph)** (Table S1).

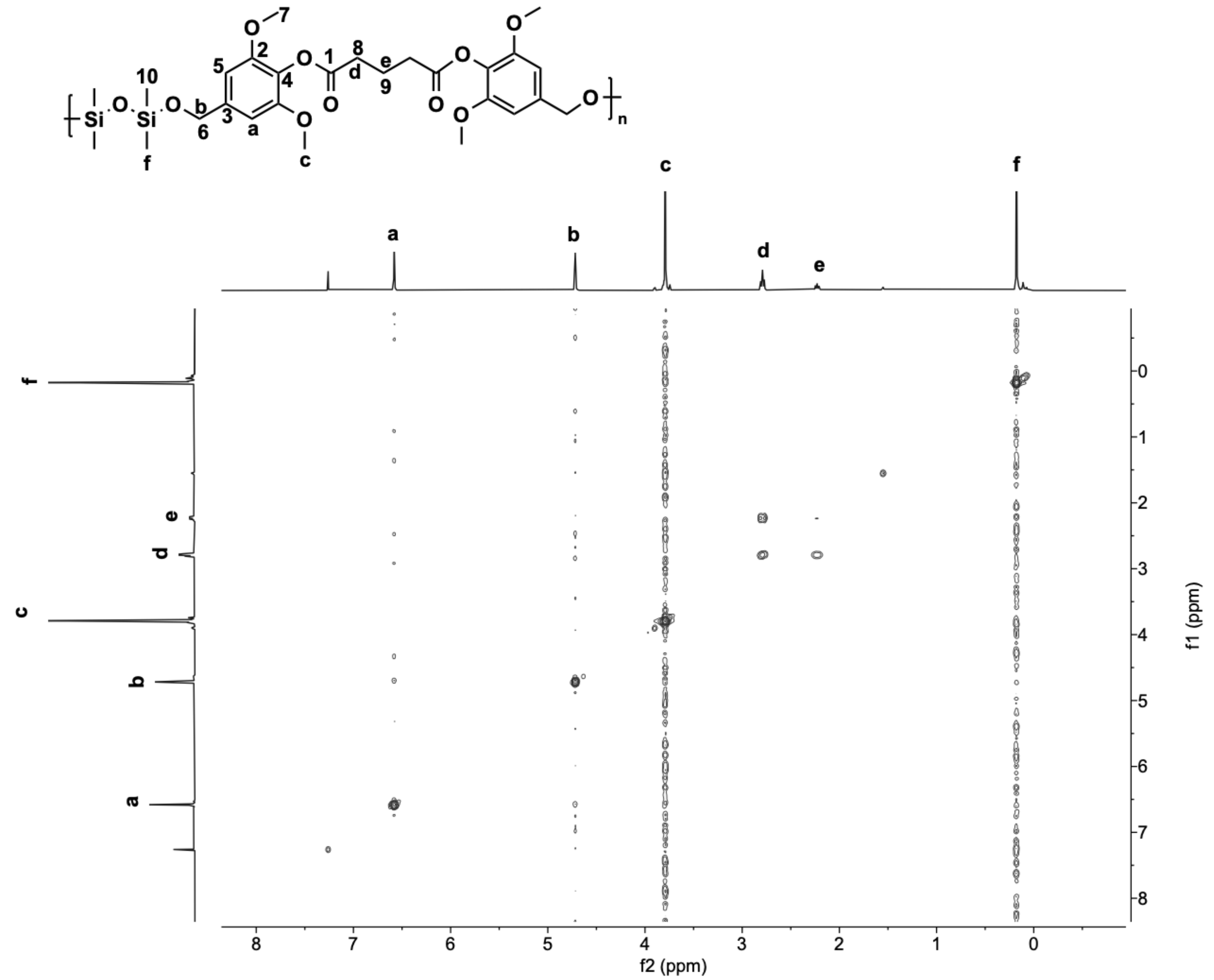

Figure S143 COSY NMR spectrum (400 MHz, $CDCl_3$) of **poly(GA-Sy-*co*-TMDS)** (Table S1).

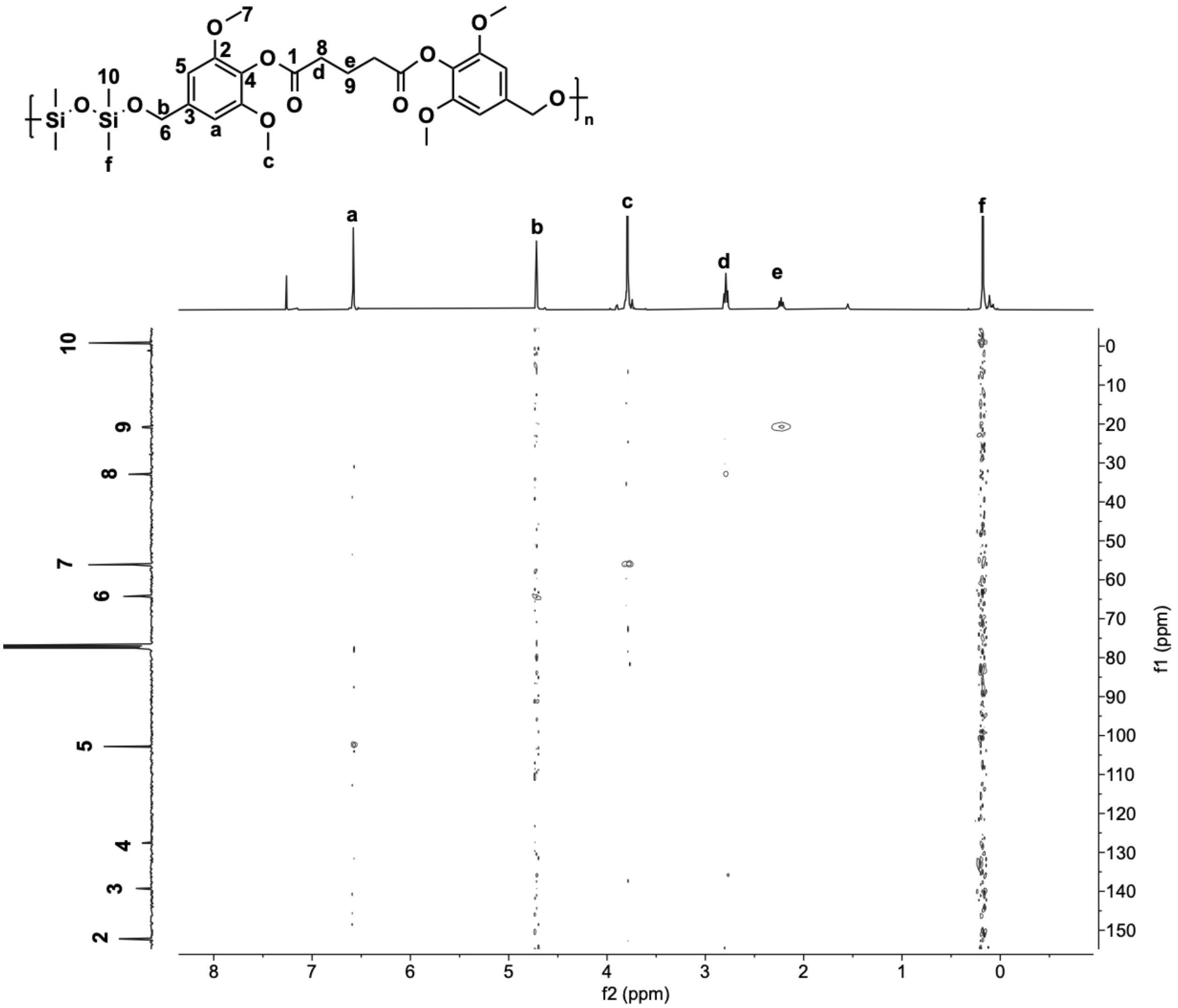

Figure S144 HSQC NMR spectrum (400 MHz, $CDCl_3$) of **poly(GA-Sy-*co*-TMDS)** (Table S1).

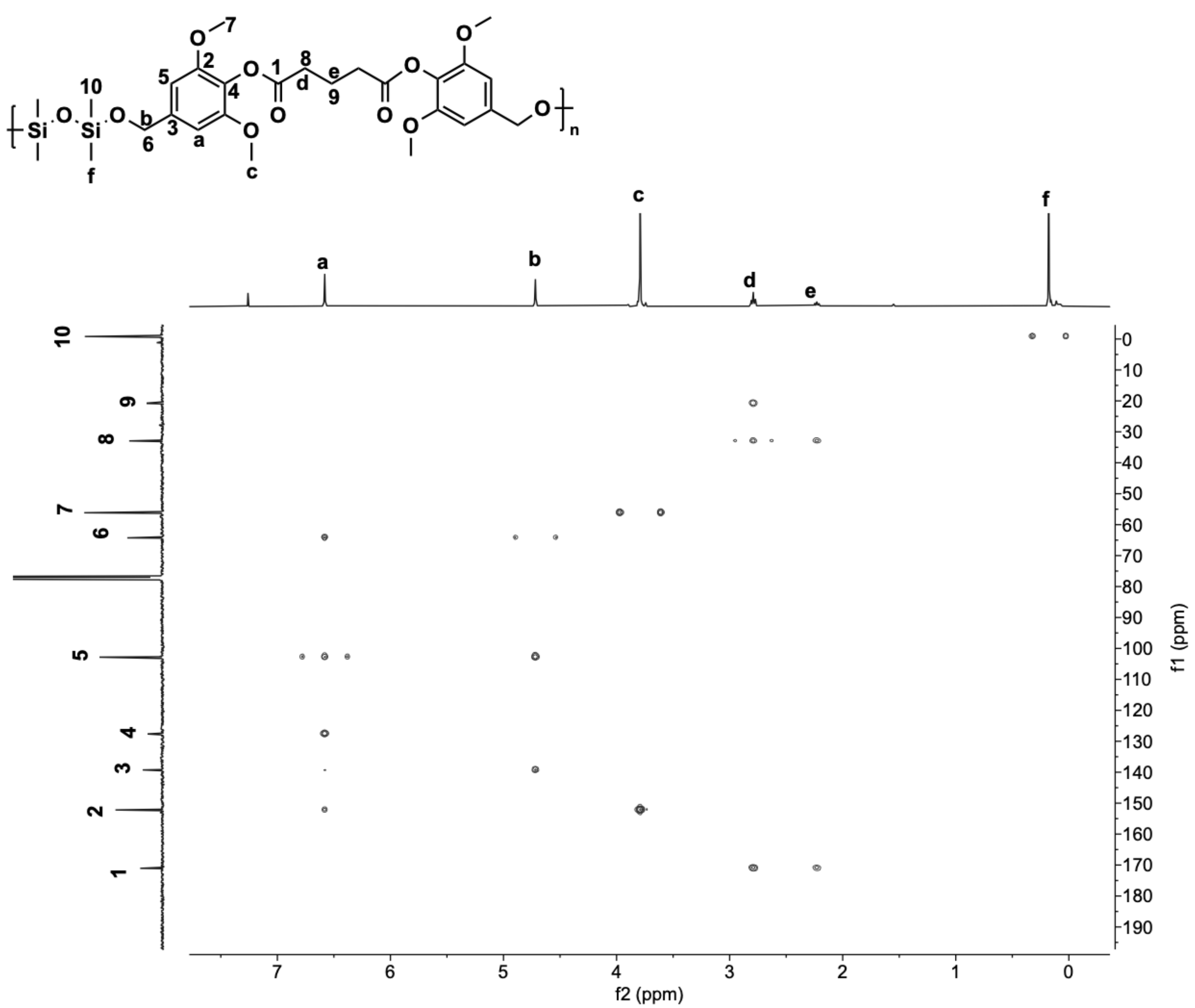

Figure S145 HMBC NMR spectrum (400 MHz, $CDCl_3$) of **poly(GA-Sy-*co*-TMDS)** (Table S1).

Table S3 Diffusion coefficients obtained from DOSY NMR spectra of PSEs.

Sample	M_n[a] (g/mol)	Diffusion coefficient[b] (x 10^{-11} m^2/s)
Poly(GA-Sy-co-TMDS)	20600	7.48
Poly(AA-Va-co-Ph)	13000	8.34

[a] Determined by SEC measurements with polystyrene as the standard in THF. [b] Diffusion coefficient obtained from the 2D DOSY NMR experiments.

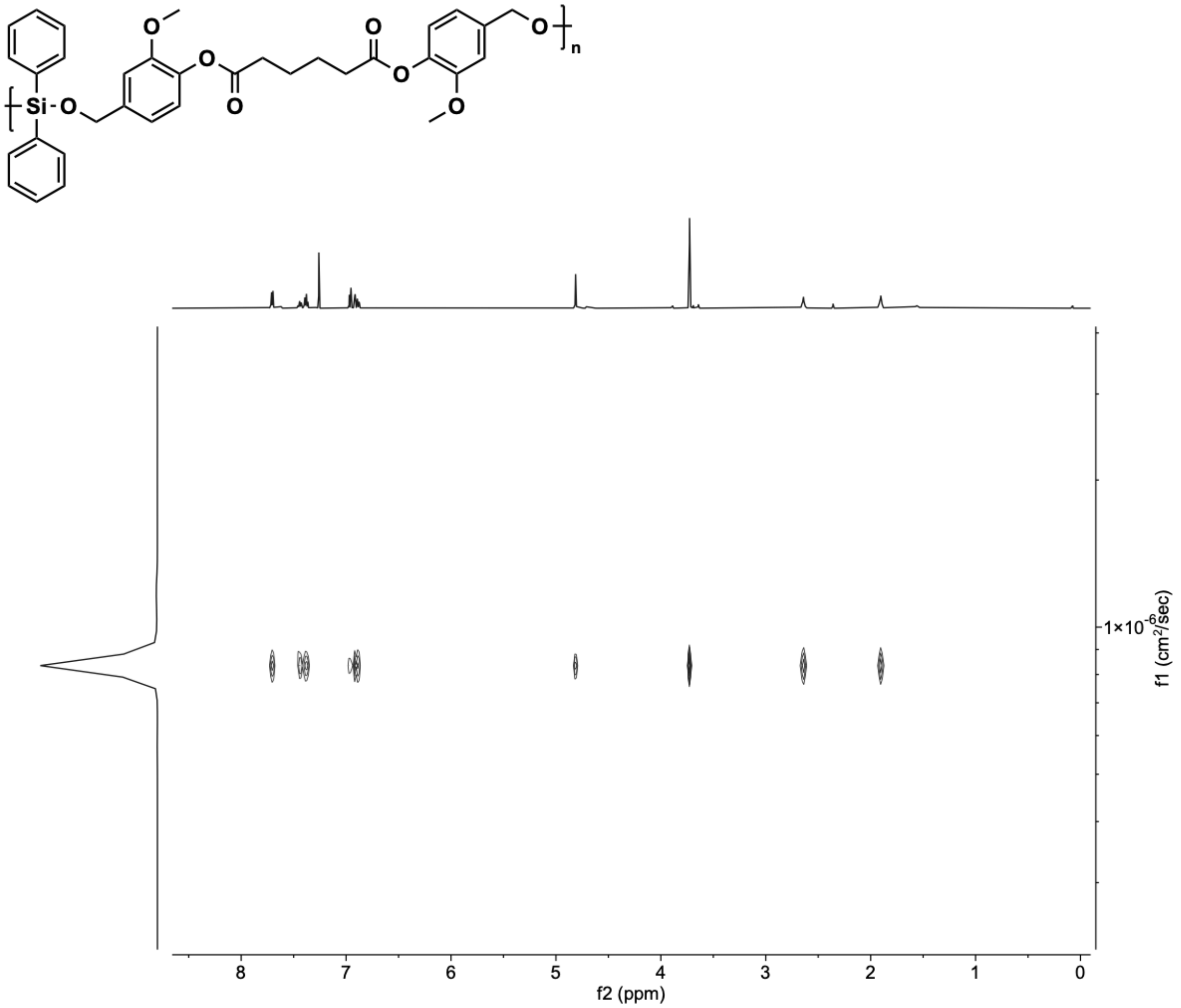

Figure S146 DOSY NMR spectrum (500 MHz, $CDCl_3$) of **poly(AA-Va-*co*-Ph)** (Table S1).

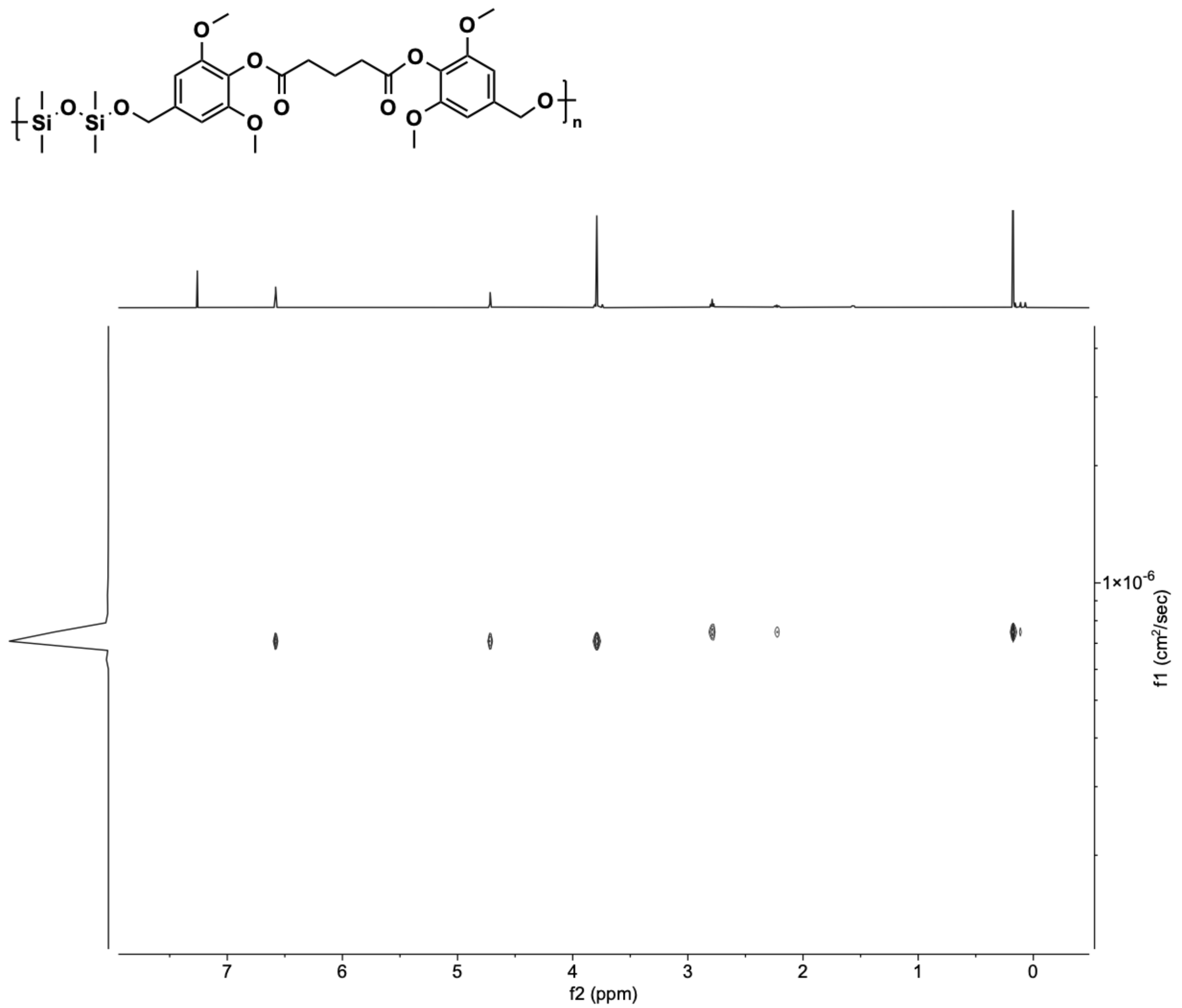

Figure S147 DOSY NMR spectrum (500 MHz, $CDCl_3$) of **poly(GA-Sy-*co*-TMDS)** (Table S1).

Table S4 Synthesis of **poly(AA-Va-*co*-Ph)** with different catalysts. [a]

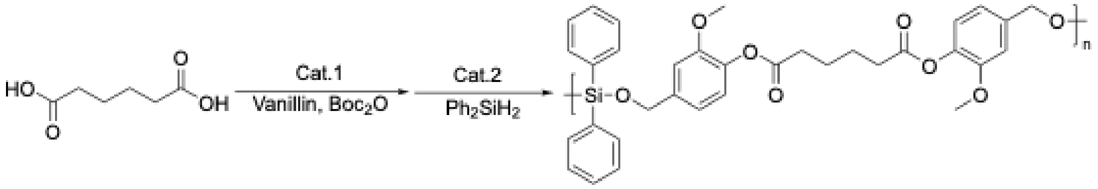

Entry	Cat. 1	Cat. 2	M_n[b] (g/mol)	M_w[b] (g/mol)	$Đ$[b]
1	TMPMgClLiCl	$B(C_6F_5)_3$	4555	6923	1.5
2	$MgCl_2$	$B(C_6F_5)_3$	6202	10519	1.6
3	*i*PrMgCl·LiCl	Zhan 1B	2751	5253	1.9

[a] All reactions were performed under argon, ester formation was carried out by 4 mol% Cat.1 in THF at 50 °C, and polymerization was performed by 1 mol% Cat.2 at room temperature for 24 h. [b] M_n, M_w and $Đ$ of polymer determined by SEC-RI in THF calibrated with polystyrene standards at 35 °C.

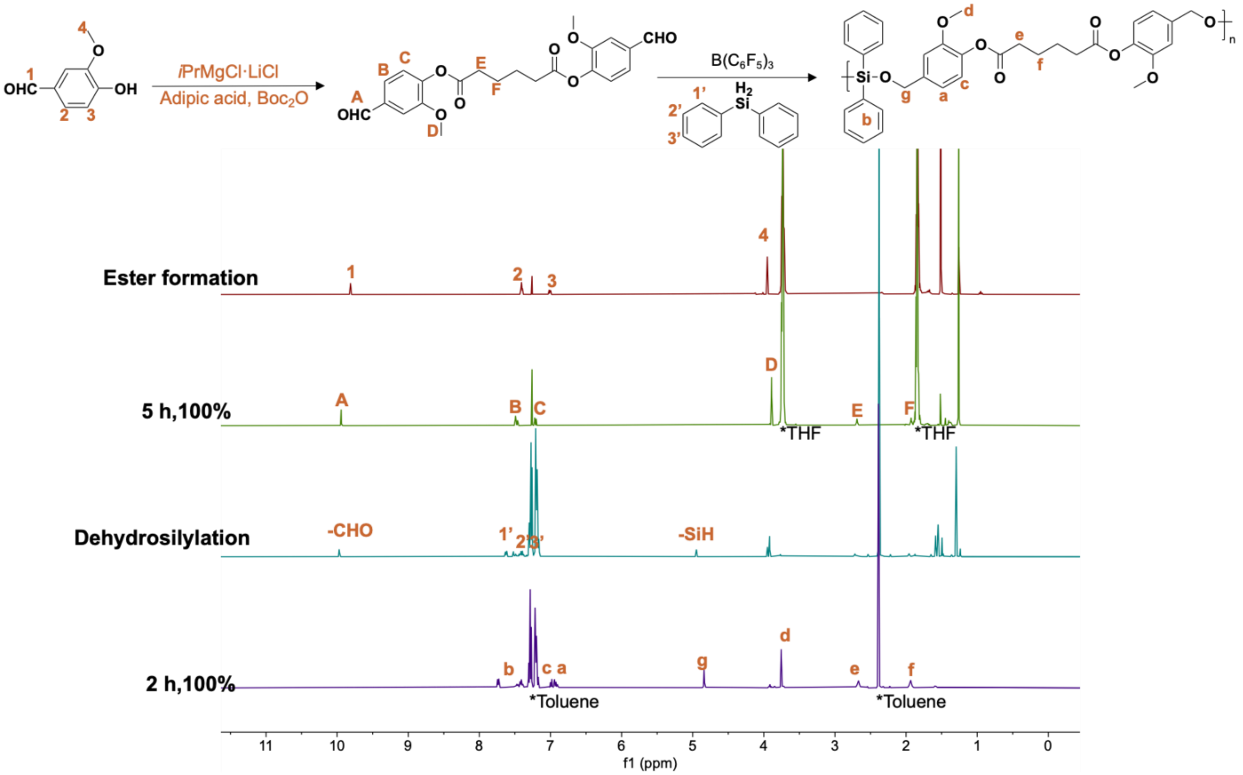

Figure S148 ^1H NMR spectra (400 MHz, $CDCl_3$) of the one-pot formation of **poly(AA-Va-*co*-Ph)** using *i*PrMgCl·LiCl and $B(C_6F_5)_3$.

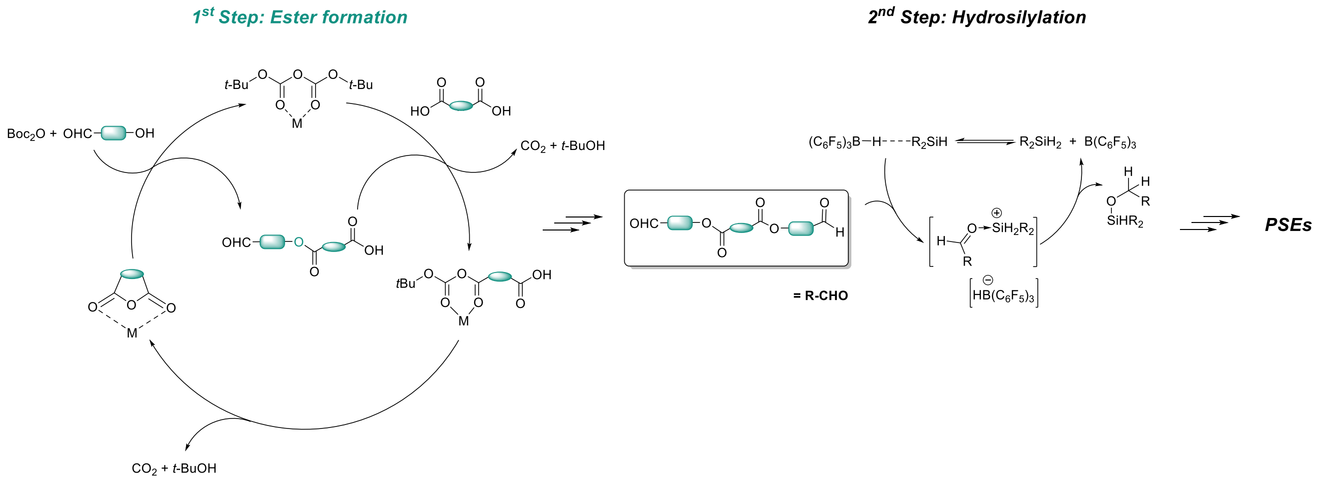

Scheme S1 Proposed mechanism for the multicatalytic sequence.
